# (P^N^C) Ligands to Stabilize Gold(III): A Straightforward Access to Hydroxo, Formate, and Hydride Complexes

**DOI:** 10.1021/acs.inorgchem.4c00788

**Published:** 2024-04-24

**Authors:** Jaime Martín, Johannes Schörgenhumer, Michał Biedrzycki, Cristina Nevado

**Affiliations:** Department of Chemistry, University of Zurich, Winterthurerstrasse 190, Zurich, CH 8057, Switzerland

## Abstract

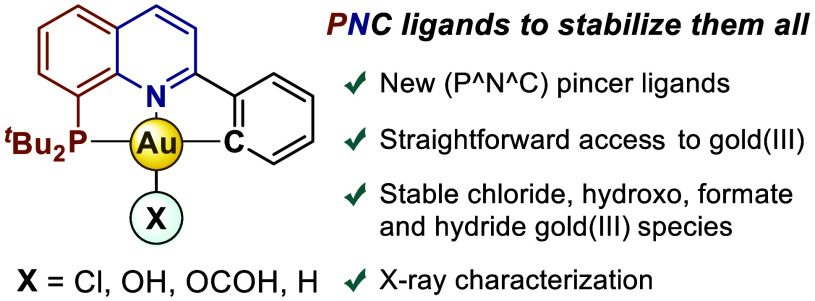

A novel class of (P^N^C) pincer ligands capable of stabilizing elusive gold(III) species is reported here. Straightforward access to (P^N^C)gold(III) hydroxo, formate, and hydride complexes has been streamlined by first incorporating a cycloauration step devoid of toxic metals or harsh conditions. The resulting gold complexes exhibit remarkable stability in solution as well as in the solid state under ambient conditions, which enabled their characterization by X-ray diffraction analyses. Interestingly, the influence of the ligand allowed the preparation of gold(III)-hydrides using mild hydride donors such as H-Bpin, which contrasts with sensitive super hydrides or strong acids and cryogenic conditions employed in previous protocols. A detailed bonding characterization of these species is complemented by reactivity studies.

## Abstract 2

This work reports a novel class of gold(III)-hydrides stabilized by newly designed (P^N^C) pincer ligands. The synthetic approach toward these species relies on a straightforward cycloauration step to incorporate the ligand to the gold center and the use of H-Bpin as a mild hydride donor. Both experimental and computational investigations unveil the protic character of the hydride and confirm the covalent nature of the Au−H bond.

## Introduction

**Scheme 1:**
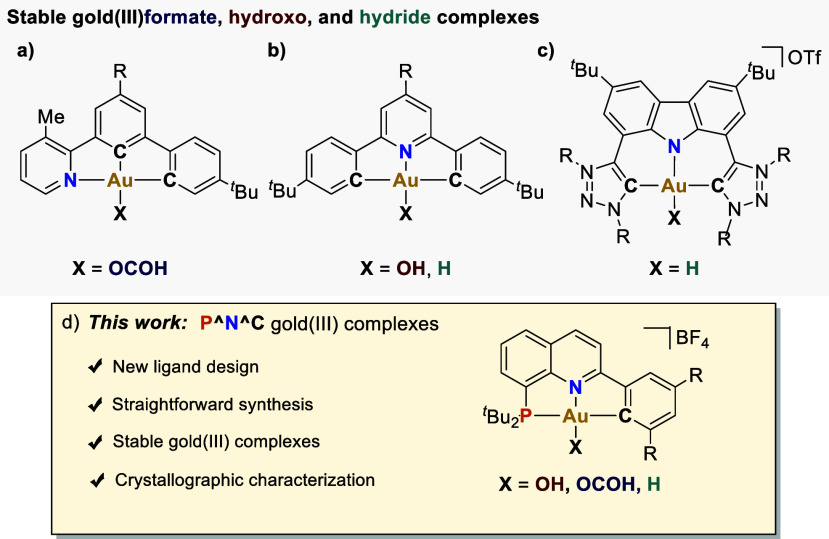
(a, b, c) Ligands Used to Stabilize Gold(III)-Formate, Hydroxo, and Hydride Complexes; (d) This Work—Stable (P^N^C)Gold(III) Complexes

Gold(III) complexes, extensively applied in material science and biomedical research,^1,2^ are prominent not only as Lewis acid catalysts^3^ but also as crucial intermediates in cross-coupling reactions involving redox catalytic cycles.^4^ With a high predisposition for reduction, gold(III) presents challenges in stability so that chelating pincer ligands, especially (N^C^C) and (C^N^C), have been employed to modify the redox properties of the metal^5^ in order to both stabilize and facilitate its detailed study (Scheme 1).^6^ Gold(III)-hydroxo, formate, and hydride complexes have been proposed as key intermediates in several transformations of societal interest such as the water–gas-shift reaction.^7^ Further, gold(III)-hydrides have also been invoked as crucial intermediates in the hydrofunctionalization of alkenes and alkynes, the dehydrogenation of formic acid, as well as β-hydride elimination processes.^8^ However, access to stable versions of these compound classes has proven challenging,^9^ likely as a result of the electronic mismatch between the strong oxidizing metal and the highly electron-rich character of the corresponding anionic counterparts.^10^ In 2017, our group reported the first gold-formate complex stabilized by a (N^C^C) ligand (Scheme 1a). Upon heating, β-hydride elimination occurred, enabling the catalytic dehydrogenation of formic acid.^11^ However, the labile nature of the hydride intermediate precluded its characterization under the reaction conditions.^12^

The seminal work by Bochmann and co-workers showcased the first isolated gold(III)-hydride using a 2,6-diphenylpyridine (C^N^C) ligand (Scheme 1b).^13^ Bezuidenhout and Bertrand disclosed a different (C^N^C)gold(III)-hydride employing a carbazole flanked by two triazole-based carbene units (Scheme 1c).^14^ In sharp contrast, gold(III)-hydrides featuring a strong *trans*-carbon σ-donor^15^ are much more labile.^11,12^ The synthesis of the above-mentioned gold(III)-hydrides requires the use of highly toxic organomercury reagents, or cryogenic conditions to template the metal center.^12−14^ Further, either sensitive complex hydrides (LiHBEt_3_, LiAlH_4_)^12,13^ or strong acids (TFA, HOTf)^14^ are needed to incorporate the hydride moiety, which restricts the synthetic accessibility to these valuable organometallic species.^16^

Sparked by these precedents, we set out to develop a new ligand template able to stabilize gold(III) enabling straightforward access to bench-stable gold(III) hydroxo, formate, and hydride species. We hypothesized that the use of a weak nitrogen donor *trans* to the anionic moiety combined with a phosphine group in the *cis* position would aid the stabilization of the corresponding complexes. Thus, a tridentate (P^N^C) ligand featuring a C(sp^2^)-anionic group in the second *cis* position was envisioned as the optimal platform to render, under mild reaction conditions, stable gold(III)-species amenable to detailed structural and reactivity profiling (Scheme 1d).

## Results and Discussion

**Figure 1:**
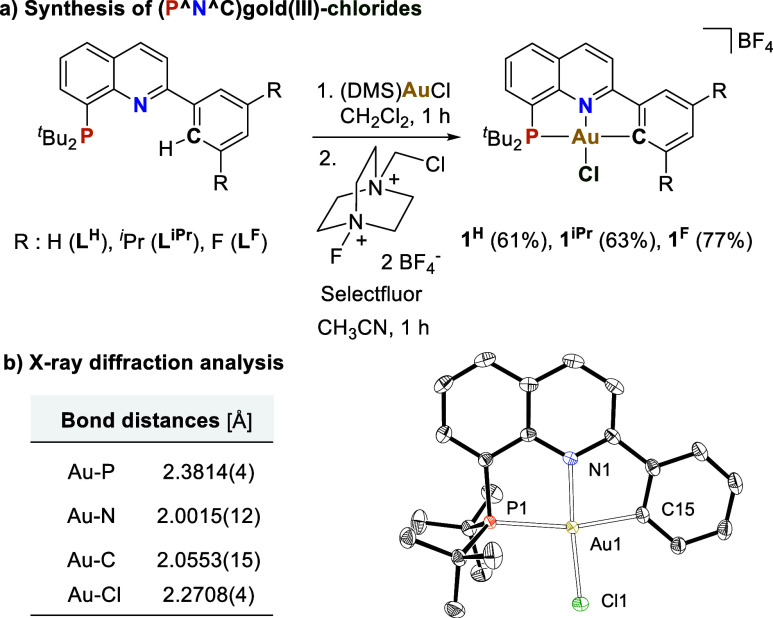
(a) Synthesis of (P^N^C)gold(III)-chloride complexes **1**^**H**^, **1**^**iPr**^, and **1**^**F**^. (b) ORTEP representation of **1**^**H**^ with 50% probability ellipsoids. Counter-anion and hydrogen atoms are omitted for clarity. Selected angles (deg) in **1**^**H**^: C15–Au1–P1 166.36(5), Cl1–Au1–N1 175.11(4).

Bidentate (P^N) ligands have demonstrated superb abilities to stabilize gold(III) complexes upon oxidative addition of gold(I) precursors^17^ and have also been extensively applied in gold(I)/gold(III) redox catalysis.^18^ The (P^N^C) ligands **L**^**H**^, **L**^**iPr**^, and **L**^**F**^ were prepared by selective Suzuki–Miyaura cross-coupling between 2,8-dibromoquinoline and the corresponding 3,5-disubstituted phenyl boronic acids, followed by a Pd-catalyzed phosphorylation step.^19,20^ Coordination of the phosphine group to Au(DMS)Cl followed by oxidation with Selectfluor^21^ triggered the *in situ* activation of the C(sp^2^)–H bond of the aryl ring producing cationic cyclometalated (P^N^C)gold(III)-chlorides **1**^**H**^, **1**^**iPr**^, and **1**^**F**^ as yellow solids in 61, 63, and 77% yields, respectively (Figure 1a). This synthetic protocol offers a simplified approach to bypass the challenging cyclometalation step in gold(III) and is unaffected by steric factors. The ^31^P{^1^H} NMR spectra of **1**^**H**^, **1**^**iPr**^, and **1**^**F**^ displayed singlets at 80.0, 80.5, and 83.3 ppm, respectively, signaling the presence of gold in a high oxidation state.^17b,17c^ X-ray diffraction analysis of **1**^**H**^ confirmed the proposed structure, which features a tetracoordinated Au center in a slightly distorted square-planar geometry (Figure 1b). Additionally, this complex displays a short Au–Cl bond distance (2.2708(4) Å) signaling also a stronger Au–Cl bond compared to (C^N^C)^22^ as well as (N^C^C), (C^N), or (P^N) counterparts.^15,17^

**Figure 2:**
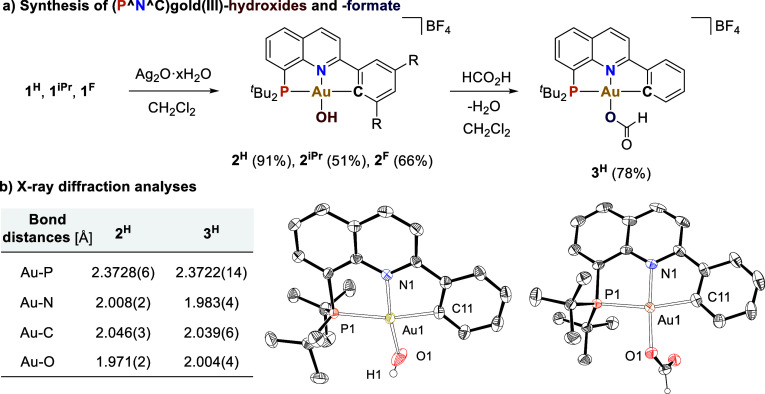
(a) Synthesis of (P^N^C)gold(III)-hydroxide (**2**^**H**^, **2**^**iPr**^, and **2**^**F**^), and formate (**3**^**H**^) complexes. (b) ORTEP representation of **2**^**H**^ and one of the two molecules in the symmetry unit of complex **3**^**H**^ with 50% probability ellipsoids. Counter-anions, solvent molecules, and hydrogen atoms (except H1 in **2**^**H**^ and H16 in **3**^**H**^) are omitted for clarity. Selected angles (deg) in **2**^**H**^: C11–Au1–P1 166.48(8), O1–Au1–N1 172.64(11). Selected angles (deg) in **3**^**H**^: C11–Au1–P1 166.97(19), O1–Au1–N1 176.21(17).

Access to the corresponding gold(III)-hydrides was attempted next. Previous studies have largely relied on the use of strong hydride donors such as LiHBEt_3_ and LiAlH_4_ under cryogenic conditions for the preparation of gold(III)-hydrides from the corresponding chloride precursors.^12,13^ Further, careful control of the stoichiometry of the reaction is needed to prevent the formation of even more labile *bis*-hydride species. However, the reactions of (P^N^C)gold(III)-chlorides **1** with these reagents resulted in mixtures of unidentified species or decomposition products. Alternatively, transition-metal hydroxides can also serve as promising synthons for introducing a variety of interesting groups, including hydrides.^9b,23^ The treatment of dichloromethane solutions of **1**^**H**^, **1**^**iPr**^, and **1**^**F**^ with moist silver oxide yielded the desired (P^N^C)gold(III)-hydroxo complexes **2**^**H**^, **2**^**iPr**^, and **2**^**F**^ in 91, 51, and 66% yields, respectively (Figure 2a). In agreement with the presence of a OH ligand *cis* to the phosphine group, the ^1^H NMR spectra in dichloromethane-*d*_2_ at 298 K exhibited doublets at 1.75 ppm (^3^*J*_HP_ = 2.8 Hz) for **2**^**H**^ and at 1.90 ppm (^3^*J*_HP_ = 2.4 Hz) for **2**^**iPr**^. Complex **2**^**F**^ showed a doublet of doublets at 4.05 ppm, because of the additional coupling with one of the fluorine substituents of the ligand (^3^*J*_HP_ = 2.2 Hz, ^5^*J*_HF_ = 17.0 Hz). Furthermore, the IR spectra contain the characteristic ν(OH) bands at 3559 cm^–1^ for **2**^**H**^, 3631 cm^–1^ for **2**^**iPr**^, and 3620 cm^–1^ for **2**^**F**^. Complex **2**^**H**^ was further characterized by X-ray diffraction analysis (Figure 2b). Interestingly, the Au–O bond distance (1.971(2) Å) is one of the shortest reported for Au(III)–OH complexes,^24^ which is consistent with the weak *trans* influence of the N atom of the quinoline group.

The reaction of complex **2**^**H**^ with 1 equiv of formic acid delivered gold(III) formate **3**^**H**^ in 78% isolated yield. The presence of the formate group was confirmed by the distinctive signals at 8.42 and 165.2 ppm in the ^1^H and ^13^C NMR spectra, respectively, as well as by the presence of the characteristic IR band at 1646 cm^–1^ for the C=O stretching. X-ray diffraction analysis further confirmed the proposed structure (Figure 2b). In contrast to our previously reported (N^C^C)gold(III)formate,^11^ complex **3**^**H**^ is stable and does not release CO_2_ even at high temperatures over long reaction times. The reduced tendency for β-hydride elimination can be attributed to the strong Au–O bond (2.004(4) Å in **3**^**H**^ versus 2.102(3) Å in the (N^C^C)gold(III)formate). This underscores the enhanced stability that the (P^N^C) ligand imparts to otherwise unstable gold(III) species.

**Figure 3:**
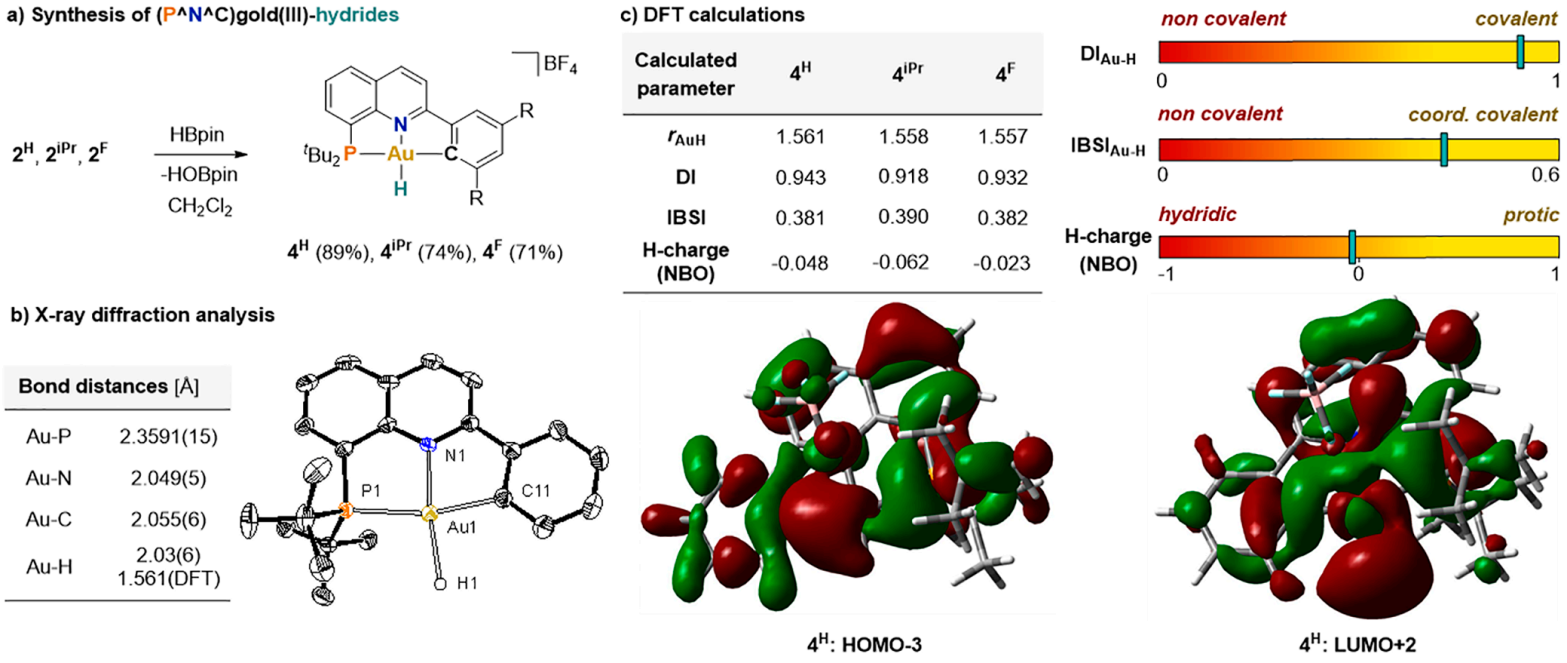
(a) Synthesis of (P^N^C)gold(III)-hydride complexes **4**^**H**^, **4**^**iPr**^, and **4**^**F**^. (b) ORTEP representation of **4**^**H**^ with 50% probability ellipsoids. Counter-anion and hydrogen atoms (except H1) are omitted for clarity. Selected angles (deg): C11–Au1–P1 165.45(18), H1–Au1–N1 172.9(18). (c) Calculated metrics for the Au–H bond analysis. Most relevant MOs of **4**^**H**^ (isosurface plots, 0.02 au). Gas-phase geometry optimizations were performed on the GD3BJ-PBE1PBE/DEF2TZVP level of theory using Gaussian 16; further analysis was performed using the MultiWFN package.^20^

Finally, we hypothesized that the thermodynamic gain associated with the formation of a stable B–O bond could be the driving force for the efficient transfer of the H atom in H-Bpin to our hydroxide precursors.^25^ Further, the use of a mild hydride donor would grant better control over the reaction conditions.^26,27^ To our delight, the reaction of **2**^**H**^, **2**^**iPr**^, and **2**^**F**^ with H-Bpin at room temperature produced (P^N^C)gold(III)-hydrides **4**^**H**^, **4**^**iPr**^, and **4**^**F**^ in 89, 74, and 71% yields, respectively (Figure 3a). The newly prepared hydrides exhibited remarkable stability in both the solid state and solution under standard ambient conditions. The ^1^H NMR spectra in dichloromethane-*d*_2_ at 298 K revealed a chemical shift of the hydride signals in the high-field region, in line with previously reported (C^N^C)gold(III)-hydrides.^12,13^ The spectra showed doublets at −5.51 ppm (^2^*J*_HP_ = 13.0 Hz) for **4**^**H**^ and at −5.18 ppm (^2^*J*_HP_ = 12.9 Hz) for **4**^**iPr**^, while they displayed a doublet of doublets at −5.60 ppm (^2^*J*_HP_ = 9.5 Hz, ^4^*J*_HF_ = 9.9 Hz) for **4**^**F**^. This behavior is in sharp contrast to gold-hydride complexes featuring strong *trans*-influence C-donor ligands, whose hydride chemical shifts appears at much lower fields.^12^

The structure of gold(III)-hydride **4**^**H**^ was confirmed by X-ray diffraction analysis (Figure 3b). Interestingly, the Au–N bond distance (2.049(5) Å) is longer than that in the chloro-**1**^**H**^ (2.0015(12) Å), hydroxo-**2**^**H**^ (2.008(2) Å), and formate-**3**^**H**^ (1.983(4) Å) analogues, which can be attributed to the stronger *trans* influence of the hydride. The H-atom was placed in the position indicated by a difference electron density map and refined together with an isotropic displacement parameter (Au–H = 2.03(6) Å; see Figure S73 in the Supporting Information (SI)). Considering the limitations in accurately determining the hydride position by crystallographic analysis, the structures of the new gold(III)-hydrides were minimized by DFT calculations.^20^ The computed Au–H bond distances of 1.561 Å for **4**^**H**^, 1.558 Å for **4**^**iPr**^, and 1.557 Å for **4**^**F**^ (Figure 3c) nicely correlate with the experimental hydride chemical shifts (Figure S75 in the SI).^20,28^

Additional insights regarding the bonding situation of the new gold(III)-hydrides could also be extracted from these calculations. As shown in Figure 3c for complex **4**^**H**^, molecular orbital (MO) analysis reveals a predominant contribution of the gold d-orbitals in the Au–H bond, indicating a strong shielding effect by the π-donation of the gold center (HOMO–3), as well as an electrophilic site (LUMO+2) at the hydride. The covalent nature of the Au–H bond in all three (P^N^C)gold(III)-hydrides is further supported by the delocalization indices (DIs) computed to be between 0.91 and 0.95. Compared to known stable Au(III)-hydrides, the values for complexes **4** were found to be in a similar range, and higher than the DI values computed for (N^C^C) and P(C^C) complexes with a stronger *trans* substituent at Au (Table S20 in the SI).^20^ A similar trend was observed for the intrinsic bond strength indices (IBSIs) of complexes **4**, which exhibited values ranging from 0.38 to 0.40, well within the range typically associated with coordinative covalent bonds. Furthermore, the natural bond orbital (NBO) partial charges at the H atoms of **4** were nearly neutral, indicating a protic rather than a hydridic nature of the H atom. These characteristics clearly distinguish complexes **4** from the more hydridic gold hydride complexes.^20^

**Figure 4:**
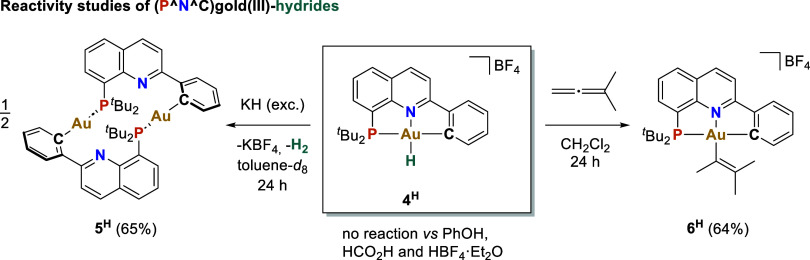
Reactivity of **4**^**H**^ with Brønsted acids, potassium hydride, and 1,1-dimethylallene.

In order to experimentally characterize the strength and reactivity of the gold(III)–H bond, complex **4**^**H**^ was treated with weak and strong Brønsted acids (Figure 4). No reaction was observed in the presence of phenol, formic, or tetrafluoroboric acid at room temperature for 24 h, indicating a non-hydridic nature of the hydride moiety. In contrast, **4**^**H**^ reacted with potassium hydride, producing molecular hydrogen and the dinuclear gold(I) compound **5**^**H**^ in 65% yield. Similar behavior was observed with the gold(III)-hydrides **4**^**iPr**^ and **4**^**F**^, although the corresponding dinuclear gold(I) species could not be isolated in pure form from the reaction mixtures. Interestingly, compound **4**^**H**^ undergoes 1,2 insertion with dimethylallene to form a gold(III) vinyl complex **6**^**H**^ in 64% isolated yield, thus highlighting the synthetic potential of these newly described gold(III) hydrides.

## Conclusions

This study presents a new pincer-type ligand class able to stabilize labile gold(III) species,^29^ exemplified by the successful synthesis and characterization of bench stable (P^N^C)gold(III)-chloro, -hydroxo, -formate, and -hydride complexes. H-Bpin was identified as a mild hydride donor able to deliver gold(III)-hydrides from the corresponding hydroxide precursors, thus circumventing the use of highly reactive lithium hydride salts, as typically required in previous protocols. Experimental and computational investigations revealed a non-hydridic nature and covalent character of the Au–H bond. Despite their protic reactivity in the presence of strong bases, insertion reactions with allenes were successfully developed. The straightforward access to these gold(III)-species opens up exciting opportunities for further research in this field, uncovering their reactivity and implications in catalysis, including the WGS reaction.

## Supporting Information Available

Experimental procedures, NMR spectra, X-ray details, and computational studies (PDF)
