# Supplementary material for: (P^N^C) Ligands to Stabilize Gold(III): A Straightforward Access to Hydroxo, Formate, and Hydride Complexes
Source: Inorg Chem. 2024 Apr 24;63(18):8390–6. doi: 10.1021/acs.inorgchem.4c00788 (PMC11080065; doi:10.1021/acs.inorgchem.4c00788)
Supplement: Supplementary file 1 — ic4c00788_si_001.pdf [file ic4c00788_si_001.pdf]

Supporting Information

(P^N^C) Ligands to Stabilize Gold(III): A Straightforward Access to Hydroxo, Formate and Hydride Complexes

*Jaime Martín, Johannes Schörgenhumer, Michal Biedrzycki, and Cristina Nevado**

Department of Chemistry, University of Zurich, Winterthurerstrasse 190, Zurich, CH 8057, Switzerland

*Corresponding author e-mail address: cristina.nevado@chem.uzh.ch

Contents	Page
1. General information and experimental methods	S2
2. General procedure for the synthesis of (P^N^C) ligands	S3
3. General procedure for the synthesis of (P^N^C)gold(III)-hydrides	S4
4. Synthesis and characterization of new compounds	S6
5. Reactivity studies	S17
6. NMR and IR spectra	S20
7. X-ray diffraction analyses	S55
8. DFT calculations	S96
9. Photophysical measurements	S129
10. References	S135

1. General information and experimental methods

Unless otherwise stated, reactions were performed under nitrogen atmosphere using Schlenk techniques and dry solvents. Commercial chemicals were used as received. Compounds 2,8-dibromoquinoline,[1] and 3,5-diisopropylphenylboronic acid[2] were prepared according to reported procedures. Flash column chromatography was performed over silica gel (230-400 mesh). NMR (^{1}H, ^{13}C, ^{19}F, ^{31}P and 2D experiments) were recorded on either AV2 400, AV2 500 or Avance Neo 500 MHz Bruker spectrometers. Chemical shifts are given in ppm. The spectra were referenced to residual solvent peaks. Multiplicities are abbreviated: singlet (s), doublet (d), triplet (t), quartet (q), septuplet (sept), multiplet (m), and broad (br). Constant couplings are given in Hz. High-resolution electrospray ionization and electronic impact mass spectrometry were performed on a Finnigan MAT 900 (Thermo Finnigan, San Jose, CA; USA) double-focusing magnetic sector mass spectrometer. Ten spectra were acquired. A mass accuracy ≤ 2 ppm was obtained in the peak matching acquisition mode by using a solution containing 2 < IPEG200, 2 < IPPG450, and 1.5 mg NaOAc (all obtained from Sigma-Aldrich, CH-Buchs) dissolved in 100 mL MeOH (HPLC Supra grade, Scharlau, E-Barcelona) as internal standard. Infrared spectra were recorded on a JASCO FT/IR-4100 spectrometer.

Safety Statement. No uncommon hazards are noted.

2. General procedure for the synthesis of (P^N^C) ligands

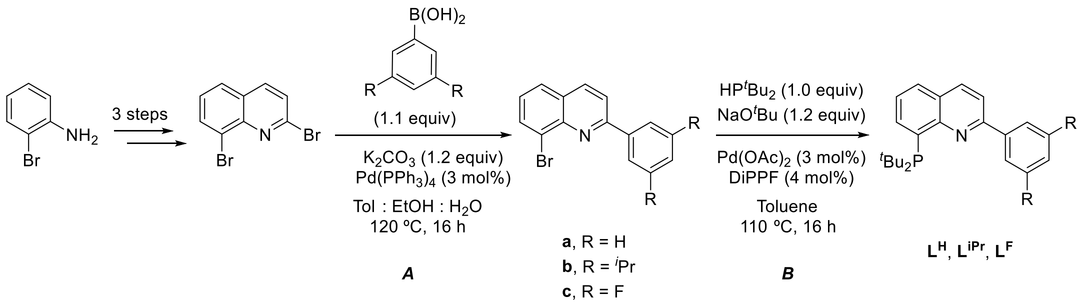

Scheme S1. Synthesis of (P^N^C) ligands $\mathbf{L^{H}}$**,** $\mathbf{L^{iPr}}$, and $\mathbf{L^{F}}$.

General procedure *A* for the synthesis of (P^N^C) precursors (a, b, c). 2,8-dibromoquinoline (1.0 equiv), the corresponding boronic acid (1.1 equiv), and K_2CO_3 (1.2 equiv) were placed in a Schlenk flask and a mixture of toluene:ethanol:water (6:1:3) was added. After degassing the system, $Pd(PPh_3)_4$ (3 mol%) was added under N_2 atmosphere and the reaction mixture was stirred at 120 ºC for 16 h. The mixture was cooled to room temperature and diluted with dichloromethane. The organic layer was washed with deionized water, dried over anhydrous $MgSO_4$, filtered, and concentrated under reduced pressure. The residue was purified by column chromatography on silica gel using cyclohexane:dichloromethane as eluent mixture.

General procedure *B* for the synthesis of (P^N^C) ligands ($\mathbf{L^{H}}$, $\mathbf{L^{iPr}}$, $\mathbf{L^{F}}$). The corresponding 8-bromo-2-arylquinoline (1.0 equiv), di-*tert*-butylphosphine (1.0 equiv), NaO*^t^*Bu (1.2 equiv), $Pd(OAc)_2$ (3 mol%), and 1,1'-bis(diisopropylphosphino)ferrocene (DiPPF) (4 mol%) were placed in a Schlenk flask under N_2 atmosphere and toluene was added. The reaction mixture was stirred at 110 ºC for 16 h. The mixture was cooled to room temperature and filtered through Celite under N_2 atmosphere. The solvent was concentrated under reduced pressure. To the resulting residue, cold methanol was added to afford a solid, which was washed with cold methanol and cold diethyl ether, and dried under vacuum.

3. General procedure for the synthesis of (P^N^C)gold(III)-hydrides

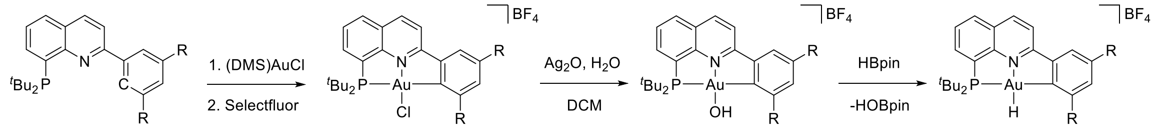

R : H (L^H), ^{i}Pr (L^{iPr}), F (L^F)   ***C***   **1^H, 1^{iPr}, 1^F**   ***D***   **2^H, 2^{iPr}, 2^F**   ***E***   **4^H, 4^{iPr}, 4^F**

Scheme S2. Synthesis of (P^N^C)gold(III)-hydrides.

General procedure *C* for the synthesis of (P^N^C)Au(III)-Cl (1^H, 1^{iPr}, 1^F). The corresponding 8-(di-*tert*-buthylphosphino)-2-arylquinoline (1.0 equiv) and chloro(dimethyl sulfide)gold(I) (1.0 equiv) were placed in a Schlenk flask under N_2 atmosphere and dichloromethane was added. The mixture was stirred at 25 °C for 1 h. After completion of the reaction, the solvent was concentrated under reduced pressure, and pentane was added to afford an off-white solid, which was washed with pentane and dried under vacuum. When the off-white solid was dried, Selectfluor (1.2 equiv) was added into the Schlenk flask followed by acetonitrile, and the resulting mixture was stirred at 25 °C for 1 h. After completion of the reaction, the solvent was concentrated under reduced pressure and subsequently diluted with dichloromethane. The resulting solution was washed with deionized water, dried over anhydrous $MgSO_4$, filtered, and concentrated under reduced pressure. The resulting crude was treated with cold diethyl ether to afford the corresponding (P^N^C)gold(III)-chloride complexes as solids, which were washed with cold diethyl ether and cold pentane and subsequently dried under vacuum.

General procedure *D* for the synthesis of (P^N^C)Au(III)-OH (2^H, 2^{iPr}, 2^F). The corresponding (P^N^C)gold(III)-chloride (1.0 equiv) and Ag_2O (10 equiv) were placed in a Schlenk flask and reagent grade (non-dry) dichloromethane with a drop of deionized water was added. The resulting mixture was stirred at 25 °C for 15 h protected from light. After completion of the reaction, the mixture was filtered through Celite and concentrated under reduced pressure. The resulting crude was treated with cold diethyl ether to afford the corresponding (P^N^C)gold(III)-hydroxide complexes as solids. These were washed with cold diethyl ether and cold pentane, and subsequently dried under vacuum. Note: the synthesis of (P^N^C)gold(III)-hydroxides from the chloride precursors was attempted via metathesis reactions with alkali metal hydroxides. However, this strategy resulted in the formation of mixtures containing both chloride and hydroxide species.[3]

General procedure *E* for the synthesis of (P^N^C)Au(III)-H (4^H, 4^{iPr}, 4^F). A solution of the corresponding (P^N^C)gold(III)-hydroxide (1.0 equiv) in dichloromethane was treated with H-Bpin (5.0 equiv) at room temperature. After completion of the reaction, the

solution was concentrated under reduced pressure and the resulting crude was treated with cold diethyl ether to afford the corresponding (P^N^C)gold(III)-hydride complex as a solid, which was washed with cold diethyl ether and cold pentane, and subsequently dried under vacuum.

4. Synthesis and characterization of new compounds

8-Bromo-2-phenylquinoline **(a)**

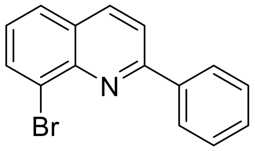

Compound **a** was synthesized following the general procedure ***A*** using phenylboronic acid (1.66 g, 13.6 mmol), 2,8-dibromoquinoline (3.56 g, 12.4 mmol), potassium carbonate (1.97 g, 14.2 mmol), and $Pd(PPh_3)_4$ (0.43 g, 0.37 mmol) in a mixture of toluene (12 mL), ethanol (2 mL), and deionized water (6 mL). Yield: 2.55 g (70%). The NMR analysis was in accordance with the previously reported data.[1]

8-Bromo-2-(3,5-diisopropyl)phenylquinoline **(b)**

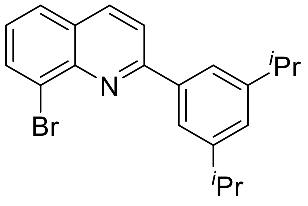

Compound **b** was synthesized following the general procedure ***A*** using 3,5-diisopropylphenylboronic acid (166 mg, 0.574 mmol), 2,8-dibromoquinoline (150 mg, 0.523 mmol), potassium carbonate (83 mg, 0.600 mmol), and $Pd(PPh_3)_4$ (18 mg, 0.016 mmol) in a mixture of toluene (1.2 mL), ethanol (0.2 mL), and deionized water (0.6 mL). Colorless oil. Yield: 146 mg (76%). ^{1}H NMR (400.13 MHz, $CDCl_3$, 298 K): δ 8.20 (d, $^3J_{HH}$ = 8.6, 1H, C*H*), 8.06 (dd, $^3J_{HH}$ = 7.5, $^4J_{HH}$ = 1.2, 1H, C*H*), 7.98-7.95 (m, 3H, C*H*), 7.79 (dd, $^3J_{HH}$ = 8.1, $^4J_{HH}$ = 1.2, 1H, C*H*), 7.36 (dd, $^3J_{HH}$ = 8.0, $^3J_{HH}$ = 7.5, 1H, C*H*), 7.22 (t, $^4J_{HH}$ = 1.6, 1H, C*H*), 3.04 (sept, $^3J_{HH}$ = 6.9, 2H, C*H*), 1.35 (d, $^3J_{HH}$ = 6.9, 12H, C*H*$_3$). ^{13}C{^{1}H} NMR (100.65 MHz, $CDCl_3$, 298 K): δ 158.6 (s, *C*), 149.7 (s, C), 145.2 (s, *C*), 139.2 (s, *C*), 137.2 (s, *C*H), 133.3 (s, *C*H), 128.5 (s, *C*), 127.4 (s, *C*H), 126.6 (s, *C*H), 126.5 (s, *C*H), 125.7 (s, *C*), 123.7 (s, *C*H), 119.8 (s, *C*H), 34.5 (s, *C*H), 24.3 (s, *C*H$_3$). (+)-HR-ESI-MS (electrospray, m/z): calcd for $C_{21}H_{23}BrN$ $[M+H]^+$, 368.10084; found, 368.10125.

8-Bromo-2-(3,5-difluoro)phenylquinoline **(c)**

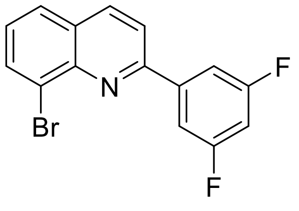

Compound **c** was synthesized following the general procedure ***A*** using 3,5-difluorophenylboronic acid (60 mg, 0.38 mmol), 2,8-dibromoquinoline (100 mg, 0.35 mmol), potassium carbonate (55 mg, 0.40 mmol), and $Pd(PPh_3)_4$ (12 mg, 0.01 mmol) in a mixture of toluene (1.2 mL), ethanol (0.2 mL), and deionized water (0.6 mL). White solid. Yield: 100 mg (90 containing <5% of residual 3,5-difluorophenylboronic acid). ^{1}H NMR (400.13 MHz, $CDCl_3$, 298 K): δ 8.26 (d, $^3J_{HH}$ = 8.6, 1H, C*H*), 8.09 (dd, $^3J_{HH}$ = 7.4, $^4J_{HH}$ = 1.3, 1H, C*H*), 7.90 (d, $^3J_{HH}$ = 8.6, 1H, C*H*), 7.87-7.84 (m, 2H, C*H*), 7.82 (dd, $^3J_{HH}$ = 8.1, $^4J_{HH}$ = 1.2, 1H, C*H*), 7.42 (dd, $^3J_{HH}$ = 8.1, $^3J_{HH}$ = 7.5, 1H, C*H*), 6.93 (tt, $^3J_{HF}$ = 8.6, $^3J_{HH}$ = 2.3, 1H, C*H*). ^{13}C{^{1}H} NMR (100.62 MHz, $CDCl_3$, 298

K): δ 163.6 (dd, $^{1}J_{CF}$ = 248.1, $^{3}J_{CF}$ = 12.6, *C*), 155.0 (t, $^{4}J_{CF}$ = 3.1, *C*), 151.2 (s, *C*), 145.1 (s, *C*), 142.4 (t, $^{3}J_{CF}$ = 9.2, *C*), 138.0 (s, *C*H), 133.9 (s, *C*H), 129.0 (s, *C*), 127.5 (s, *C*H), 127.5 (s, *C*H), 118.9 (s, *C*H), 110.7-110.5 (m, *C*H), 105.2 (t, $^{2}J_{CF}$ = 25.5, *C*H). ^{19}F{^{1}H} NMR (376.50 MHz, $CDCl_3$, 298 K): δ -109.2 (s, *F*). (+)-HR-ESI-MS (electrospray, m/z): calcd for $C_{15}H_9F_2BrN$ $[M+H]^+$, 319.98809; found, 319.98799.

P^N^C^{H} (L^{H})

8-(di-tert-Butylphosphino)-2-phenylquinoline. Compound **L^{H}** was synthesized following the general procedure ***B*** using 8-bromo-2-phenylquinoline (675 mg, 2.37 mmol), di-*tert*-butylphosphine (0.44 mL, 2.37 mmol), NaO^{t}Bu (270 mg, 2.84 mmol), Pd(OAc)$_2$ (16 mg, 0.07 mmol), and DiPPF (40 mg, 0.095 mmol) in 16 mL of toluene. Dark yellow solid. Yield: 610 mg (74%). ^{1}H NMR (400.13 MHz, CD_2Cl_2, 298 K): δ 8.29 (d, $^{3}J_{HH}$ = 7.5, 2H, H^{11} and H^{15}), 8.23-8.19 (m, 2H, H^{3} and H^{7}), 7.92 (d, $^{3}J_{HH}$ = 8.6, 1H, H^{8}), 7.86 (d, $^{3}J_{HH}$ = 8.0, 1H, H^{5}), 7.56-7.53 (m, 3H, H^{4}, H^{12}, and H^{14}), 7.47 (t, $^{3}J_{HH}$ = 7.1, 1H, H^{13}), 1.28 (d, $^{3}J_{HP}$ = 11.4, 18H, H^{17}). ^{13}C{^{1}H} NMR (100.65 MHz, CD_2Cl_2, 298 K): δ 155.9 (d, $^{4}J_{CP}$ = 2.0, C^{9}), 152.4 (d, $^{2}J_{CP}$ = 21.0, C^{1}), 140.1 (s, C^{10}), 139.0 (d, $^{1}J_{CP}$ = 29.7, C^{2}), 137.5 (d, $^{2}J_{CP}$ = 2.0, C^{3}), 137.3 (d, $^{4}J_{CP}$ = 4.8, C^{7}), 129.7 (s, C^{13}), 129.1 (s, C^{12} and C^{14}), 128.9 (s, C^{5}), 127.9 (s, C^{11} and C^{15}), 127.7 (d, ^{3}J = 2.9, C^{6}), 125.4 (s, C^{4}), 118.3 (d, $^{5}J_{CP}$ = 1.8, C^{8}), 32.7 (d, $^{1}J_{CP}$ = 26.4, C^{16}), 31.1 (d, $^{2}J_{CP}$ = 15.6, C^{17}). ^{31}P{^{1}H} NMR (161.99 MHz, CD_2Cl_2, 298 K): δ 14.1 (s, *P*). (+)-HR-ESI-MS (electrospray, m/z): calcd for $C_{23}H_{29}NP$ $[M+H]^+$, 350.20321; found, 350.20318.

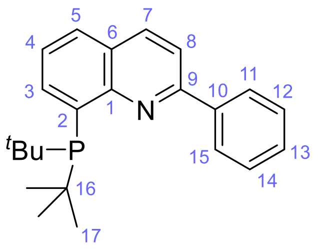

P^N^C^{iPr} (L^{iPr})

8-(di-tert-Butylphosphino)-2-(3,5-diisopropyl)phenylquinoline. Compound **L^{iPr}** was synthesized following the general procedure ***B*** using 8-bromo-2-(3,5-diisopropyl)phenylquinoline (70 mg, 0.190 mmol), di-*tert*-butylphosphine (35 µL, 0.190 mmol), NaO^{t}Bu (22 mg, 0.228 mmol), Pd(OAc)$_2$ (1.3 mg, 0.006 mmol), and DiPPF (3.2 mg, 0.008 mmol) in 1.6 mL of toluene. After filtration through celite and concentration of the solvent, a light brown oil was obtained. Further purification was not possible due to its high solubility even at -40 °C in solvents such as methanol, diethyl ether, or pentane. NMR analysis showed quantitative yield. Dark oil. Yield: 82 mg (quantitative incl. <5% residual toluene). ^{1}H NMR (400.13 MHz, CD_2Cl_2, 298 K): δ 8.20 (d, $^{3}J_{HH}$ = 8.3, 1H, H^{7}), 8.18 (ddd, $^{3}J_{HH}$ = 7.2, $^{4}J_{HH}$ = 1.2, $^{3}J_{HP}$ = 1.2, 1H, H^{3}), 7.96 (d, $^{4}J_{HH}$ = 1.7, 2H, H^{11}

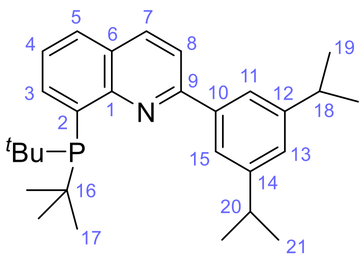

and H^{15}), 7.90 (d, $^{3}J_{HH}$ = 8.6, 1H, H^{8}), 7.85 (dd, $^{3}J_{HH}$ = 8.3, $^{4}J_{HH}$ = 1.3, 1H, H^{5}), 7.53 (dd, $^{3}J_{HH}$ = 7.7, $^{3}J_{HH}$ = 7.5, 1H, H^{4}), 7.20 (t, $^{4}J_{HH}$ = 1.4, 1H, H^{13}), 3.02 (sept, $^{3}J_{HH}$ = 6.9, 2H, H^{18} and H^{20}), 1.34 (d, $^{3}J_{HH}$ = 6.9, 12H, H^{19} and H^{21}), 1.29 (d, $^{3}J_{HP}$ = 11.2, 18H, H^{17}). $^{13}C\{^{1}H\}$ NMR (100.65 MHz, CD_2Cl_2, 298 K): δ 156.5 (d, $^{4}J_{CP}$ = 2.0, C^{9}), 152.3 (d, $^{2}J_{CP}$ = 21.3, C^{1}), 149.7 (s, C^{12} and C^{14}), 149.5 (s, C^{10}), 139.0 (d, $^{1}J_{CP}$ = 29.7, C^{2}), 137.2 (d, $^{4}J_{CP}$ = 2.0, C^{7}), 137.0 (d, $^{2}J_{CP}$ = 4.8, C^{3}), 128.8 (s, C^{5}), 127.5 (d, $^{3}J_{CP}$ = 2.9, C^{6}), 126.5 (s, C^{13}), 125.3 (s, C^{4}), 123.6 (s, C^{11} and C^{15}), 118.5 (d, $^{5}J_{CP}$ = 1.7, C^{8}), 34.7 (s, C^{18} and C^{20}), 32.6 (d, $^{1}J_{CP}$ = 26.8, C^{16}), 31.1 (d, $^{2}J_{CP}$ = 15.7, C^{17}), 24.3 (s, C^{19} and C^{21}). $^{31}P\{^{1}H\}$ NMR (161.99 MHz, CD_2Cl_2, 298 K): δ 14.9 (s, *P*). Impurities at 2.34, 7.15 and 7.24 ppm (toluene) and 7.71 ppm were found in the ^{1}H NMR spectrum. Impurities at 140.1 ppm were found in the ^{13}C NMR spectrum. (+)-HR-ESI-MS (electrospray, m/z): calcd for $C_{29}H_{41}NP$ $[M+H]^+$, 434.29711; found, 434.29621.

P^N^C^{F} (L^{F})

8-(di-tert-Butylphosphino)-2-(3,5-difluoro)phenylquinoline. Compound **L^{F}** was synthesized following the general procedure ***B*** using 8-bromo-2-(3,5-difluoro)phenylquinoline (102 mg, 0.319 mmol), di-*tert*-butylphosphine (59 µL, 0.319 mmol), NaO^{t}Bu (37 mg, 0.382 mmol), $Pd(OAc)_2$ (2 mg, 0.01 mmol), and DiPPF (5 mg, 0.012 mmol) in 3 mL of toluene. After filtration through celite and concentration of the solvent, a light brown oil was obtained. Further purification was not possible due to its high solubility even at -40 ºC in solvents such as methanol, diethyl ether, or pentane. NMR analysis showed quantitative yield. Dark oil. Yield: 123 mg (quantitative). ^{1}H NMR (500.30 MHz, CD_2Cl_2, 298 K): δ 8.25 (d, $^{3}J_{HH}$ = 8.6, 1H, H^{7}), 8.23 (d, $^{3}J_{HH}$ = 7.0, 1H, H^{3}), 7.90-7.84 (m, 4H, H^{5}, H^{8}, H^{11}, and H^{15}), 7.58 (dd, $^{3}J_{HH}$ = 7.6, $^{3}J_{HH}$ = 7.6, 1H, H^{4}), 6.93 (tt, $^{3}J_{HF}$ = 8.7, $^{3}J_{HH}$ = 2.3, 1H, H^{13}), 1.27 (d, $^{3}J_{HP}$ = 11.4, 18H, H^{17}). $^{13}C\{^{1}H\}$ NMR (125.81 MHz, CD_2Cl_2, 298 K): δ 162.9 (dd, $^{1}J_{CF}$ = 246.9, $^{3}J_{CF}$ = 13.0, C^{12} and C^{14}), 152.2-152.1 (m, C^{9}), 151.2 (d, $^{2}J_{CP}$ = 21.2, C^{1}), 142.7 (t, $^{3}J_{CF}$ = 9.1, C^{10}), 138.4 (d, $^{1}J_{CP}$ = 30.3, C^{2}), 137.1 (d, $^{2}J_{CP}$ = 1.8, C^{3}), 136.7 (d, $^{4}J_{CP}$ = 4.8, C^{7}), 127.9 (s, C^{5}), 127.1 (d, $^{3}J_{CP}$ = 2.8, C^{6}), 125.1 (s, C^{4}), 116.9 (d, $^{5}J_{CP}$ = 1.7, C^{8}), 109.8-109.5 (m, C^{11} and C^{15}), 103.7 (t, $^{2}J_{CF}$ = 25.8, C^{13}), 31.7 (d, $^{1}J_{CP}$ = 26.5, C^{16}), 30.0 (d, $^{2}J_{CP}$ = 15.6, C^{17}). $^{19}F\{^{1}H\}$ NMR (376.50 MHz, CD_2Cl_2, 298 K): δ -110.1 (s, *F*). $^{31}P\{^{1}H\}$ NMR (161.99 MHz, CD_2Cl_2, 298 K): δ 14.4 (s, *P*). (+)-HR-ESI-MS (electrospray, m/z): calcd for $C_{23}H_{27}F_2NP$ $[M+H]^+$, 386.18437; found, 386.18419.

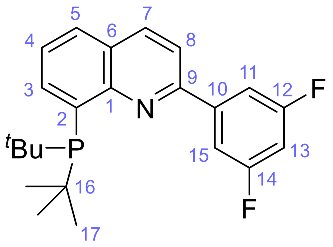

(P^N^C^{H})Au-Cl (1^{H})

[κ^{3}-P,N,C-(8-(di-tert-Butylphosphino)-2-phenylquinoline)]AuCl. Compound **1^{H}** was synthesized following the general procedure ***C*** using **L^{H}** (370 mg, 1.06 mmol) and chloro(dimethyl sulfide)gold(I) (310 mg, 1.06 mmol) in 8 mL of dichloromethane, and Selectfluor (450 mg, 1.27 mmol) in 8 mL of acetonitrile. Yellow solid. Yield: 430 mg (61%, >98% purity). ^{1}H NMR (400.13 MHz, CD_2Cl_2, 298 K): δ 9.05 (dd, $^{3}J_{HH}$ = 8.8, $^{5}J_{HP}$ = 1.4, 1H, H^{7}), 8.52-8.45 (m, 2H, H^{3} and H^{5}), 8.40 (d, $^{3}J_{HH}$ = 8.8, 1H, H^{8}), 8.10 (ddd, $^{3}J_{HH}$ = 7.8, $^{3}J_{HH}$ = 7.7, $^{4}J_{HP}$ = 1.6, 1H, H^{4}), 8.04-7.99 (m, 2H, H^{11} and H^{14}), 7.67 (dddd, $^{3}J_{HH}$ = 7.8, $^{3}J_{HH}$ = 7.5, $^{5}J_{HP}$ = 3.8, $^{4}J_{HH}$ = 1.5, 1H, H^{13}), 7.59 (dd, $^{3}J_{HH}$ = 7.5, $^{3}J_{HH}$ = 7.5, 1H, H^{12}), 1.59 (d, $^{3}J_{HP}$ = 16.9, 18H, H^{17}). ^{13}C{^{1}H} NMR (125.81 MHz, CD_2Cl_2, 298 K): δ 169.4 (d, $^{2}J_{CP}$ = 122.7, C^{15}), 169.2 (d, $^{3}J_{CP}$ = 6.0, C^{9}), 151.3 (d, $^{2}J_{CP}$ = 12.0, C^{1}), 146.8 (s, C^{10}), 146.4 (s, C^{7}), 142.5 (d, $^{2}J_{CP}$ = 1.1, C^{3}), 135.4 (d, $^{4}J_{CP}$ = 2.0, C^{5}), 134.8 (d, $^{4}J_{CP}$ = 9.2, C^{13}), 130.8 (s, C^{14}), 130.8 (d, $^{3}J_{CP}$ = 7.1, C^{6}), 130.6 (d, $^{3}J_{CP}$ = 6.5, C^{4}), 130.5 (s, C^{12}), 129.4 (d, $^{4}J_{CP}$ = 5.9, C^{11}), 125.6 (d, $^{1}J_{CP}$ = 37.1, C^{2}), 120.8 (s, C^{8}), 40.3 (d, $^{1}J_{CP}$ = 14.5, C^{16}), 29.9 (d, $^{2}J_{CP}$ = 3.3, C^{17}). ^{19}F{^{1}H} NMR (470.71 MHz, CD_2Cl_2, 298 K): δ -152.7 (br, B*F*$_4$). ^{31}P{^{1}H} NMR (202.52 MHz, CD_2Cl_2, 298 K): δ 80.0 (s, *P*). (+)-HR-ESI-MS (electrospray, m/z): calcd for $C_{23}H_{27}NAuClP$ $[M]^+$, 580.12297; found, 580.12407. Colorless crystals suitable for X-ray diffraction analysis were obtained from a concentrated solution of the compound in CH_3CN at room temperature.

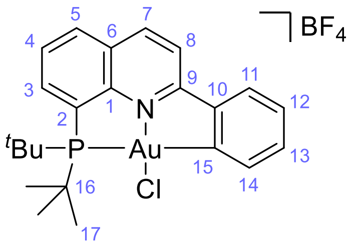

(P^N^C^{iPr})Au-Cl (1^{iPr})

[κ^{3}-P,N,C-(8-(di-tert-Butylphosphino)-2-(3,5-diisopropyl)phenylquinoline]AuCl.

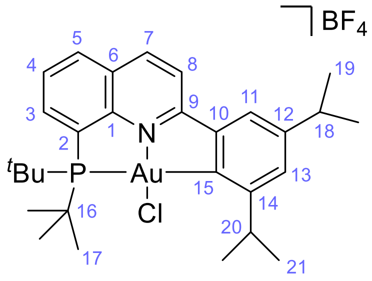

Compound **1^{iPr}** was synthesized following the general procedure ***C*** using **L^{iPr}** (82 mg, 0.190 mmol) and chloro(dimethyl sulfide)gold(I) (56 mg, 0.190 mmol) in 3 mL of dichloromethane, and Selectfluor (81 mg, 0.228 mmol) in 3 mL of acetonitrile. Light brown solid. Yield: 89.5 mg (63%).^{1}H NMR (400.13 MHz, CD_2Cl_2, 298 K): δ 8.98 (dd, $^{3}J_{HH}$ = 8.9, $^{5}J_{HP}$ = 1.6, 1H, H^{7}), 8.50-8.45 (m, 2H, H^{3} and H^{5}), 8.39 (d, $^{3}J_{HH}$ = 9.0, 1H, H^{8}), 8.08 (ddd, $^{3}J_{HH}$ = 7.8, $^{3}J_{HH}$ = 7.7, $^{4}J_{HP}$ = 1.6, 1H, H^{4}), 7.71 (dd, $^{5}J_{HP}$ = 4.3, $^{4}J_{HH}$ = 2.0, 1H, H^{11}), 7.41 (dd, $^{5}J_{HP}$ = 4.8, $^{4}J_{HH}$ = 2.1, 1H, H^{13}), 4.55 (sept, $^{3}J_{HH}$ = 6.8, 1H, H^{20}), 3.02 (sept, $^{3}J_{HH}$ = 6.8, 1H, H^{18}), 1.63 (d, $^{3}J_{HP}$ = 16.6, 18H, H^{17}), 1.33 (d, $^{3}J_{HH}$ = 7.0, 6H, H^{19}), 1.31 (d, $^{3}J_{HH}$ = 6.8, 6H, H^{21}). ^{13}C{^{1}H} NMR (100.62 MHz, CD_2Cl_2, 298 K): δ 170.0 (d, $^{3}J_{CP}$ = 5.1, C^{9}), 167.7 (d, $^{2}J_{CP}$ = 136.7, C^{15}), 157.1 (d, $^{3}J_{CP}$ = 6.8, C^{14}), 151.3 (d, $^{2}J_{CP}$ =

12.4, C^{1}), 150.8 (d, $^{5}J_{CP}$ = 1.6, C^{12}), 147.1 (d, $^{3}J_{CP}$ = 1.7, C^{10}), 145.6 (s, C^{7}), 142.5 (d, $^{2}J_{CP}$ = 2.0, C^{3}), 135.4 (d, $^{4}J_{CP}$ = 2.0, C^{5}), 133.3 (d, $^{4}J_{CP}$ = 12.3, C^{13}), 130.3 (d, $^{3}J_{CP}$ = 7.9, C^{6}), 130.2 (d, $^{3}J_{CP}$ = 6.3, C^{4}), 125.8 (d, $^{4}J_{CP}$ = 5.9, C^{11}), 124.3 (d, $^{1}J_{CP}$ = 37.4, C^{2}), 121.0 (s, C^{8}), 41.2 (d, $^{1}J_{CP}$ = 12.8, C^{16}), 34.4 (s, C^{18}), 31.1 (d, $^{4}J_{CP}$ = 1.8, C^{20}), 30.0 (d, $^{2}J_{CP}$ = 2.5, C^{17}), 25.0 (s, C^{21}), 23.8 (s, C^{19}). ^{19}F{^{1}H} NMR (376.55 MHz, CD_2Cl_2, 298 K): δ -152.9 (br, B*F*$_4$). ^{31}P{^{1}H} NMR (161.99 MHz, CD_2Cl_2, 298 K): δ 80.5 (s, *P*). (+)-HR-ESI-MS (electrospray, m/z): calcd for $C_{29}H_{39}NAuClP$ [M]$^+$, 664.21687; found, 664.21621.

(P^N^C^{F})Au-Cl (1^{F})

[κ^{3}-P,N,C-(8-(di-tert-Butylphosphino)-2-(3,5-difluoro)phenylquinoline]AuCl. Compound **1^{F}** was synthesized following the general procedure ***C*** using **L^{F}** (123 mg, 0.319 mmol) and chloro(dimethyl sulfide)gold(I) (94 mg, 0.319 mmol) in 5 mL of dichloromethane, and Selectfluor (136 mg, 0.383 mmol) in 5 mL of acetonitrile. Light brown solid. Yield: 173 mg (77%, >95% purity).^{1}H NMR (400.13 MHz, CD_2Cl_2, 298 K): δ 9.15 (dd, $^{3}J_{HH}$ = 8.8, $^{5}J_{HP}$ = 1.1, 1H, H^{7}), 8.59-8.53 (m, 2H, H^{3} and H^{5}), 8.44 (d, $^{3}J_{HH}$ = 8.7, 1H, H^{8}), 8.16 (ddd, $^{3}J_{HH}$ = 7.8, $^{3}J_{HH}$ = 7.7, $^{4}J_{HP}$ = 1.5, 1H, H^{4}), 7.72-7.67 (m, 1H, H^{11}), 7.11-7.04 (m, 1H, H^{13}), 1.64 (d, $^{3}J_{HP}$ = 17.1, 18H, H^{17}). ^{13}C{^{1}H} NMR (100.62 MHz, CD_2Cl_2, 298 K): δ 167.9-167.7 (m, C^{9}), 165.0 (dd, $^{1}J_{CF}$ = 254.3, $^{3}J_{CF}$ = 11.5, C^{14}), 163.4 (dd, $^{1}J_{CF}$ = 251.0, $^{3}J_{CF}$ = 11.6, C^{12}), 151.3 (d, $^{2}J_{CP}$ = 10.9, C^{1}), 149.5-149.1 (m, C^{10}), 147.4 (ddd, $^{2}J_{CP}$ = 112.9, $^{2}J_{CF}$ = 29.7, $^{4}J_{CF}$ = 3.8, C^{15}), 146.9 (s, C^{7}), 143.3 (s, C^{3}), 135.7 (d, $^{4}J_{CP}$ = 2.1, C^{5}), 131.2 (d, $^{3}J_{CP}$ = 7.1, C^{4}), 131.1 (d, $^{3}J_{CP}$ = 7.2, C^{6}), 125.0 (d, $^{1}J_{CP}$ = 39.0, C^{2}), 121.3 (s, C^{8}), 112.6 (ddd, $^{2}J_{CF}$ = 23.6, $^{4}J_{CP}$ = 5.6, $^{4}J_{CF}$ = 3.6, C^{11}), 111.3 (ddd, $^{2}J_{CF}$ = 31.1, $^{2}J_{CF}$ = 24.3, $^{4}J_{CP}$ = 6.7, C^{13}), 41.5 (d, $^{1}J_{CP}$ = 14.5, C^{16}), 29.8 (d, $^{2}J_{CP}$ = 2.5, C^{17}). ^{19}F{^{1}H} NMR (376.50 MHz, CD_2Cl_2, 298 K): δ -92.6 (d, $^{4}J_{FF}$ = 10.8), -107.1 (d, $^{4}J_{FF}$ = 10.8), -152.2 (br, B*F*$_4$). ^{31}P{^{1}H} NMR (161.99 MHz, CD_2Cl_2, 298 K): δ 83.3 (s, *P*). (+)-HR-ESI-MS (electrospray, m/z): calcd for $C_{23}H_{25}NAuClF_2P$ [M]$^+$, 616.10412; found, 616.10410.

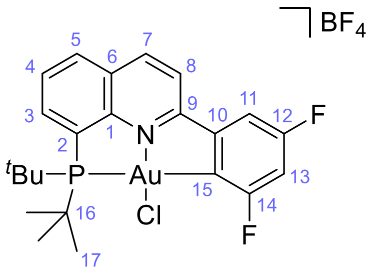

(P^N^C^{H})Au-OH (2^{H})

[κ^{3}-P,N,C-(8-(di-tert-Buthylphosphino)-2-phenylquinoline)]AuOH. Compound **2^{H}** was synthesized following the general procedure ***D*** using **1^{H}** (80 mg, 0.120 mmol) and Ag_2O (280 mg, 1.2 mmol) in 4 mL of dichloromethane. Pale yellow solid. Yield: 71.1 mg (91%). ^{1}H NMR (500.30 MHz, CD_2Cl_2, 298 K): δ 8.98 (dd, $^{3}J_{HH}$ = 8.8, $^{5}J_{HP}$ = 1.3, 1H, H^{7}), 8.45-8.41 (m, 2H, H^{3} and H^{5}), 8.36 (d, $^{3}J_{HH}$ = 8.8, 1H, H^{8}), 8.08-8.03 (m, 2H, H^{4} and

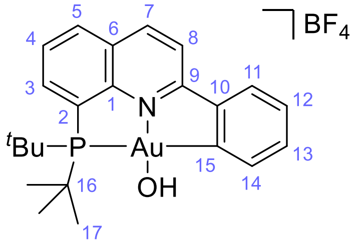

H[11]), 7.71-7.66 (m, 1H, H[13]), 7.63-7.58 (m, 2H, H[12] and H[14]), 1.75 (d, $^3J_{HP}$ = 2.8, 1H, O*H*), 1.59 (d, $^3J_{HP}$ = 16.7, 18H, H[17]). ^{13}C{^{1}H} NMR (125.82 MHz, CD_2Cl_2, 298 K): *δ* 168.1 (d, $^3J_{CP}$ = 5.8, C[9]), 165.7 (d, $^2J_{CP}$ = 123.0, C[15]), 151.4 (d, $^2J_{CP}$ = 11.9, C[1]), 146.7 (s, C[10]), 145.3 (s, C[7]), 141.7 (s, C[3]), 134.8 (d, $^4J_{CP}$ = 2.0, C[5]), 133.8 (d, $^4J_{CP}$ = 9.0, C[13]), 130.5 (d, $^3J_{CP}$ = 7.5, C[6]), 130.4 (d, $^3J_{CP}$ = 6.6, C[4]), 130.2 (s, C[12]), 129.1 (d, $^4J_{CP}$ = 5.3, C[11]), 128.4 (d, $^3J_{CP}$ = 1.5, C[14]), 126.0 (d, $^1J_{CP}$ = 37.4, C[2]), 120.4 (s, C[8]), 39.4 (d, $^1J_{CP}$ = 15.1, C[16]), 29.6 (d, $^2J_{CP}$ = 3.7, C[17]). ^{19}F{^{1}H} NMR (376.50 MHz, CD_2Cl_2, 298 K): *δ* -152.8 (br, B*F*$_4$). ^{31}P{^{1}H} NMR (202.52 MHz, CD_2Cl_2, 298 K): *δ* 72.2 (s, *P*). (+)-HR-ESI-MS (electrospray, m/z): calcd for $C_{23}H_{28}$ONAuP [M]$^+$, 562.15685; found, 562.15703. IR (ATR, cm^{-1}): *v* (OH) 3559 (br). Crystals suitable for X-ray diffraction analysis were obtained by slow diffusion of hexane into a dichloromethane solution of the complex at -30 °C.

(P^N^C^{iPr})Au-OH (2^{iPr})

[κ^3-P,N,C-(8-(di-tert-Butylphosphino)-2-(3,5-diisopropyl)quinoline)]AuOH. Compound

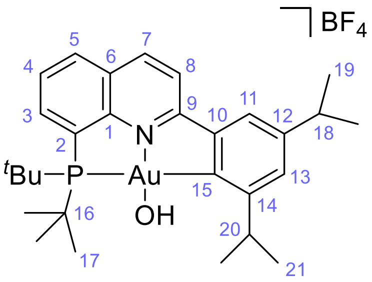

2^{iPr} was synthesized following the general procedure ***D*** using **1^{iPr}** (80 mg, 0.106 mmol) and Ag_2O (250 mg, 1.07 mmol) in 4 mL of dichloromethane. Light brown solid. Yield: 40 mg (51%). ^{1}H NMR (400.13 MHz, CD_2Cl_2, 298 K): *δ* 8.91 (dd, $^3J_{HH}$ = 8.8, $^5J_{HP}$ = 1.1, 1H, H[7]), 8.44-8.37 (m, 2H, H[3] and H[5]), 8.32 (d, $^3J_{HH}$ = 9.0, 1H, H[8]), 8.03 (ddd, $^3J_{HH}$ = 7.7, $^3J_{HH}$ = 7.7, $^4J_{HP}$ = 1.5, 1H, H[4]), 7.72 (dd, $^5J_{HP}$ = 3.5, $^4J_{HH}$ = 2.0, 1H, H[11]), 7.42 (dd, $^5J_{HP}$ = 4.3, $^3J_{HH}$ = 2.1, 1H, H[13]), 4.02 (sept, $^3J_{HH}$ = 7.0, 1H, H[20]), 3.03 (sept, $^3J_{HH}$ = 6.9, 1H, H[18]), 1.90 (d, $^3J_{HP}$ = 2.4, 1H, O*H*), 1.61 (d, $^3J_{HP}$ = 16.5, 18H, H[17]), 1.38 (d, $^3J_{HH}$ = 6.9, 6H, H[21]), 1.33 (d, $^3J_{HH}$ = 6.9, 6H, H[19]). ^{13}C{^{1}H} NMR (100.62 MHz, CD_2Cl_2, 298 K): *δ* 168.9 (d, $^3J_{CP}$ = 5.2, C[9]), 162.7 (d, $^2J_{CP}$ = 131.9, C[15]), 155.7 (d, $^3J_{CP}$ = 6.0, C[14]), 151.5 (d, $^2J_{CP}$ = 12.2, C[1]), 150.5 (d, $^5J_{CP}$ = 1.0, C[12]), 147.0 (d, $^3J_{CP}$ = 1.5, C[10]), 144.7 (s, C[7]), 141.4 (d, $^2J_{CP}$ = 1.6, C[3]), 134.9 (d, $^4J_{CP}$ = 2.1, C[5]), 131.7 (d, $^4J_{CP}$ = 10.7, C[13]), 130.1 (d, $^3J_{CP}$ = 7.6, C[6]), 130.0 (d, $^3J_{CP}$ = 6.4, C[4]), 125.2 (d, $^4J_{CP}$ = 5.4, C[11]), 124.8 (d, $^1J_{CP}$ = 37.7, C[2]), 120.5 (s, C[8]), 40.0 (d, $^1J_{CP}$ = 14.2, C[16]), 34.5 (s, C[18]), 30.2 (d, $^4J_{CP}$ = 2.1, C[20]), 29.5 (d, $^2J_{CP}$ = 3.1, C[17]), 24.6 (s, C[21]), 23.9 (s, C[19]). ^{19}F{^{1}H} NMR (376.50 MHz, CD_2Cl_2, 298 K): *δ* -152.8 (br, B*F*$_4$). ^{31}P{^{1}H} NMR (161.99 MHz, CD_2Cl_2, 298 K): *δ* 71.9 (s, *P*). (+)-HR-ESI-MS (electrospray, m/z): calcd for $C_{29}H_{40}$ONAuP [M]$^+$, 646.25075; found, 646.25011. IR (ATR, cm^{-1}): *v* (OH) 3631 (w).

(P^N^C^F)Au-OH (2^F)

[κ^3-P,N,C-(8-(di-tert-Butylphosphino)-2-(3,5-difluoro)quinoline)]AuOH. Compound **2^F** was synthesized following the general procedure ***D*** using **1^F** (95 mg, 0.135 mmol) and Ag_2O (310 mg, 1.35 mmol) in 4 mL of dichloromethane. Light brown solid. Yield: 61 mg (66%). ^{1}H NMR (400.13 MHz, CD_2Cl_2, 298 K): δ 9.08 (dd, $^3J_{HH}$ = 8.8, $^5J_{HP}$ = 1.1, 1H, H^7), 8.52-8.44 (m, 2H, H^3 and H^5), 8.36 (d, $^3J_{HH}$ = 8.8, 1H, H^8), 8.11 (ddd, $^3J_{HH}$ = 7.8, $^3J_{HH}$ = 7.7, $^4J_{HP}$ = 1.8, 1H, H^4), 7.71 (ddd, $^3J_{HF}$ = 8.5, $^5J_{HP}$ = 2.3, $^4J_{HH}$ = 2.0, 1H, H^{11}), 7.15-7.08 (m, 1H, H^{13}), 4.05 (dd, $^5J_{HF}$ = 17.0, $^3J_{HP}$ = 2.2, 1H, O*H*), 1.62 (d, $^3J_{HP}$ = 17.0, 18H, H^{17}). ^{13}C{^{1}H} NMR (100.62 MHz, CD_2Cl_2, 298 K): δ 166.3-166.1 (m, C^9), 165.3 (ddd, $^1J_{CF}$ = 240.3, $^3J_{CF}$ = 11.7, $^3J_{CP}$ = 1.2, C^{14}), 163.3 (dd, $^1J_{CF}$ = 250.6, $^3J_{CF}$ = 13.1, C^{12}), 151.4 (d, $^2J_{CP}$ = 11.0, C^1), 149.1 (ddd, $^3J_{CF}$ = 15.3, $^3J_{CF}$ = 9.4, $^3J_{CP}$ = 3.6, C^{10}), 145.9 (s, C^7), 143.6 (ddd, $^2J_{CP}$ = 125.7, $^2J_{CF}$ = 32.1, $^4J_{CF}$ = 3.9, C^{15}), 142.3 (s, C^3), 135.2 (d, $^4J_{CP}$ = 2.2, C^5), 131.1 (d, $^3J_{CP}$ = 6.8, C^4), 131.0 (d, $^3J_{CP}$ = 7.2, C^6), 125.6 (d, $^1J_{CP}$ = 39.2, C^2), 121.0 (s, C^8), 112.8 (ddd, $^2J_{CF}$ = 23.7, $^4J_{CP}$ = 5.1, $^4J_{CF}$ = 3.5, C^{11}), 110.0 (ddd, $^2J_{CF}$ = 32.3, $^2J_{CF}$ = 25.2, $^4J_{CP}$ = 5.8, C^{13}), 40.3 (d, $^1J_{CP}$ = 15.9, C^{16}), 29.3 (d, $^2J_{CP}$ = 3.1, C^{17}). ^{19}F{^{1}H} NMR (376.50 MHz, CD_2Cl_2, 298 K): δ -103.2 (dd, $^4J_{FF}$ = 9.8, $^4J_{FP}$ = 3.2, F^{14}), -107.3 (d, $^4J_{FF}$ = 9.8, F^{12}), -152.4 (br, B*F*$_4$). ^{31}P{^{1}H} NMR (161.99 MHz, CD_2Cl_2, 298 K): δ 74.6 (d, $^4J_{FP}$ = 3.2, *P*). (+)-HR-ESI-MS (electrospray, m/z): calcd for $C_{23}H_{26}ONAuF_2P$ [M]$^+$, 598.13801; found, 598.13737. IR (ATR, cm^{-1}): *ν* (OH) 3620 (w).

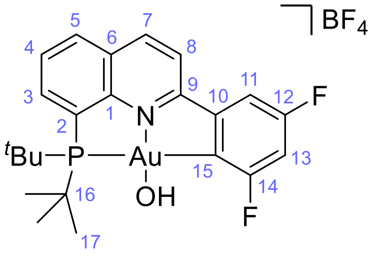

(P^N^C^H)Au-OCOH (3^H)

[κ^3-P,N,C-(8-(di-tert-Butylphosphino)-2-phenylquinoline)]Au-formate. A solution of **2^H** (17 mg, 0.026 mmol) in 1 mL of dichloromethane was treated with 1 equiv of formic acid at room temperature. After 30 min, the solvent was concentrated, and the resulting residue was washed with Et_2O (3 x 1 mL) and pentane (2 x 1 mL) to afford a solid, which was dried under reduced pressure. Yield 14 mg (78%). ^{1}H NMR (400.13 MHz, CD_2Cl_2, 298 K): δ 9.04 (dd, $^3J_{HH}$ = 8.8, $^5J_{HP}$ = 1.3, 1H, H^7), 8.48-8.44 (m, 2H, H^3 and H^5), 8.42 (d, $^4J_{HP}$ = 1.6, 1H, H^{18}), 8.36 (d, $^3J_{HH}$ = 8.9, 1H, H^8), 8.10 (ddd, $^3J_{HH}$ = 7.8, $^3J_{HH}$ = 7.7, $^4J_{HP}$ = 1.4, 1H, H^4), 8.00-7.97 (m, 1H, H^{11}), 7.63-7.56 (m, 2H, H^{12} and H^{13}), 7.38-7.33 (m, 1H, H^{14}), 1.57 (d, $^3J_{HP}$ = 16.9, 18H, H^{17}). ^{13}C{^{1}H} NMR (100.62 MHz, CD_2Cl_2, 298 K): δ 169.7 (d, $^3J_{CP}$ = 5.8, C^9), 167.3 (d, $^2J_{CP}$ = 116.5, C^{15}), 165.2 (s, C^{18}), 151.4 (d, $^2J_{CP}$ = 11.9, C^1), 146.6 (s, C^7), 145.9 (d, $^3J_{CP}$ = 1.3, C^{10}), 142.2 (d, $^2J_{CP}$ = 1.1, C^3), 135.3 (d, $^4J_{CP}$ = 2.2,

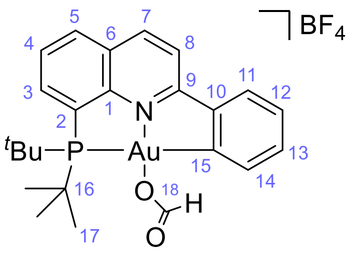

C^5), 134.6 (d, $^4J_{CP}$ = 9.3, C^{13}), 130.7 (d, $^3J_{CP}$ = 6.5, C^4), 130.6 (s, C^{12}), 130.4 (d, $^3J_{CP}$ = 2.3, C^{14}), 130.4 (d, $^3J_{CP}$ = 7.2, C^6), 128.9 (d, $^4J_{CP}$ = 5.5, C^{11}), 124.6 (d, $^1J_{CP}$ = 38.7, C^2), 120.6 (s, C^8), 39.8 (d, $^1J_{CP}$ = 14.5, C^{16}), 29.6 (d, $^2J_{CP}$ = 3.8, C^{17}). $^{31}P\{^1H\}$ NMR (161.99 MHz, CD_2Cl_2, 298 K): δ 79.3 (s, *P*). (+)-HR-ESI-MS (electrospray, m/z): calcd for $C_{24}H_{28}AuNO_2P$ $[M]^+$, 590.15179; found, 590.15158. IR (ATR, cm^{-1}): *v* (OCOH) 1646 (s). In order to confirm the structure of the cation, colorless crystals suitable for X-ray diffraction analysis were obtained by slow diffusion of pentane into a dichloromethane solution of the complex with BAr^F as counterion at 4 °C.

(P^N^C^H)Au-H (4^H)

[κ^3-P,N,C-(8-(di-tert-Butylphosphino)-2-phenylquinoline)]AuH. Compound **4^H** was synthesized following the general procedure ***E*** using **2^H** (102 mg, 0.157 mmol), HBpin (114 µL, 0.786 mmol) in 2 mL of dichloromethane for a period of 1 h. Pale yellow solid. Yield 88 mg (89%). ^1H NMR (400.13 MHz, CD_2Cl_2, 298 K): δ 8.89 (dd, $^3J_{HH}$ = 8.9, $^5J_{HP}$ = 1.2, 1H, H^7), 8.40-8.35 (m, 2H, H^3 and H^5), 8.33 (d, $^3J_{HH}$ = 8.8, 1H, H^8), 8.11-8.06 (m, 1H, H^{11}), 8.06-8.00 (m, 2H, H^4 and H^{14}), 7.57-7.53 (m, 2H, H^{12} and H^{13}), 1.52 (d, $^3J_{HP}$ = 16.9, 18H, H^{17}), -5.51 (d, $^2J_{HP}$ = 13.0, 1H, Au*H*). $^{13}C\{^1H\}$ NMR (100.62 MHz, CD_2Cl_2, 298 K): δ 166.8 (d, $^3J_{CP}$ = 5.6, C^9), 162.5 (d, $^2J_{CP}$ = 110.9, C^{15}), 150.8 (d, $^2J_{CP}$ = 11.5, C^1), 149.1 (s, C^{10}), 144.2 (s, C^7), 141.4 (d, $^2J_{CP}$ = 1.2, C^3), 139.8 (d, $^3J_{CP}$ = 3.0, C^{14}), 134.8 (d, $^4J_{CP}$ = 8.4, C^{13}), 134.2 (d, $^4J_{CP}$ = 2.1, C^5), 130.4 (d, $^3J_{CP}$ = 6.9, C^6), 130.1 (d, $^3J_{CP}$ = 6.7, C^4), 129.0 (d, $^1J_{CP}$ = 38.9, C^2), 128.9 (s, C^{11}), 128.8 (s, C^{12}), 120.0 (s, C^8), 38.5 (d, $^1J_{CP}$ = 17.8, C^{16}), 30.1 (d, $^2J_{CP}$ = 4.6, C^{17}). $^{19}F\{^1H\}$ NMR (470.71 MHz, CD_2Cl_2, 298 K): δ -152.7 (br, BF_4). $^{31}P\{^1H\}$ NMR (161.99 MHz, CD_2Cl_2, 298 K): δ 85.8 (s, *P*). Impurities at 1.61 and 1.24 ppm were found in the ^1H NMR spectrum. Impurities at 66.0 and 15.5 ppm (diethyl ether) were found in the ^{13}C NMR spectrum. (+)-HR-ESI-MS (electrospray, m/z): calcd for $C_{23}H_{28}NAuP$ $[M]^+$, 546.16196; found, 546.16198. Crystals suitable for X-ray diffraction analysis were obtained by slow diffusion of pentane into a dichloromethane solution of the complex at -30 °C.

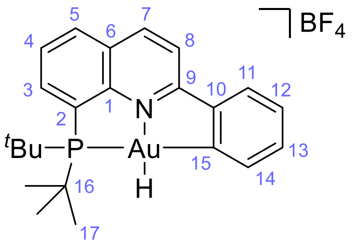

(P^N^C^{iPr})Au-H (4^{iPr})

[κ^3-P,N,C-(8-(di-tert-Butylphosphino)-2-(3,5-diisopropyl)quinoline)]AuH. Compound **4^{iPr}** was synthesized following the general procedure ***E*** using **2^{iPr}** (78 mg, 0.106 mmol), HBpin (77 µL, 0.531 mmol) in 1 mL of dichloromethane for a period of 24 h. Light brown solid. Yield: 56 mg (74%). ^{1}H NMR (400.13 MHz, CD_2Cl_2, 298 K): δ 8.87 (dd, $^3J_{HH}$ = 8.9, $^5J_{HP}$ = 1.4, 1H, H^7), 8.39-8.35 (m, 2H, H^3 and H^5), 8.33 (d, $^3J_{HH}$ = 8.9, 1H, H^8), 8.02 (ddd, $^3J_{HH}$ = 7.8, $^3J_{HH}$ = 7.8, $^4J_{HP}$ = 1.7, 1H, H^4), 7.79-7.77 (m, 1H, H^{11}), 7.48-7.45 (m, 1H, H^{13}), 3.25 (sept, $^3J_{HH}$ = 6.9, 1H, H^{20}), 3.03 (sept, $^3J_{HH}$ = 6.9, 1H, H^{19}), 1.52 (d, $^3J_{HP}$ = 16.8, 18H, H^{17}), 1.36 (d, $^3J_{HH}$ = 6.9, 6H, H^{21}), 1.34 (d, $^3J_{HH}$ = 6.9, 6H, H^{19}), -5.18 (d, $^2J_{HP}$ = 12.9, 1H, Au*H*). ^{13}C{^{1}H} NMR (100.62 MHz, CD_2Cl_2, 298 K): δ 167.6 (d, $^3J_{CP}$ = 5.6, C^9), 165.8 (d, $^2J_{CP}$ = 109.2, C^{15}), 153.4 (d, $^3J_{CP}$ = 4.0, C^{14}), 151.2 (d, $^5J_{CP}$ < 1.0, C^{12}), 150.8 (d, $^2J_{CP}$ = 11.7, C^1), 149.5 (s, C^{10}), 143.7 (s, C^7), 141.2 (d, $^2J_{CP}$ = 1.2, C^3), 134.2 (d, $^4J_{CP}$ = 2.1, C^5), 130.2 (d, $^3J_{CP}$ = 7.1, C^6), 129.9 (d, $^3J_{CP}$ = 6.8, C^4), 128.9 (d, $^4J_{CP}$ = 8.4, C^{13}), 128.8 (d, $^1J_{CP}$ = 36.6, C^2), 125.1 (d, $^4J_{CP}$ = 5.1, C^{11}), 120.1 (s, C^8), 44.3 (d, $^4J_{CP}$ = 2.9, C^{20}), 38.5 (d, $^1J_{CP}$ = 17.8, C^{16}), 34.8 (s, C^{18}), 30.2 (d, $^2J_{CP}$ = 4.5, C^{17}), 24.5 (s, C^{21}), 24.0 (s, C^{19}). ^{19}F{^{1}H} NMR (376.50 MHz, CD_2Cl_2, 298 K): δ -152.7 (br, B*F*$_4$). ^{31}P{^{1}H} NMR (161.99 MHz, CD_2Cl_2, 298 K): δ 86.1 (s, *P*). (+)-HR-ESI-MS (electrospray, m/z): calcd for $C_{29}H_{40}NAuP$ [M]$^+$, 630.25586; found, 630.25641.

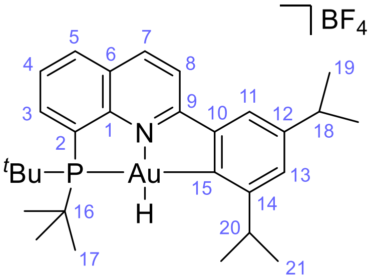

(P^N^C^F)Au-H (4^F)

[κ^3-P,N,C-(8-(di-tert-Butylphosphino)-2-(3,5-difluoro)quinoline)]AuH. Compound **4^F** was synthesized following the general procedure ***E*** using **2^F** (29 mg, 0.042 mmol), HBpin (30 µL, 0.207 mmol) in 0.5 mL of dichloromethane for a period of 24 h. Light brown solid. Yield: 20 mg (71%, >95% purity). ^{1}H NMR (500.30 MHz, CD_2Cl_2, 298 K): δ 9.01 (dd, $^3J_{HH}$ = 8.8, $^5J_{HP}$ = 1.2, 1H, H^7), 8.47-8.43 (m, 2H, H^3 and H^5), 8.34 (d, $^3J_{HH}$ = 8.9, 1H, H^8), 8.11 (ddd, $^3J_{HH}$ = 7.8, $^3J_{HH}$ = 7.7, $^4J_{HP}$ = 1.6, 1H, H^4), 7.69 (ddd, $^3J_{HF}$ = 8.6, $^5J_{HP}$ = 2.2, $^4J_{HH}$ = 2.1, 1H, H^{11}), 7.20-7.15 (m, 1H, H^{13}), 1.54 (d, $^3J_{HP}$ = 17.2, 18H, H^{17}), -5.60 (dd, $^4J_{HF}$ = 9.9, $^2J_{HP}$ = 9.5, 1H, Au*H*). ^{13}C{^{1}H} NMR (125.81 MHz, CD_2Cl_2, 298 K): δ 165.1-164.9 (m, C^9), 164.1 (dd, $^1J_{CF}$ = 249.7, $^3J_{CF}$ = 11.1, C^{12}), 163.3 (ddd, $^1J_{CF}$ = 244.7, $^3J_{CF}$ = 11.6, $^3J_{CP}$ = 2.0, C^{14}), 151.6 (ddd, $^3J_{CF}$ = 14.5, $^3J_{CF}$ = 9.0, $^3J_{CP}$ = 1.5, C^{10}), 150.6 (d, $^2J_{CP}$ = 10.6, C^1), 144.9 (s, C^7), 143.2 (ddd, $^2J_{CP}$ = 108.1, $^2J_{CF}$ = 46.0, $^4J_{CF}$ = 4.2, C^{15}), 142.0 (s, C^3), 134.6 (d, $^4J_{CP}$ = 2.1, C^5), 130.9 (d, $^3J_{CP}$ = 7.2, C^6), 130.8 (d, $^3J_{CP}$ = 7.2, C^4), 129.1 (d,

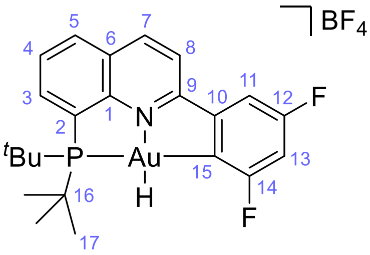

$^{1}J_{CP}$ = 37.8, C^{2}), 120.4 (s, C^{8}), 112.5 (ddd, $^{2}J_{CF}$ = 23.7, $^{4}J_{CP}$ = 4.6, $^{4}J_{CF}$ = 3.6, C^{11}), 108.3 (ddd, $^{2}J_{CF}$ = 32.5, $^{2}J_{CF}$ = 24.8, $^{4}J_{CP}$ = 4.9, C^{13}), 39.0 (d, $^{1}J_{CP}$ = 18.8, C^{16}), 30.2 (d, $^{2}J_{CP}$ = 4.2, C^{17}). ^{19}F{^{1}H} NMR (376.50 MHz, CD_2Cl_2, 298 K): δ -80.9 (dd, $^{4}J_{FP}$ = 10.3, $^{4}J_{FF}$ = 9.7, C*F*), -107.8 (d, $^{4}J_{FF}$ = 9.7, C*F*), -152.6 (br, B*F*$_4$). ^{31}P{^{1}H} NMR (202.54 MHz, CD_2Cl_2, 298 K): δ 89.0 (d, $^{4}J_{FP}$ = 10.3, *P*). (+)-HR-ESI-MS (electrospray, m/z): calcd for $C_{23}H_{26}NAuF_2P$ [M]$^{+}$, 582.14311; found, 582.14280.

[(P^N^C^{H})Au]$_2$ (5^{H})

{Au[κ^{2}-P,C-(8-(di-tert-buthylphosphino)-2-phenylquinoline)]}$_2$. Compound **4^{H}** (16 mg, 0.0253 mmol) and KH (25 mg, 0.632 mmol) were placed in a Schlenk, and toluene (2 mL) was added. The resulting mixture was stirred at room temperature for 24 h. The mixture was filtered through celite and concentrated under reduced pressure. The resulting crude was treated with pentane (1 mL) at 0 ºC to afford a brown solid, which was washed with pentane (2 x 1 mL), and subsequently dried under reduced pressure. Light brown solid. Yield: 9 mg (65%).

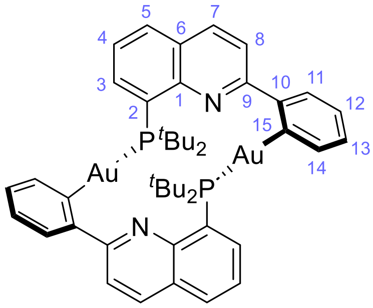

^{1}H NMR (400.13 MHz, CD_2Cl_2, 298 K): δ 8.55 (dd, $^{3}J_{HP}$ = 17.7, $^{3}J_{HH}$ = 6.9, 2H, H^{3}), 8.06 (d, $^{3}J_{HH}$ = 8.4, 2H, H^{7}), 7.97 (d, $^{3}J_{HH}$ = 8.6, 2H, H^{8}), 7.70-7.64 (m, 4H, H^{5} and H^{11}), 7.51 (d, $^{3}J_{HH}$ = 7.6, 2H, H^{14}), 7.35-7.30 (m, 2H, H^{12}), 7.12 (ddd, $^{3}J_{HH}$ = 7.5, $^{3}J_{HH}$ = 7.5, $^{4}J_{HP}$ = 1.1, 2H, H^{13}), 6.66 (ddd, $^{3}J_{HH}$ = 7.9, $^{3}J_{HH}$ = 7.5, 2H, H^{4}), 1.19 (d, $^{3}J_{HP}$ = 15.4, 36H, H^{17}). ^{13}C{^{1}H} NMR (100.62 MHz, CD_2Cl_2, 298 K): δ 175.2 (d, $^{2}J_{CP}$ = 102.5, C^{15}), 166.6 (d, $^{4}J_{CP}$ = 2.0, C^{9}), 150.1 (d, $^{3}J_{CP}$ = 1.0, C^{10}), 148.8 (s, C^{1}), 145.3 (d, $^{2}J_{CP}$ = 28.5, C^{3}), 139.5 (s, C^{11}), 135.3 (s, C^{7}), 132.0 (d, $^{4}J_{CP}$ = 1.8, C^{5}), 131.9 (d, $^{1}J_{CP}$ = 28.1, C^{2}), 128.0 (d, $^{3}J_{CP}$ = 5.0, C^{14}), 127.6 (d, $^{3}J_{CP}$ = 4.6, C^{6}), 127.0 (d, $^{5}J_{CP}$ = 4.8, C^{12}), 125.9 (s, C^{8}), 124.5 (d, $^{3}J_{CP}$ = 5.0, C^{4}), 124.4 (s, C^{13}), 37.2 (d, $^{1}J_{CP}$ = 19.5, C^{16}), 31.1 (d, $^{2}J_{CP}$ = 7.4, C^{17}). ^{31}P{^{1}H} NMR (161.99 MHz, CD_2Cl_2, 298 K): δ 95.9 (s, *P*). (+)-HR-ESI-MS (electrospray, m/z): calcd for $C_{46}H_{55}N_2Au_2P_2$ [M+H]$^{+}$, 1091.31696; found, 1091.31696.

(P^N^C^{H})Au-(CMe)=(CMe$_2$) (6^{H})

[κ^{3}-P,N,C-(8-(di-tert-Butylphosphino)-2-phenylquinoline)]Au(CMe)=(CMe$_2$). Compound **4^{H}** (16 mg, 0.0253 mmol) and 1,1-dimethylallene (15 µL, 0.155 mmol) were placed in a Schlenk, and dichloromethane (1 mL) was added. The resulting mixture was stirred at room temperature for 24 h. The solution was concentrated under reduced pressure and the resulting crude was treated with cold diethyl ether to afford the

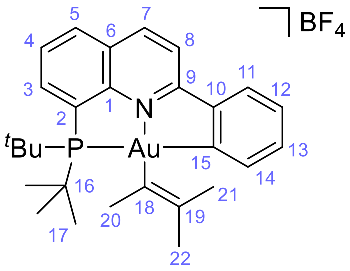

corresponding complex **6^H^** as a solid, which was washed with cold diethyl ether and cold pentane, and subsequently dried under vacuum pressure. Yield: 14 mg (64%). ^{1}H NMR (400.13 MHz, CD_2Cl_2, 298 K): δ 8.86 (dd, $^{3}J_{HH}$ = 8.8, $^{5}J_{HP}$ = 1.3, 1H, H^{7}), 8.41 (ddd, $^{3}J_{HH}$ = 7.5, $^{3}J_{HP}$ = 7.5, $^{4}J_{HH}$ = 1.1, 1H, H^{3}), 8.36-8.33 (m, 2H, H^{5} and H^{8}), 8.08-8.05 (m, 1H, H^{11}), 8.00 (ddd, $^{3}J_{HH}$ = 7.9, $^{3}J_{HH}$ = 7.6, $^{4}J_{HP}$ = 1.5, 1H, H^{4}), 7.60-7.56 (m, 2H, H^{13} and H^{14}) 7.55-7.50 (m, 1H, H^{12}), 2.41 (s, 3H, H^{20}), 1.88 (s, 3H, H^{22}), 1.82 (s, 3H, H^{21}), 1.52 (d, $^{3}J_{HP}$ = 16.2, 18H, H^{17}). ^{13}C{^{1}H} NMR (100.62 MHz, CD_2Cl_2, 298 K): δ 165.6 (d, $^{3}J_{CP}$ = 6.3, C^{9}), 162.3 (d, $^{2}J_{CP}$ = 116.0, C^{15}), 150.2 (d, $^{2}J_{CP}$ = 10.8, C^{1}), 148.1 (s, C^{10}), 143.7 (s, C^{7}), 141.5 (d, $^{2}J_{CP}$ = 1.7, C^{3}), 138.1 (d, $^{2}J_{CP}$ = 7.8, C^{18}), 134.2 (d, $^{4}J_{CP}$ = 7.4, C^{13}), 134.0 (d, $^{4}J_{CP}$ = 2.0, C^{5}), 133.6 (d, $^{3}J_{CP}$ = 1.0, C^{14}), 130.2 (d, $^{3}J_{CP}$ = 6.1, C^{6}), 129.6 (d, $^{3}J_{CP}$ = 6.2, C^{4}), 129.6 (d, $^{1}J_{CP}$ = 34.8, C^{2}), 129.2 (s, C^{12}), 128.5 (d, $^{4}J_{CP}$ = 5.5, C^{11}), 126.9 (d, $^{3}J_{CP}$ = 2.7, C^{19}), 119.8 (s, C^{8}), 38.8 (d, $^{1}J_{CP}$ = 14.4, C^{16a}), 38.6 (d, $^{1}J_{CP}$ = 15.0, C^{16b}), 30.8 (d, $^{4}J_{CP}$ = 1.1, C^{21}). 29.9 (d, $^{2}J_{CP}$ = 4.9, C^{17a}), 29.5 (d, $^{2}J_{CP}$ = 4.1, C^{17b}), 24.3 (d, $^{3}J_{CP}$ = 1.0, C^{20}), 24,1 (d, $^{4}J_{CP}$ = 1.0, C^{22}). ^{31}P{^{1}H} NMR (161.99 MHz, CD_2Cl_2, 298 K): δ 72.0 (s, *P*). Impurities at 1.24 ppm in the ^{1}H NMR and at 83.3 and 24.7 ppm in the ^{13}C NMR spectra correspond to $O(Bpin)_2$. (+)-HR-ESI-MS (electrospray, m/z): calcd for $C_{28}H_{36}NAuP$ $[M]^{+}$, 614.22454; found, 614.22396.

5. Reactivity studies

5.1 Reactions with Brønsted acids

Three independent experiments were performed in Young NMR tubes, where a solution of compound **4^{H}** (3 mg, 0.0047 mmol) in dicholormethane-d_2 (0.5 mL) was treated with 5 equiv of weak acids (PhOH or HCO_2H) or 1 equiv of strong acid ($HBF_4 \cdot Et_2O$). The reaction progress was monitored by ^{1}H NMR spectroscopy at room temperature for 24 h. Similarly, compounds **4^{iPr}** and **4^{F}** were subjected to identical reaction conditions. The results of these experiments are summarized in Table S1.

Table S1. Reactions of gold(III)-hydrides **4^{H}**, **4^{iPr}** and **4^{F}** with Brønsted acids.

	**PhOH** (5 equiv)	**HCO_2H** (5 equiv)	**$HBF_4 \cdot Et_2O$** (1 equiv)
**4^{H}**	n.r.	n.r.	n.r.
**4^{iPr}**	n.r.	n.r.	n.r.
**4^{F}**	n.r.	n.r.	decomposition

Compounds **4^{H}** and **4^{iPr}** did not show any reaction towards Brønsted acids, whereas **4^{F}** did not react with weak acids but decomposed when treated with $HBF_4 \cdot Et_2O$. Note that only 1 equiv of $HBF_4 \cdot Et_2O$ was used in these experiments as with 5 equiv or above formation of the gold(III)-chloride derivatives was observed.

5.2 Reactivity vs KH

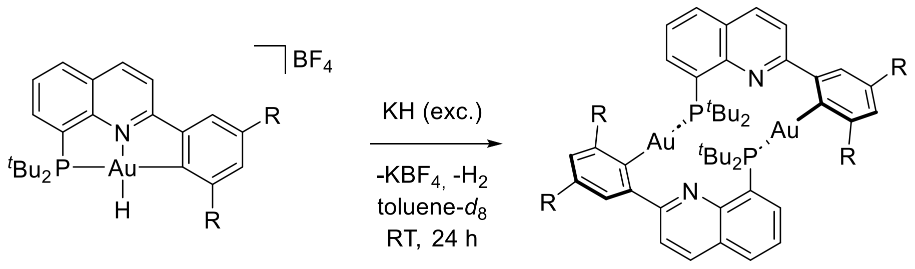

4^{H}, **4^{iPr}**, **4^{F}**

5^{H}, **5^{iPr}**, **5^{F}**

Scheme S3. Reaction of (P^N^C)gold(III)-hydrides with KH.

Compound **4^{H}** (3 mg, 0.0047 mmol) and KH (1 mg, 0.025 mmol) were placed in a Young NMR tube. Toluene-d_8 (0.5 mL) was added and the reaction progress was monitored by NMR spectroscopy (Figure S1). The initial gold(III)-hydride **4^{H}** was insoluble in toluene and compound **5^{H}** was formed over time. Generation of H_2 was detected by ^{1}H NMR spectroscopy.

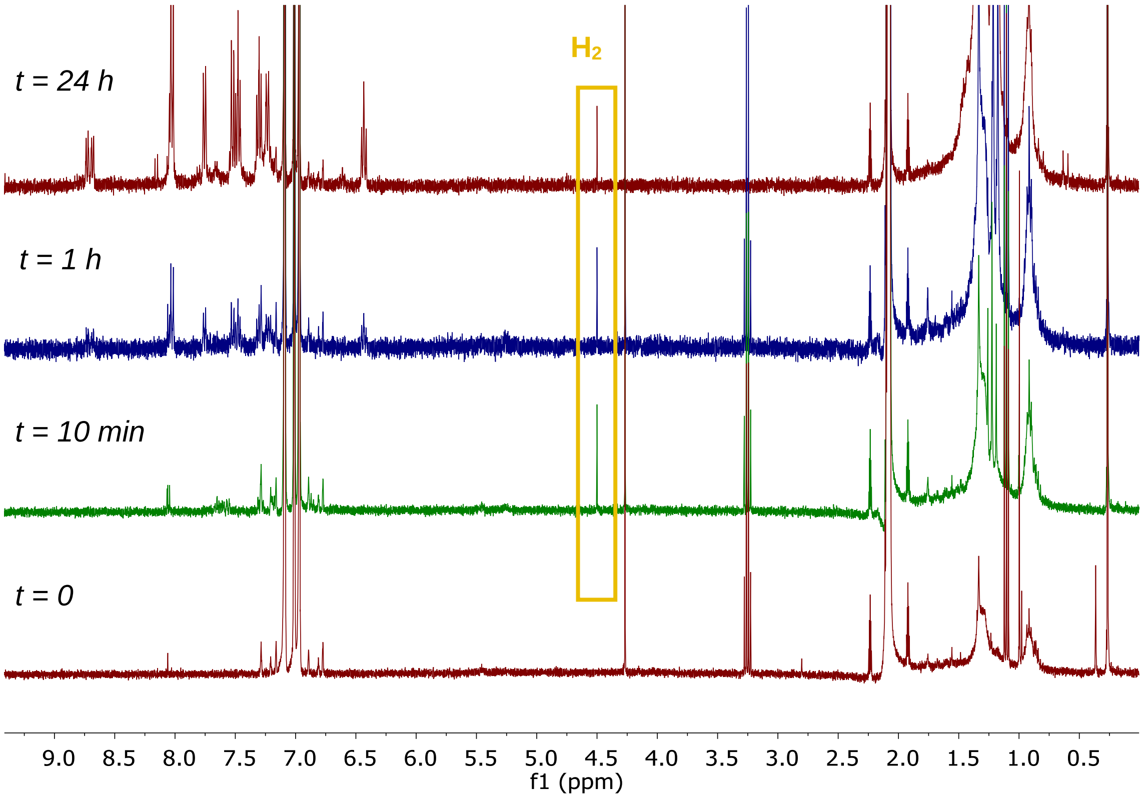

Figure S1. Stacked ^{1}H NMR spectra (400 MHz, in toluene-d_8) showing the formation of **5^H** and H_2 at time 0, 10 min, 1 h and 24 h. Impurities were found at 4.32 (dichloromethane), 3.25 and 1.10 (diethyl ether), 1.33 and 0.90 (H grease) and 0.26 ppm (silicon grease).

Similarly, upon reacting gold(III)-hydrides **4^iPr** and **4^F** with KH in toluene, the formation of dinuclear derivatives **5^iPr** and **5^F** was confirmed through the analysis of the reaction crude using $^{31}P\{^{1}H\}$ NMR spectroscopy (Figure S2) and HRMS-ESI spectrometry. The high solubility of compounds **5^iPr** and **5^F**, even in pentane at low temperatures, as well as the formation of unidentified subspecies in the reaction media, hindered the isolation of pure crystalline samples.

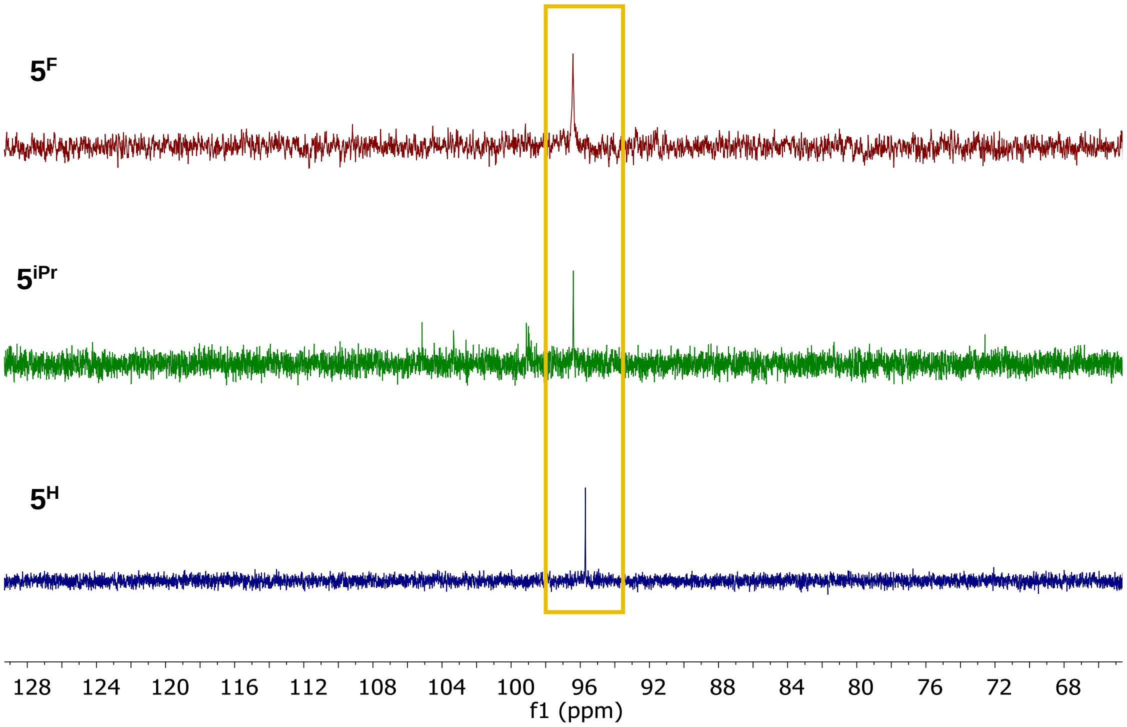

Figure S2. Stacked $^{31}P\{^1H\}$ NMR spectra (400 MHz, in toluene-d_8) showing the formation of $\mathbf{5^H}$ (95.9 ppm), $\mathbf{5^{iPr}}$ (96.4 ppm), and $\mathbf{5^F}$ (96.4 ppm).

6. NMR and IR spectra

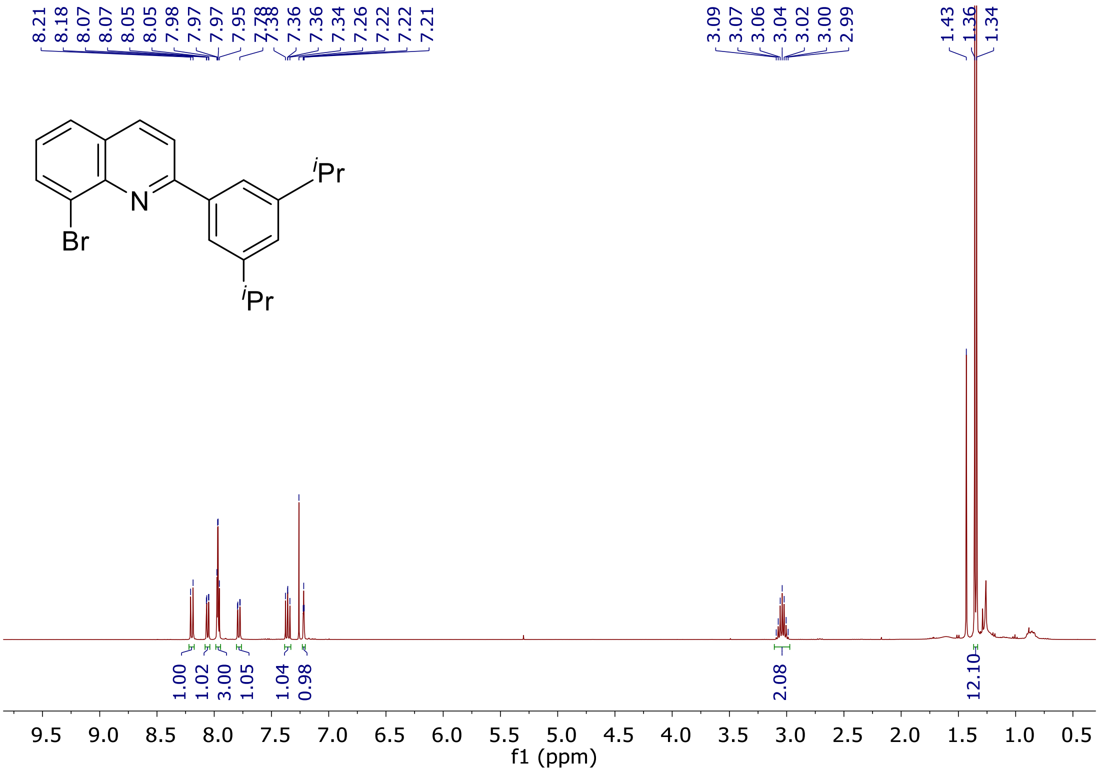

Figure S3. ^{1}H NMR spectrum (400.13 MHz, $CDCl_3$, 298 K) of compound **b** (residual cyclohexane 1.43 ppm).

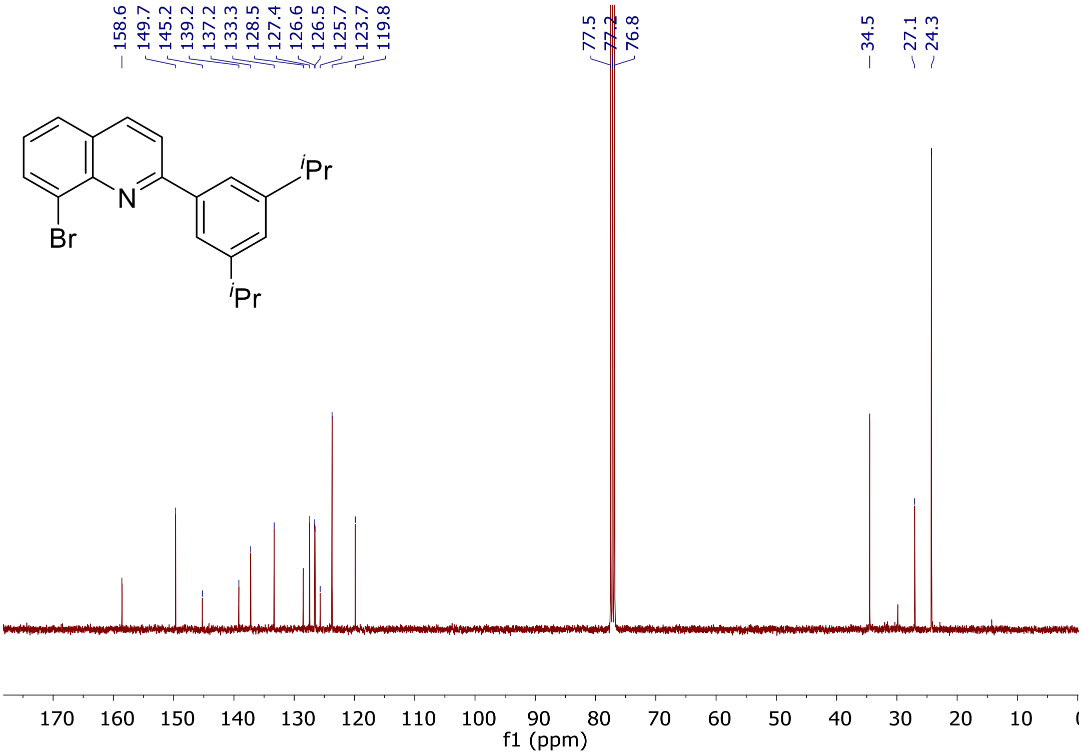

Figure S4. $^{13}C\{^{1}H\}$ NMR spectrum (100.65 MHz, $CDCl_3$, 298 K) of compound **b** (residual cyclohexane 27.1 ppm).

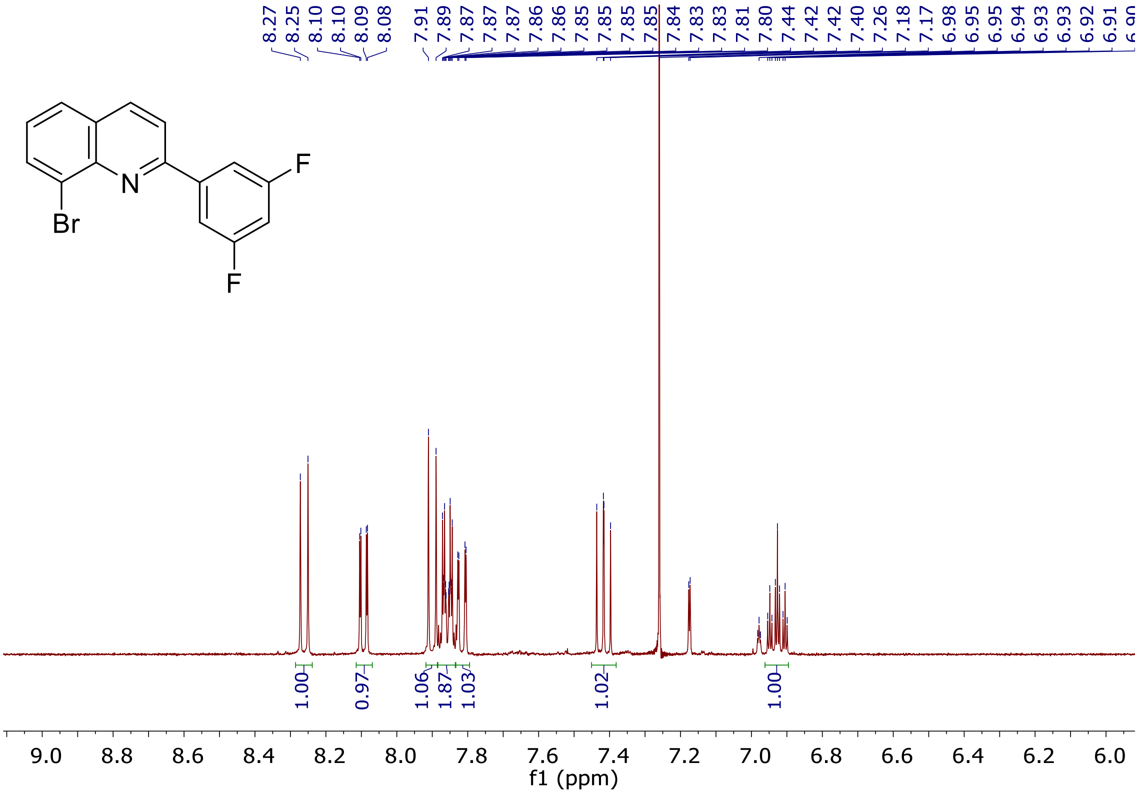

Figure S5. ^{1}H NMR spectrum (400.13 MHz, $CDCl_3$, 298 K) of compound **c**.

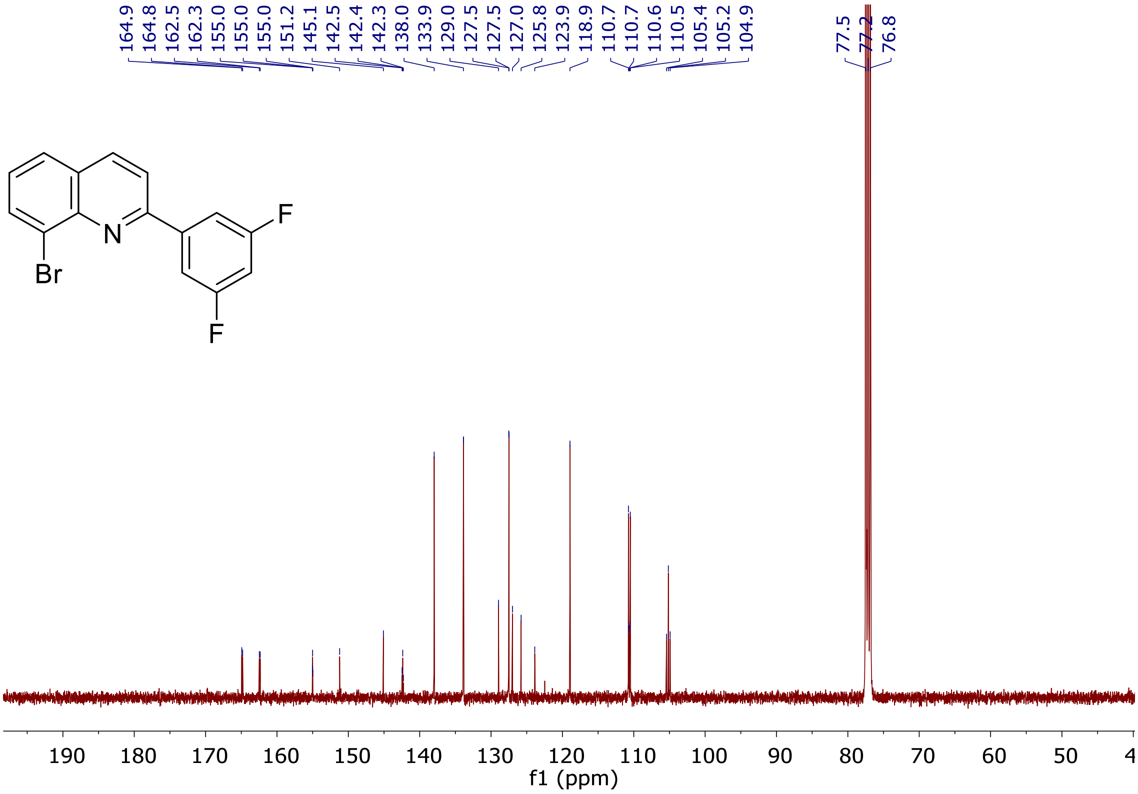

Figure S6. $^{13}C\{^{1}H\}$ NMR spectrum (100.65 MHz, $CDCl_3$, 298 K) of compound **c**.

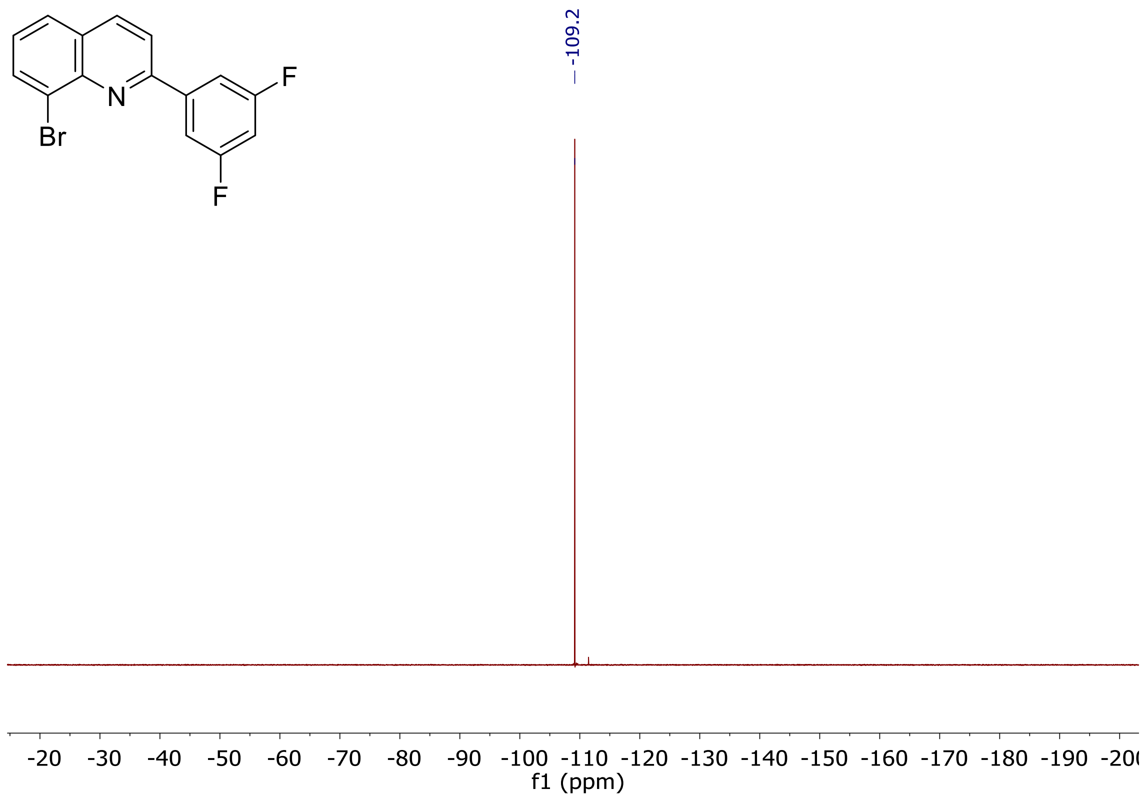

Figure S7. $^{19}F\{^{1}H\}$ NMR spectrum (376.50 MHz, $CDCl_3$, 298 K) of compound **c**.

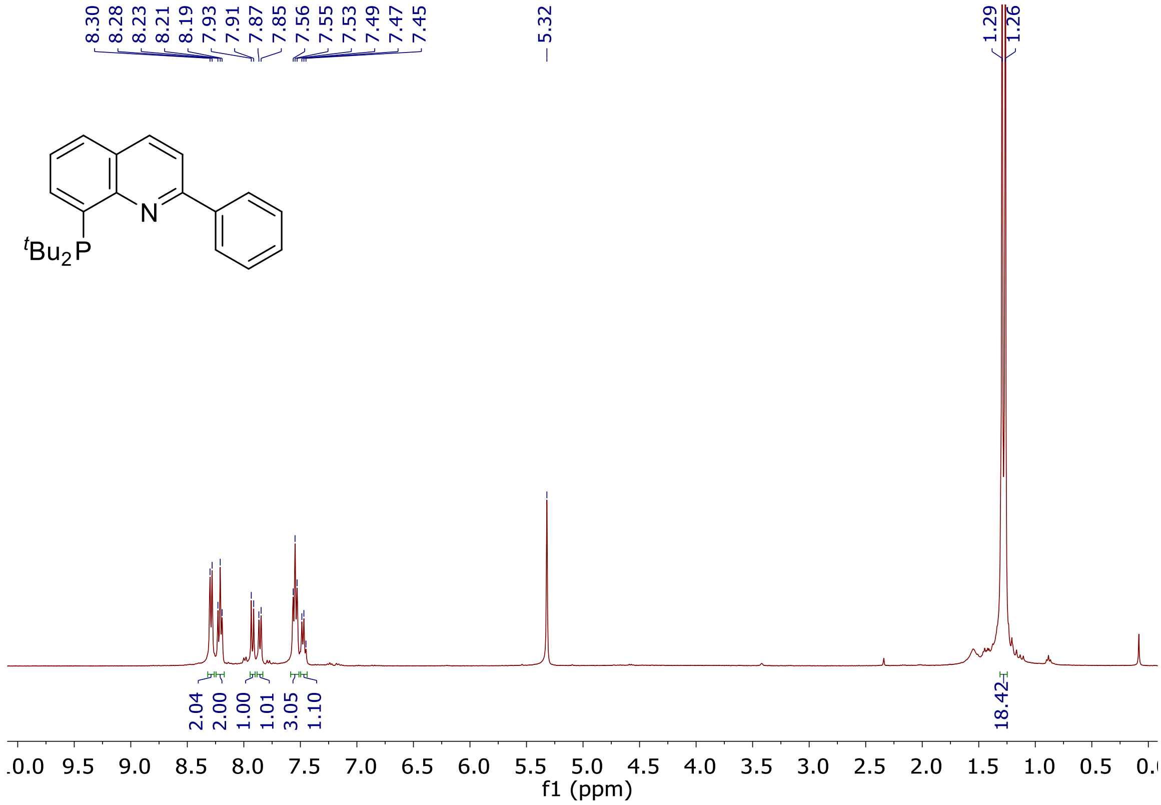

Figure S8. ^{1}H NMR spectrum (400.13 MHz, CD_2Cl_2, 298 K) of compound **L^H**.

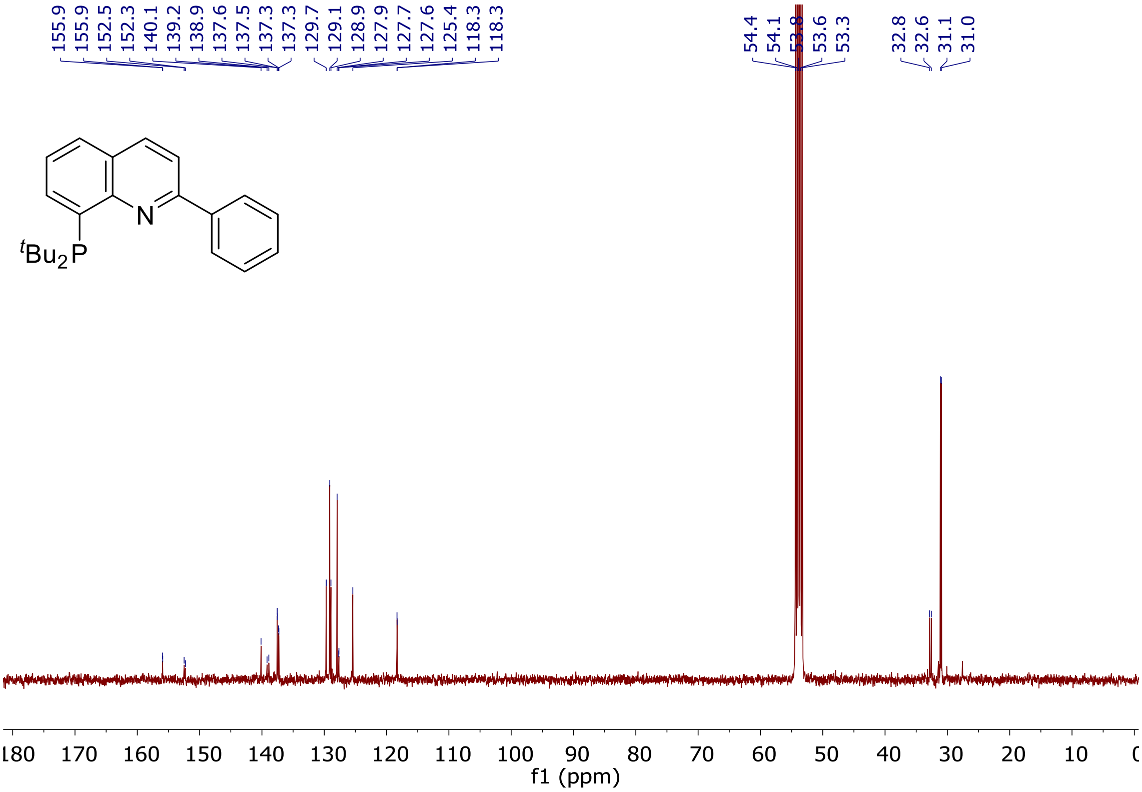

Figure S9. $^{13}C\{^1H\}$ NMR spectrum (100.65 MHz, CD_2Cl_2, 298 K) of compound $\mathbf{L^H}$.

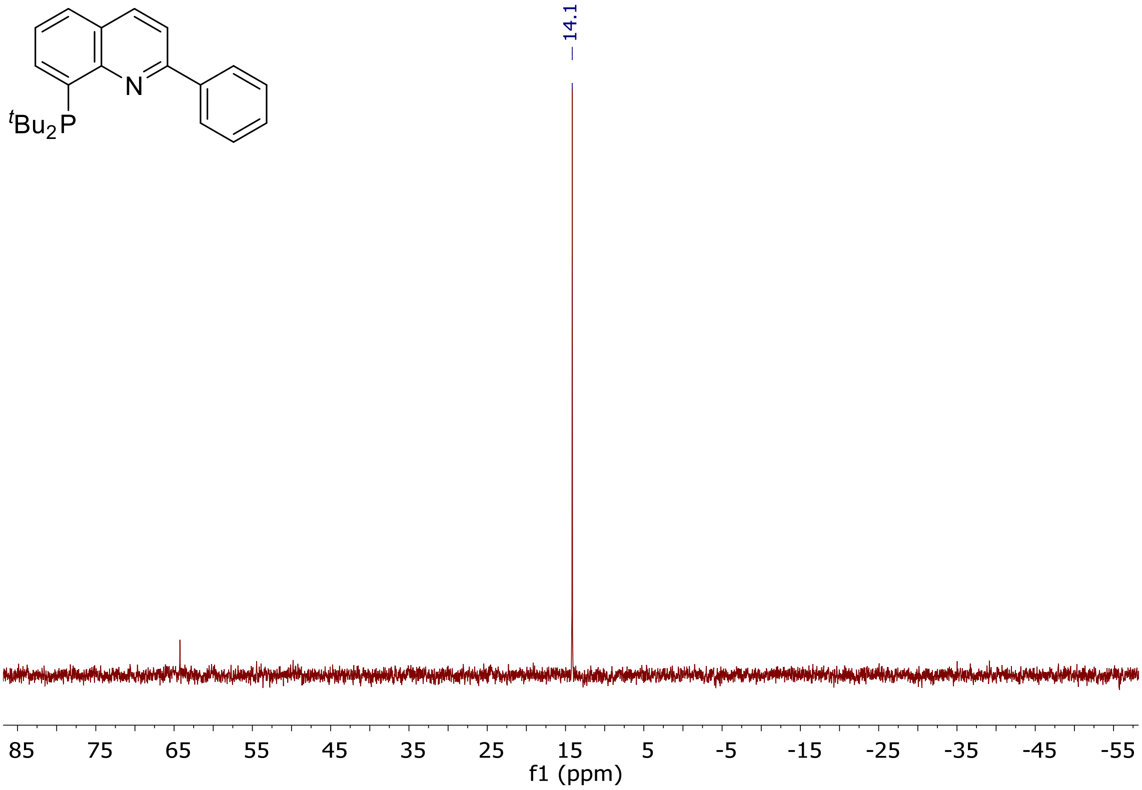

Figure S10. $^{31}P\{^1H\}$ NMR spectrum (161.99 MHz, CD_2Cl_2, 298 K) of compound $\mathbf{L^H}$.

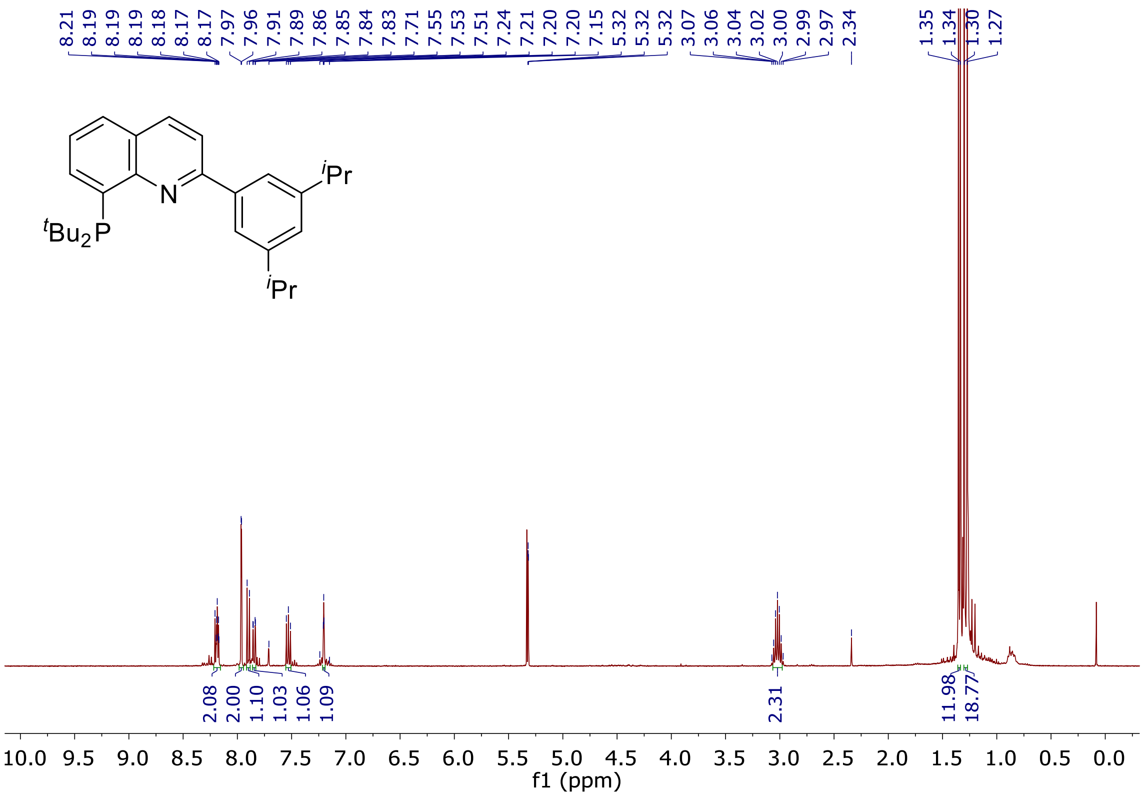

Figure S11. ^{1}H NMR spectrum (400.13 MHz, CD_2Cl_2, 298 K) of compound **L^{iPr}**.

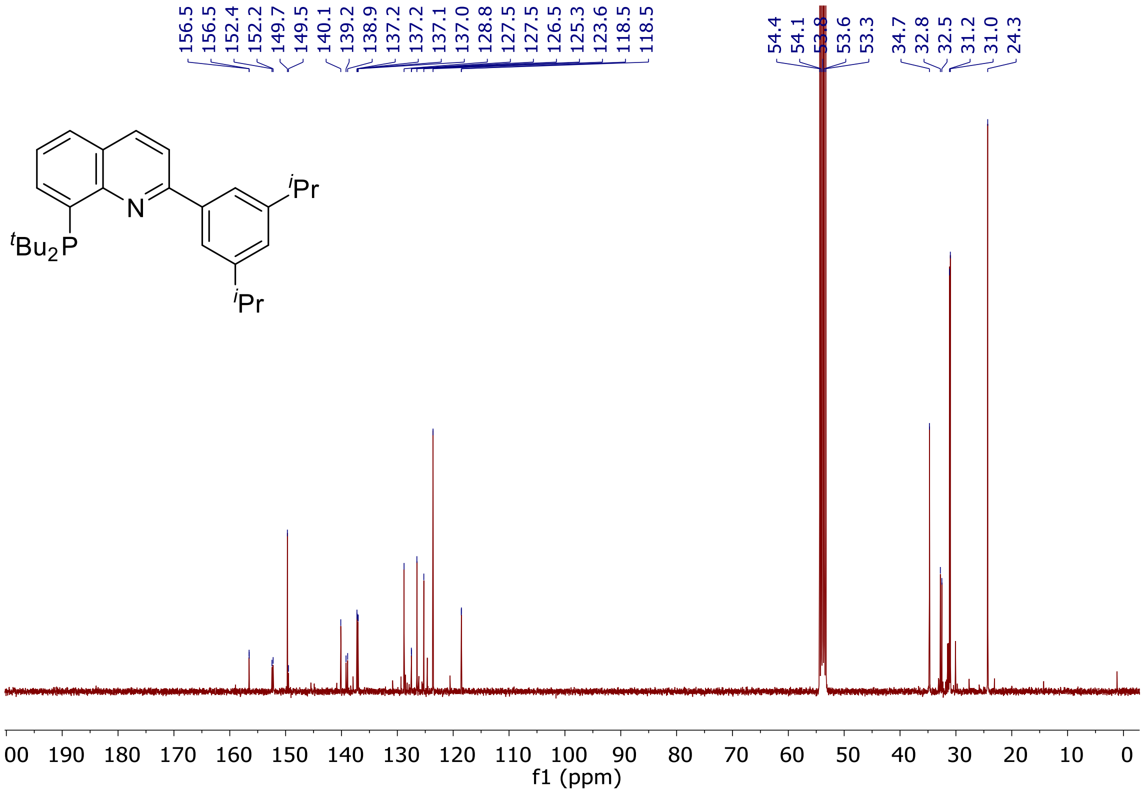

Figure S12. $^{13}C\{^{1}H\}$ NMR spectrum (100.65 MHz, CD_2Cl_2, 298 K) of compound **L^{iPr}**.

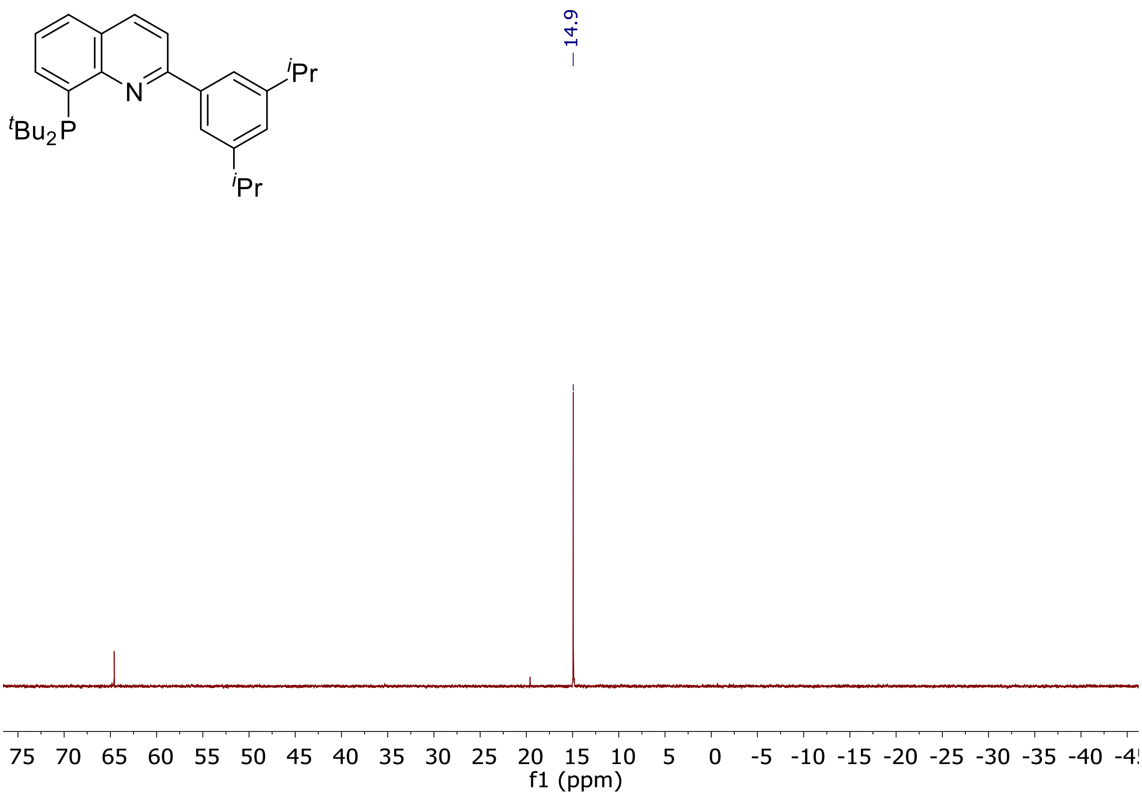

Figure S13. $^{31}P\{^{1}H\}$ NMR spectrum (161.99 MHz, CD_2Cl_2, 298 K) of compound $\mathbf{L^{iPr}}$.

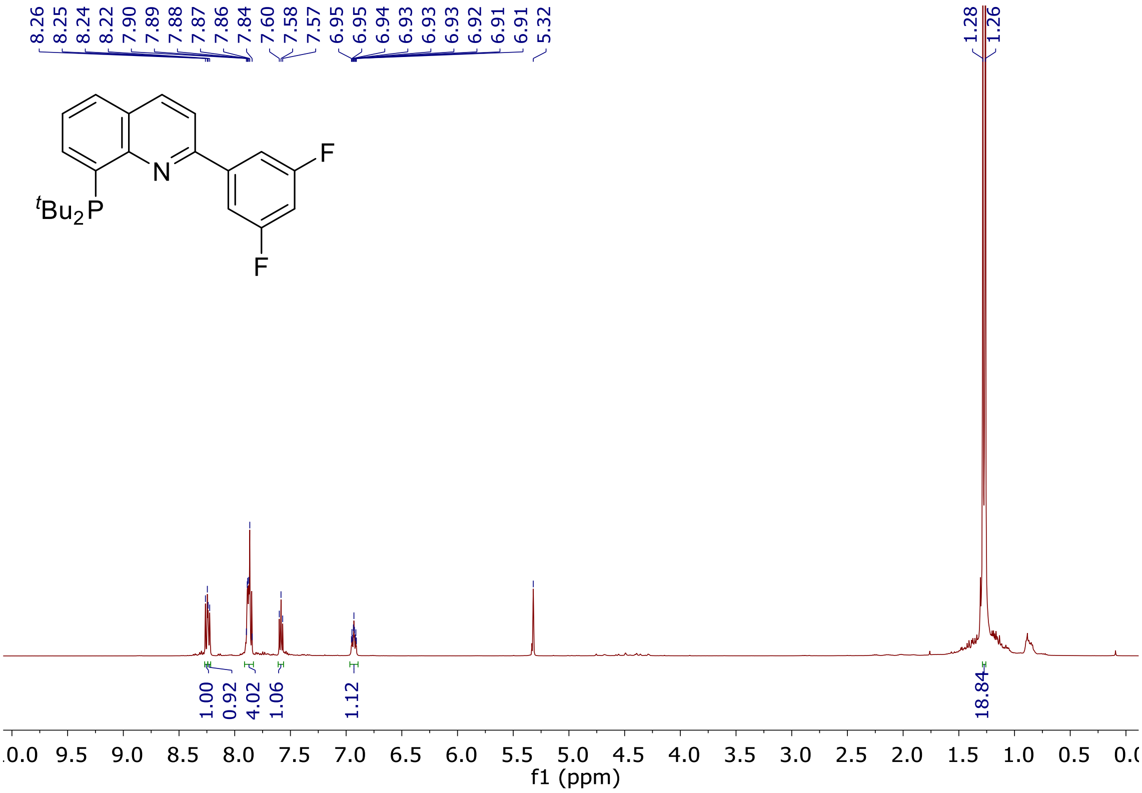

Figure S14. ^{1}H NMR spectrum (500.30 MHz, CD_2Cl_2, 298 K) of compound $\mathbf{L^{F}}$.

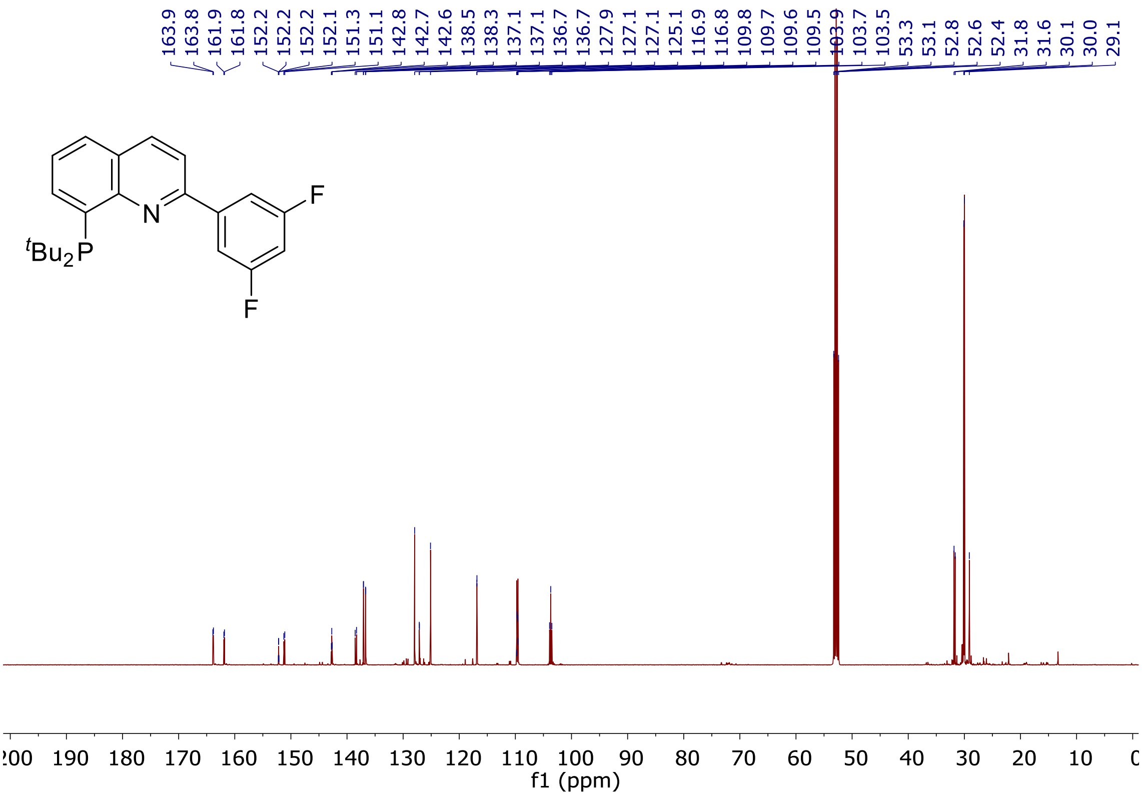

Figure S15. $^{13}C\{^{1}H\}$ NMR spectrum (125.81 MHz, CD_2Cl_2, 298 K) of compound **L^F^**.

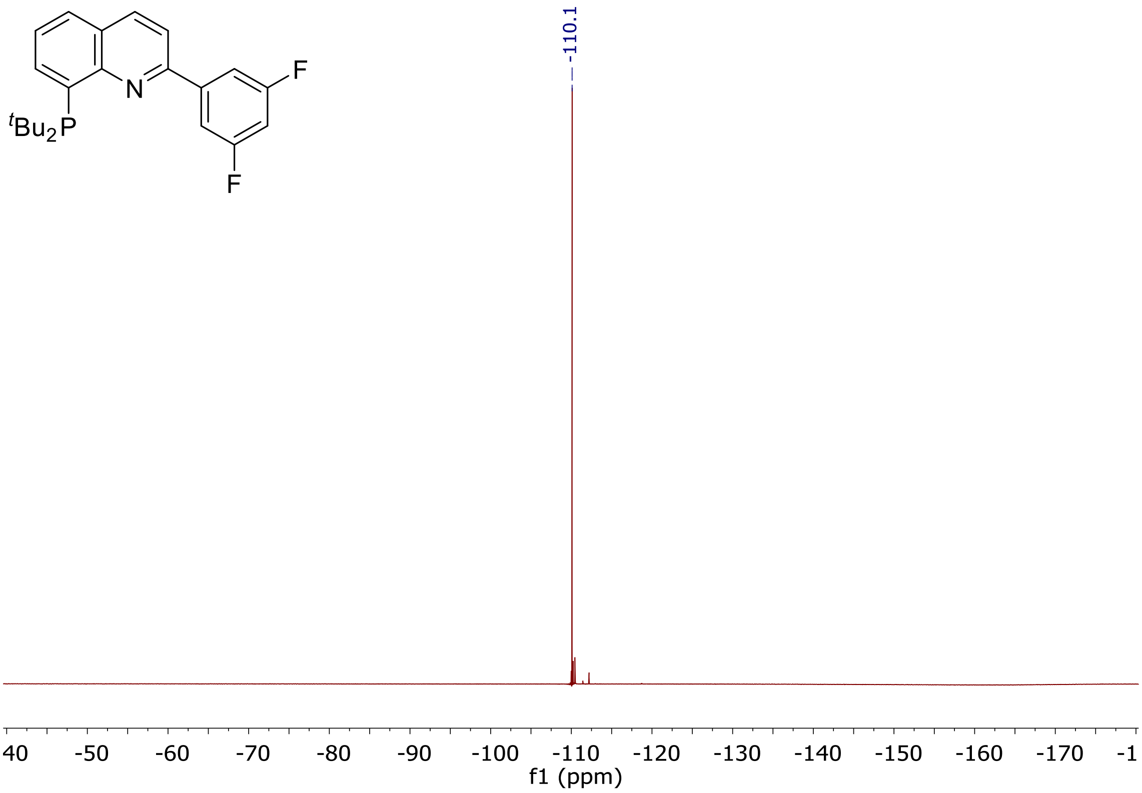

Figure S16. $^{19}F\{^{1}H\}$ NMR spectrum (376.50 MHz, CD_2Cl_2, 298 K) of compound **L^F^**.

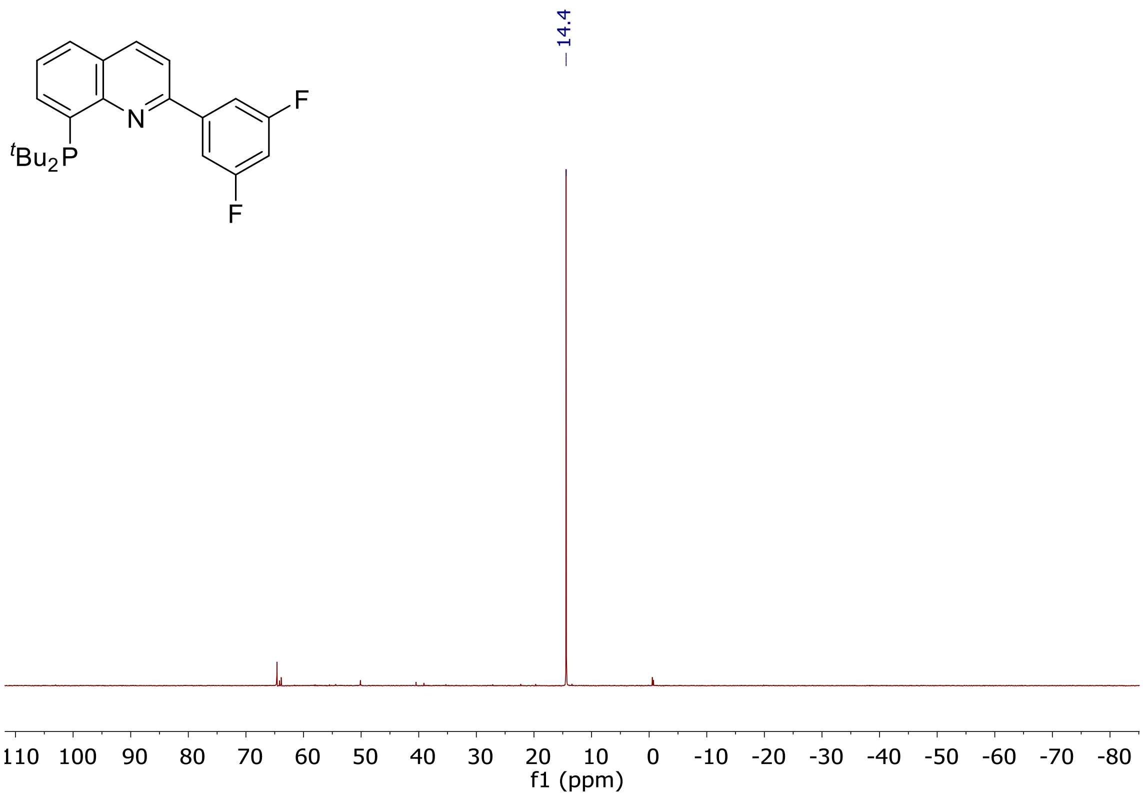

Figure S17. $^{31}P\{^{1}H\}$ NMR spectrum (161.99 MHz, CD_2Cl_2, 298 K) of compound **L^F**.

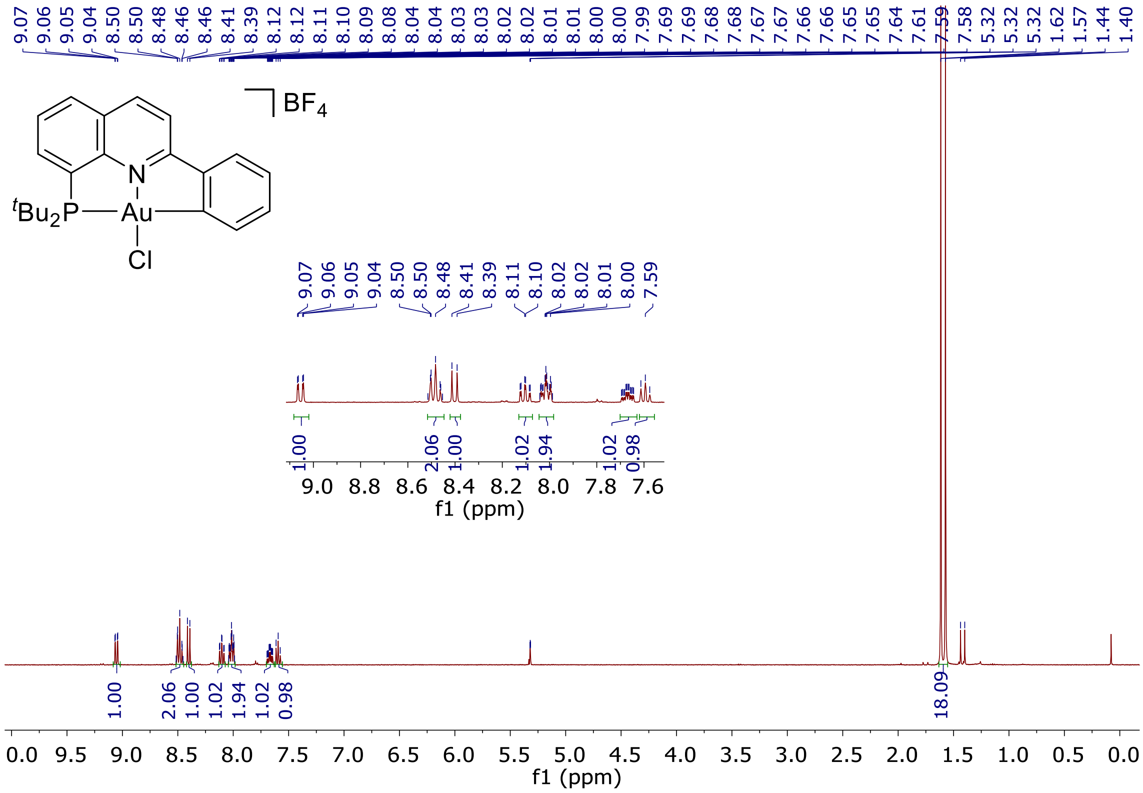

Figure S18. ^{1}H NMR spectrum (400.13 MHz, CD_2Cl_2, 298 K) of compound **1^H**.

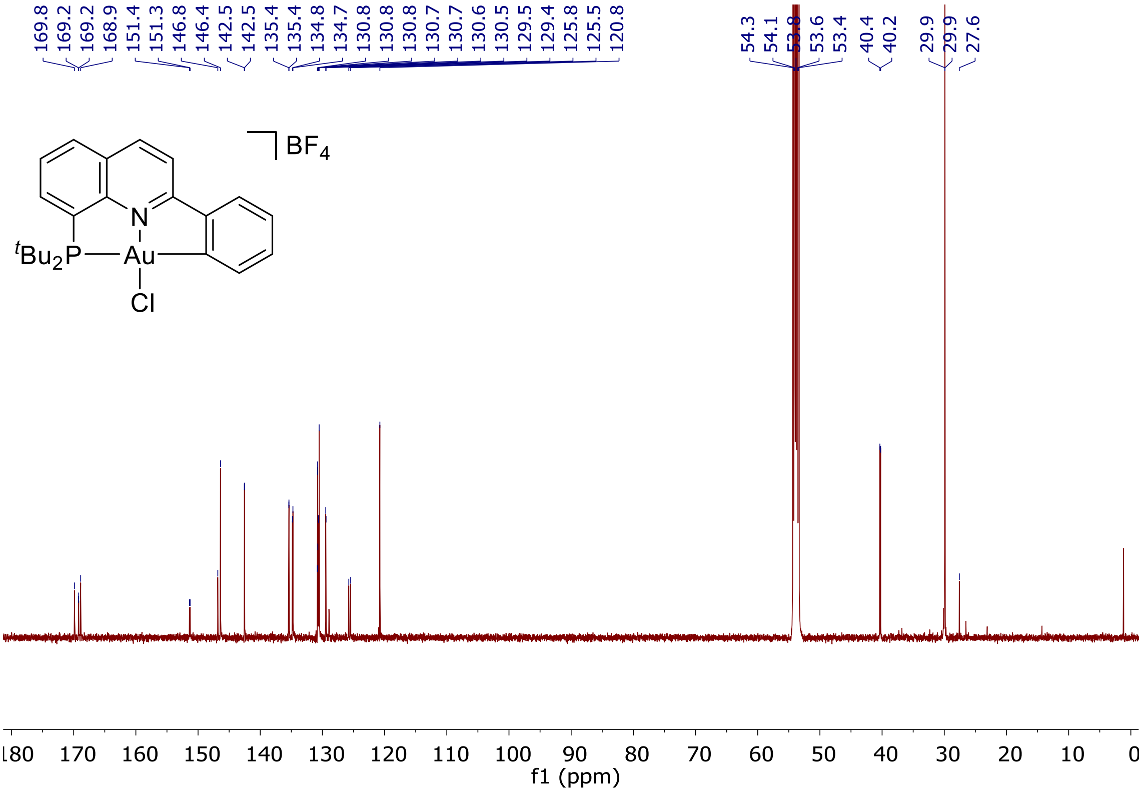

Figure S19. $^{13}C\{^1H\}$ NMR spectrum (125.81 MHz, CD_2Cl_2, 298 K) of compound $\mathbf{1^H}$.

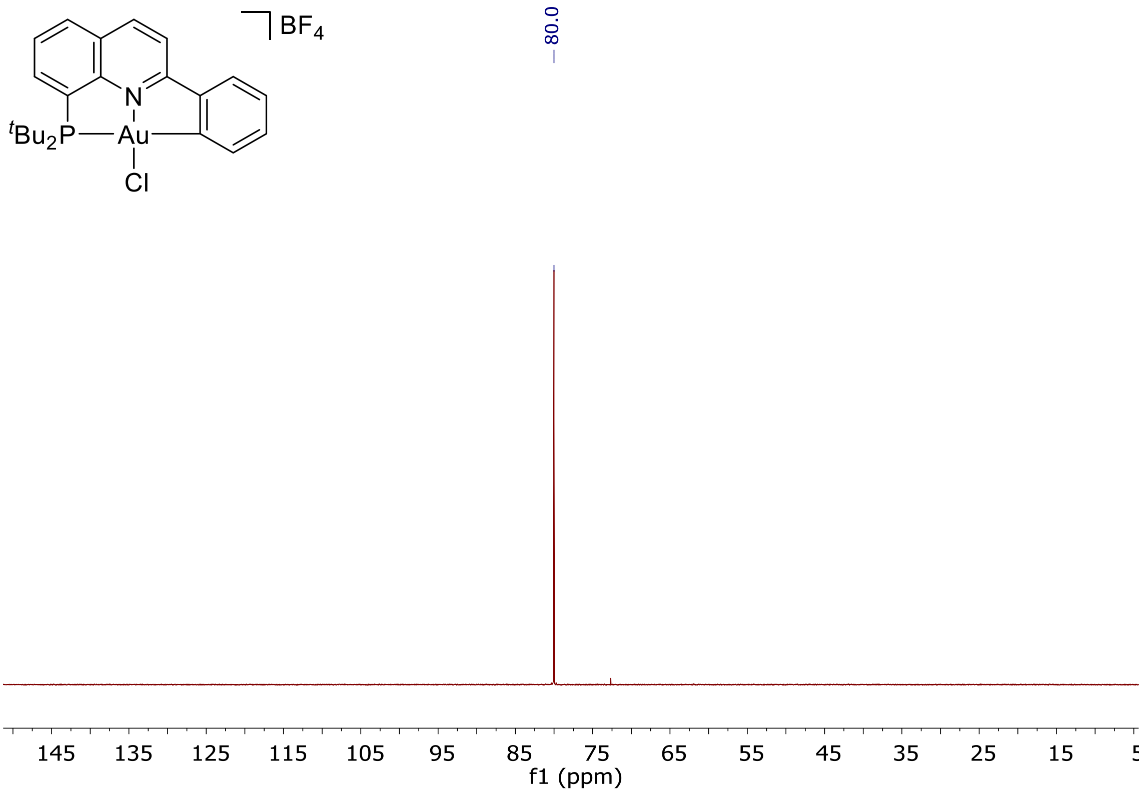

Figure S20. $^{31}P\{^1H\}$ NMR spectrum (202.52 MHz, CD_2Cl_2, 298 K) of compound $\mathbf{1^H}$.

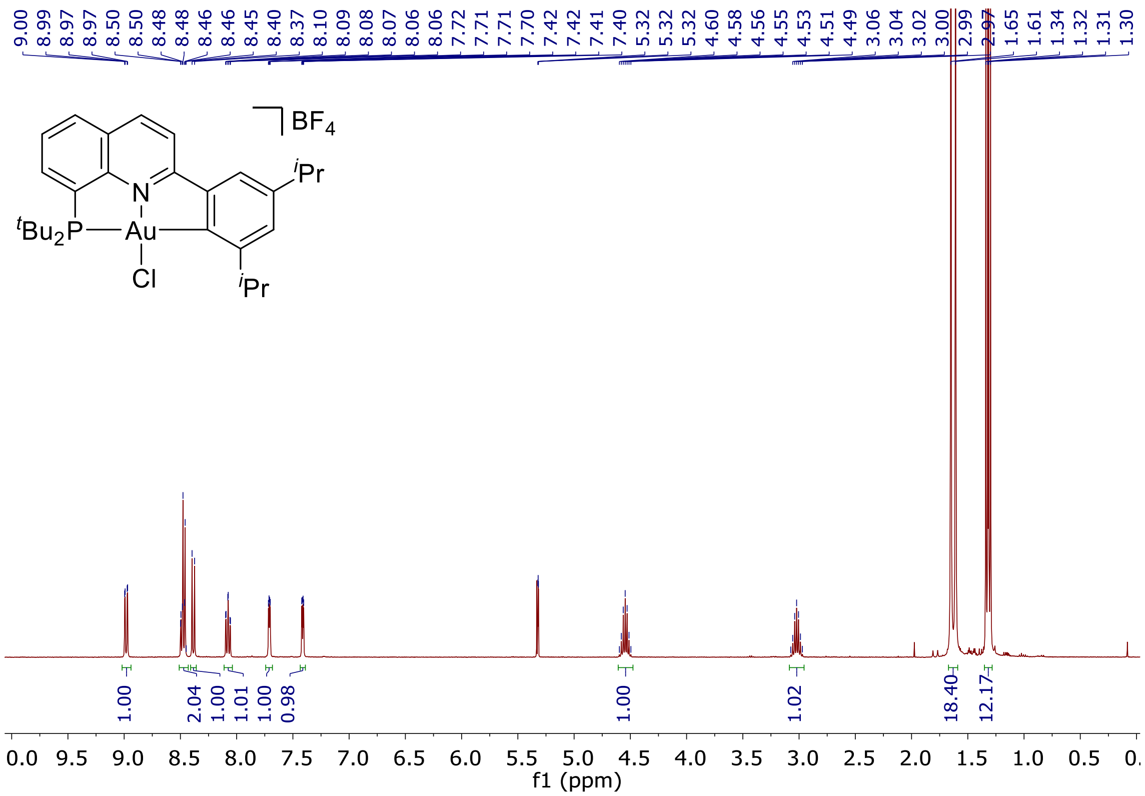

Figure S21. ^{1}H NMR spectrum (400.13 MHz, CD_2Cl_2, 298 K) of compound **1^{iPr}.**

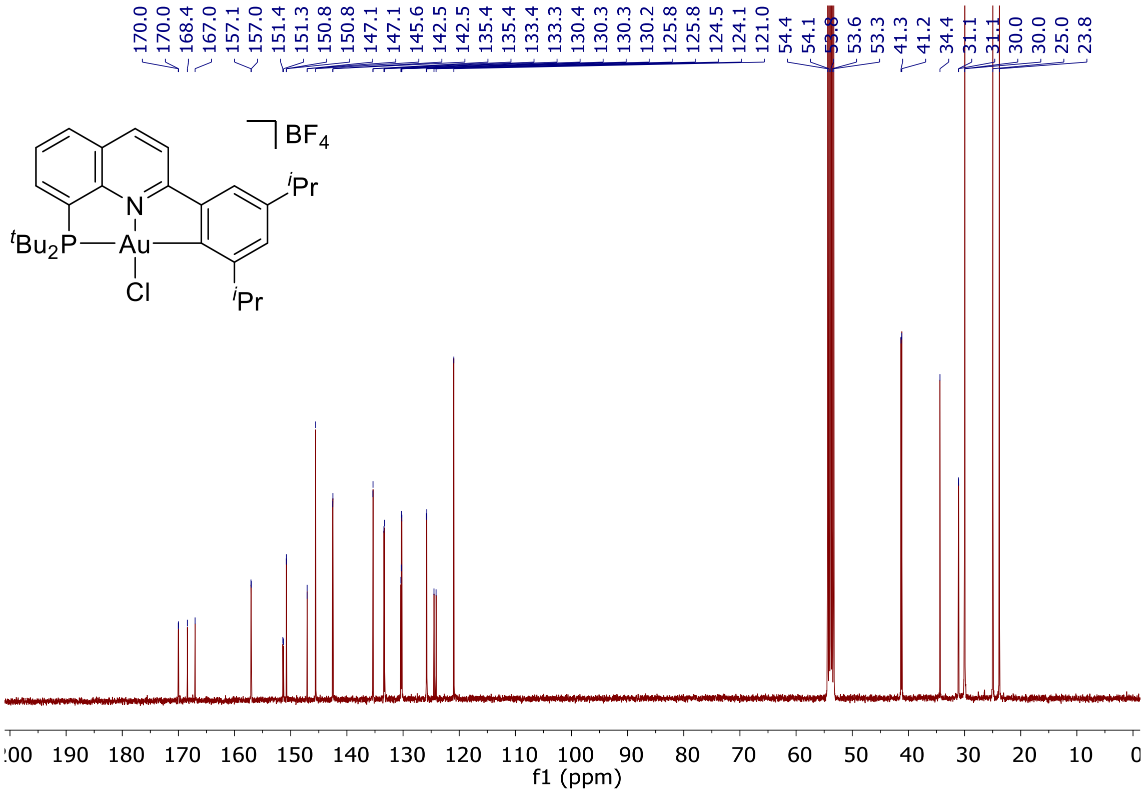

Figure S22. $^{13}C\{^{1}H\}$ NMR spectrum (100.62 MHz, CD_2Cl_2, 298 K) of compound **1^{iPr}**.

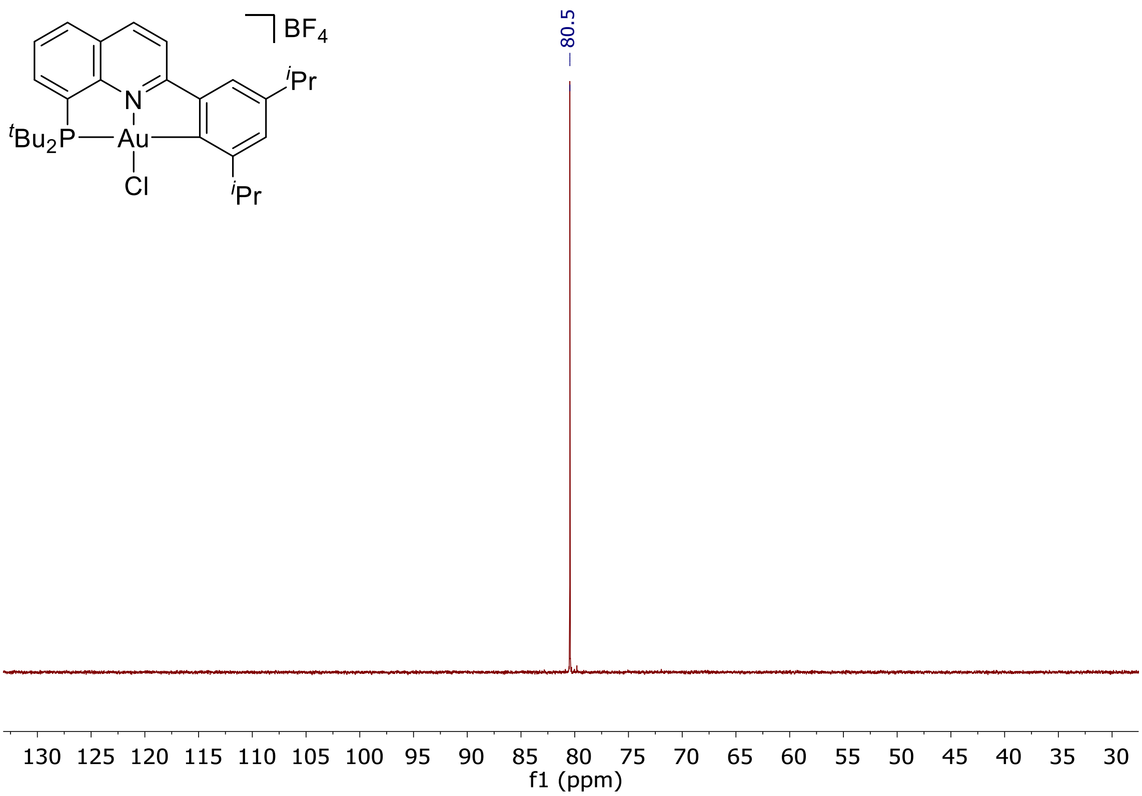

Figure S23. $^{31}P\{^{1}H\}$ NMR spectrum (161.99 MHz, CD_2Cl_2, 298 K) of compound $\mathbf{1^{iPr}}$.

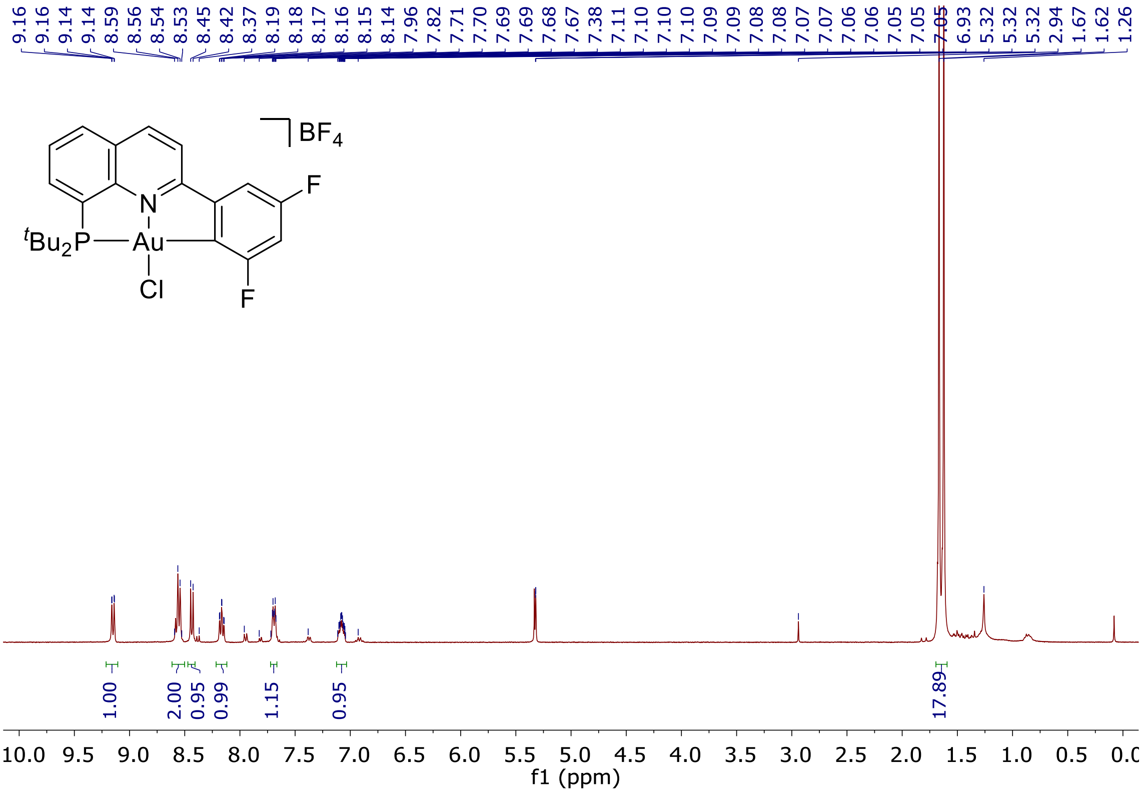

Figure S24. ^{1}H NMR spectrum (400.13 MHz, CD_2Cl_2, 298 K) of compound $\mathbf{1^{F}}$.

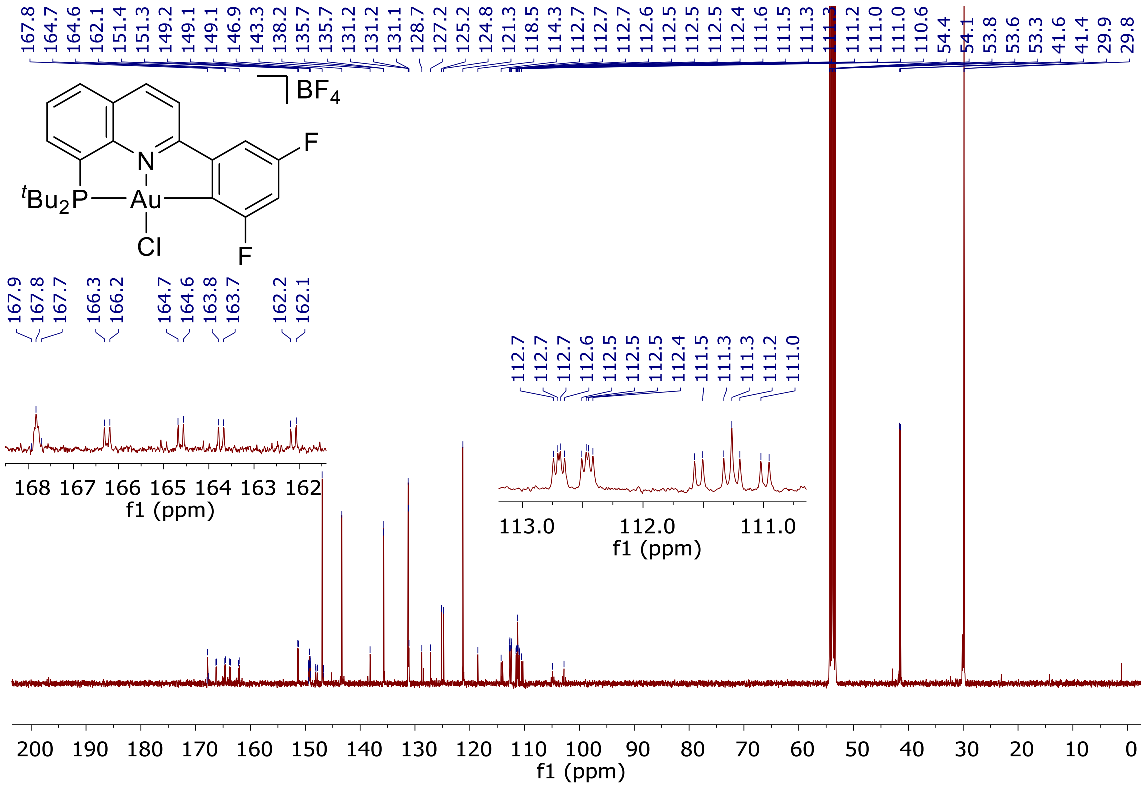

Figure S25. $^{13}C\{^{1}H\}$ NMR spectrum (100.62 MHz, CD_2Cl_2, 298 K) of compound $\mathbf{1^F}$.

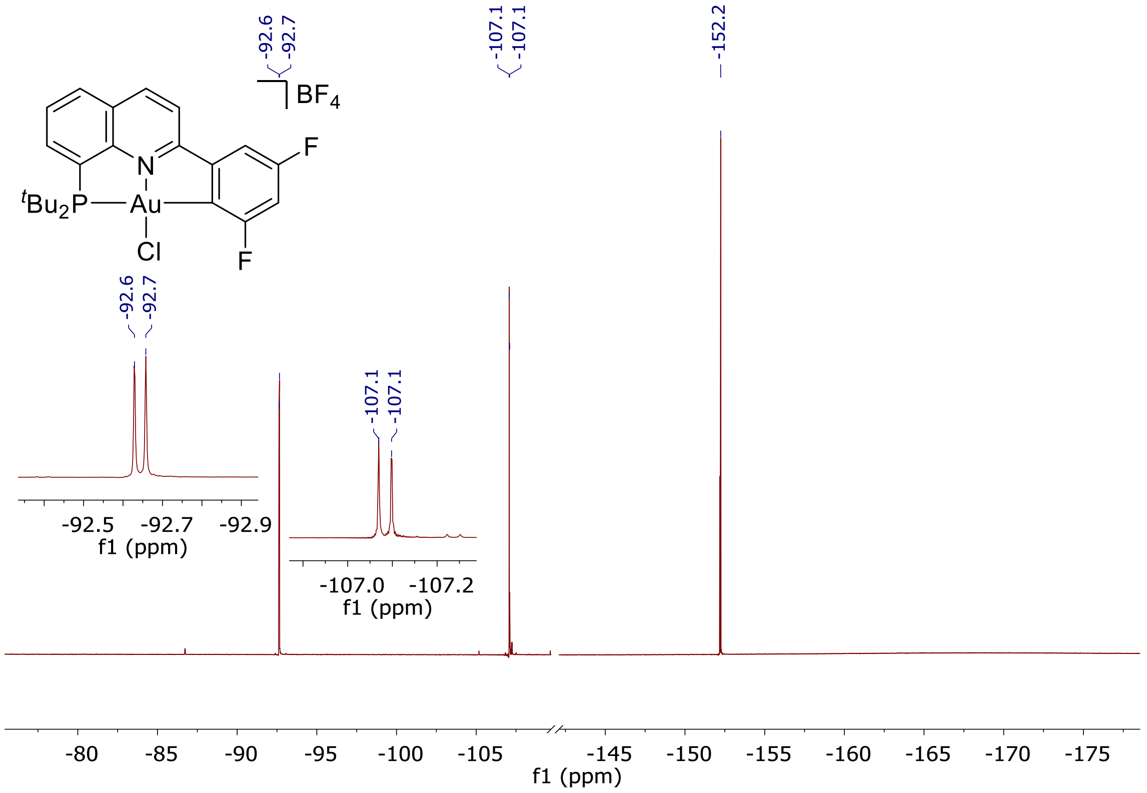

Figure S26. $^{19}F\{^{1}H\}$ NMR spectrum (376.50 MHz, CD_2Cl_2, 298 K) of compound $\mathbf{1^F}$.

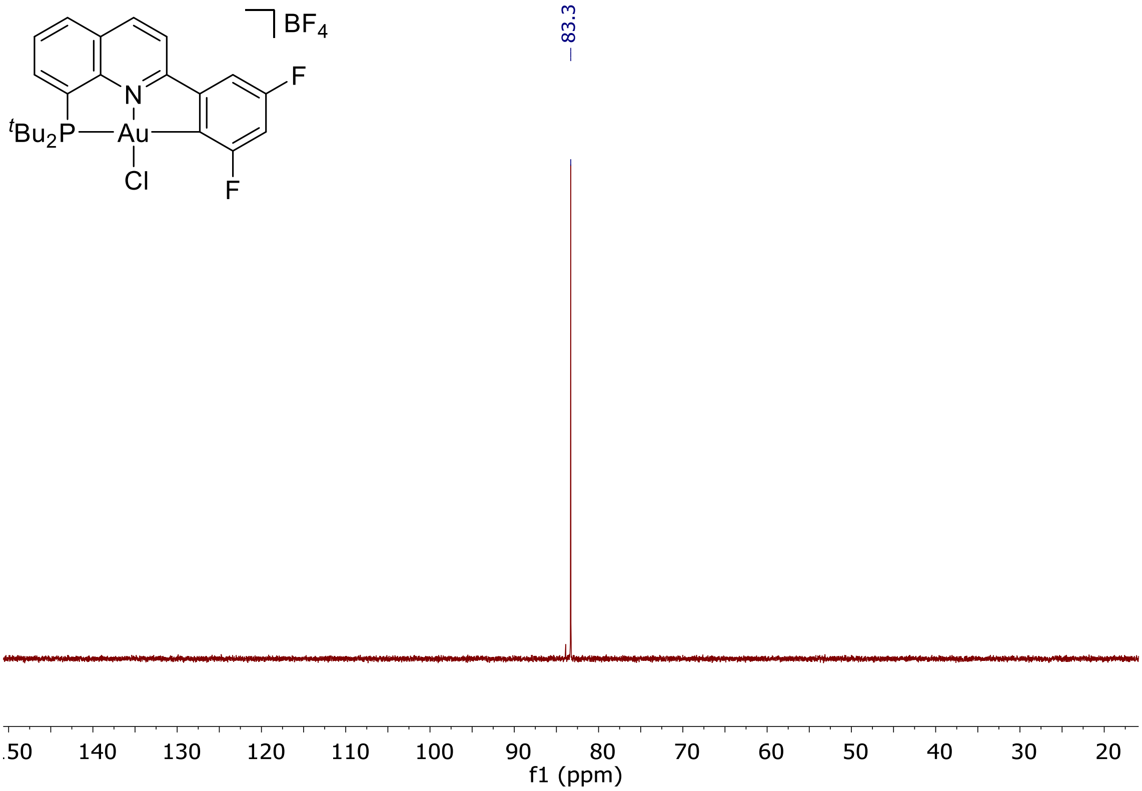

Figure S27. $^{31}P\{^{1}H\}$ NMR spectrum (161.99 MHz, CD_2Cl_2, 298 K) of compound $\mathbf{1^F}$.

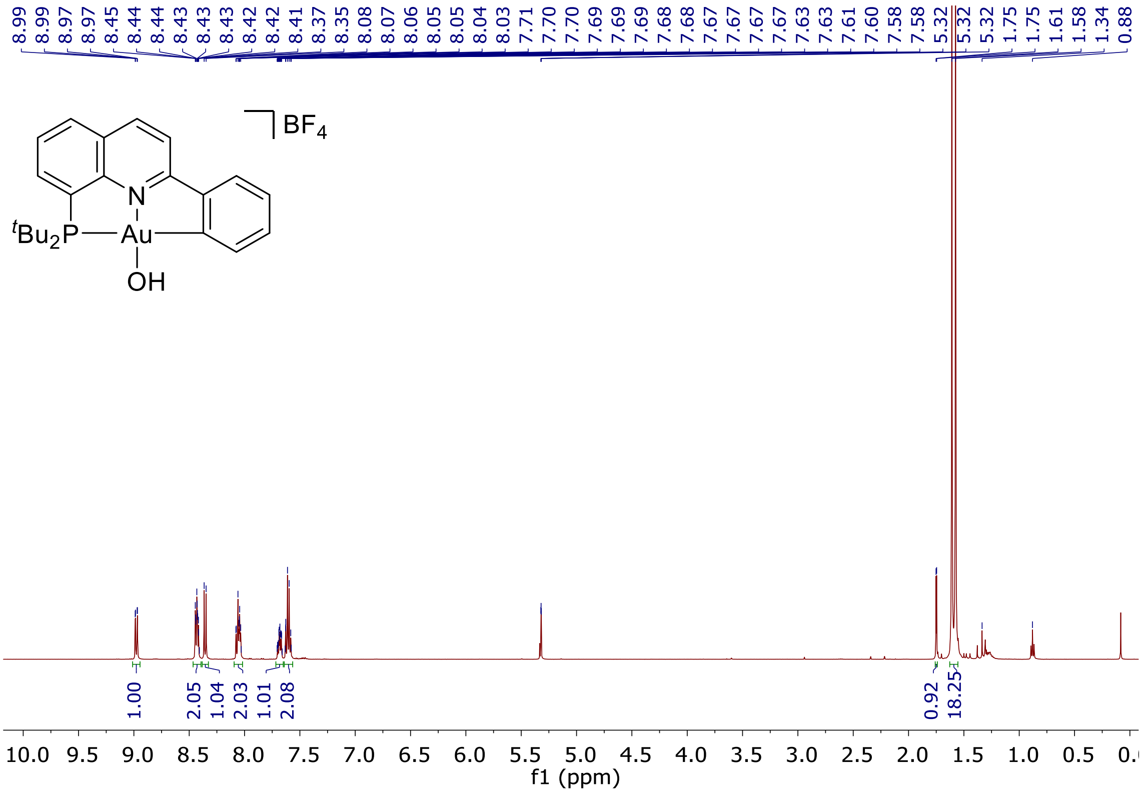

Figure S28. ^{1}H NMR spectrum (500.30 MHz, CD_2Cl_2, 298 K) of compound $\mathbf{2^H}$ (<2% residual pentane).

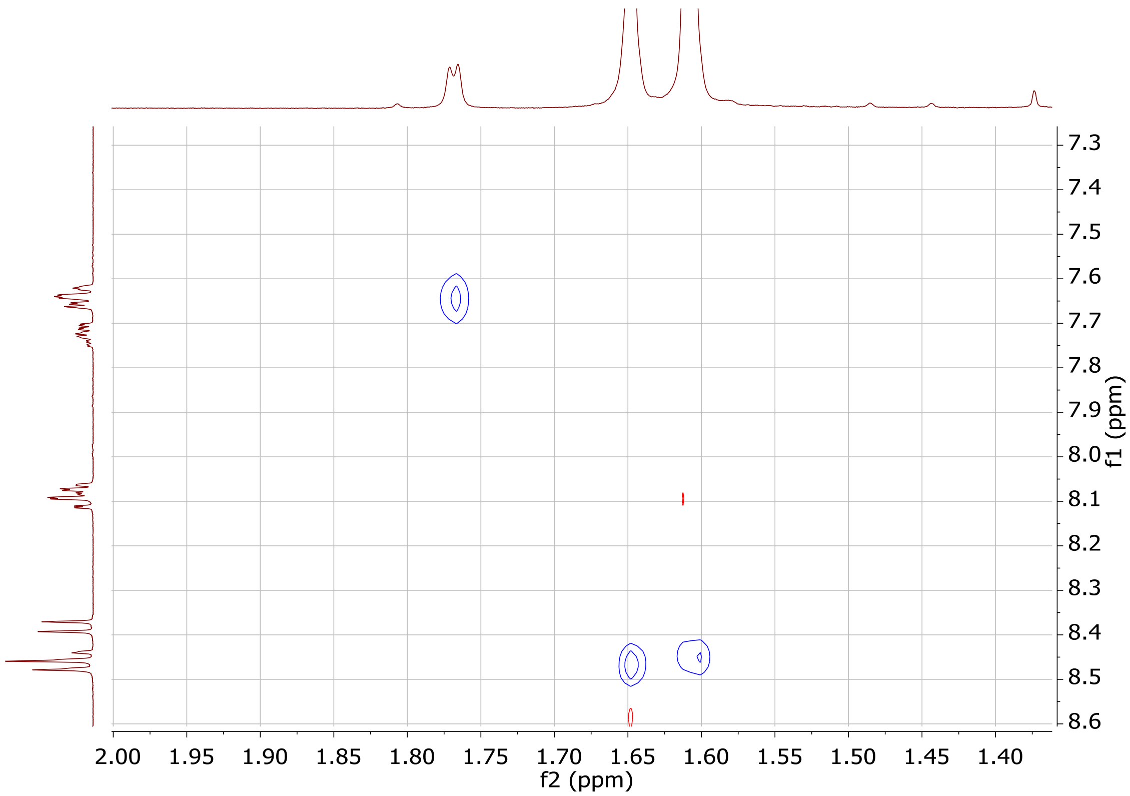

Figure S29. ^{1}H-^{1}H NOESY NMR spectrum (400.13 MHz, CD_2Cl_2, 298 K) of compound **2^{H}**. Expansion of O*H* cross peaks.

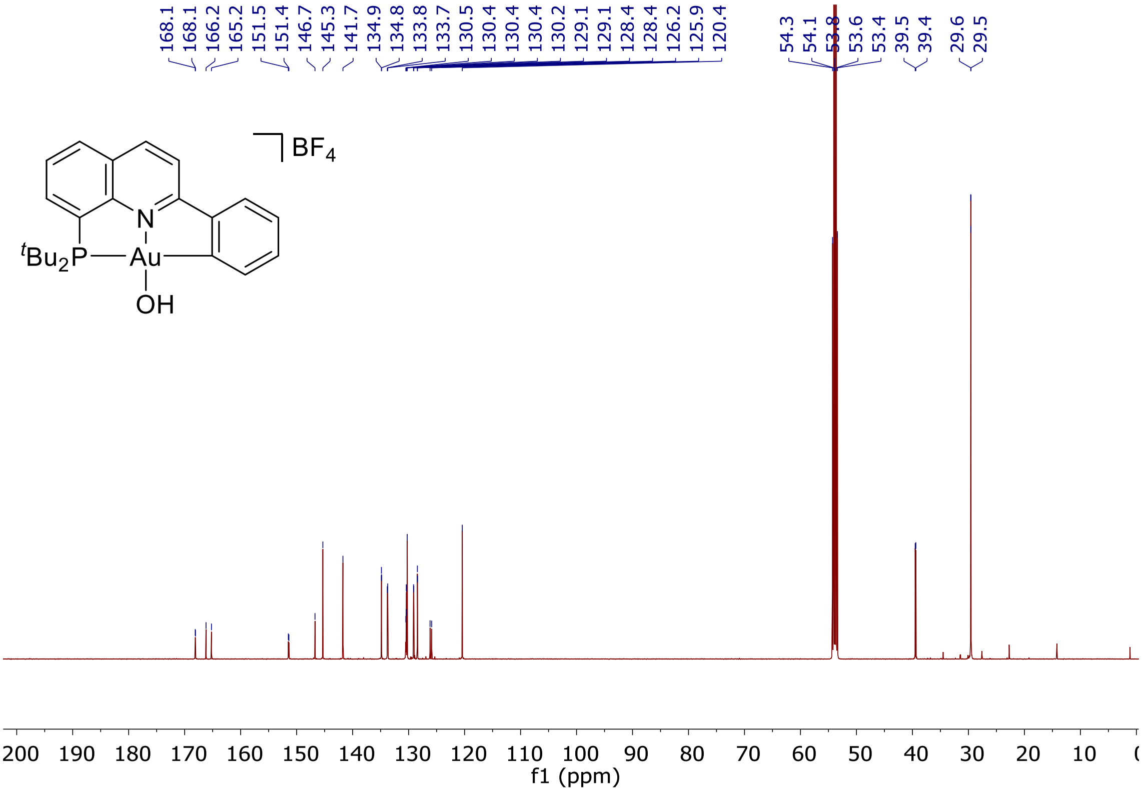

Figure S30. $^{13}C\{^{1}H\}$ NMR spectrum (125.82 MHz, CD_2Cl_2, 298 K) of compound **2^{H}**.

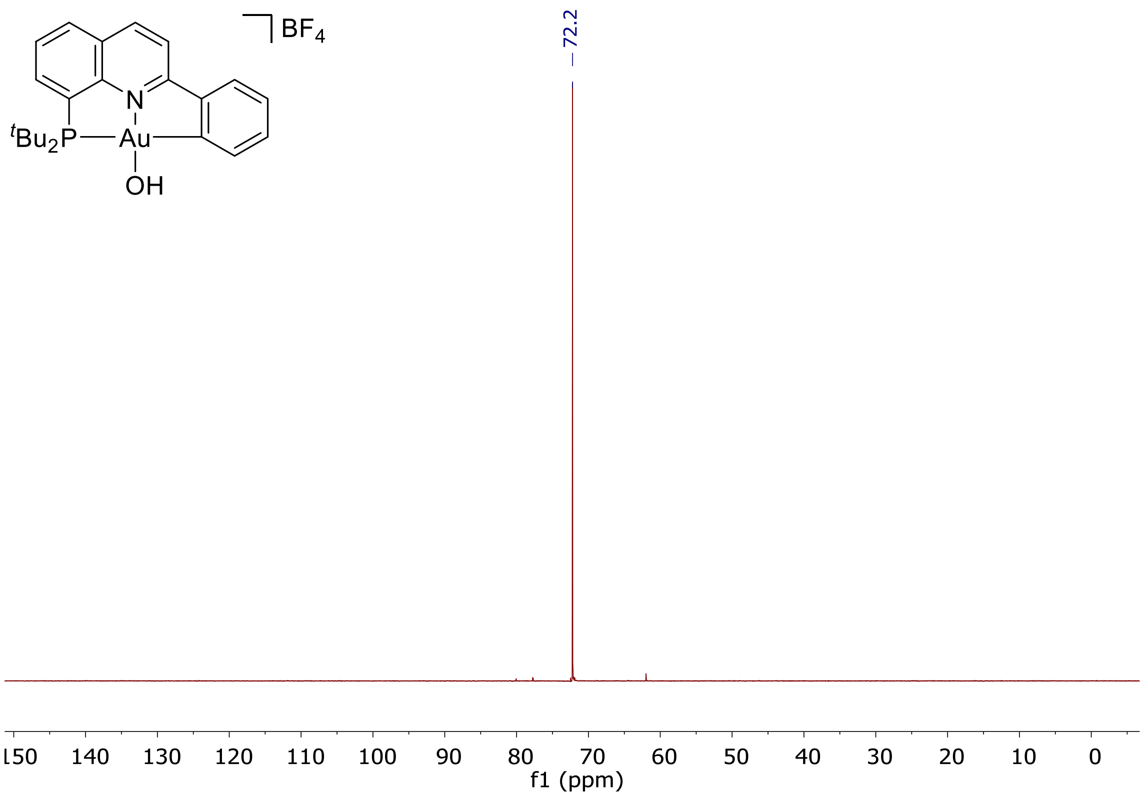

Figure S31. $^{31}P\{^{1}H\}$ NMR spectrum (202.52 MHz, CD_2Cl_2, 298 K) of compound $\mathbf{2^{H}}$.

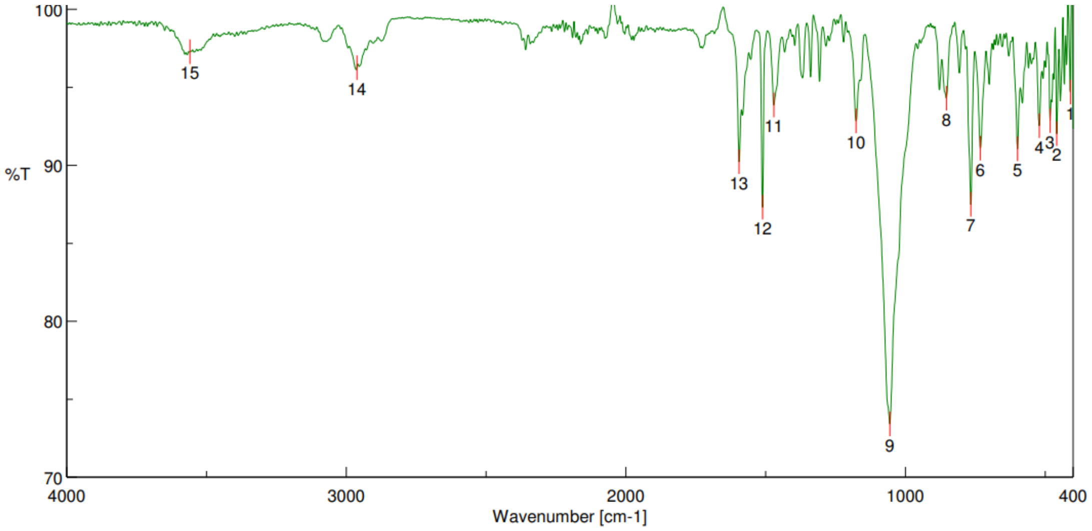

[Result of Peak Picking]

No.	Position	Intensity	No.	Position	Intensity	No.	Position	Intensity	No.	Position	Intensity
1	409.8	94.7	2	459.0	92.0	3	482.1	92.9	4	520.7	92.5
5	598.8	91.0	6	731.9	91.1	7	765.6	87.5	8	853.4	94.3
9	1055.8	73.4	10	1176.4	92.8	11	1470.5	93.9	12	1511.0	87.3
13	1594.8	90.2	14	2962.1	96.3	15	3559.0	97.3			

Figure S32. IR spectrum of compound $\mathbf{2^{H}}$.

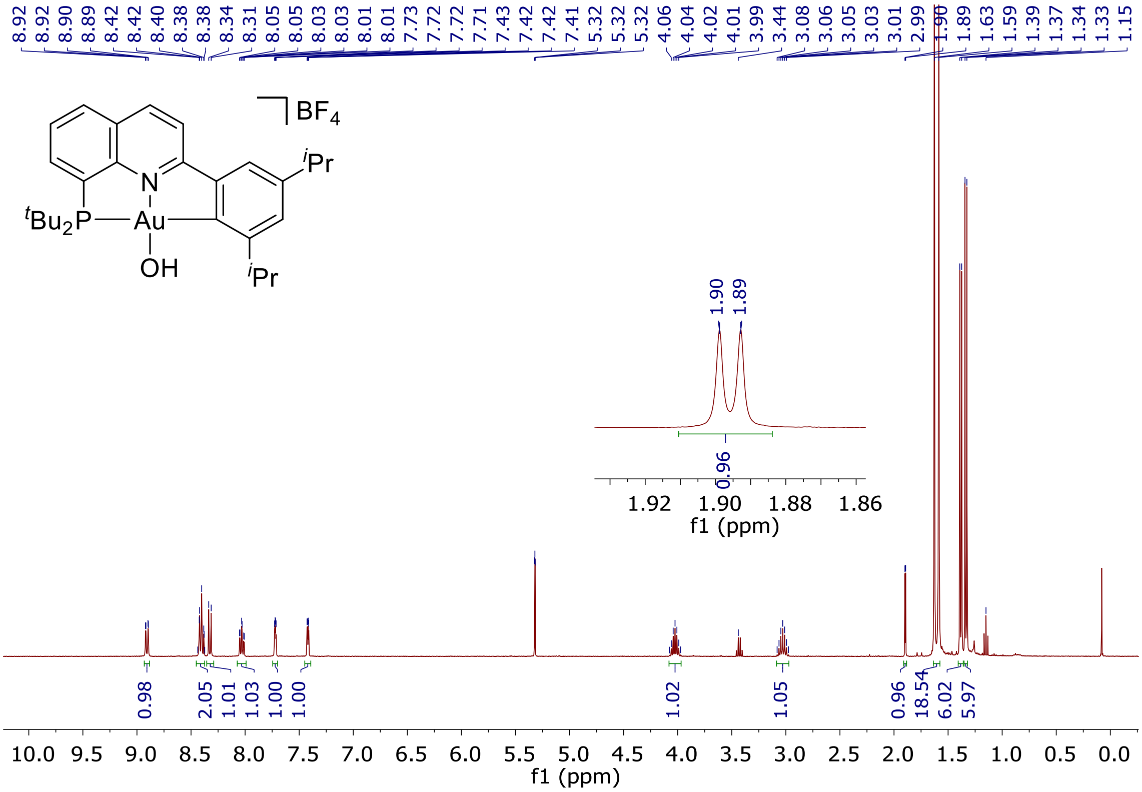

Figure S33. ^{1}H NMR spectrum (400.13 MHz, CD_2Cl_2, 298 K) of compound **2^{iPr}** (<2% residual diethyl ether).

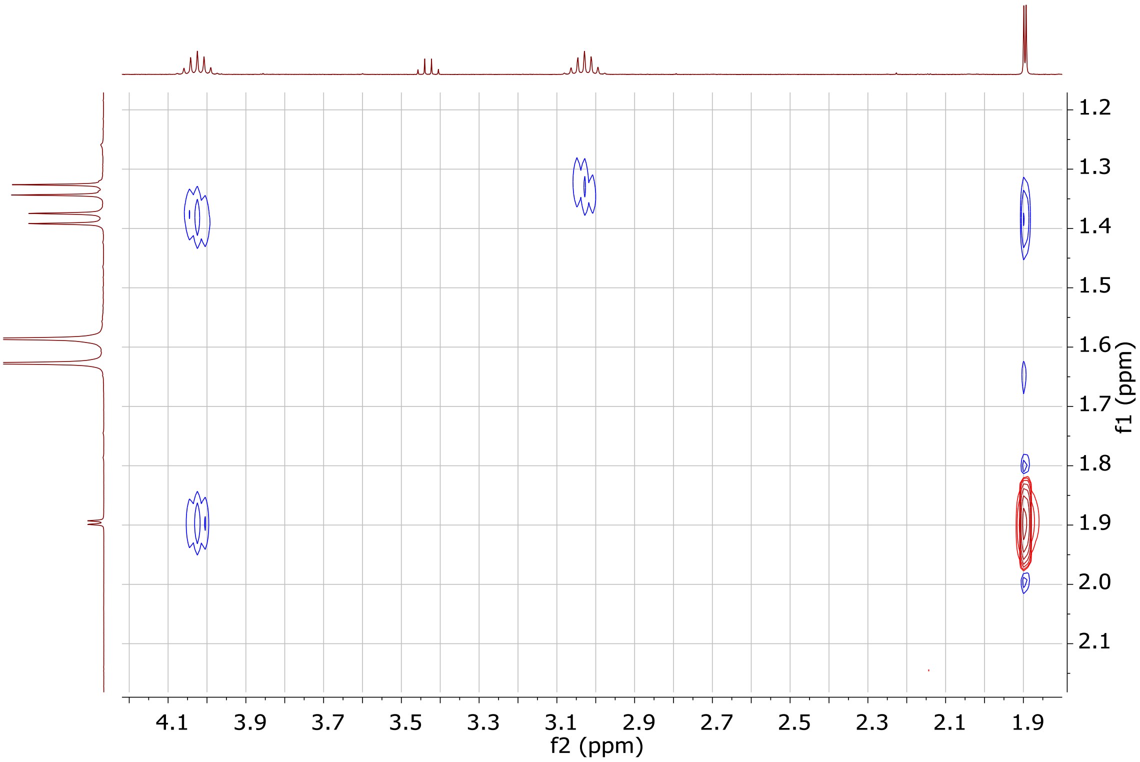

Figure S34. ^{1}H-^{1}H NOESY NMR spectrum (400.13 MHz, CD_2Cl_2, 298 K) of compound **2^{iPr}**. Expansion of O*H* cross peaks.

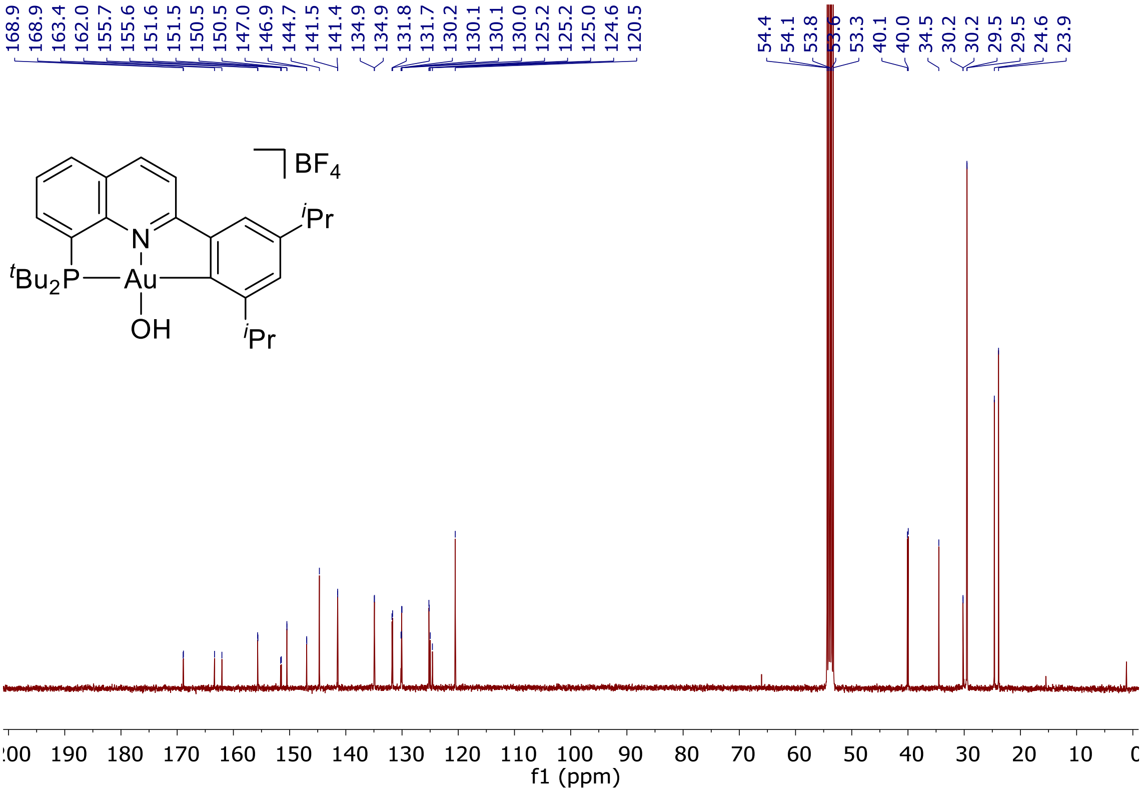

Figure S35. $^{13}C\{^{1}H\}$ NMR spectrum (100.62 MHz, CD_2Cl_2, 298 K) of compound **2^{iPr}**.

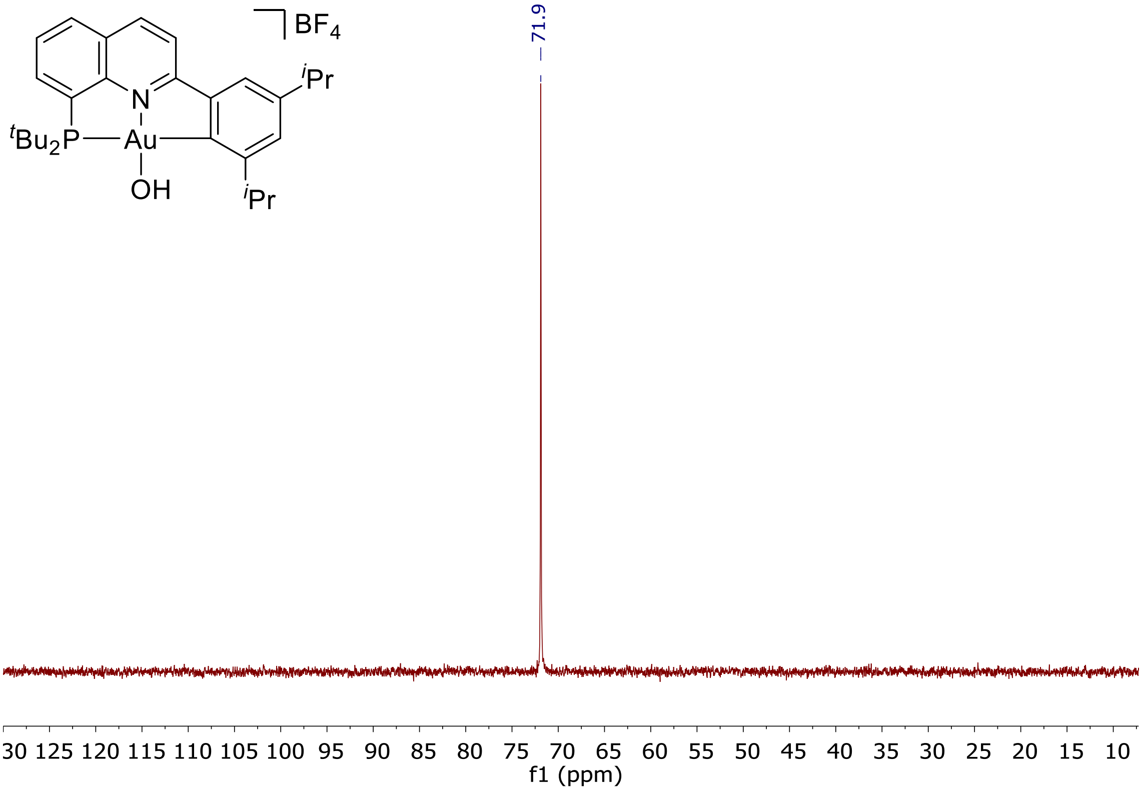

Figure S36. $^{31}P\{^{1}H\}$ NMR spectrum (161.99 MHz, CD_2Cl_2, 298 K) of compound **2^{iPr}**.

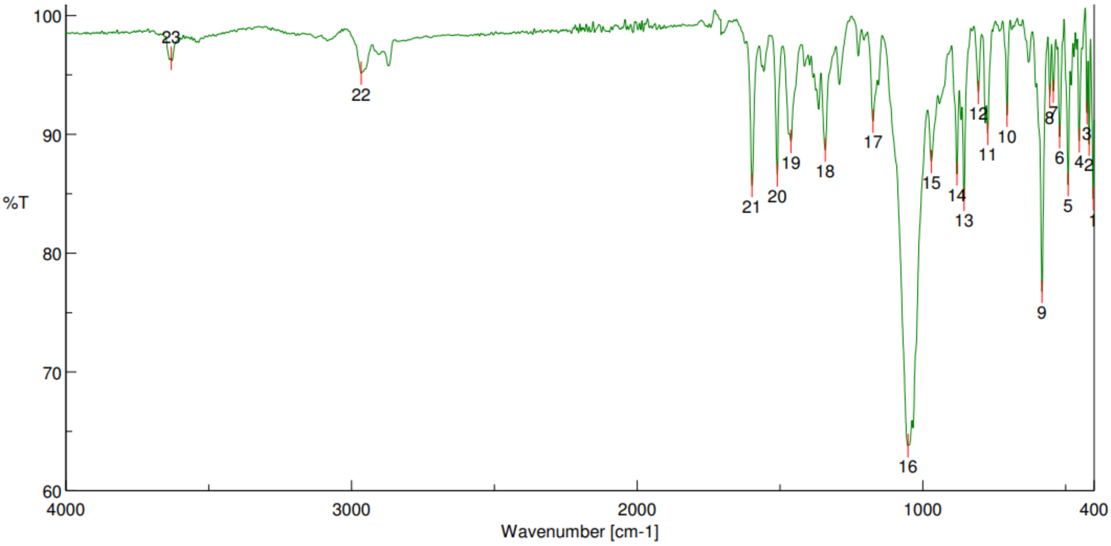

[Result of Peak Picking]

No.	Position	Intensity	No.	Position	Intensity	No.	Position	Intensity	No.	Position	Intensity
1	403.1	84.6	2	417.5	89.2	3	425.2	91.8	4	452.2	89.4
5	490.8	85.8	6	520.7	89.8	7	542.9	93.6	8	555.4	93.2
9	582.4	76.8	10	704.9	91.6	11	772.4	90.0	12	805.1	93.5
13	855.3	84.5	14	880.4	86.7	15	970.0	87.7	16	1052.0	63.8
17	1174.4	91.1	18	1341.3	88.7	19	1461.8	89.4	20	1509.0	86.6
21	1597.7	85.7	22	2966.0	95.1	23	3631.3	96.4			

Figure S37. IR spectrum of compound **2**^{iPr}.

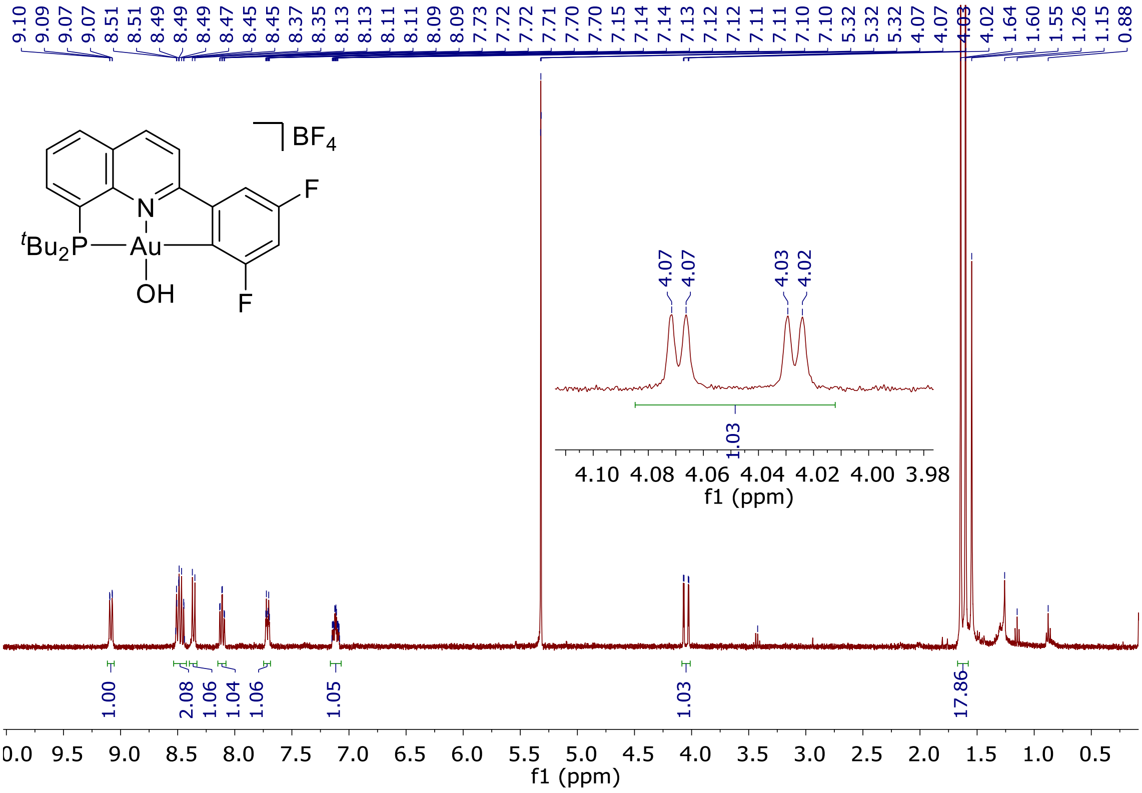

Figure S38. ^{1}H NMR spectrum (400.13 MHz, CD_2Cl_2, 298 K) of compound **2**^{F}.

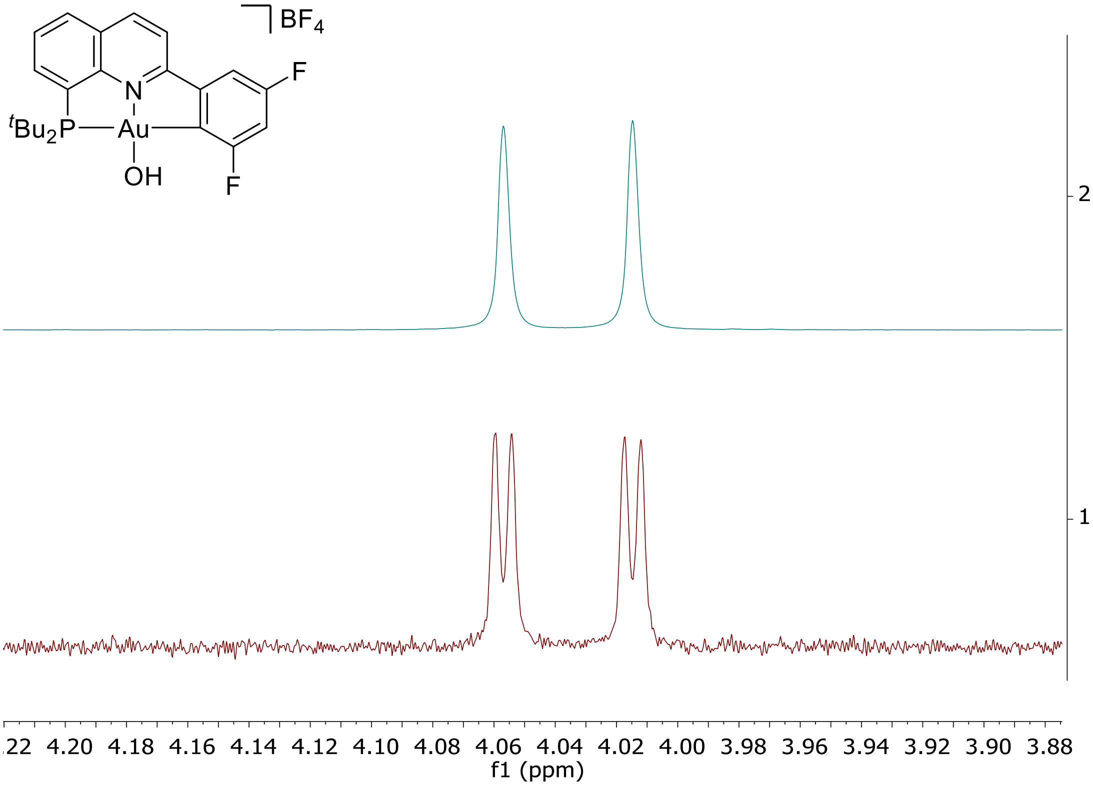

Figure S39. Au-O*H* region for the $^{1}H\{^{31}P\}$ (top) vs ^{1}H (bottom) NMR spectra (400.13 MHz, CD_2Cl_2, 298 K) of compound **2^F^**.

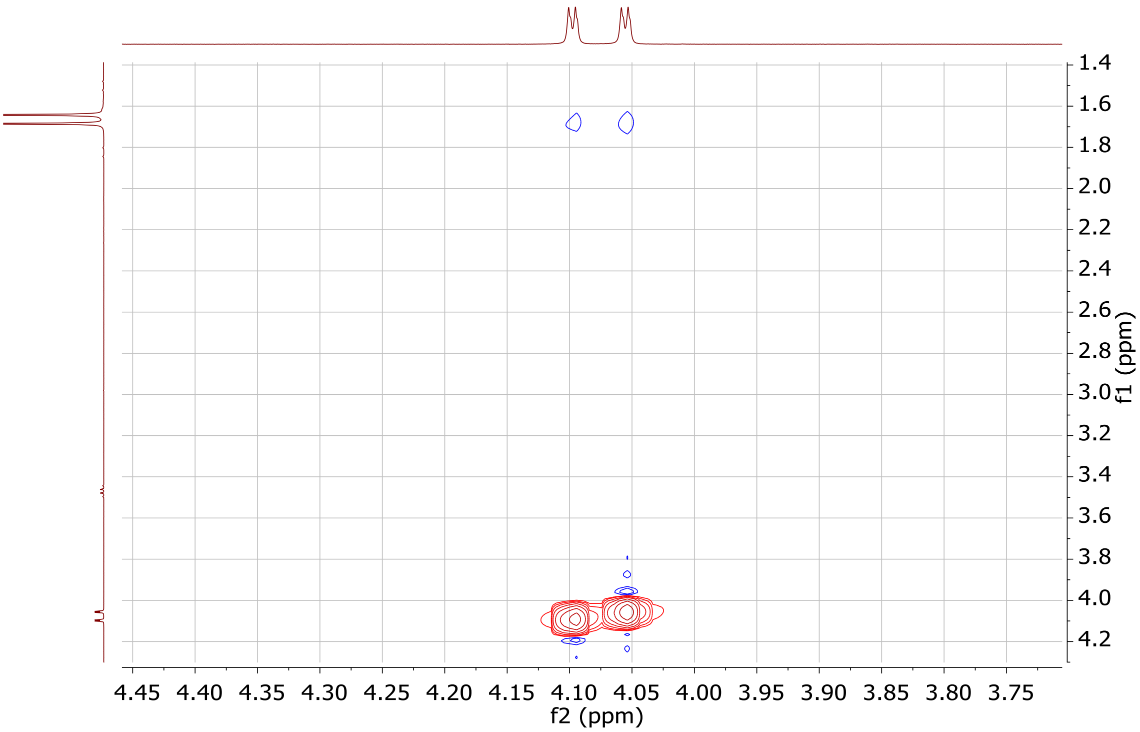

Figure S40. ^{1}H-^{1}H NOESY NMR spectrum (400.13 MHz, CD_2Cl_2, 298 K) of compound **2^F^**. Expansion of O*H* cross peaks.

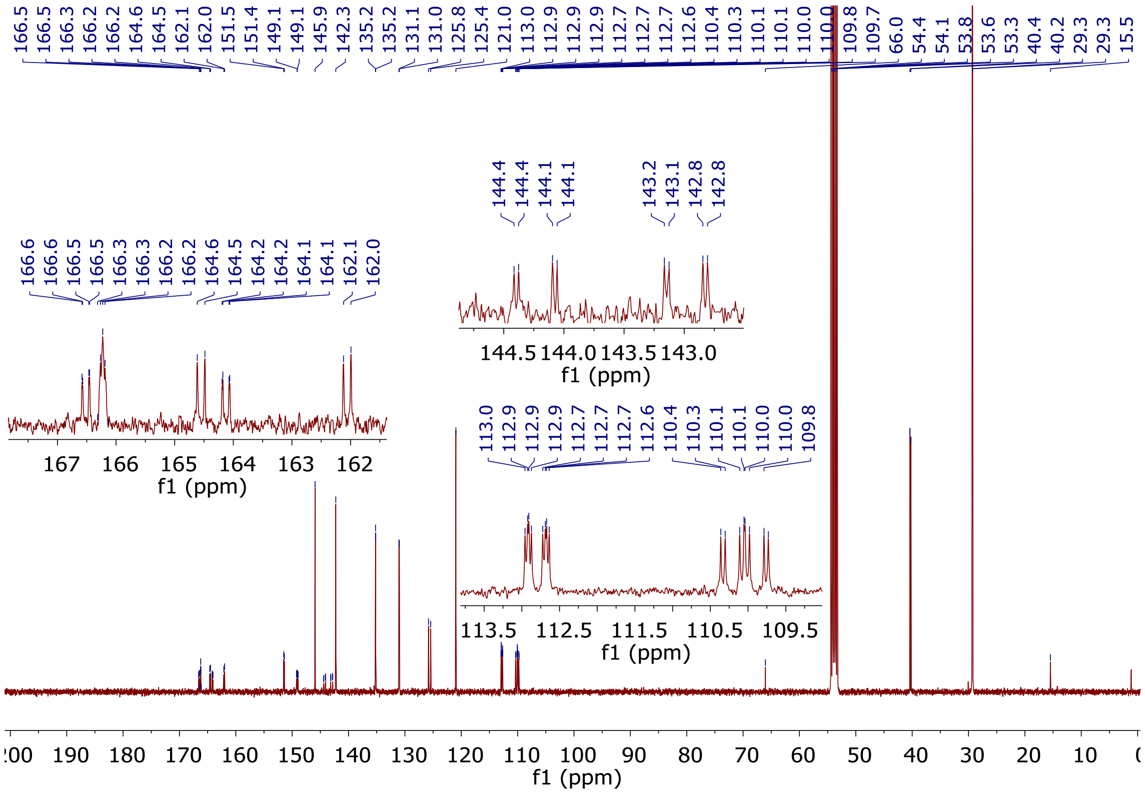

Figure S41. $^{13}C\{^{1}H\}$ NMR spectrum (100.62 MHz, CD_2Cl_2, 298 K) of compound **2^F** (residual Et_2O).

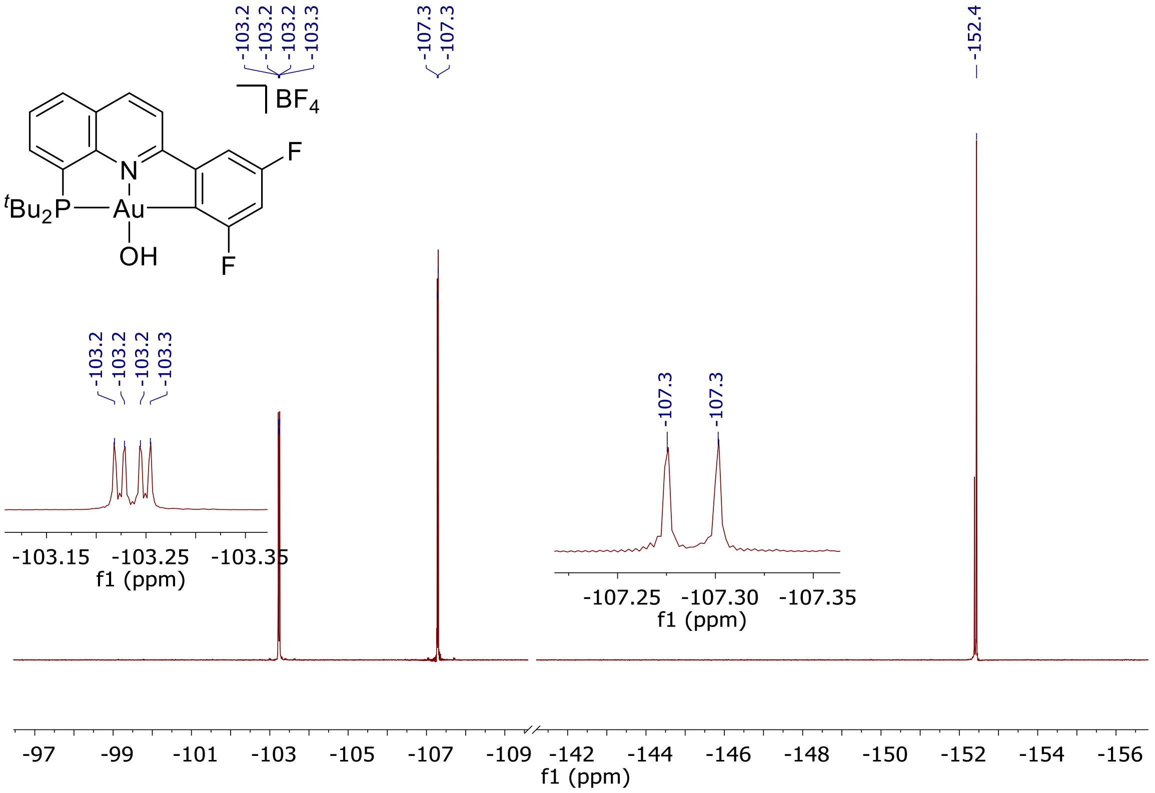

Figure S42. $^{19}F\{^{1}H\}$ NMR spectrum (376.50 MHz, CD_2Cl_2, 298 K) of compound **2^F**.

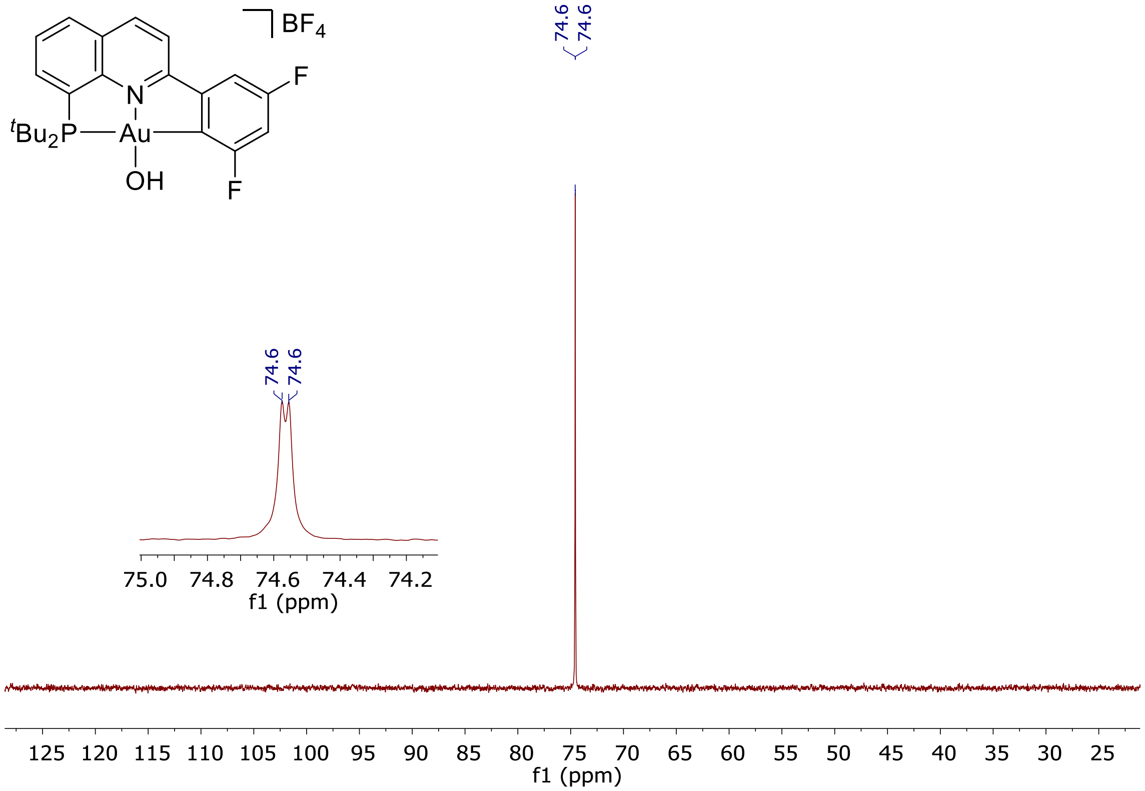

Figure S43. $^{31}P\{^1H\}$ NMR spectrum (161.99 MHz, CD_2Cl_2, 298 K) of compound **2**$^{\mathbf{F}}$.

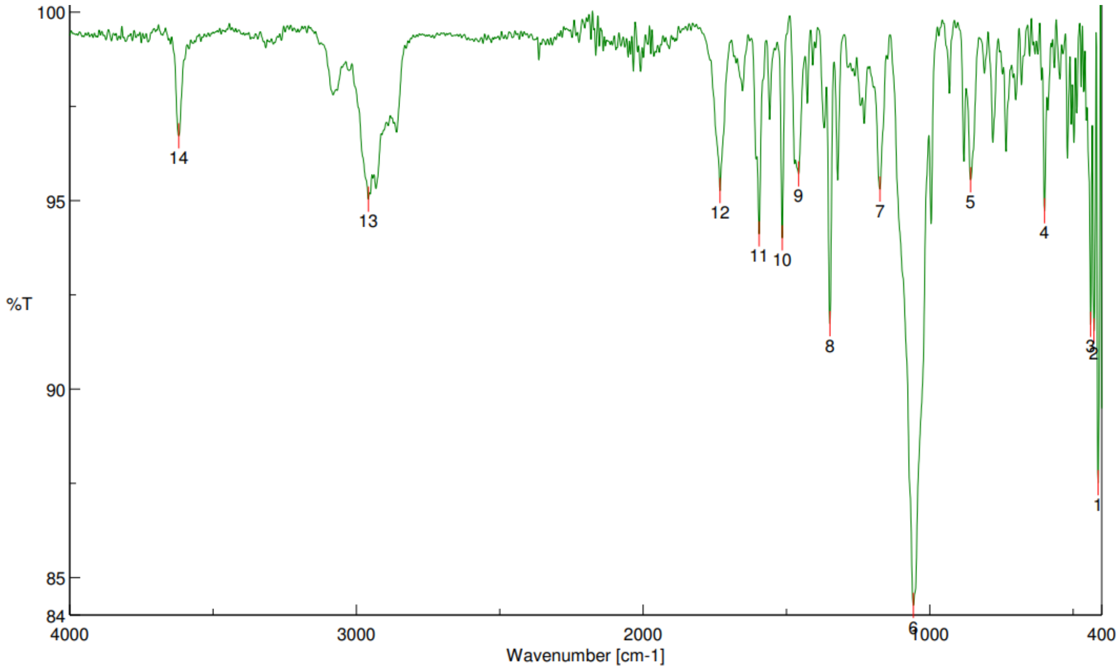

[Result of Peak Picking]

No.	Position	Intensity	No.	Position	Intensity	No.	Position	Intensity	No.	Position	Intensity
1	412.7	87.5	2	427.2	91.5	3	438.7	91.7	4	599.8	94.7
5	857.2	95.5	6	1056.8	84.3	7	1173.5	95.3	8	1348.0	91.7
9	1457.0	95.7	10	1514.8	94.0	11	1594.8	94.1	12	1731.8	95.3
13	2958.3	95.0	14	3619.7	96.7						

Figure S44. IR spectrum of compound **2**$^{\mathbf{F}}$.

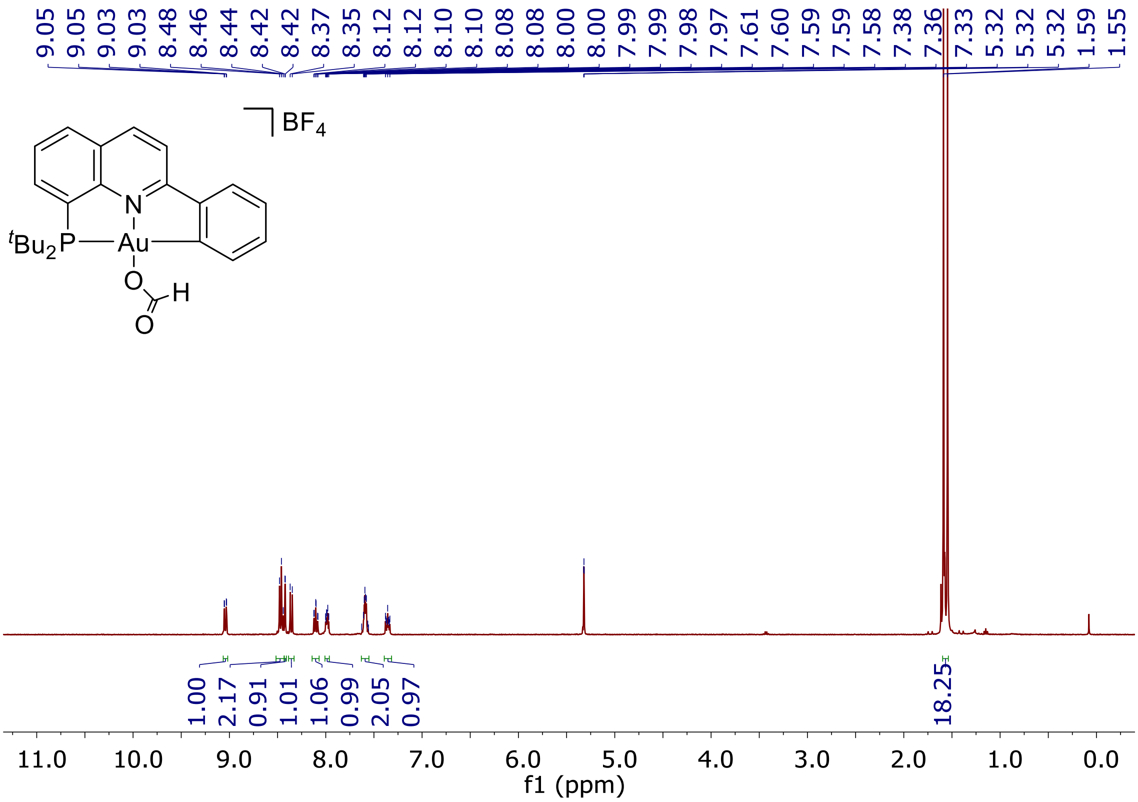

Figure S45. ^{1}H NMR spectrum (400.13 MHz, CD_2Cl_2, 298 K) of compound **3^H**.

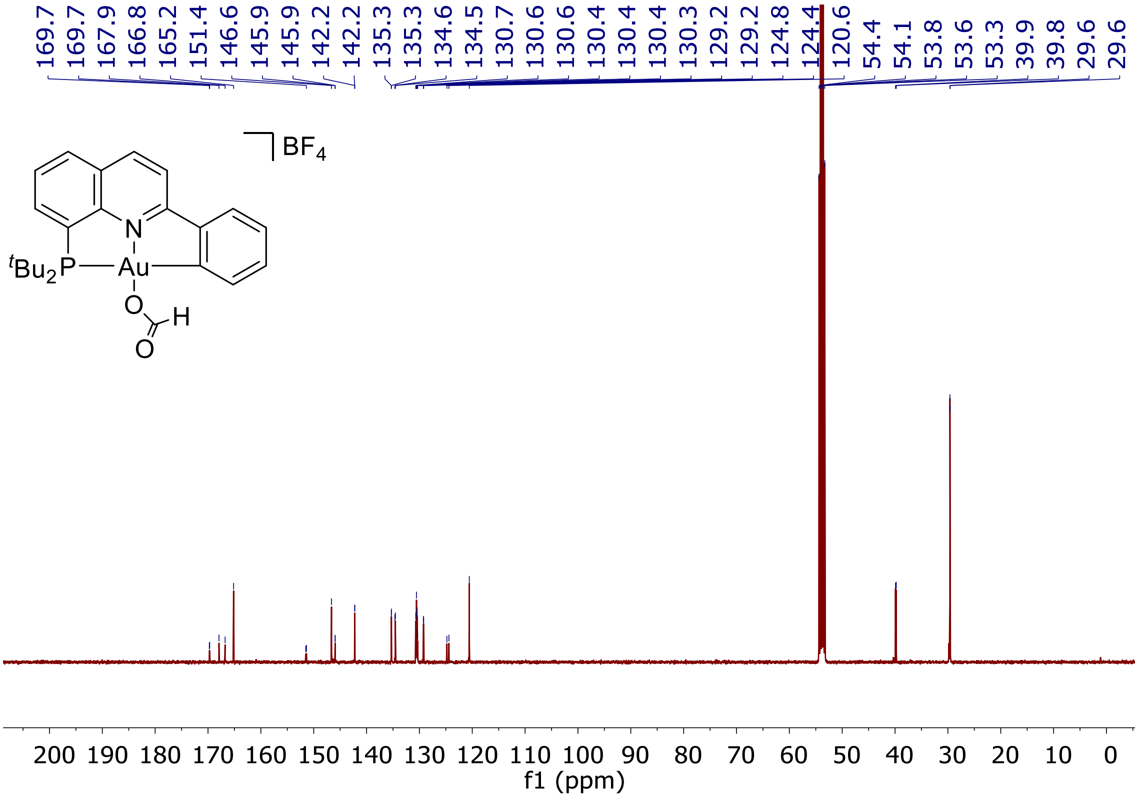

Figure S46. $^{13}C\{^1H\}$ NMR spectrum (100.62 MHz, CD_2Cl_2, 298 K) of compound **3^H**.

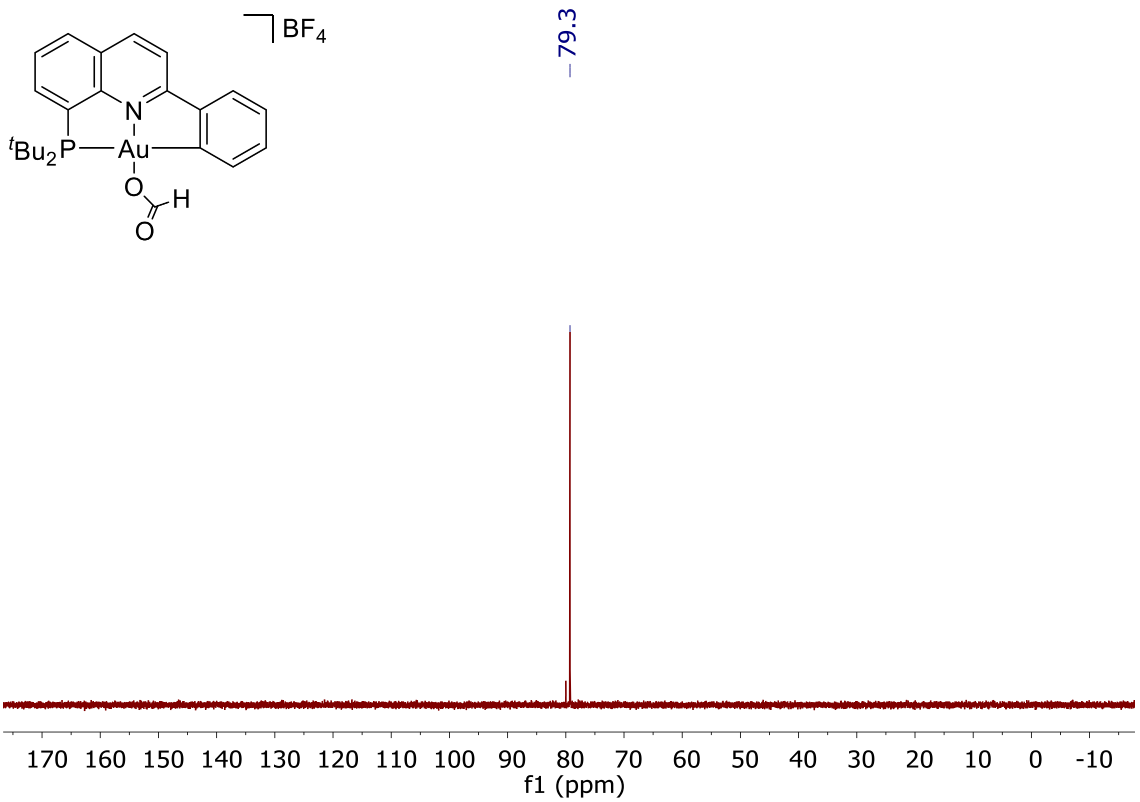

Figure S47. $^{31}P\{^{1}H\}$ NMR spectrum (161.99 MHz, CD_2Cl_2, 298 K) of compound $\mathbf{3^{H}}$.

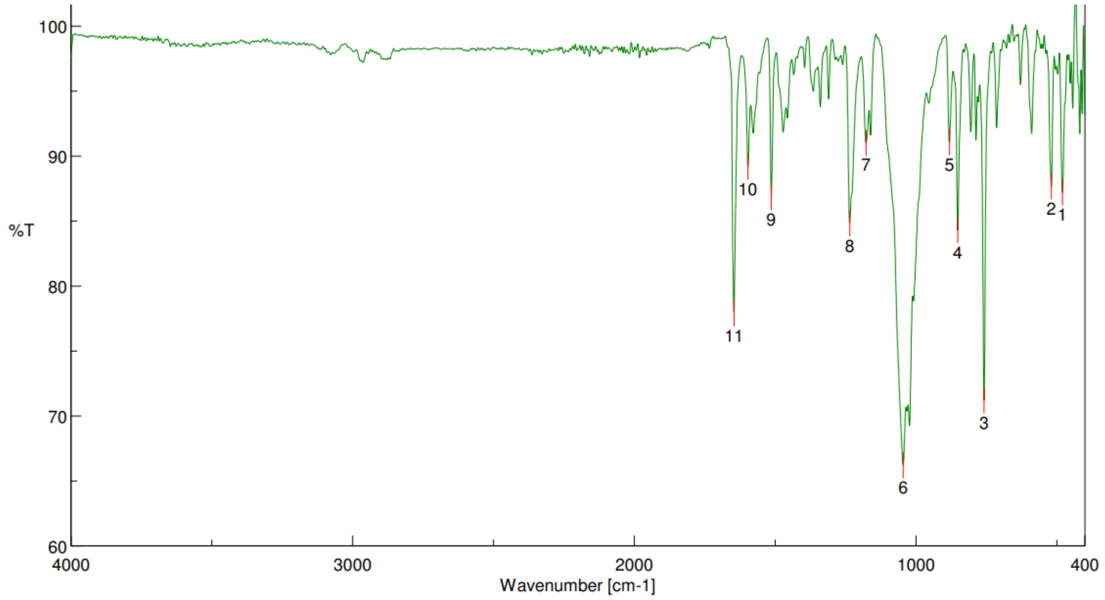

[Result of Peak Picking]

No.	Position	Intensity	No.	Position	Intensity	No.	Position	Intensity	No.	Position	Intensity
1	480.2	87.2	2	519.7	87.7	3	758.9	71.2	4	851.4	84.3
5	881.3	91.1	6	1045.2	66.2	7	1177.3	91.0	8	1235.2	84.8
9	1513.9	86.8	10	1596.8	89.1	11	1646.0	77.9			

Figure S48. IR spectrum of compound $\mathbf{3^{H}}$.

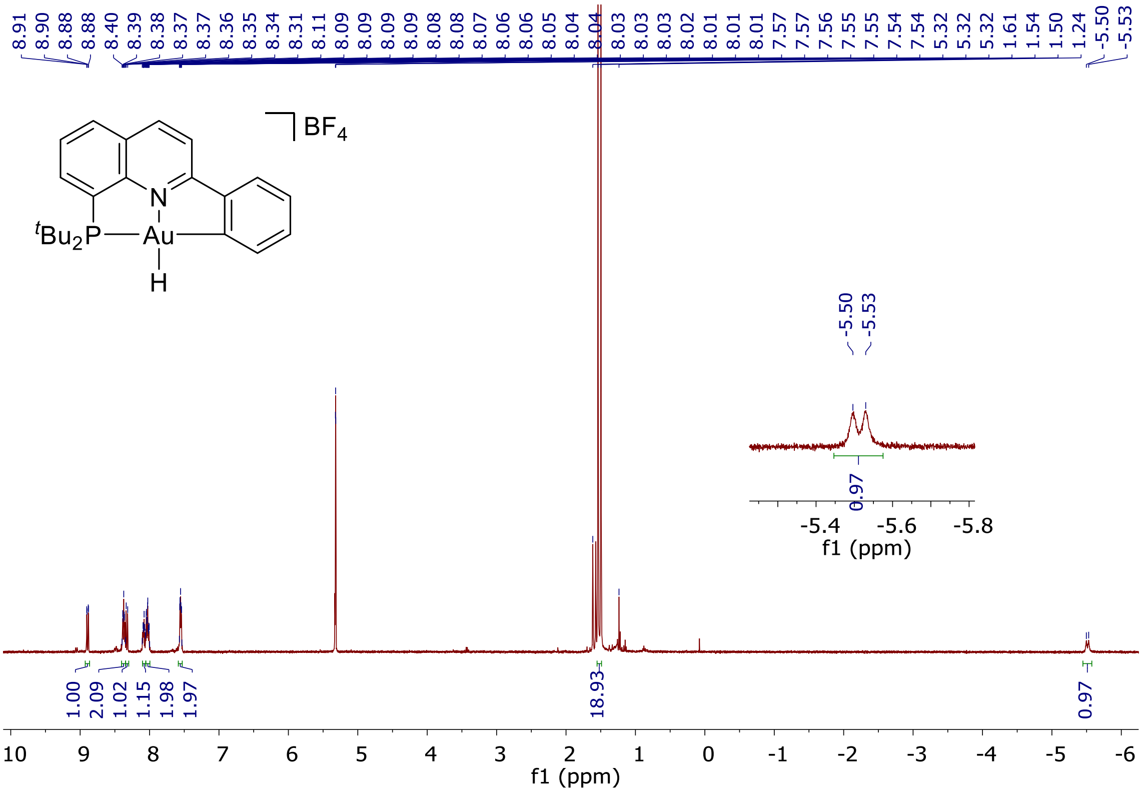

Figure S49. ^{1}H NMR spectrum (400.13 MHz, CD_2Cl_2, 298 K) of compound $\mathbf{4^H}$.

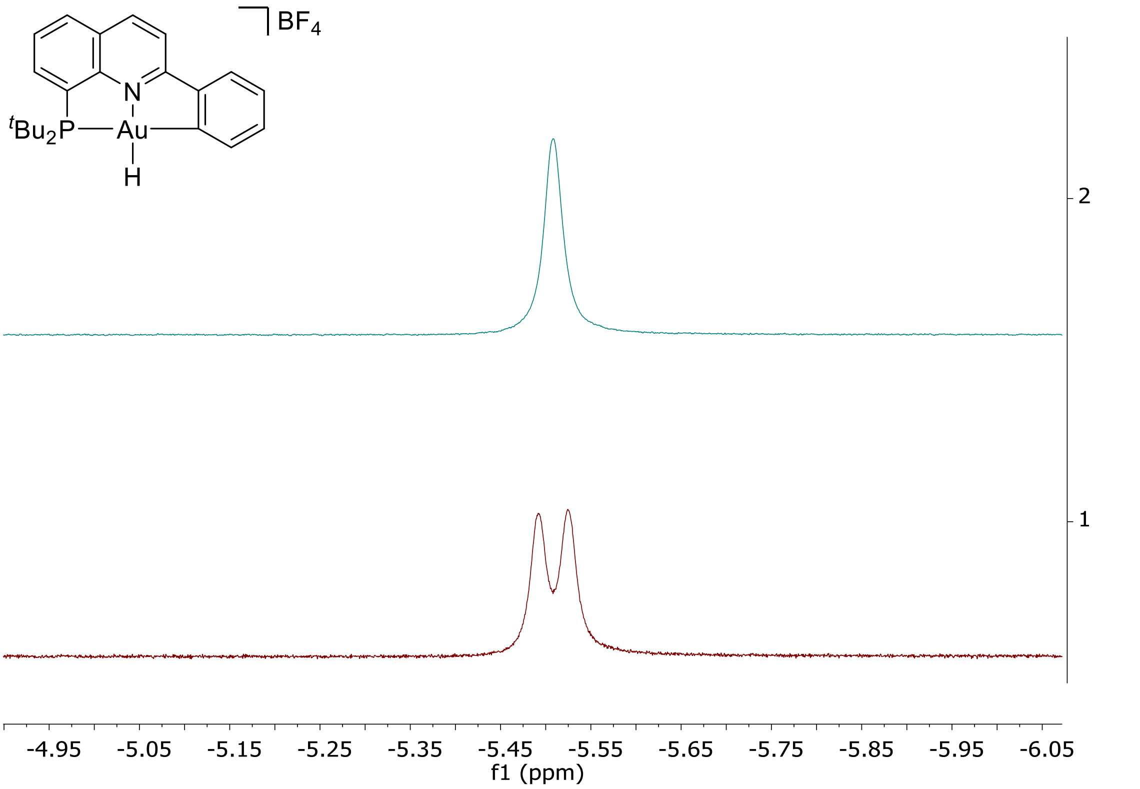

Figure S50. High-field region of the $^{1}H\{^{31}P\}$ (top) vs ^{1}H (bottom) NMR spectra (400.13 MHz, CD_2Cl_2, 298 K) of compound $\mathbf{4^H}$.

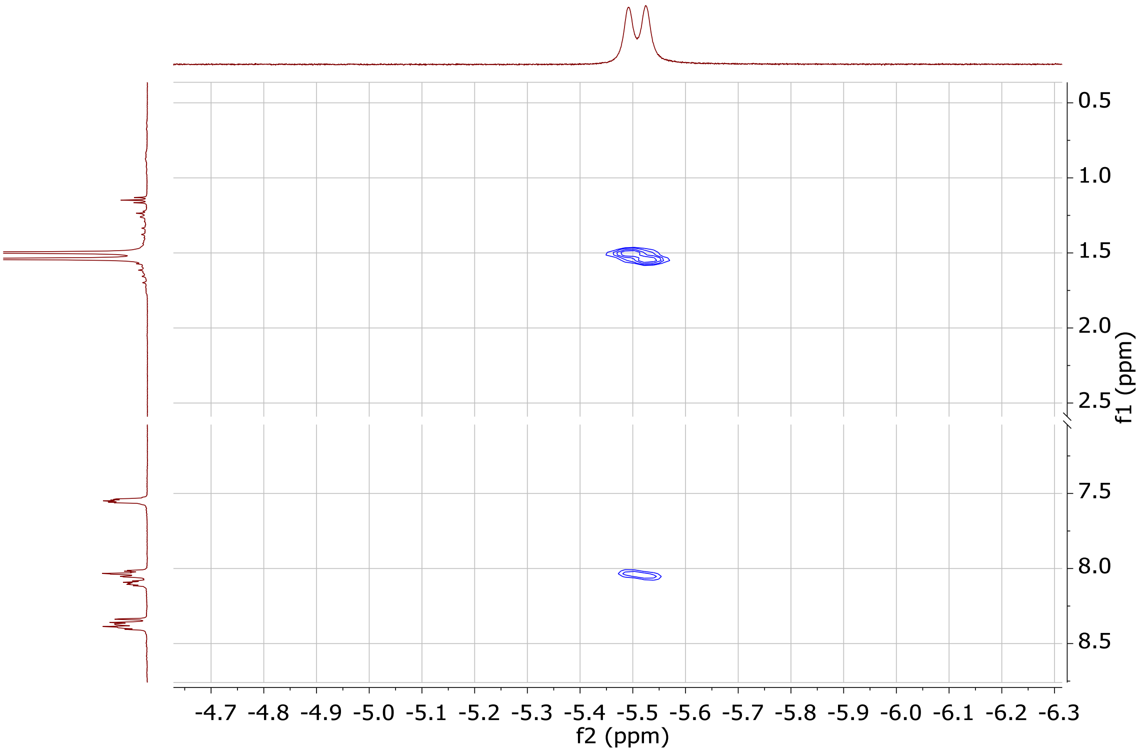

Figure S51. ^{1}H-^{1}H NOESY NMR spectrum (400.13 MHz, CD_2Cl_2, 298 K) of compound **4^{H}**. Expansion of Au-hydride cross peaks.

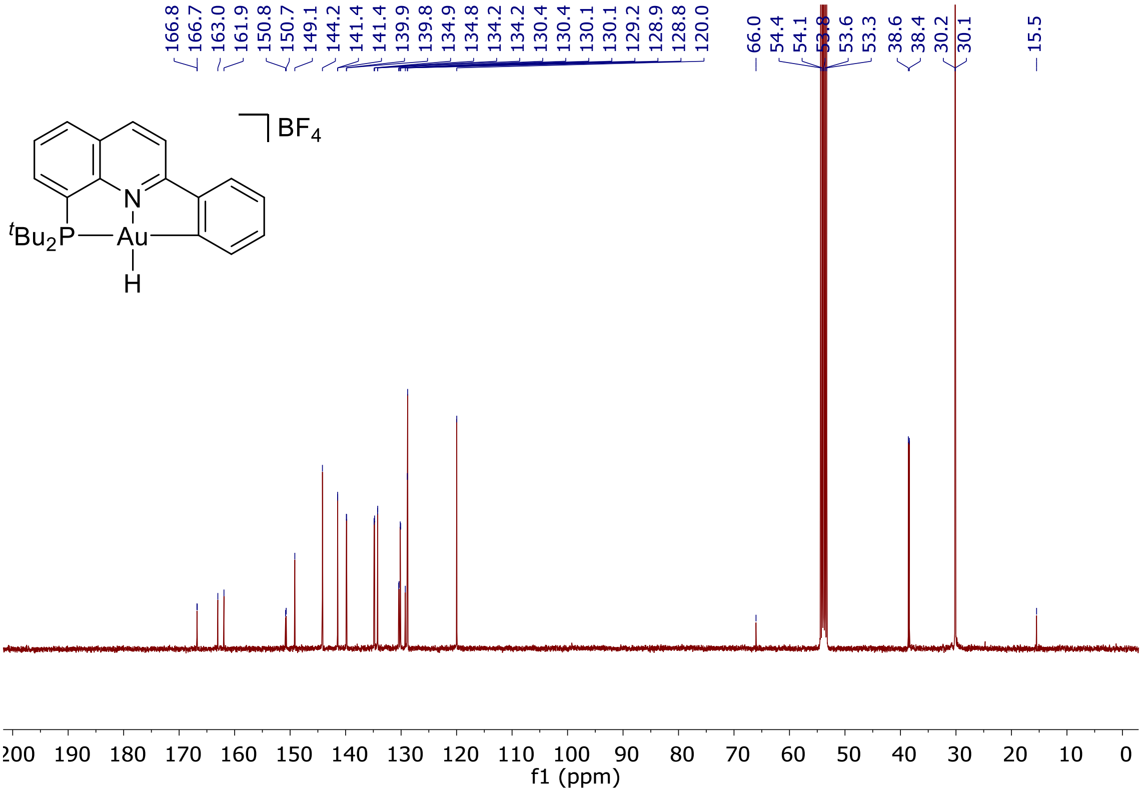

Figure S52. $^{13}C\{^{1}H\}$ NMR spectrum (100.62 MHz, CD_2Cl_2, 298 K) of compound **4^{H}** (residual Et_2O).

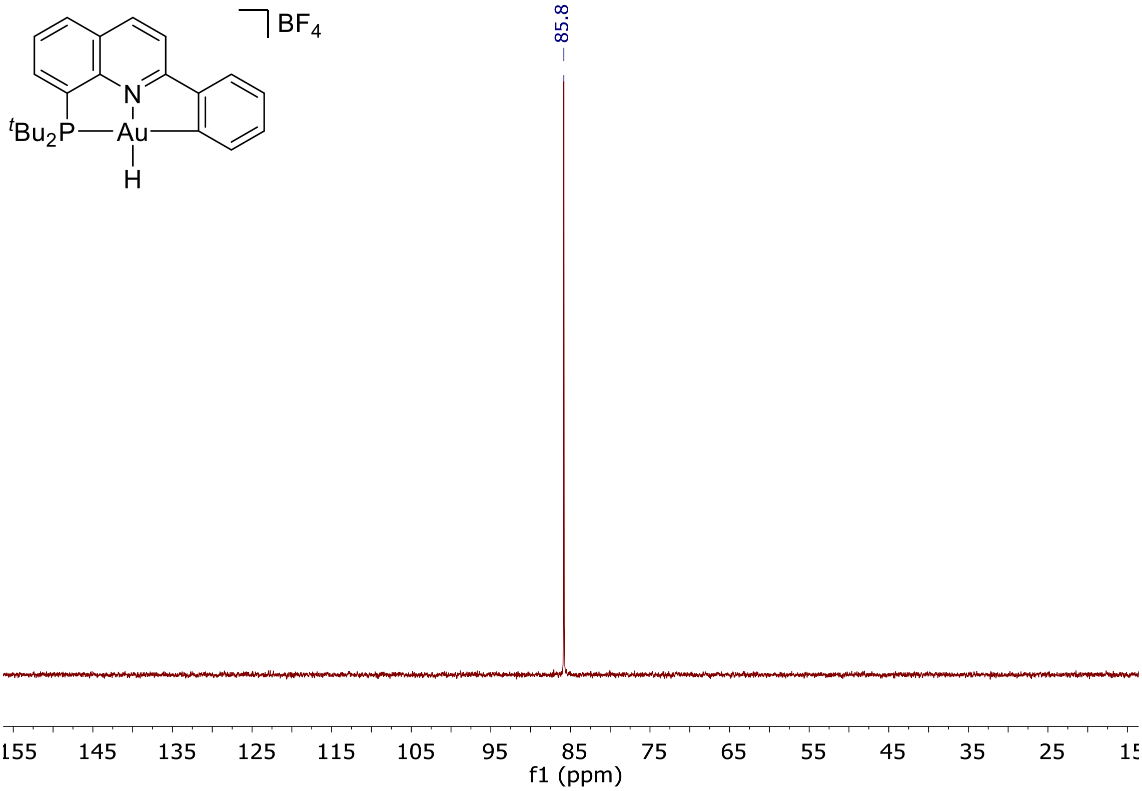

Figure S53. $^{31}P\{^{1}H\}$ NMR spectrum (161.99 MHz, CD_2Cl_2, 298 K) of compound $\mathbf{4^{H}}$.

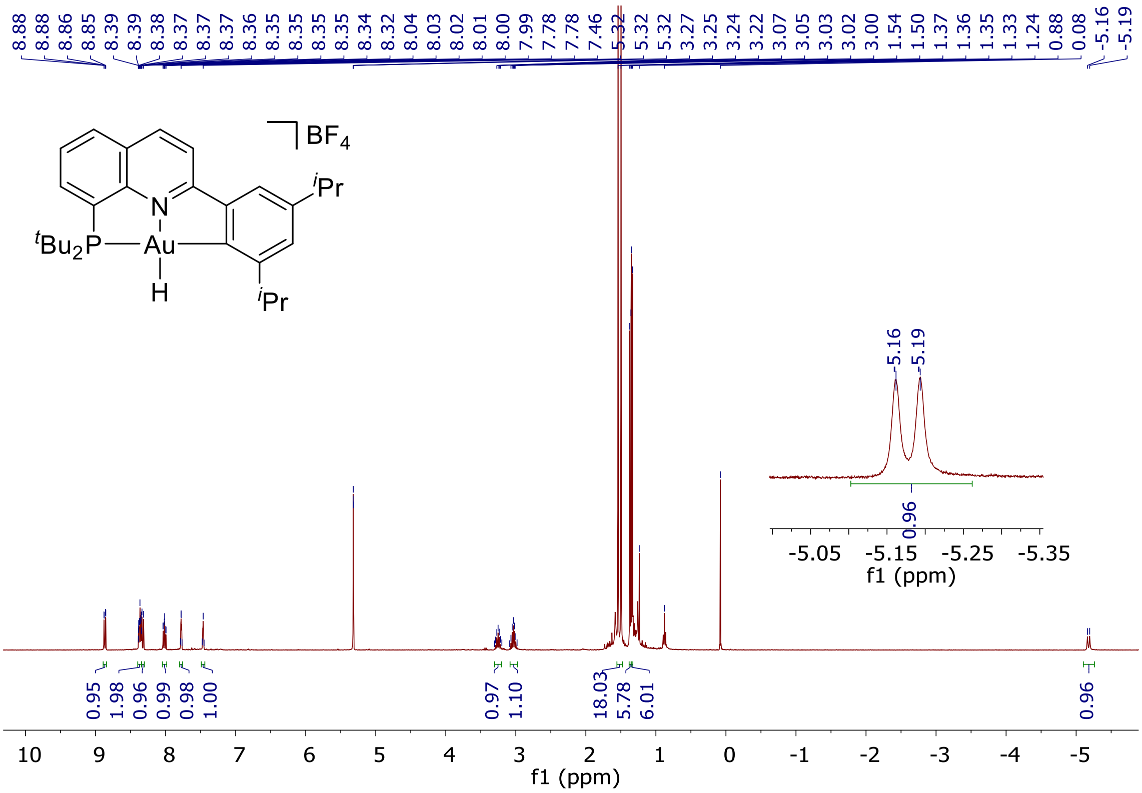

Figure S54. ^{1}H NMR spectrum (400.13 MHz, CD_2Cl_2, 298 K) of compound $\mathbf{4^{iPr}}$.

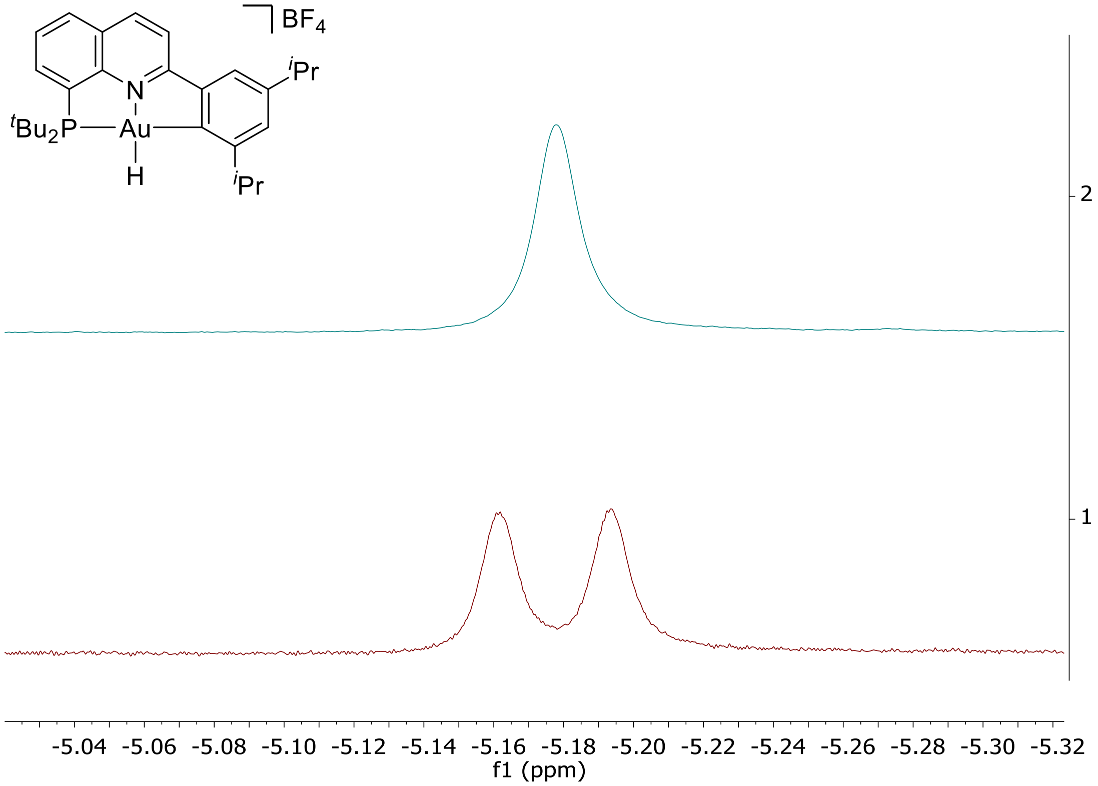

Figure S55. High-field region of the $^{1}H\{^{31}P\}$ (top) vs ^{1}H (bottom) NMR spectra (400.13 MHz, CD_2Cl_2, 298 K) of compound **4^{iPr}**.

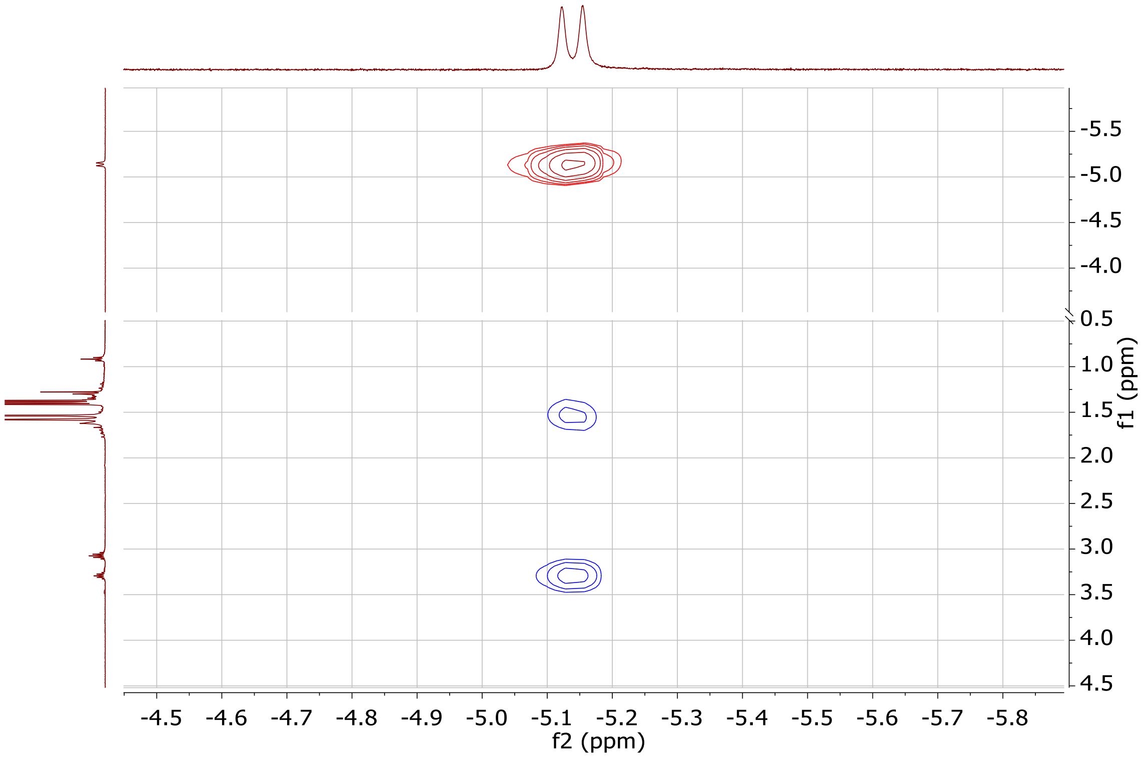

Figure S56. ^{1}H-^{1}H NOESY NMR spectrum (400.13 MHz, CD_2Cl_2, 298 K) of compound **4^{iPr}**. Expansion of Au-hydride cross peaks.

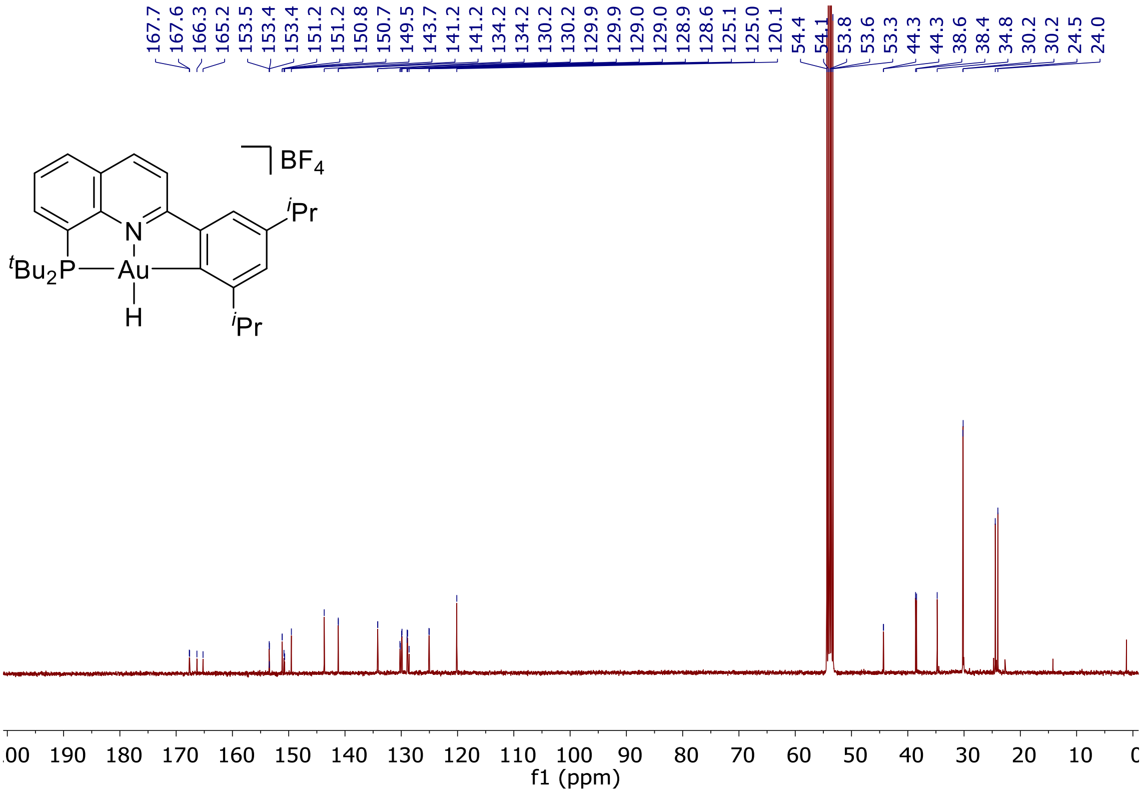

Figure S57. $^{13}C\{^1H\}$ NMR spectrum (100.62 MHz, CD_2Cl_2, 298 K) of compound **4^{iPr}**.

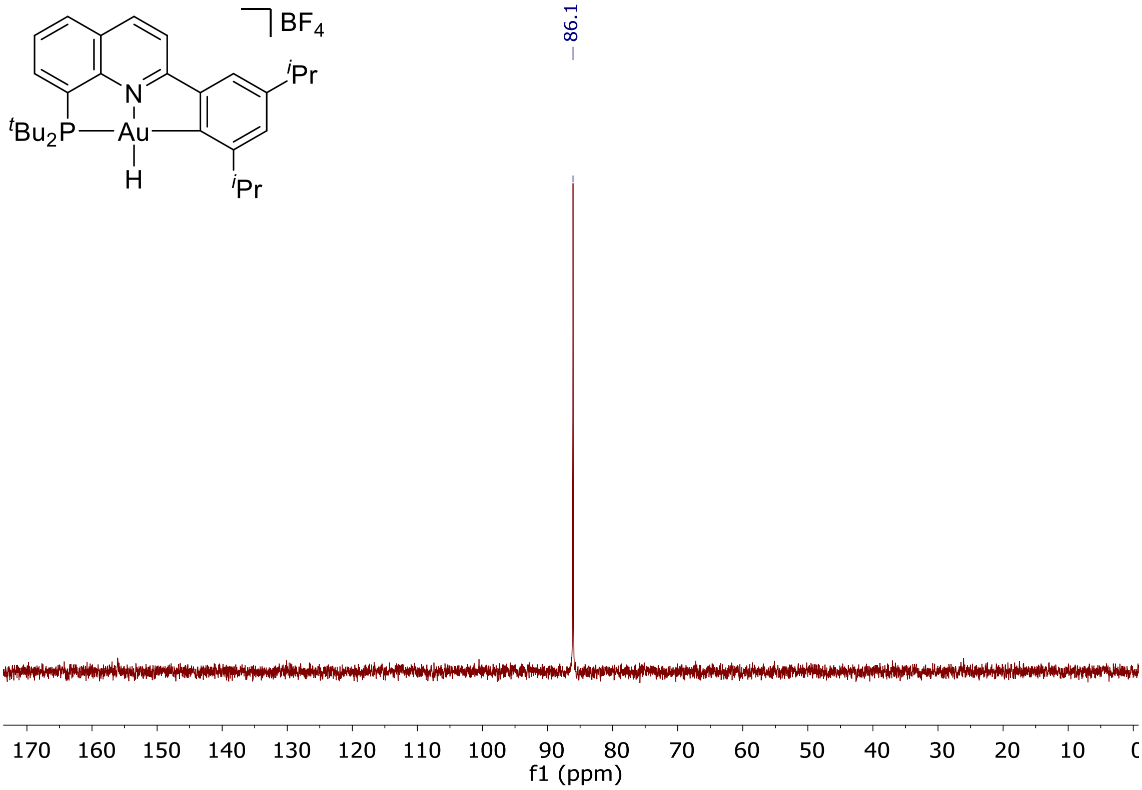

Figure S58. $^{31}P\{^1H\}$ NMR spectrum (161.99 MHz, CD_2Cl_2, 298 K) of compound **4^{iPr}**.

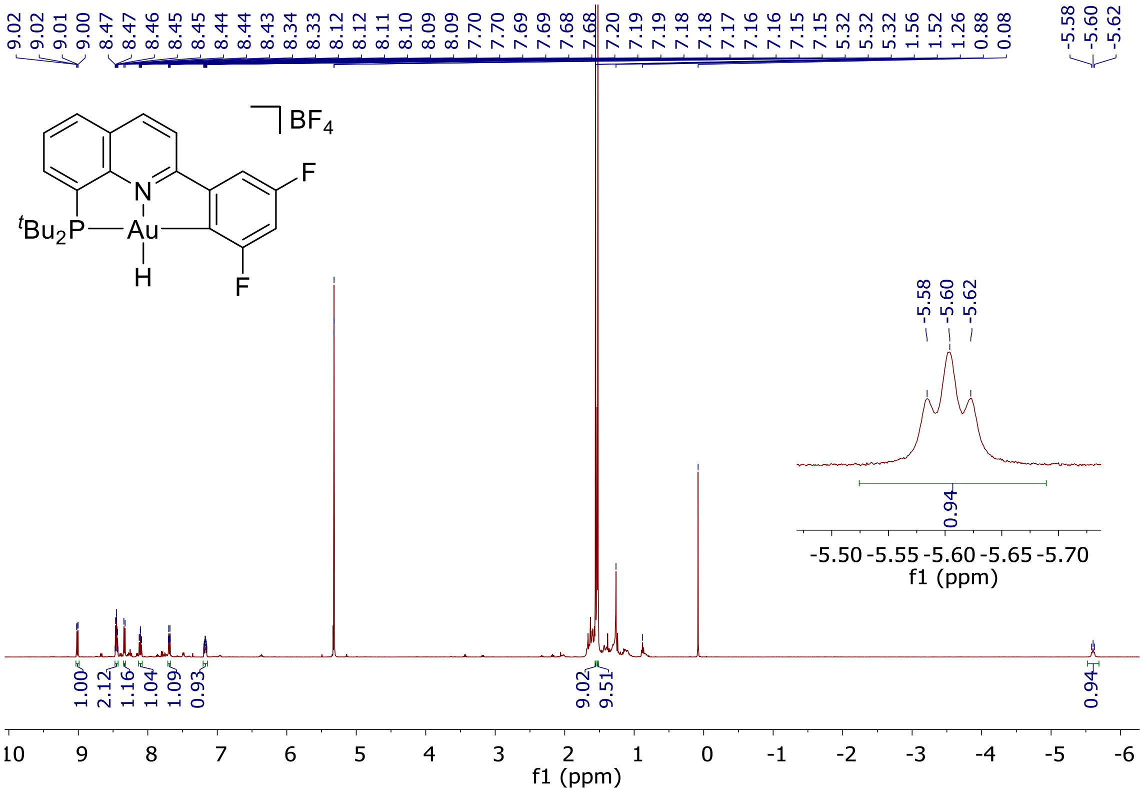

Figure S59. ^{1}H NMR spectrum (500.30 MHz, CD_2Cl_2, 298 K) of compound **4^F^**.

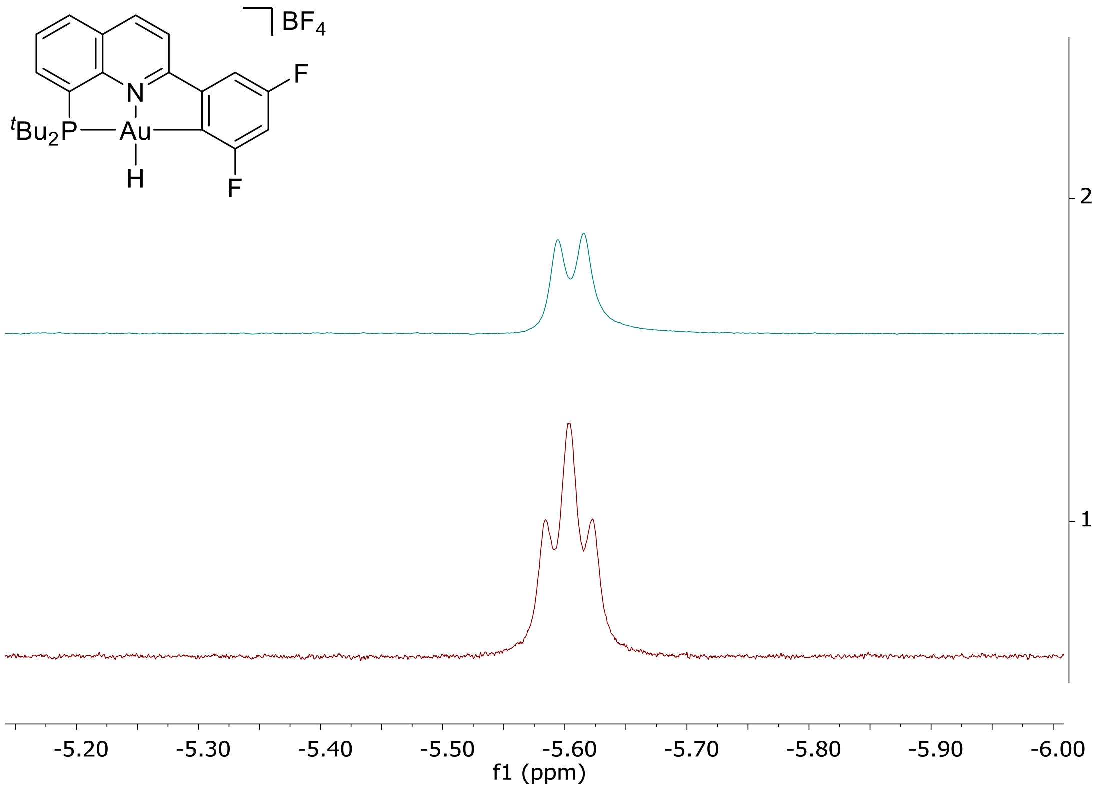

Figure S60. High-field region of the $^{1}H\{^{31}P\}$ (top) vs ^{1}H (bottom) NMR spectra (500.30 MHz, CD_2Cl_2, 298 K) of compound $\mathbf{4^{F}}$.

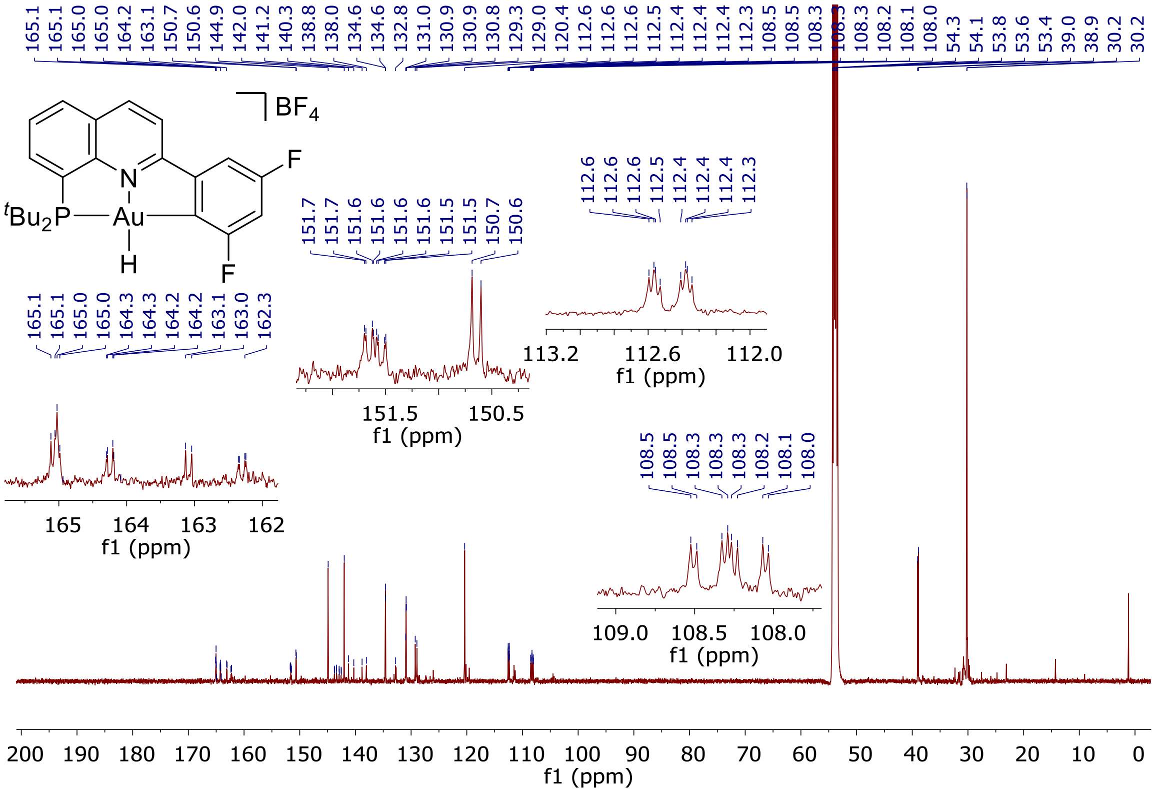

Figure S61. $^{13}C\{^1H\}$ NMR spectrum (125.81 MHz, CD_2Cl_2, 298 K) of compound $\mathbf{4^F}$.

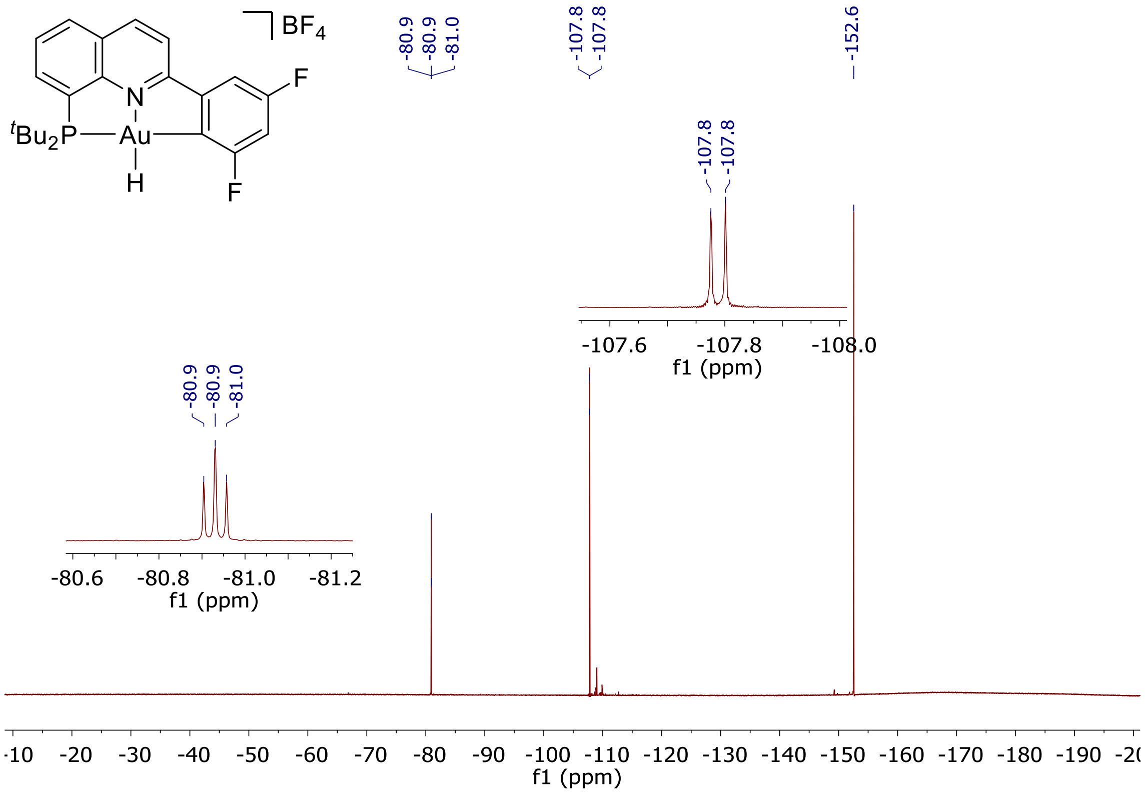

Figure S62. $^{19}F\{^1H\}$ NMR spectrum (376.50 MHz, CD_2Cl_2, 298 K) of compound $\mathbf{4^F}$.

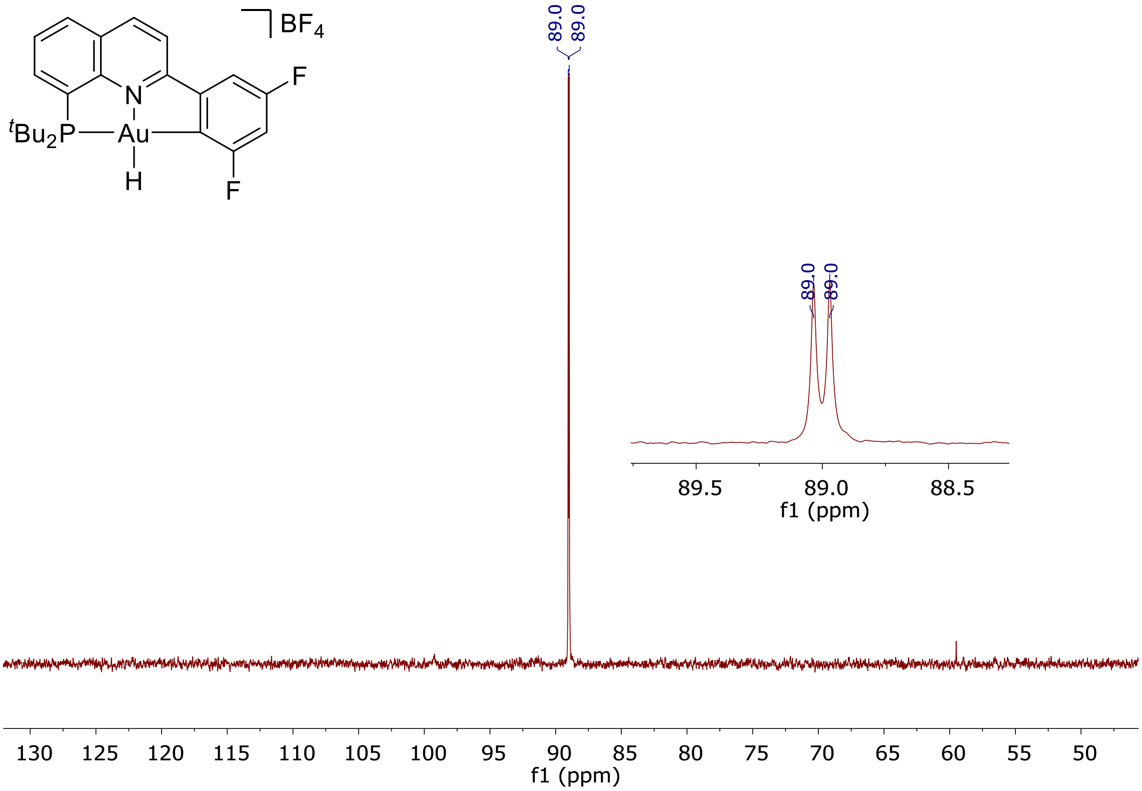

Figure S63. $^{31}P\{^{1}H\}$ NMR spectrum (202.54 MHz, CD_2Cl_2, 298 K) of compound **4^F^**.

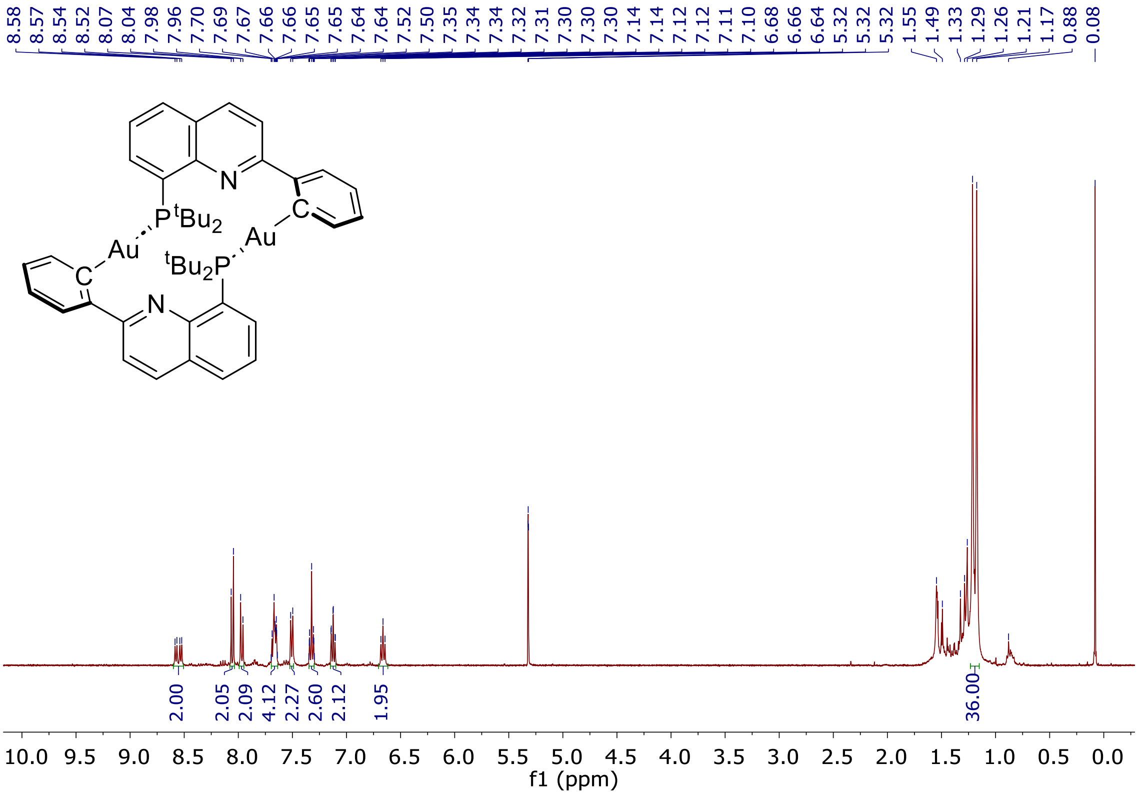

Figure S64. ^{1}H NMR spectrum (400.13 MHz, CD_2Cl_2, 298 K) of compound **5^H^** (residual pentane).

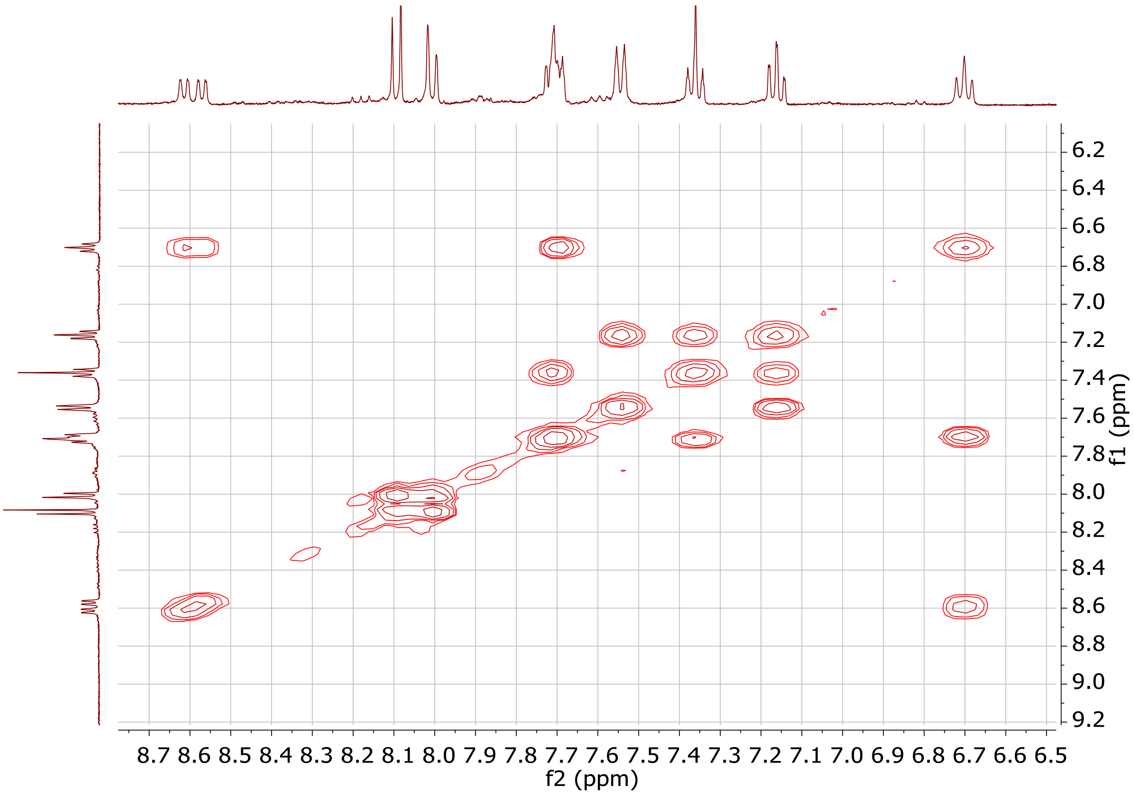

Figure S65. ^{1}H-^{1}H COSY NMR spectrum (400.13 MHz, CD_2Cl_2, 298 K) of compound **5^{H}**. Expansion of aromatic region.

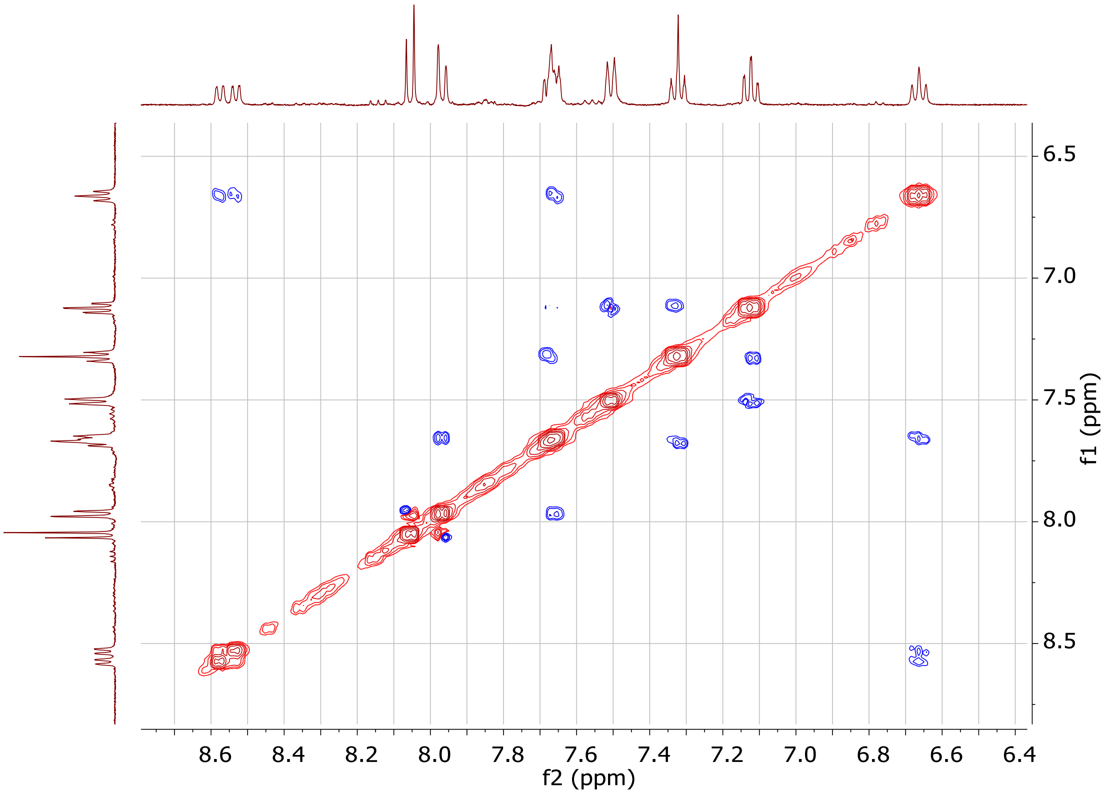

Figure S66. ^{1}H-^{1}H NOESY NMR spectrum (400.13 MHz, CD_2Cl_2, 298 K) of compound **5^{H}**. Expansion of aromatic region.

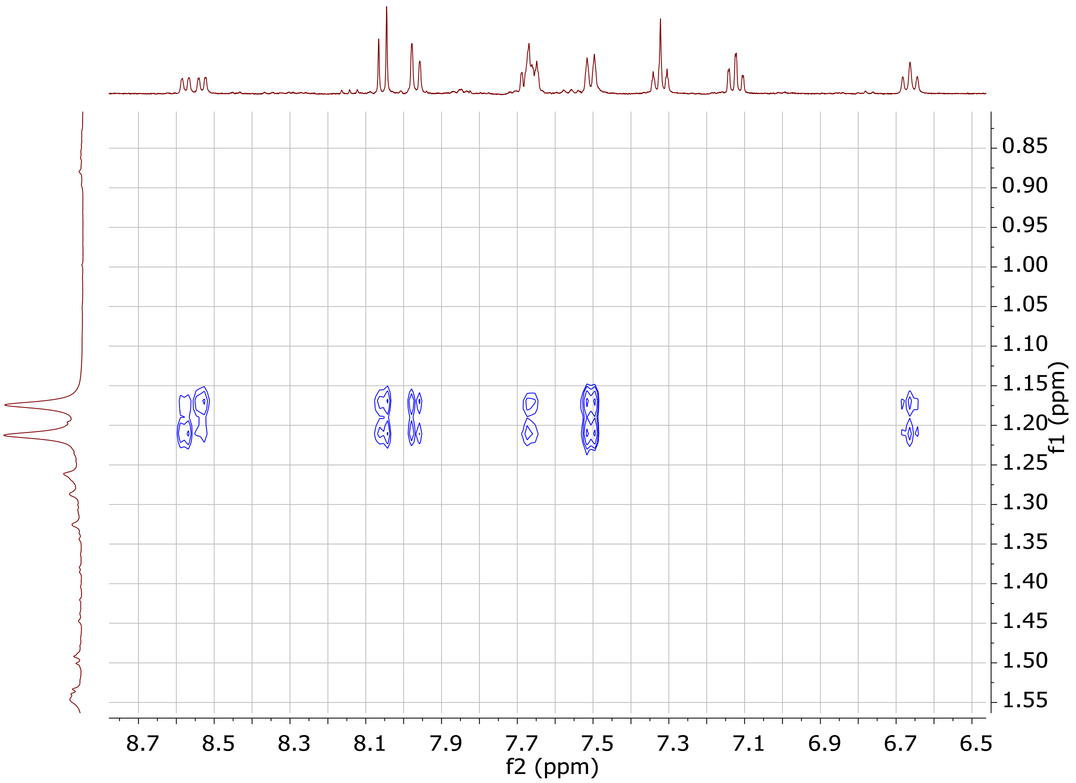

Figure S67. ^{1}H-^{1}H NOESY NMR spectrum (400.13 MHz, CD_2Cl_2, 298 K) of compound **5^H**. Expansion of *t*Bu cross peaks.

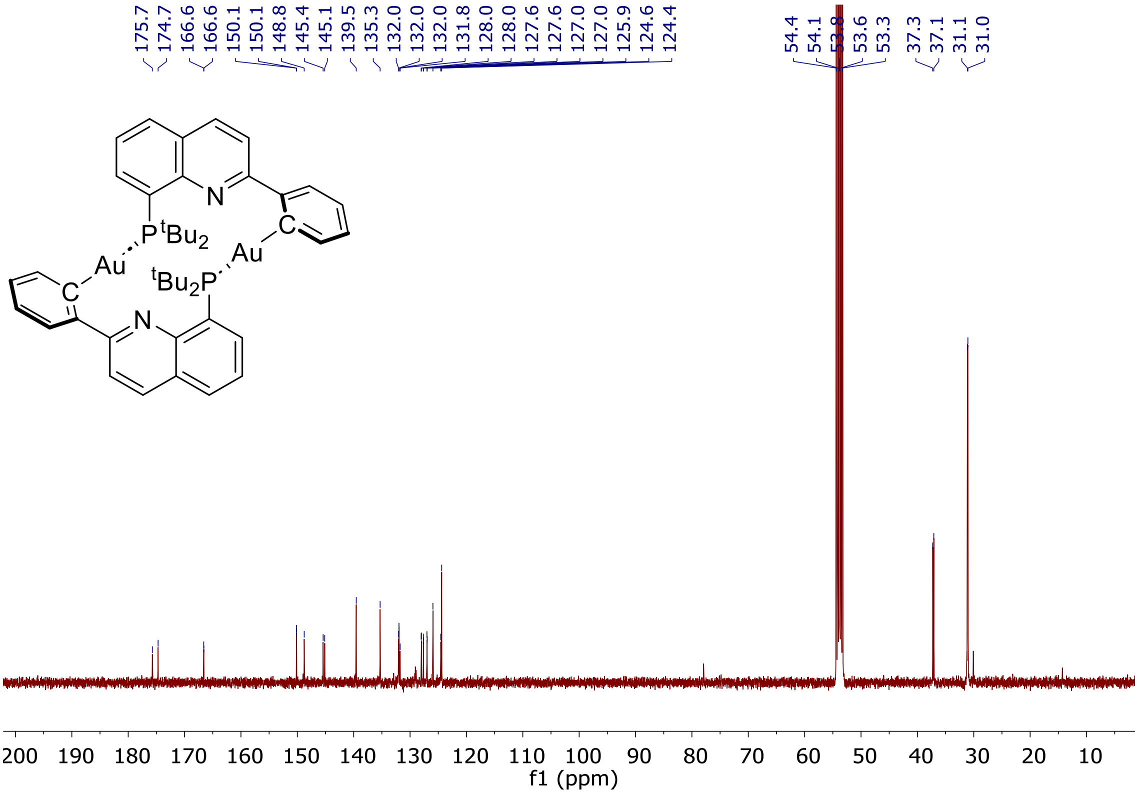

Figure S68. $^{13}C\{^{1}H\}$ NMR spectrum (100.62 MHz, CD_2Cl_2, 298 K) of compound $\mathbf{5^{H}}$.

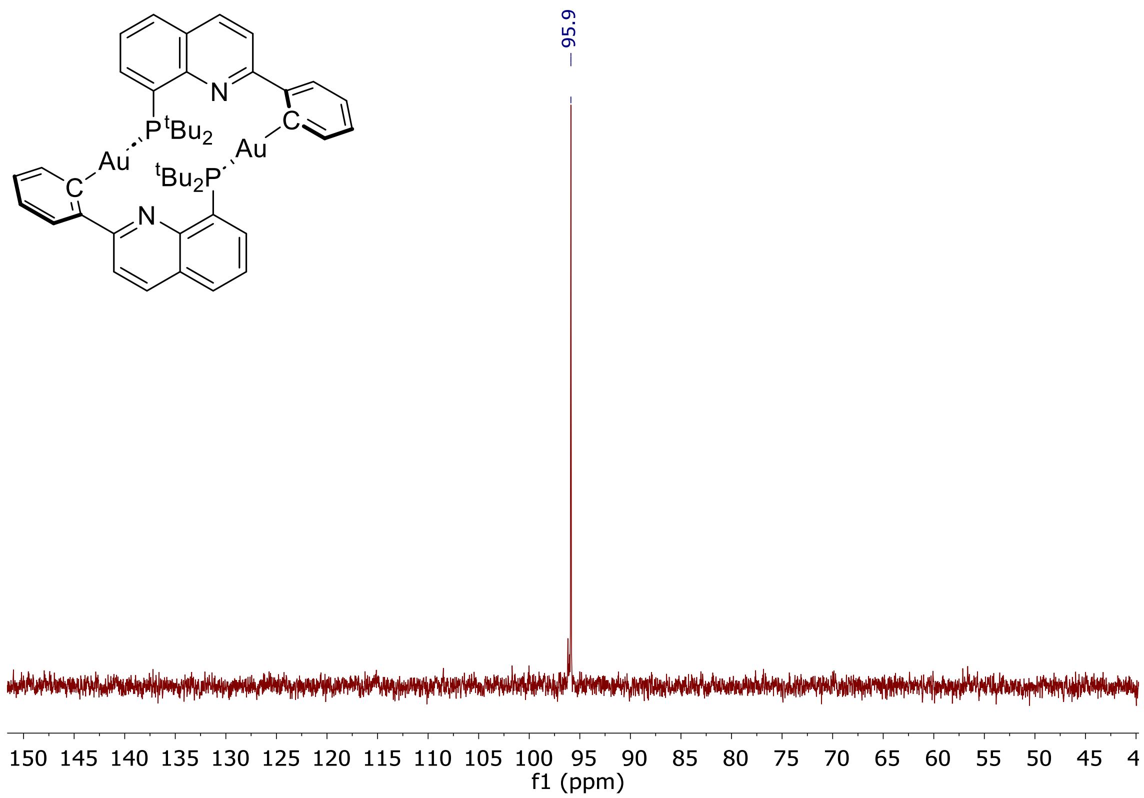

Figure S69. $^{31}P\{^1H\}$ NMR spectrum (161.99 MHz, CD_2Cl_2, 298 K) of compound **5^H**.

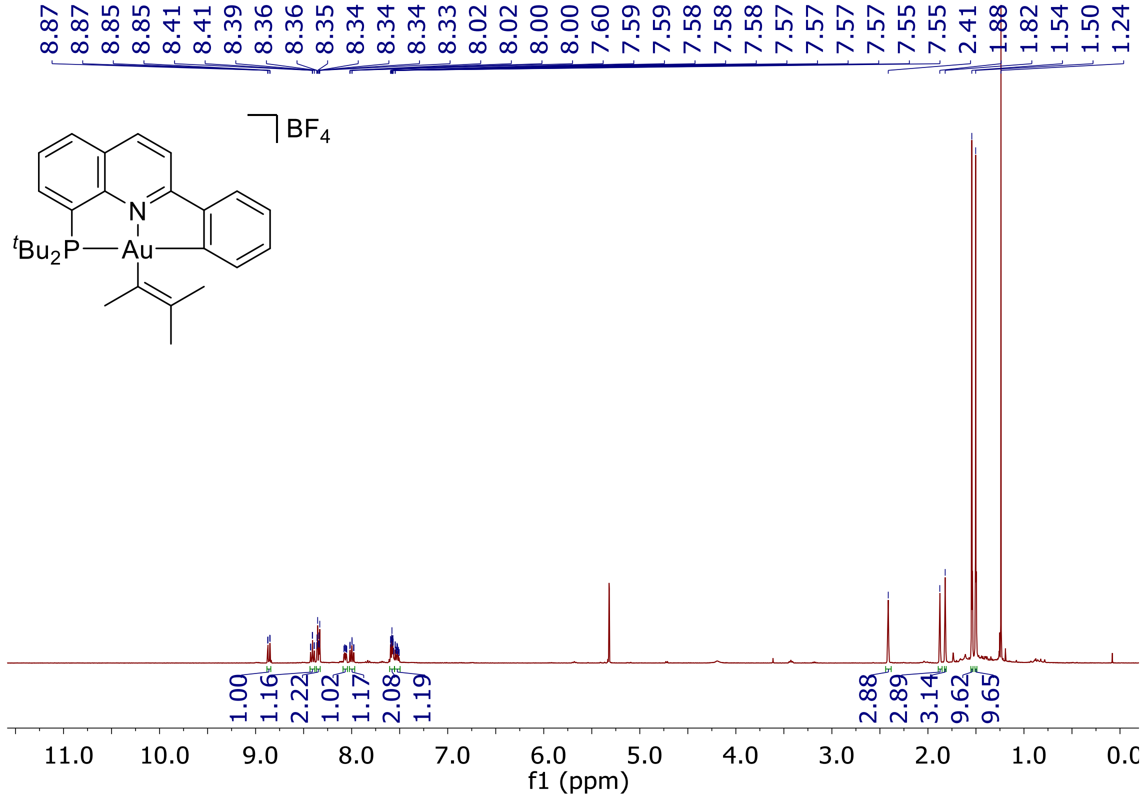

Figure S70. ^1H NMR spectrum (400.13 MHz, CD_2Cl_2, 298 K) of compound **6^H.**

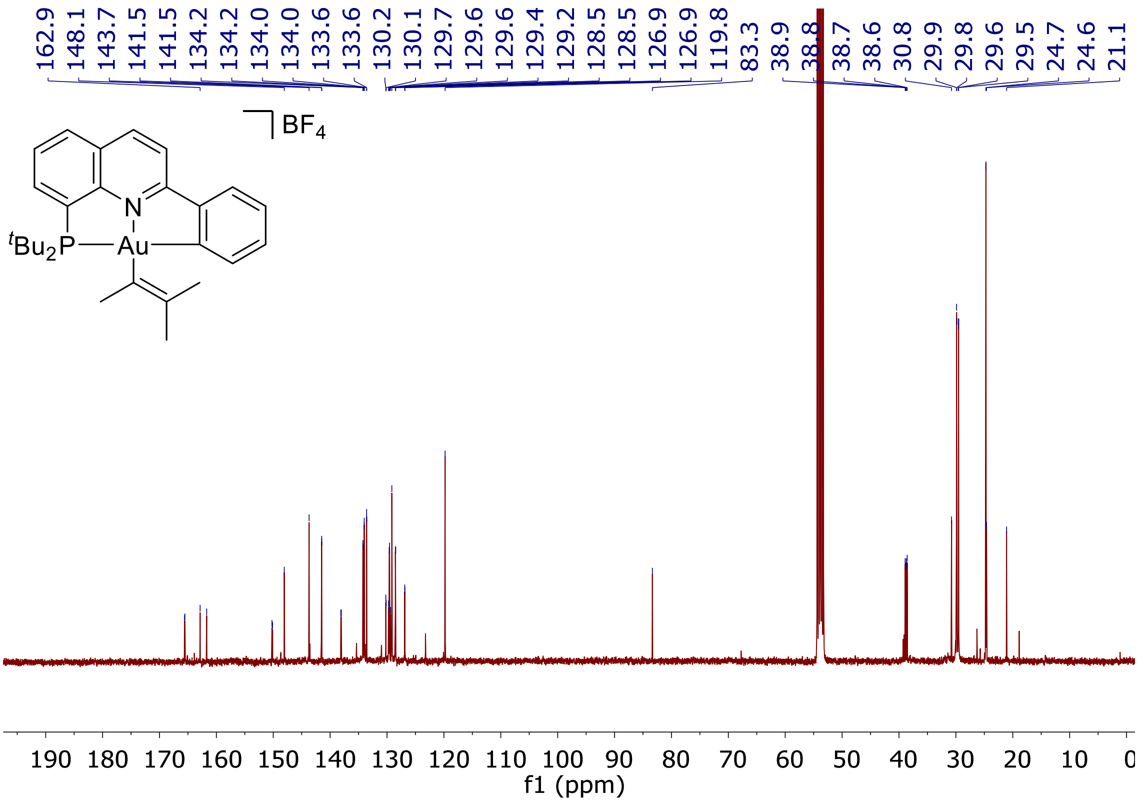

Figure S71. $^{13}C\{^{1}H\}$ NMR spectrum (100.62 MHz, CD_2Cl_2, 298 K) of compound $\mathbf{6^{H}}$.

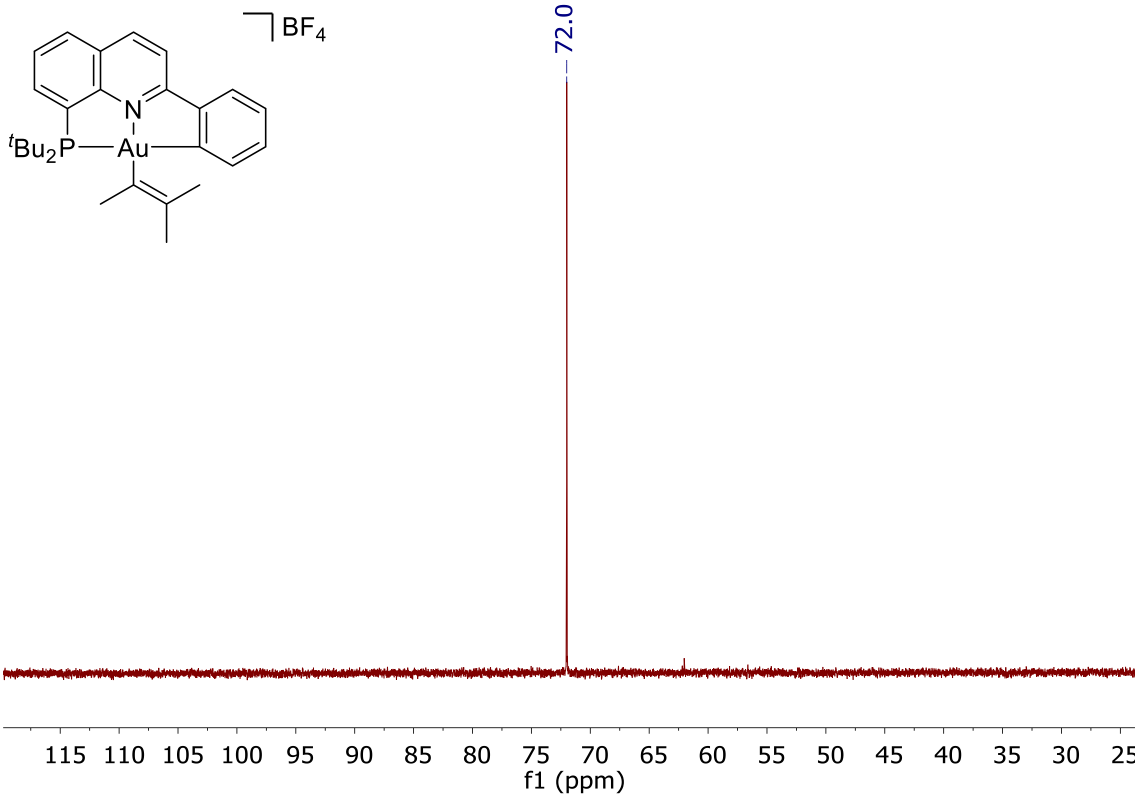

Figure S72. $^{31}P\{^{1}H\}$ NMR spectrum (161.99 MHz, CD_2Cl_2, 298 K) of compound $\mathbf{6^{H}}$.

7. X-ray diffraction analyses

All measurements were made on a *Rigaku Oxford Diffraction SuperNova* area-detector diffractometer,[4] using Cu *K*α radiation (λ = 1.54184 Å) for compound **2^H^**, and Mo *K*α radiation (λ = 0.71073 Å) for compounds **1^H^**, **3^H^**, and **4^H^** from a micro-focus X-ray source and an *Oxford Instruments Cryojet XL* cooler.

Data reduction was performed with *CrysAlisPro.*[4] The intensities were corrected for Lorentz and polarization effects, and a numerical absorption correction[5] was applied. The space group was uniquely determined by the systematic absences for compounds **1^H^**, **2^H^**, and **4^H^**. The space group was determined from packing considerations, a statistical analysis of intensity distribution, and the successful solution and refinement of the structure **3^H^**. Equivalent reflections, other than Friedel pairs (**4^H^**), were merged. The data collection and refinement parameters for all structures are given in the Tables S2-S18.

The structures were solved by dual space methods using *SHELXT-2018*,[6] which revealed the positions of all non-hydrogen atoms. The non-hydrogen atoms were refined anisotropically. All the H-atoms (unless otherwise stated) were placed in geometrically calculated positions and refined by using a riding model where each H-atom was assigned a fixed isotropic displacement parameter with a value equal to 1.2Ueq of its parent atom (1.5Ueq for the methyl groups). The refinement of the structures was carried out on F^2 by using full-matrix least-squares procedures, which minimized the function $\Sigma w(F_o^2 - F_c^2)^2$. The weighting scheme was based on counting statistics and included a factor to downweigh the intense reflections. Plots of $\Sigma w(F_o^2 - F_c^2)^2$ versus F_c/F_c (max) and resolution showed no unusual trends. A correction for secondary extinction was applied for **1^H^** and **2^H^**.

Neutral atom scattering factors for non-hydrogen atoms were taken from Maslen, Fox and O'Keefe,[7a] and the scattering factors for H-atoms were taken from Stewart, Davidson and Simpson.[8] Anomalous dispersion effects were included in F_c,[9] the values for *f'* and *f''* were those of Creagh and McAuley.[7b] The values of the mass attenuation coefficients are those of Creagh and Hubbel.[7c] The *SHELXL-2018* program[10] was used for all calculations.

(P^N^C^H)Au-Cl (1^H) (CCDC 2264146)

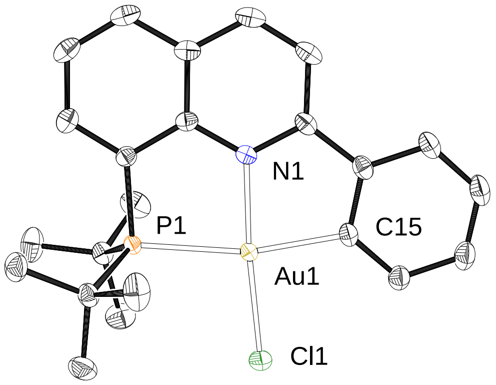

Note: The structure of $C_{23}H_{27}AuClNP^+$ BF_4^- has been solved and refined successfully with no unusual features.

The unit cell constants and an orientation matrix for data collection were obtained from a least-squares refinement of the setting angles of 25261 reflections in the range 4° < 2θ < 66°. A total of 655 frames were collected using ω scans with κ offsets, 12.0 seconds exposure time and a rotation angle of 0.8° per frame, and a crystal-detector distance of 52.0 mm.

Table S2. Crystallographic data of **1^H**.

Crystallized from		MeCN
Empirical formula		$C_{23}H_{27}AuBClF_4NP$
Formula weight [g mol^{-1}]		667.65
Crystal color, habit		colorless, prism
Crystal dimensions [mm]		0.13 x 0.17 x 0.26
Temperature [K]		160(1)
Crystal system		monoclinic
Space group		$P2_1/n$ (#14)
Z		4
Reflections for the cell determination		25261
2θ range for cell determination [º]		4-66
Unit cell parameters	*a* [Å]	13.3419(2)
	b [Å]	12.58338(18)
	c [Å]	14.7238(3)
	α [º]	90
	β [º]	110.248(2)

γ [º]	90
V [Å^3]	2319.17(7)
F(000)	1296
D_x [g·cm^3]	1.912
μ(Mo Kα) [mm^{-1}]	6.572
Scan type	ω
$2\theta_{(max)}$ [º]	65.7
Transmission factors (min; max)	0.358; 0.786
Total reflections measured	38756
Symmetry independent reflections	8037
R_{int}	0.024
Reflections with $I > 2\sigma(I)$	7359
Reflections used in refinement	8037
Parameters refined; restrains	296
Final $R(F)$ [$I > 2\sigma(I)$ reflections]	0.0155
$wR(F^2)$ (all data)	0.0377
Weights	$w = [\sigma^2(F_o^2) + (0.0163P)^2 + 1.0498P]^{-1}$ where $P = (F_o^2 + 2F_c^2)/3$
Goodness of fit	1.055
Secondary extinction coefficient	0.00034(4)
Final Δ_{max}/σ	0.003
$\Delta\rho$ (max; min) [e Å^{-3}]	0.98; -0.74
$\sigma(d_{(C-C)})$ [Å]	0.002 – 0.003

Table S3. Bond lengths (Å) of **1^{H}** with standard uncertainties in parentheses.

Au(1) -N(1)	2.0015(12)	C(9) -C(10)	1.455(2)
Au(1) -C(15)	2.0553(15)	C(10) -C(11)	1.403(2)
Au(1) -Cl(1)	2.2708(4)	C(10) -C(15)	1.410(2)
Au(1) -P(1)	2.3814(4)	C(11) -C(12)	1.379(3)
P(1) -C(1)	1.8254(16)	C(12) -C(13)	1.387(3)
P(1) -C(16)	1.8611(16)	C(13) -C(14)	1.399(2)
P(1) -C(20)	1.8655(16)	C(14) -C(15)	1.384(2)
N(1) -C(9)	1.350(2)	C(16) -C(18)	1.525(2)
N(1) -C(6)	1.375(2)	C(16) -C(17)	1.534(2)
C(1) -C(2)	1.384(2)	C(16) -C(19)	1.536(2)
C(1) -C(6)	1.418(2)	C(20) -C(23)	1.526(2)

C(2) -C(3)	1.412(2)	C(20) -C(21)	1.530(2)
C(3) -C(4)	1.361(3)	C(20) -C(22)	1.534(2)
C(4) -C(5)	1.413(2)	F(1) -B(1)	1.389(2)
C(5) -C(6)	1.415(2)	F(2) -B(1)	1.376(3)
C(5) -C(7)	1.418(2)	F(3) -B(1)	1.389(2)
C(7) -C(8)	1.356(3)	F(4) -B(1)	1.386(3)
C(8) -C(9)	1.412(2)		

Table S4. Bond angles (°) of **1^{H}** with standard uncertainties in parentheses.

N(1) -Au(1) -C(15)	81.51(6)	N(1) -C(9) -C(10)	114.73(14)
N(1) -Au(1) -Cl(1)	175.11(4)	C(8) -C(9) -C(10)	125.65(15)
C(15) -Au(1) -Cl(1)	94.10(5)	C(11) -C(10) -C(15)	119.94(16)
N(1) -Au(1) -P(1)	84.86(4)	C(11) -C(10) -C(9)	123.57(15)
C(15) -Au(1) -P(1)	166.36(5)	C(15) -C(10) -C(9)	116.48(14)
Cl(1) -Au(1) -P(1)	99.543(14)	C(12) -C(11) -C(10)	119.35(17)
C(1) -P(1) -C(16)	108.49(7)	C(11) -C(12) -C(13)	120.80(16)
C(1) -P(1) -C(20)	106.56(7)	C(12) -C(13) -C(14)	120.45(17)
C(16) -P(1) -C(20)	117.00(7)	C(15) -C(14) -C(13)	119.47(17)
C(1) -P(1) -Au(1)	96.68(5)	C(14) -C(15) -C(10)	119.98(15)
C(16) -P(1) -Au(1)	112.10(5)	C(14) -C(15) -Au(1)	128.93(13)
C(20) -P(1) -Au(1)	113.70(5)	C(10) -C(15) -Au(1)	111.09(11)
C(9) -N(1) -C(6)	122.55(14)	C(18) -C(16) -C(17)	109.81(16)
C(9) -N(1) -Au(1)	116.16(11)	C(18) -C(16) -C(19)	110.23(14)
C(6) -N(1) -Au(1)	121.27(10)	C(17) -C(16) -C(19)	108.52(15)
C(2) -C(1) -C(6)	118.12(15)	C(18) -C(16) -P(1)	109.53(12)
C(2) -C(1) -P(1)	124.87(13)	C(17) -C(16) -P(1)	104.51(11)
C(6) -C(1) -P(1)	116.96(11)	C(19) -C(16) -P(1)	114.05(11)
C(1) -C(2) -C(3)	120.94(16)	C(23) -C(20) -C(21)	110.29(16)
C(4) -C(3) -C(2)	120.99(16)	C(23) -C(20) -C(22)	109.71(16)
C(3) -C(4) -C(5)	120.24(16)	C(21) -C(20) -C(22)	108.40(16)
C(4) -C(5) -C(6)	118.71(16)	C(23) -C(20) -P(1)	112.19(12)
C(4) -C(5) -C(7)	123.02(16)	C(21) -C(20) -P(1)	106.09(12)
C(6) -C(5) -C(7)	118.28(16)	C(22) -C(20) -P(1)	110.04(12)

N(1)	-C(6)	-C(5)	118.82(14)	F(2)	-B(1)	-F(4)	108.96(17)
N(1)	-C(6)	-C(1)	120.18(14)	F(2)	-B(1)	-F(3)	109.52(17)
C(5)	-C(6)	-C(1)	120.99(15)	F(4)	-B(1)	-F(3)	109.51(18)
C(8)	-C(7)	-C(5)	121.04(15)	F(2)	-B(1)	-F(1)	110.18(19)
C(7)	-C(8)	-C(9)	119.66(16)	F(4)	-B(1)	-F(1)	109.47(16)
N(1)	-C(9)	-C(8)	119.61(15)	F(3)	-B(1)	-F(1)	109.19(17)

Table S5. Torsion angles (°) of **1^H** with standard uncertainties in parentheses.

C(16)	-P(1)	-C(1)	-C(2)	-64.5(2)	N(1)	-C(9)	-C(10)	-C(11)	179.5(2)
C(20)	-P(1)	-C(1)	-C(2)	62.3(2)	C(8)	-C(9)	-C(10)	-C(11)	0.7(3)
Au(1)	-P(1)	-C(1)	-C(2)	179.5(1)	N(1)	-C(9)	-C(10)	-C(15)	0.8(2)
C(16)	-P(1)	-C(1)	-C(6)	118.1(1)	C(8)	-C(9)	-C(10)	-C(15)	-178.0(2)
C(20)	-P(1)	-C(1)	-C(6)	-115.1(1)	C(15)	-C(10)	-C(11)	-C(12)	-0.5(3)
Au(1)	-P(1)	-C(1)	-C(6)	2.1(1)	C(9)	-C(10)	-C(11)	-C(12)	-179.2(2)
C(6)	-C(1)	-C(2)	-C(3)	0.5(2)	C(10)	-C(11)	-C(12)	-C(13)	1.5(3)
P(1)	-C(1)	-C(2)	-C(3)	-176.8(1)	C(11)	-C(12)	-C(13)	-C(14)	-1.2(3)
C(1)	-C(2)	-C(3)	-C(4)	-0.8(3)	C(12)	-C(13)	-C(14)	-C(15)	-0.2(3)
C(2)	-C(3)	-C(4)	-C(5)	0.2(3)	C(13)	-C(14)	-C(15)	-C(10)	1.2(2)
C(3)	-C(4)	-C(5)	-C(6)	0.5(3)	C(13)	-C(14)	-C(15)	-Au(1)	-179.3(1)
C(3)	-C(4)	-C(5)	-C(7)	-179.4(2)	C(11)	-C(10)	-C(15)	-C(14)	-0.8(2)
C(9)	-N(1)	-C(6)	-C(5)	2.3(2)	C(9)	-C(10)	-C(15)	-C(14)	177.9(2)
Au(1)	-N(1)	-C(6)	-C(5)	-179.3(1)	C(11)	-C(10)	-C(15)	-Au(1)	179.5(1)
C(9)	-N(1)	-C(6)	-C(1)	-178.0(1)	C(9)	-C(10)	-C(15)	-Au(1)	-1.8(2)
Au(1)	-N(1)	-C(6)	-C(1)	0.4(2)	C(1)	-P(1)	-C(16)	-C(18)	170.1(1)
C(4)	-C(5)	-C(6)	-N(1)	179.0(2)	C(20)	-P(1)	-C(16)	-C(18)	49.5(2)
C(7)	-C(5)	-C(6)	-N(1)	-1.1(2)	Au(1)	-P(1)	-C(16)	-C(18)	-84.4(1)
C(4)	-C(5)	-C(6)	-C(1)	-0.8(2)	C(1)	-P(1)	-C(16)	-C(17)	-72.4(1)
C(7)	-C(5)	-C(6)	-C(1)	179.1(2)	C(20)	-P(1)	-C(16)	-C(17)	167.1(1)
C(2)	-C(1)	-C(6)	-N(1)	-179.5(1)	Au(1)	-P(1)	-C(16)	-C(17)	33.2(1)
P(1)	-C(1)	-C(6)	-N(1)	-2.0(2)	C(1)	-P(1)	-C(16)	-C(19)	46.0(1)
C(2)	-C(1)	-C(6)	-C(5)	0.3(2)	C(20)	-P(1)	-C(16)	-C(19)	-74.5(2)
P(1)	-C(1)	-C(6)	-C(5)	177.8(1)	Au(1)	-P(1)	-C(16)	-C(19)	151.6(1)
C(4)	-C(5)	-C(7)	-C(8)	179.7(2)	C(1)	-P(1)	-C(20)	-C(23)	-71.3(2)

C(6)	-C(5)	-C(7)	-C(8)	-0.2(3)	C(16)	-P(1)	-C(20)	-C(23)	50.2(2)
C(5)	-C(7)	-C(8)	-C(9)	0.5(3)	Au(1)	-P(1)	-C(20)	-C(23)	-176.6(1)
C(6)	-N(1)	-C(9)	-C(8)	-2.0(2)	C(1)	-P(1)	-C(20)	-C(21)	49.2(1)
Au(1)	-N(1)	-C(9)	-C(8)	179.5(1)	C(16)	-P(1)	-C(20)	-C(21)	170.7(1)
C(6)	-N(1)	-C(9)	-C(10)	179.1(1)	Au(1)	-P(1)	-C(20)	-C(21)	-56.1(1)
Au(1)	-N(1)	-C(9)	-C(10)	0.6(2)	C(1)	-P(1)	-C(20)	-C(22)	166.2(1)
C(7)	-C(8)	-C(9)	-N(1)	0.6(2)	C(16)	-P(1)	-C(20)	-C(22)	-72.3(2)
C(7)	-C(8)	-C(9)	-C(10)	179.3(2)	Au(1)	-P(1)	-C(20)	-C(22)	61.0(1)

(P^N^C^H)Au-OH (2^H) (CCDC 2264148)

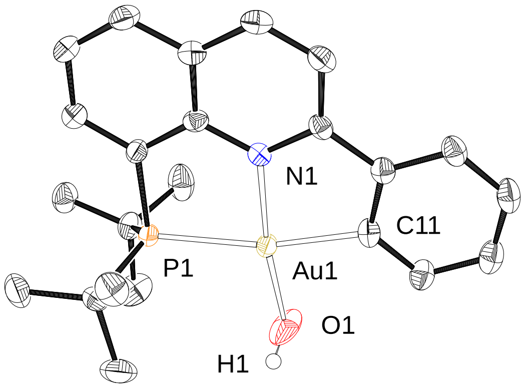

Note: The structure of $C_{23}H_{28}AuNOP^+$ BF_4^- CH_2Cl_2 has been solved and refined successfully. The asymmetric unit contains one Au-complex cation, one anion and one molecule of CH_2Cl_2. The hydroxy H-atom was placed initially in the position indicated by a difference electron density map, although the Au–O–H geometry was not ideal with the O–H distance being too short and the Au–O–H angle too small. The position of the hydroxy H-atom was allowed to refine together with an isotropic displacement parameter, while restraining the O–H and Au···H distances to 0.84(1) and 2.40(1) Å, respectively.

The unit cell constants and an orientation matrix for data collection were obtained from a least-squares refinement of the setting angles of 20914 reflections in the range 8° < 2θ < 153°. A total of 1762 frames were collected using ω scans with κ offsets, 1.0-4.0 seconds exposure time and a rotation angle of 1.0° per frame, and a crystal-detector distance of 52.0 mm.

Table S6. Crystallographic data of **2^H**.

Crystallized from	CH_2Cl_2 / *n*-hexane
Empirical formula	$C_{24}H_{30}AuBCl_2F_4NOP$
Formula weight [g mol^{-1}]	734.13
Crystal color, habit	pale-yellow, plate
Crystal dimensions [mm]	0.04 x 0.30 x 0.31
Temperature [K]	160(1)
Crystal system	Monoclinic
Space group	$P2_1/n$ (#14)
Z	4
Reflections for the cell determination	20914
2θ range for cell determination [º]	8 – 153

Unit cell parameters	a [Å]	12.84219(11)
	b [Å]	13.43843(10)
	c [Å]	15.21397(13)
	α [º]	90
	β [º]	92.0950(7)
	γ [º]	90
	V [Å^3]	2623.85(4)
F(000)		1432
D_x [g·cm^3]		1.858
μ(Cu Kα) [mm^{-1}]		13.407
Scan type		ω
$2\theta_{(max)}$ [º]		152.7
Transmission factors (min; max)		0.032; 1.000
Total reflections measured		27087
Symmetry independent reflections		5470
R_{int}		0.024
Reflections with $I > 2\sigma(I)$		5278
Reflections used in refinement		5470
Parameters refined; restrains		327; 2
Final $R(F)$ [$I > 2\sigma(I)$ reflections]		0.0210
$wR(F^2)$ (all data)		0.0530
Weights	$w = [\sigma^2(F_o^2) + (0.0241P)^2 + 4.8373P]^{-1}$ where $P = (F_o^2 + 2F_c^2)/3$	
Goodness of fit		1.044
Secondary extinction coefficient		0.00031(2)
Final Δ_{max}/σ		0.002
$\Delta\rho$ (max; min) [e Å^{-3}]		1.19; -0.89
$\sigma(d_{(C-C)})$ [Å]		0.003 – 0.005

Table S7. Bond lengths (Å) of **2^{H}** with standard uncertainties in parentheses.

Au(1) -O(1)	1.971(2)	C(10) -C(15)	1.397(4)
Au(1) -N(1)	2.008(2)	C(10) -C(11)	1.406(4)
Au(1) -C(11)	2.046(3)	C(11) -C(12)	1.376(4)
Au(1) -P(1)	2.3728(6)	C(12) -C(13)	1.394(4)
P(1) -C(1)	1.840(3)	C(13) -C(14)	1.384(5)
P(1) -C(20)	1.861(3)	C(14) -C(15)	1.385(4)

P(1) -C(16)	1.869(3)	C(16) -C(19)	1.519(4)
N(1) -C(9)	1.348(3)	C(16) -C(18)	1.530(4)
N(1) -C(6)	1.375(3)	C(16) -C(17)	1.536(4)
C(1) -C(2)	1.384(4)	C(20) -C(21)	1.529(4)
C(1) -C(6)	1.403(4)	C(20) -C(23)	1.534(4)
C(2) -C(3)	1.410(4)	C(20) -C(22)	1.542(4)
C(3) -C(4)	1.371(4)	F(1) -B(1)	1.378(5)
C(4) -C(5)	1.409(4)	F(2) -B(1)	1.386(5)
C(5) -C(7)	1.412(4)	F(3) -B(1)	1.367(4)
C(5) -C(6)	1.417(3)	F(4) -B(1)	1.378(5)
C(7) -C(8)	1.372(4)	Cl(1) -C(24)	1.749(4)
C(8) -C(9)	1.403(4)	Cl(2) -C(24)	1.760(4)
C(9) -C(10)	1.465(4)		

Table S8. Bond angles (°) of $\mathbf{2^{H}}$ with standard uncertainties in parentheses.

O(1) -Au(1) -N(1)	172.64(11)	C(8) -C(9) -C(10)	125.6(2)
O(1) -Au(1) -C(11)	91.28(11)	C(15) -C(10) -C(11)	119.8(2)
N(1) -Au(1) -C(11)	81.69(10)	C(15) -C(10) -C(9)	123.7(2)
O(1) -Au(1) -P(1)	102.22(8)	C(11) -C(10) -C(9)	116.5(2)
N(1) -Au(1) -P(1)	84.79(6)	C(12) -C(11) -C(10)	120.5(2)
C(11) -Au(1) -P(1)	166.48(8)	C(12) -C(11) -Au(1)	128.1(2)
C(1) -P(1) -C(20)	108.44(13)	C(10) -C(11) -Au(1)	111.37(19)
C(1) -P(1) -C(16)	106.37(12)	C(11) -C(12) -C(13)	119.2(3)
C(20) -P(1) -C(16)	117.08(13)	C(14) -C(13) -C(12)	120.8(3)
C(1) -P(1) -Au(1)	96.68(8)	C(13) -C(14) -C(15)	120.5(3)
C(20) -P(1) -Au(1)	113.27(9)	C(14) -C(15) -C(10)	119.2(3)
C(16) -P(1) -Au(1)	112.64(9)	C(19) -C(16) -C(18)	110.4(3)
C(9) -N(1) -C(6)	122.9(2)	C(19) -C(16) -C(17)	109.5(3)
C(9) -N(1) -Au(1)	115.90(18)	C(18) -C(16) -C(17)	108.2(3)
C(6) -N(1) -Au(1)	121.17(17)	C(19) -C(16) -P(1)	114.0(2)
C(2) -C(1) -C(6)	118.4(2)	C(18) -C(16) -P(1)	108.9(2)
C(2) -C(1) -P(1)	124.7(2)	C(17) -C(16) -P(1)	105.51(19)
C(6) -C(1) -P(1)	116.83(18)	C(21) -C(20) -C(23)	108.9(3)

C(1) -C(2) -C(3)	121.3(3)		C(21) -C(20) -C(22)	109.5(3)
C(4) -C(3) -C(2)	119.9(3)		C(23) -C(20) -C(22)	110.5(3)
C(3) -C(4) -C(5)	120.9(3)		C(21) -C(20) -P(1)	113.9(2)
C(4) -C(5) -C(7)	123.1(3)		C(23) -C(20) -P(1)	108.9(2)
C(4) -C(5) -C(6)	118.3(3)		C(22) -C(20) -P(1)	105.0(2)
C(7) -C(5) -C(6)	118.7(2)		F(3) -B(1) -F(4)	108.2(3)
N(1) -C(6) -C(1)	120.4(2)		F(3) -B(1) -F(1)	110.0(4)
N(1) -C(6) -C(5)	118.3(2)		F(4) -B(1) -F(1)	110.4(3)
C(1) -C(6) -C(5)	121.2(2)		F(3) -B(1) -F(2)	107.7(3)
C(8) -C(7) -C(5)	120.7(3)		F(4) -B(1) -F(2)	110.8(4)
C(7) -C(8) -C(9)	119.5(3)		F(1) -B(1) -F(2)	109.7(3)
N(1) -C(9) -C(8)	119.8(2)		Cl(1) -C(24) -Cl(2)	112.5(2)
N(1) -C(9) -C(10)	114.5(2)			

Table S9. Torsion angles (°) of **2**$^{\mathbf{H}}$ with standard uncertainties in parentheses.

C(20) -P(1) -C(1) -C(2)	62.4(3)	N(1) -C(9) -C(10) -C(15)	178.0(2)
C(16) -P(1) -C(1) -C(2)	-64.3(3)	C(8) -C(9) -C(10) -C(15)	-2.1(4)
Au(1) -P(1) -C(1) -C(2)	179.7(2)	N(1) -C(9) -C(10) -C(11)	-1.3(3)
C(20) -P(1) -C(1) -C(6)	-120.3(2)	C(8) -C(9) -C(10) -C(11)	178.7(3)
C(16) -P(1) -C(1) -C(6)	113.0(2)	C(15) -C(10) -C(11) -C(12)	0.6(4)
Au(1) -P(1) -C(1) -C(6)	-3.0(2)	C(9) -C(10) -C(11) -C(12)	179.9(2)
C(6) -C(1) -C(2) -C(3)	-1.3(4)	C(15) -C(10) -C(11) -Au(1)	-179.5(2)
P(1) -C(1) -C(2) -C(3)	175.9(2)	C(9) -C(10) -C(11) -Au(1)	-0.2(3)
C(1) -C(2) -C(3) -C(4)	-0.4(5)	C(10) -C(11) -C(12) -C(13)	-0.7(4)
C(2) -C(3) -C(4) -C(5)	1.7(5)	Au(1) -C(11) -C(12) -C(13)	179.4(2)
C(3) -C(4) -C(5) -C(7)	179.3(3)	C(11) -C(12) -C(13) -C(14)	0.1(4)
C(3) -C(4) -C(5) -C(6)	-1.2(4)	C(12) -C(13) -C(14) -C(15)	0.7(4)
C(9) -N(1) -C(6) -C(1)	179.6(2)	C(13) -C(14) -C(15) -C(10)	-0.8(4)
Au(1) -N(1) -C(6) -C(1)	-1.0(3)	C(11) -C(10) -C(15) -C(14)	0.2(4)
C(9) -N(1) -C(6) -C(5)	-1.7(4)	C(9) -C(10) -C(15) -C(14)	-179.1(2)
Au(1) -N(1) -C(6) -C(5)	177.7(2)	C(1) -P(1) -C(16) -C(19)	71.9(2)
C(2) -C(1) -C(6) -N(1)	-179.6(2)	C(20) -P(1) -C(16) -C(19)	-49.4(3)
P(1) -C(1) -C(6) -N(1)	3.0(3)	Au(1) -P(1) -C(16) -C(19)	176.6(2)

C(2)	-C(1)	-C(6)	-C(5)	1.8(4)	C(1)	-P(1)	-C(16)	-C(18)	-164.2(2)
P(1)	-C(1)	-C(6)	-C(5)	-175.7(2)	C(20)	-P(1)	-C(16)	-C(18)	74.4(2)
C(4)	-C(5)	-C(6)	-N(1)	-179.2(2)	Au(1)	-P(1)	-C(16)	-C(18)	-59.5(2)
C(7)	-C(5)	-C(6)	-N(1)	0.3(4)	C(1)	-P(1)	-C(16)	-C(17)	-48.3(2)
C(4)	-C(5)	-C(6)	-C(1)	-0.6(4)	C(20)	-P(1)	-C(16)	-C(17)	-169.6(2)
C(7)	-C(5)	-C(6)	-C(1)	179.0(2)	Au(1)	-P(1)	-C(16)	-C(17)	56.4(2)
C(4)	-C(5)	-C(7)	-C(8)	-179.4(3)	C(1)	-P(1)	-C(20)	-C(21)	-53.9(2)
C(6)	-C(5)	-C(7)	-C(8)	1.0(4)	C(16)	-P(1)	-C(20)	-C(21)	66.3(3)
C(5)	-C(7)	-C(8)	-C(9)	-1.1(4)	Au(1)	-P(1)	-C(20)	-C(21)	-160.0(2)
C(6)	-N(1)	-C(9)	-C(8)	1.7(4)	C(1)	-P(1)	-C(20)	-C(23)	-175.7(2)
Au(1)	-N(1)	-C(9)	-C(8)	-177.8(2)	C(16)	-P(1)	-C(20)	-C(23)	-55.5(3)
C(6)	-N(1)	-C(9)	-C(10)	-178.4(2)	Au(1)	-P(1)	-C(20)	-C(23)	78.2(2)
Au(1)	-N(1)	-C(9)	-C(10)	2.2(3)	C(1)	-P(1)	-C(20)	-C(22)	65.8(2)
C(7)	-C(8)	-C(9)	-N(1)	-0.2(4)	C(16)	-P(1)	-C(20)	-C(22)	-173.9(2)
C(7)	-C(8)	-C(9)	-C(10)	179.8(3)	Au(1)	-P(1)	-C(20)	-C(22)	-40.2(2)

(P^N^C^H)Au-OCOH (3^H) (CCDC 2304095)

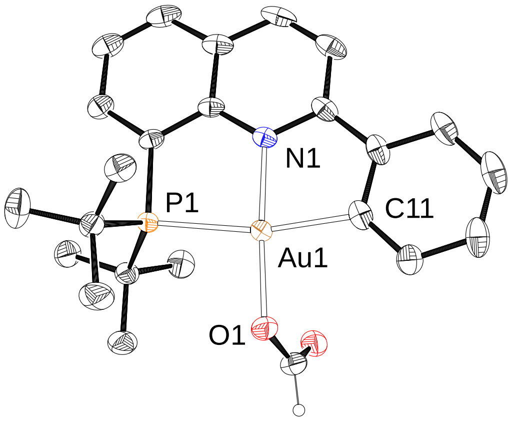

Note: The structure of $C_{24}H_{28}AuNO_2P^+$ $C_{32}H_{12}BF_{24}^-$ has been solved and refined successfully. The asymmetric unit contains two Au-complex cations and two disordered anions. The atomic coordinates of the two molecules were tested carefully for a relationship from a higher symmetry space group using the program *PLATON*,[11] but none could be found. The disorder, which is mainly in the –CF_3 groups plus one aryl ring of the anions, could only be modelled approximately. Two sets of positions were defined for three –CF_3 groups in one anion and for all –CF_3 groups and one of the parent phenyl rings in the other anion. The site occupation factors of the disordered groups were refined groupwise while constraining the sum of alternate conformations to 1.0; the occupancies of the major conformations ranged from 0.50 to 0.75. Similarity restraints were applied to the chemically equivalent bond lengths involving all disordered atoms, as well as to the F···F distances. One conformation of the disordered aryl ring was constrained to be a perfect hexagon. Neighbouring atoms within and between each of the disordered conformations, plus all F-atoms whether they were modelled as disordered or not, were restrained to have similar atomic displacement parameters. Pseudo-isotropic restraints were also applied to the atomic displacement parameters of some of the disordered atoms.

The unit cell constants and an orientation matrix for data collection were obtained from a least-squares refinement of the setting angles of 33418 reflections in the range 4° < 2θ < 54°. A total of 1434 frames were collected using ω scans with κ offsets, 25.0 seconds exposure time and a rotation angle of 0.5° per frame, and a crystal-detector distance of 55.0 mm.

Table S10. Crystallographic data of **3**^{H}.

Crystallized from	CH_2Cl_2 / *n*-pentane
Empirical formula	$C_{56}H_{40}AuBF_{24}NO_2P$
Formula weight [g mol^{-1}]	1453.63
Crystal color, habit	colorless, plate
Crystal dimensions [mm]	0.01 × 0.12 × 0.28
Temperature [K]	160(1)
Crystal system	triclinic
Space group	$P\bar{1}$ (#2)
Z	4
Reflections for the cell determination	33418
2θ range for cell determination [º]	4–54
Unit cell parameters *a* [Å]	17.5784(4)
b [Å]	19.2418(4)
c [Å]	20.3781(3)
α [º]	69.8118(17)
β [º]	89.7032(16)
γ [º]	63.002(2)
V [Å^3]	5668.9(2)
F(000)	2856
D_x [g·cm^3]	1.703
μ(Mo Kα) [mm^{-1}]	2.745
Scan type	*ω*
$2\theta_{(max)}$ [º]	56.9
Transmission factors (min; max)	0.401; 1.000
Total reflections measured	109744
Symmetry independent reflections	25204
R_{int}	0.047
Reflections with $I > 2\sigma(I)$	18705
Reflections used in refinement	25204
Parameters refined; restrains	1919; 5338
Final $R(F)$ [$I > 2\sigma(I)$ reflections]	0.0509
$wR(F^2)$ (all data)	0.1392

Weights	$w = [\sigma^2(F_o^2) + (0.0685P)^2 + 13.11P]^{-1}$ where $P = (F_o^2 + 2F_c^2)/3$
Goodness of fit	1.035
Final Δ_{max}/σ	0.001
$\Delta\rho$ (max; min) [e Å^{-3}]	2.03; -1.42
$\sigma(d_{(C-C)})$ [Å]	0.004 – 0.013

Table S11. Bond lengths (Å) of **3^H^** with standard uncertainties in parentheses.

Au(1) -N(1)	1.983(4)	C(118)-C(119)	1.398(8)
Au(1) -O(1)	2.004(4)	C(119)-C(120)	1.371(9)
Au(1) -C(11)	2.039(6)	C(119)-C(123)	1.501(9)
Au(1) -P(1)	2.3722(14)	C(120)-C(121)	1.377(9)
P(1) -C(1)	1.818(6)	C(121)-C(122)	1.400(8)
P(1) -C(17)	1.854(6)	C(121)-C(124)	1.464(10)
P(1) -C(21)	1.869(6)	C(125)-C(126)	1.394(8)
O(1) -C(16)	1.295(8)	C(125)-C(130)	1.399(8)
O(2) -C(16)	1.223(8)	C(125)-B(2)	1.651(9)
N(1) -C(9)	1.358(7)	C(126)-C(127)	1.396(8)
N(1) -C(6)	1.374(7)	C(127)-C(128)	1.368(9)
C(1) -C(2)	1.393(8)	C(127)-C(131)	1.481(9)
C(1) -C(6)	1.416(8)	C(128)-C(129)	1.397(9)
C(2) -C(3)	1.403(9)	C(129)-C(130)	1.369(9)
C(3) -C(4)	1.355(10)	C(129)-C(132)	1.429(9)
C(4) -C(5)	1.415(10)	C(132)-F(54)	1.340(4)
C(5) -C(7)	1.418(10)	C(132)-F(52)	1.345(4)
C(5) -C(6)	1.422(8)	C(132)-F(52a)	1.348(4)
C(7) -C(8)	1.354(10)	C(132)-F(53a)	1.353(4)
C(8) -C(9)	1.405(9)	C(132)-F(53)	1.354(4)

C(9) -C(10)	1.447(9)	C(132)-F(54a)	1.358(4)
C(10) -C(15)	1.395(8)	C(61) -C(62)	1.400(8)
C(10) -C(11)	1.412(9)	C(61) -C(66)	1.401(8)
C(11) -C(12)	1.388(9)	C(61) -B(1)	1.648(9)
C(12) -C(13)	1.389(9)	C(62) -C(63)	1.385(9)
C(13) -C(14)	1.362(11)	C(63) -C(64)	1.381(9)
C(14) -C(15)	1.390(11)	C(63) -C(67)	1.466(7)
C(17) -C(18)	1.534(9)	C(64) -C(65)	1.393(8)
C(17) -C(19)	1.535(9)	C(65) -C(66)	1.379(8)
C(17) -C(20)	1.538(9)	C(65) -C(68)	1.478(7)
C(21) -C(22)	1.507(9)	C(67) -F(2)	1.329(4)
C(21) -C(24)	1.536(8)	C(67) -F(3a)	1.330(4)
C(21) -C(23)	1.542(9)	C(67) -F(1)	1.336(4)
Au(2) -N(2)	1.986(4)	C(67) -F(2a)	1.342(4)
Au(2) -O(3)	2.019(4)	C(67) -F(1a)	1.373(4)
Au(2) -C(41)	2.049(5)	C(67) -F(3)	1.377(4)
Au(2) -P(2)	2.3736(14)	C(68) -F(5)	1.339(4)
P(2) -C(31)	1.828(6)	C(68) -F(6a)	1.340(4)
P(2) -C(51)	1.862(6)	C(68) -F(4a)	1.346(4)
P(2) -C(47)	1.866(6)	C(68) -F(4)	1.351(4)
O(3) -C(46)	1.293(8)	C(68) -F(6)	1.357(4)
O(4) -C(46)	1.210(8)	C(68) -F(5a)	1.360(4)
N(2) -C(39)	1.354(7)	C(69) -C(70)	1.388(4)
N(2) -C(36)	1.379(7)	C(69) -C(70a)	1.390
C(31) -C(32)	1.379(8)	C(69) -C(74a)	1.390
C(31) -C(36)	1.417(8)	C(69) -C(74)	1.392(4)

C(32) -C(33)	1.409(9)	C(69) -B(1)	1.671(9)
C(33) -C(34)	1.357(10)	C(70a)-C(71a)	1.390
C(34) -C(35)	1.409(8)	C(71a)-C(72a)	1.390
C(35) -C(36)	1.413(8)	C(71a)-C(75)	1.552(13)
C(35) -C(37)	1.419(9)	C(72a)-C(73a)	1.390
C(37) -C(38)	1.363(9)	C(73a)-C(74a)	1.390
C(38) -C(39)	1.397(8)	C(73a)-C(76a)	1.451(9)
C(39) -C(40)	1.466(8)	C(77) -C(78)	1.390(8)
C(40) -C(41)	1.401(8)	C(77) -C(82)	1.406(9)
C(40) -C(45)	1.401(8)	C(77) -B(1)	1.650(10)
C(41) -C(42)	1.380(8)	C(78) -C(79)	1.381(9)
C(42) -C(43)	1.386(9)	C(79) -C(80)	1.380(10)
C(43) -C(44)	1.367(10)	C(79) -C(83)	1.475(8)
C(44) -C(45)	1.391(9)	C(80) -C(81)	1.390(11)
C(47) -C(50)	1.518(9)	C(81) -C(82)	1.389(11)
C(47) -C(48)	1.519(10)	C(81) -C(84)	1.420(10)
C(47) -C(49)	1.536(10)	C(83) -F(14)	1.343(4)
C(51) -C(54)	1.513(9)	C(83) -F(15a)	1.345(4)
C(51) -C(52)	1.523(8)	C(83) -F(13a)	1.346(4)
C(51) -C(53)	1.543(9)	C(83) -F(15)	1.348(4)
F(31) -C(107)	1.321(10)	C(83) -F(13)	1.349(4)
F(32) -C(107)	1.326(10)	C(83) -F(14a)	1.352(4)
F(33) -C(107)	1.311(9)	C(84) -F(18a)	1.345(4)
F(34) -C(108)	1.298(8)	C(84) -F(16)	1.346(4)
F(35) -C(108)	1.318(9)	C(84) -F(17a)	1.347(4)
F(36) -C(108)	1.324(8)	C(84) -F(17)	1.348(4)

F(43) -C(123)	1.273(9)	C(84) -F(18)	1.353(4)
F(44) -C(123)	1.382(9)	C(84) -F(16a)	1.354(4)
F(45) -C(123)	1.290(8)	C(85) -C(86)	1.393(8)
F(46) -C(124)	1.309(10)	C(85) -C(90)	1.394(8)
F(47) -C(124)	1.342(10)	C(85) -B(1)	1.642(9)
F(48) -C(124)	1.347(9)	C(86) -C(87)	1.392(9)
F(49) -C(131)	1.336(10)	C(87) -C(88)	1.361(9)
F(50) -C(131)	1.341(10)	C(87) -C(91)	1.460(8)
F(51) -C(131)	1.275(9)	C(88) -C(89)	1.381(9)
C(101)-C(102)	1.395(7)	C(89) -C(90)	1.391(8)
C(101)-C(106)	1.400(7)	C(89) -C(92)	1.469(7)
C(101)-B(2)	1.643(8)	C(91) -F(20a)	1.336(4)
C(102)-C(103)	1.403(8)	C(91) -F(21)	1.337(4)
C(103)-C(104)	1.365(9)	C(91) -F(19a)	1.350(4)
C(103)-C(107)	1.464(9)	C(91) -F(19)	1.355(4)
C(104)-C(105)	1.379(8)	C(91) -F(21a)	1.357(4)
C(105)-C(106)	1.391(7)	C(91) -F(20)	1.358(4)
C(105)-C(108)	1.497(9)	C(92) -F(24)	1.343(4)
C(109)-C(110)	1.387(8)	C(92) -F(23)	1.343(4)
C(109)-C(114)	1.400(7)	C(92) -F(22a)	1.344(4)
C(109)-B(2)	1.641(8)	C(92) -F(24a)	1.350(4)
C(110)-C(111)	1.389(9)	C(92) -F(22)	1.353(4)
C(111)-C(112)	1.372(8)	C(92) -F(23a)	1.357(4)
C(111)-C(115)	1.441(8)	F(7a) -C(75a)	1.349(4)
C(112)-C(113)	1.387(8)	F(8a) -C(75a)	1.349(4)
C(113)-C(114)	1.381(8)	F(9a) -C(75a)	1.351(4)

C(113)-C(116)	1.466(7)	F(10) -C(76)	1.346(4)
C(115)-F(38)	1.340(4)	F(11) -C(76)	1.354(4)
C(115)-F(37a)	1.345(4)	F(12) -C(76)	1.351(4)
C(115)-F(38a)	1.345(4)	C(70) -C(71)	1.389(4)
C(115)-F(39)	1.348(4)	C(71) -C(72)	1.392(4)
C(115)-F(39a)	1.356(4)	C(71) -C(75a)	1.429(13)
C(115)-F(37)	1.357(4)	C(72) -C(73)	1.391(4)
C(116)-F(42a)	1.330(4)	C(73) -C(74)	1.392(4)
C(116)-F(41)	1.334(4)	C(73) -C(76)	1.454(9)
C(116)-F(40a)	1.340(4)	F(7) -C(75)	1.349(4)
C(116)-F(42)	1.360(4)	F(8) -C(75)	1.346(4)
C(116)-F(40)	1.367(4)	F(9) -C(75)	1.347(4)
C(116)-F(41a)	1.374(4)	F(10a)-C(76a)	1.345(4)
C(117)-C(118)	1.390(8)	F(11a)-C(76a)	1.347(4)
C(117)-C(122)	1.396(8)	F(12a)-C(76a)	1.349(4)
C(117)-B(2)	1.643(8)		

Table S12. Bond angles (°) of **3**$^{\mathbf{H}}$ with standard uncertainties in parentheses.

N(1) -Au(1) -O(1)	176.21(17)	C(126)-C(125)-B(2)	123.8(5)
N(1) -Au(1) -C(11)	82.1(2)	C(130)-C(125)-B(2)	121.4(5)
O(1) -Au(1) -C(11)	94.2(2)	C(125)-C(126)-C(127)	122.4(6)
N(1) -Au(1) -P(1)	85.14(14)	C(128)-C(127)-C(126)	121.2(6)
O(1) -Au(1) -P(1)	98.59(12)	C(128)-C(127)-C(131)	118.4(6)
C(11) -Au(1) -P(1)	166.97(19)	C(126)-C(127)-C(131)	120.4(6)
C(1) -P(1) -C(17)	107.6(3)	C(127)-C(128)-C(129)	117.7(6)
C(1) -P(1) -C(21)	108.3(3)	C(130)-C(129)-C(128)	120.4(6)

C(17) -P(1) -C(21)	118.3(3)	C(130)-C(129)-C(132)	119.5(6)
C(1) -P(1) -Au(1)	96.34(19)	C(128)-C(129)-C(132)	120.0(6)
C(17) -P(1) -Au(1)	111.2(2)	C(129)-C(130)-C(125)	123.7(6)
C(21) -P(1) -Au(1)	112.6(2)	F(51) -C(131)-F(49)	108.2(8)
C(16) -O(1) -Au(1)	114.4(4)	F(51) -C(131)-F(50)	105.3(7)
C(9) -N(1) -C(6)	122.7(5)	F(49) -C(131)-F(50)	100.4(7)
C(9) -N(1) -Au(1)	115.7(4)	F(51) -C(131)-C(127)	116.3(7)
C(6) -N(1) -Au(1)	121.3(4)	F(49) -C(131)-C(127)	112.4(7)
C(2) -C(1) -C(6)	117.6(5)	F(50) -C(131)-C(127)	112.9(7)
C(2) -C(1) -P(1)	125.2(5)	F(54) -C(132)-F(52)	104.8(4)
C(6) -C(1) -P(1)	117.2(4)	F(52a)-C(132)-F(53a)	103.7(4)
C(1) -C(2) -C(3)	121.6(6)	F(54) -C(132)-F(53)	104.4(4)
C(4) -C(3) -C(2)	120.5(6)	F(52) -C(132)-F(53)	103.9(4)
C(3) -C(4) -C(5)	121.1(6)	F(52a)-C(132)-F(54a)	103.4(4)
C(4) -C(5) -C(7)	124.6(6)	F(53a)-C(132)-F(54a)	102.8(4)
C(4) -C(5) -C(6)	118.0(6)	F(54) -C(132)-C(129)	118.2(6)
C(7) -C(5) -C(6)	117.4(6)	F(52) -C(132)-C(129)	114.0(6)
N(1) -C(6) -C(1)	119.8(5)	F(52a)-C(132)-C(129)	125.9(12)
N(1) -C(6) -C(5)	119.0(5)	F(53a)-C(132)-C(129)	118.0(10)
C(1) -C(6) -C(5)	121.2(5)	F(53) -C(132)-C(129)	110.2(6)
C(8) -C(7) -C(5)	121.7(6)	F(54a)-C(132)-C(129)	99.5(11)
C(7) -C(8) -C(9)	119.9(6)	C(109)-B(2) -C(101)	109.8(4)
N(1) -C(9) -C(8)	119.2(6)	C(109)-B(2) -C(117)	110.0(4)
N(1) -C(9) -C(10)	114.6(5)	C(101)-B(2) -C(117)	110.0(4)
C(8) -C(9) -C(10)	126.2(6)	C(109)-B(2) -C(125)	110.6(4)
C(15) -C(10) -C(11)	119.4(6)	C(101)-B(2) -C(125)	109.6(4)

C(15) -C(10) -C(9)	124.1(6)	C(117)-B(2) -C(125)	106.8(4)
C(11) -C(10) -C(9)	116.5(5)	C(62) -C(61) -C(66)	115.4(5)
C(12) -C(11) -C(10)	120.6(6)	C(62) -C(61) -B(1)	125.1(5)
C(12) -C(11) -Au(1)	128.5(5)	C(66) -C(61) -B(1)	119.5(5)
C(10) -C(11) -Au(1)	110.8(4)	C(63) -C(62) -C(61)	122.1(5)
C(11) -C(12) -C(13)	118.7(7)	C(64) -C(63) -C(62)	121.5(5)
C(14) -C(13) -C(12)	121.0(7)	C(64) -C(63) -C(67)	116.8(5)
C(13) -C(14) -C(15)	121.5(7)	C(62) -C(63) -C(67)	121.7(5)
C(14) -C(15) -C(10)	118.8(7)	C(63) -C(64) -C(65)	117.5(6)
O(2) -C(16) -O(1)	126.2(6)	C(66) -C(65) -C(64)	120.8(6)
C(18) -C(17) -C(19)	108.7(6)	C(66) -C(65) -C(68)	118.8(5)
C(18) -C(17) -C(20)	109.5(6)	C(64) -C(65) -C(68)	120.3(5)
C(19) -C(17) -C(20)	110.2(6)	C(65) -C(66) -C(61)	122.7(5)
C(18) -C(17) -P(1)	105.1(4)	F(2) -C(67) -F(1)	106.9(4)
C(19) -C(17) -P(1)	109.6(4)	F(3a) -C(67) -F(2a)	106.1(4)
C(20) -C(17) -P(1)	113.4(5)	F(3a) -C(67) -F(1a)	103.4(4)
C(22) -C(21) -C(24)	110.1(5)	F(2a) -C(67) -F(1a)	102.0(3)
C(22) -C(21) -C(23)	110.0(6)	F(2) -C(67) -F(3)	102.9(3)
C(24) -C(21) -C(23)	107.9(6)	F(1) -C(67) -F(3)	101.9(3)
C(22) -C(21) -P(1)	109.7(5)	F(2) -C(67) -C(63)	117.2(5)
C(24) -C(21) -P(1)	114.4(4)	F(3a) -C(67) -C(63)	118.1(6)
C(23) -C(21) -P(1)	104.6(4)	F(1) -C(67) -C(63)	114.4(5)
N(2) -Au(2) -O(3)	175.44(17)	F(2a) -C(67) -C(63)	116.7(5)
N(2) -Au(2) -C(41)	82.2(2)	F(1a) -C(67) -C(63)	108.7(5)
O(3) -Au(2) -C(41)	93.3(2)	F(3) -C(67) -C(63)	111.8(5)
N(2) -Au(2) -P(2)	85.06(12)	F(6a) -C(68) -F(4a)	105.2(4)

O(3) -Au(2) -P(2)	99.50(12)	F(5) -C(68) -F(4)	104.8(4)
C(41) -Au(2) -P(2)	166.96(17)	F(5) -C(68) -F(6)	104.0(4)
C(31) -P(2) -C(51)	108.0(3)	F(4) -C(68) -F(6)	102.8(3)
C(31) -P(2) -C(47)	107.4(3)	F(6a) -C(68) -F(5a)	103.6(4)
C(51) -P(2) -C(47)	118.7(3)	F(4a) -C(68) -F(5a)	103.2(4)
C(31) -P(2) -Au(2)	96.50(18)	F(5) -C(68) -C(65)	121.8(6)
C(51) -P(2) -Au(2)	112.13(19)	F(6a) -C(68) -C(65)	119.3(7)
C(47) -P(2) -Au(2)	111.5(2)	F(4a) -C(68) -C(65)	114.1(6)
C(46) -O(3) -Au(2)	112.7(4)	F(4) -C(68) -C(65)	112.5(5)
C(39) -N(2) -C(36)	122.4(5)	F(6) -C(68) -C(65)	109.1(6)
C(39) -N(2) -Au(2)	115.8(3)	F(5a) -C(68) -C(65)	109.9(6)
C(36) -N(2) -Au(2)	121.8(4)	C(70a)-C(69) -C(74a)	120.0
C(32) -C(31) -C(36)	118.0(5)	C(70) -C(69) -C(74)	118.0(8)
C(32) -C(31) -P(2)	124.9(5)	C(70) -C(69) -B(1)	123.3(5)
C(36) -C(31) -P(2)	117.1(4)	C(70a)-C(69) -B(1)	109.8(5)
C(31) -C(32) -C(33)	121.0(6)	C(74a)-C(69) -B(1)	129.5(5)
C(34) -C(33) -C(32)	120.9(6)	C(74) -C(69) -B(1)	118.7(6)
C(33) -C(34) -C(35)	120.5(6)	C(71a)-C(70a)-C(69)	120.0
C(34) -C(35) -C(36)	118.3(6)	C(70a)-C(71a)-C(72a)	120.0
C(34) -C(35) -C(37)	124.0(6)	C(70a)-C(71a)-C(75)	126.4(8)
C(36) -C(35) -C(37)	117.7(5)	C(72a)-C(71a)-C(75)	113.4(8)
N(2) -C(36) -C(35)	119.2(5)	C(73a)-C(72a)-C(71a)	120.0
N(2) -C(36) -C(31)	119.5(5)	C(72a)-C(73a)-C(74a)	120.0
C(35) -C(36) -C(31)	121.3(5)	C(72a)-C(73a)-C(76a)	118.9(9)
C(38) -C(37) -C(35)	121.2(6)	C(74a)-C(73a)-C(76a)	121.0(9)
C(37) -C(38) -C(39)	120.0(5)	C(73a)-C(74a)-C(69)	120.0

N(2) -C(39) -C(38)	119.5(5)	C(78) -C(77) -C(82)	114.9(6)
N(2) -C(39) -C(40)	114.6(5)	C(78) -C(77) -B(1)	125.2(6)
C(38) -C(39) -C(40)	125.9(5)	C(82) -C(77) -B(1)	119.8(5)
C(41) -C(40) -C(45)	119.7(5)	C(79) -C(78) -C(77)	122.5(6)
C(41) -C(40) -C(39)	116.4(5)	C(80) -C(79) -C(78)	121.7(6)
C(45) -C(40) -C(39)	123.8(5)	C(80) -C(79) -C(83)	119.1(6)
C(42) -C(41) -C(40)	120.7(5)	C(78) -C(79) -C(83)	119.2(6)
C(42) -C(41) -Au(2)	128.3(5)	C(79) -C(80) -C(81)	117.6(7)
C(40) -C(41) -Au(2)	110.9(4)	C(82) -C(81) -C(80)	120.1(7)
C(41) -C(42) -C(43)	118.9(6)	C(82) -C(81) -C(84)	121.2(7)
C(44) -C(43) -C(42)	121.2(6)	C(80) -C(81) -C(84)	118.8(7)
C(43) -C(44) -C(45)	120.9(6)	C(81) -C(82) -C(77)	123.1(7)
C(44) -C(45) -C(40)	118.6(6)	F(15a)-C(83) -F(13a)	111.3(13)
O(4) -C(46) -O(3)	127.8(6)	F(14) -C(83) -F(15)	107.3(7)
C(50) -C(47) -C(48)	110.3(6)	F(14) -C(83) -F(13)	104.3(4)
C(50) -C(47) -C(49)	108.3(6)	F(15) -C(83) -F(13)	103.2(4)
C(48) -C(47) -C(49)	111.2(7)	F(15a)-C(83) -F(14a)	103.9(4)
C(50) -C(47) -P(2)	105.9(4)	F(13a)-C(83) -F(14a)	103.6(4)
C(48) -C(47) -P(2)	113.0(5)	F(14) -C(83) -C(79)	114.1(6)
C(49) -C(47) -P(2)	107.9(5)	F(15a)-C(83) -C(79)	116.3(9)
C(54) -C(51) -C(52)	110.5(5)	F(13a)-C(83) -C(79)	113.8(9)
C(54) -C(51) -C(53)	109.1(5)	F(15) -C(83) -C(79)	114.8(5)
C(52) -C(51) -C(53)	108.1(6)	F(13) -C(83) -C(79)	112.0(5)
C(54) -C(51) -P(2)	109.7(4)	F(14a)-C(83) -C(79)	106.4(8)
C(52) -C(51) -P(2)	114.4(5)	F(18a)-C(84) -F(17a)	104.5(4)
C(53) -C(51) -P(2)	104.8(4)	F(16) -C(84) -F(17)	104.2(4)

C(102)-C(101)-C(106)	115.4(5)	F(16) -C(84) -F(18)	104.0(12)
C(102)-C(101)-B(2)	122.0(5)	F(17) -C(84) -F(18)	103.9(4)
C(106)-C(101)-B(2)	122.5(5)	F(18a)-C(84) -F(16a)	94.8(13)
C(101)-C(102)-C(103)	122.4(5)	F(17a)-C(84) -F(16a)	103.8(4)
C(104)-C(103)-C(102)	120.6(5)	F(18a)-C(84) -C(81)	121.5(8)
C(104)-C(103)-C(107)	119.3(6)	F(16) -C(84) -C(81)	117.6(9)
C(102)-C(103)-C(107)	119.9(6)	F(17a)-C(84) -C(81)	119.8(9)
C(103)-C(104)-C(105)	118.5(5)	F(17) -C(84) -C(81)	116.3(9)
C(104)-C(105)-C(106)	121.0(5)	F(18) -C(84) -C(81)	109.4(9)
C(104)-C(105)-C(108)	119.1(5)	F(16a)-C(84) -C(81)	108.3(11)
C(106)-C(105)-C(108)	119.8(6)	C(86) -C(85) -C(90)	115.3(5)
C(105)-C(106)-C(101)	122.1(5)	C(86) -C(85) -B(1)	120.4(5)
F(33) -C(107)-F(31)	103.5(7)	C(90) -C(85) -B(1)	124.3(5)
F(33) -C(107)-F(32)	105.3(7)	C(87) -C(86) -C(85)	122.9(6)
F(31) -C(107)-F(32)	104.9(8)	C(88) -C(87) -C(86)	120.5(6)
F(33) -C(107)-C(103)	116.0(8)	C(88) -C(87) -C(91)	118.9(6)
F(31) -C(107)-C(103)	113.5(6)	C(86) -C(87) -C(91)	120.6(6)
F(32) -C(107)-C(103)	112.5(7)	C(87) -C(88) -C(89)	118.4(6)
F(34) -C(108)-F(35)	105.7(7)	C(88) -C(89) -C(90)	121.0(5)
F(34) -C(108)-F(36)	106.1(6)	C(88) -C(89) -C(92)	118.1(5)
F(35) -C(108)-F(36)	103.9(7)	C(90) -C(89) -C(92)	120.9(5)
F(34) -C(108)-C(105)	113.3(6)	C(89) -C(90) -C(85)	121.9(6)
F(35) -C(108)-C(105)	114.2(6)	F(20a)-C(91) -F(19a)	105.3(4)
F(36) -C(108)-C(105)	112.8(6)	F(21) -C(91) -F(19)	104.7(4)
C(110)-C(109)-C(114)	114.7(5)	F(20a)-C(91) -F(21a)	104.4(4)
C(110)-C(109)-B(2)	123.3(5)	F(19a)-C(91) -F(21a)	102.8(3)

C(114)-C(109)-B(2)	121.9(5)	F(21) -C(91) -F(20)	104.2(4)
C(109)-C(110)-C(111)	122.1(5)	F(19) -C(91) -F(20)	102.6(3)
C(112)-C(111)-C(110)	121.8(6)	F(20a)-C(91) -C(87)	120.3(6)
C(112)-C(111)-C(115)	117.9(5)	F(21) -C(91) -C(87)	123.9(7)
C(110)-C(111)-C(115)	120.2(5)	F(19a)-C(91) -C(87)	111.6(5)
C(111)-C(112)-C(113)	117.7(6)	F(19) -C(91) -C(87)	111.2(6)
C(114)-C(113)-C(112)	119.8(5)	F(21a)-C(91) -C(87)	110.9(6)
C(114)-C(113)-C(116)	119.7(5)	F(20) -C(91) -C(87)	108.1(6)
C(112)-C(113)-C(116)	120.4(5)	F(24) -C(92) -F(23)	105.2(4)
C(113)-C(114)-C(109)	123.7(5)	F(22a)-C(92) -F(24a)	104.2(4)
F(37a)-C(115)-F(38a)	104.5(4)	F(24) -C(92) -F(22)	103.8(4)
F(38) -C(115)-F(39)	104.1(4)	F(23) -C(92) -F(22)	104.2(3)
F(37a)-C(115)-F(39a)	103.7(4)	F(22a)-C(92) -F(23a)	103.8(4)
F(38a)-C(115)-F(39a)	103.6(4)	F(24a)-C(92) -F(23a)	103.3(4)
F(38) -C(115)-F(37)	103.6(4)	F(24) -C(92) -C(89)	114.3(6)
F(39) -C(115)-F(37)	103.8(4)	F(23) -C(92) -C(89)	118.4(6)
F(38) -C(115)-C(111)	118.2(5)	F(22a)-C(92) -C(89)	122.9(9)
F(37a)-C(115)-C(111)	120.3(9)	F(24a)-C(92) -C(89)	116.6(11)
F(38a)-C(115)-C(111)	120.3(7)	F(22) -C(92) -C(89)	109.6(5)
F(39) -C(115)-C(111)	116.2(6)	F(23a)-C(92) -C(89)	103.7(9)
F(39a)-C(115)-C(111)	101.8(8)	C(85) -B(1) -C(61)	110.5(5)
F(37) -C(115)-C(111)	109.3(6)	C(85) -B(1) -C(77)	108.1(5)
F(42a)-C(116)-F(40a)	106.8(4)	C(61) -B(1) -C(77)	109.2(5)
F(41) -C(116)-F(42)	104.4(4)	C(85) -B(1) -C(69)	113.4(5)
F(41) -C(116)-F(40)	103.8(4)	C(61) -B(1) -C(69)	104.4(5)
F(42) -C(116)-F(40)	101.3(3)	C(77) -B(1) -C(69)	111.1(5)

F(42a)-C(116)-F(41a)	103.4(4)
F(40a)-C(116)-F(41a)	102.0(3)
F(42a)-C(116)-C(113)	116.0(6)
F(41) -C(116)-C(113)	128.4(7)
F(40a)-C(116)-C(113)	116.3(5)
F(42) -C(116)-C(113)	110.2(6)
F(40) -C(116)-C(113)	105.5(6)
F(41a)-C(116)-C(113)	110.7(5)
C(118)-C(117)-C(122)	115.5(5)
C(118)-C(117)-B(2)	122.9(5)
C(122)-C(117)-B(2)	121.6(5)
C(117)-C(118)-C(119)	122.6(6)
C(120)-C(119)-C(118)	120.7(6)
C(120)-C(119)-C(123)	120.8(6)
C(118)-C(119)-C(123)	118.4(6)
C(119)-C(120)-C(121)	118.2(6)
C(120)-C(121)-C(122)	121.0(6)
C(120)-C(121)-C(124)	119.3(6)
C(122)-C(121)-C(124)	119.5(7)
C(117)-C(122)-C(121)	122.0(6)
F(43) -C(123)-F(45)	110.2(8)
F(43) -C(123)-F(44)	102.9(6)
F(45) -C(123)-F(44)	103.4(6)
F(43) -C(123)-C(119)	113.3(6)
F(45) -C(123)-C(119)	115.2(6)
F(44) -C(123)-C(119)	110.7(7)
C(69) -C(70) -C(71)	120.6(9)
C(70) -C(71) -C(72)	120.9(10)
C(70) -C(71) -C(75a)	115.6(7)
C(72) -C(71) -C(75a)	123.5(7)
C(73) -C(72) -C(71)	119.1(9)
C(72) -C(73) -C(74)	119.2(9)
C(72) -C(73) -C(76)	117.8(7)
C(74) -C(73) -C(76)	122.5(8)
C(69) -C(74) -C(73)	122.1(9)
F(8a) -C(75a)-F(7a)	103.7(4)
F(8a) -C(75a)-F(9a)	103.9(4)
F(7a) -C(75a)-F(9a)	103.8(4)
F(8a) -C(75a)-C(71)	120.0(7)
F(7a) -C(75a)-C(71)	112.9(7)
F(9a) -C(75a)-C(71)	111.0(8)
F(10) -C(76) -F(12)	105.9(9)
F(10) -C(76) -F(11)	103.9(4)
F(12) -C(76) -F(11)	103.4(4)
F(10) -C(76) -C(73)	114.4(7)
F(12) -C(76) -C(73)	115.2(7)
F(11) -C(76) -C(73)	112.8(10)
F(8) -C(75) -F(9)	104.0(4)
F(8) -C(75) -F(7)	104.1(4)
F(9) -C(75) -F(7)	104.1(4)
F(8) -C(75) -C(71a)	108.0(11)
F(9) -C(75) -C(71a)	115.7(11)

F(46) -C(124)-F(47)	105.4(8)	F(7) -C(75) -C(71a)	119.4(10)
F(46) -C(124)-F(48)	106.4(7)	F(10a)-C(76a)-F(11a)	104.3(4)
F(47) -C(124)-F(48)	102.7(7)	F(10a)-C(76a)-F(12a)	104.2(4)
F(46) -C(124)-C(121)	112.9(7)	F(11a)-C(76a)-F(12a)	104.0(4)
F(47) -C(124)-C(121)	114.1(7)	F(10a)-C(76a)-C(73a)	114.4(9)
F(48) -C(124)-C(121)	114.4(7)	F(11a)-C(76a)-C(73a)	115.1(11)
C(126)-C(125)-C(130)	114.6(5)	F(12a)-C(76a)-C(73a)	113.6(10)

Table S13. Torsion angles (°) of **3**^{H} with standard uncertainties in parentheses

C(17) -P(1) -C(1) -C(2)	-66.5(6)	B(2) -C(125)-C(130)-C(129)	173.9(6)
C(21) -P(1) -C(1) -C(2)	62.4(6)	C(128)-C(127)-C(131)-F(51)	17(1)
Au(1) -P(1) -C(1) -C(2)	178.8(5)	C(126)-C(127)-C(131)-F(51)	-163.8(8)
C(17) -P(1) -C(1) -C(6)	112.5(5)	C(128)-C(127)-C(131)-F(49)	142.1(8)
C(21) -P(1) -C(1) -C(6)	-118.6(4)	C(126)-C(127)-C(131)-F(49)	-38(1)
Au(1) -P(1) -C(1) -C(6)	-2.2(4)	C(128)-C(127)-C(131)-F(50)	-105.2(9)
C(6) -C(1) -C(2) -C(3)	0.5(9)	C(126)-C(127)-C(131)-F(50)	74.3(9)
P(1) -C(1) -C(2) -C(3)	179.4(5)	C(130)-C(129)-C(132)-F(54)	25(1)
C(1) -C(2) -C(3) -C(4)	0(1)	C(128)-C(129)-C(132)-F(54)	-157.1(8)
C(2) -C(3) -C(4) -C(5)	-0(1)	C(130)-C(129)-C(132)-F(52)	149.0(7)
C(3) -C(4) -C(5) -C(7)	-179.9(6)	C(128)-C(129)-C(132)-F(52)	-33(1)
C(3) -C(4) -C(5) -C(6)	0.0(9)	C(130)-C(129)-C(132)-F(52a)	-179(1)
C(9) -N(1) -C(6) -C(1)	178.8(5)	C(128)-C(129)-C(132)-F(52a)	-1(2)
Au(1) -N(1) -C(6) -C(1)	4.0(7)	C(130)-C(129)-C(132)-F(53a)	-43(2)
C(9) -N(1) -C(6) -C(5)	-1.7(8)	C(128)-C(129)-C(132)-F(53a)	134(1)
Au(1) -N(1) -C(6) -C(5)	-176.6(4)	C(130)-C(129)-C(132)-F(53)	-94.6(8)
C(2) -C(1) -C(6) -N(1)	178.6(5)	C(128)-C(129)-C(132)-F(53)	83.1(9)

P(1) -C(1) -C(6) -N(1)	-0.5(7)	C(130)-C(129)-C(132)-F(54a)	67(1)
C(2) -C(1) -C(6) -C(5)	-0.9(8)	C(128)-C(129)-C(132)-F(54a)	-115(1)
P(1) -C(1) -C(6) -C(5)	-179.9(4)	C(110)-C(109)-B(2) -C(101)	16.5(8)
C(4) -C(5) -C(6) -N(1)	-178.8(5)	C(114)-C(109)-B(2) -C(101)	-164.7(5)
C(7) -C(5) -C(6) -N(1)	1.2(8)	C(110)-C(109)-B(2) -C(117)	137.7(6)
C(4) -C(5) -C(6) -C(1)	0.7(8)	C(114)-C(109)-B(2) -C(117)	-43.5(7)
C(7) -C(5) -C(6) -C(1)	-179.4(5)	C(110)-C(109)-B(2) -C(125)	-104.6(6)
C(4) -C(5) -C(7) -C(8)	-179.4(6)	C(114)-C(109)-B(2) -C(125)	74.3(6)
C(6) -C(5) -C(7) -C(8)	0.7(9)	C(102)-C(101)-B(2) -C(109)	-88.5(6)
C(5) -C(7) -C(8) -C(9)	-2.1(9)	C(106)-C(101)-B(2) -C(109)	90.9(6)
C(6) -N(1) -C(9) -C(8)	0.4(8)	C(102)-C(101)-B(2) -C(117)	150.3(5)
Au(1) -N(1) -C(9) -C(8)	175.5(4)	C(106)-C(101)-B(2) -C(117)	-30.2(7)
C(6) -N(1) -C(9) -C(10)	-179.3(5)	C(102)-C(101)-B(2) -C(125)	33.1(7)
Au(1) -N(1) -C(9) -C(10)	-4.2(6)	C(106)-C(101)-B(2) -C(125)	-147.4(5)
C(7) -C(8) -C(9) -N(1)	1.5(9)	C(118)-C(117)-B(2) -C(109)	155.4(5)
C(7) -C(8) -C(9) -C(10)	-178.8(6)	C(122)-C(117)-B(2) -C(109)	-26.6(7)
N(1) -C(9) -C(10) -C(15)	-176.6(5)	C(118)-C(117)-B(2) -C(101)	-83.6(6)
C(8) -C(9) -C(10) -C(15)	3.7(9)	C(122)-C(117)-B(2) -C(101)	94.4(6)
N(1) -C(9) -C(10) -C(11)	2.6(7)	C(118)-C(117)-B(2) -C(125)	35.3(7)
C(8) -C(9) -C(10) -C(11)	-177.1(5)	C(122)-C(117)-B(2) -C(125)	-146.6(5)
C(15) -C(10) -C(11) -C(12)	1.6(9)	C(126)-C(125)-B(2) -C(109)	-25.6(7)
C(9) -C(10) -C(11) -C(12)	-177.7(5)	C(130)-C(125)-B(2) -C(109)	160.6(5)
C(15) -C(10) -C(11) -Au(1)	179.4(5)	C(126)-C(125)-B(2) -C(101)	-146.8(5)
C(9) -C(10) -C(11) -Au(1)	0.2(6)	C(130)-C(125)-B(2) -C(101)	39.4(7)
C(10) -C(11) -C(12) -C(13)	-1.4(9)	C(126)-C(125)-B(2) -C(117)	94.1(6)
Au(1) -C(11) -C(12) -C(13)	-178.8(5)	C(130)-C(125)-B(2) -C(117)	-79.7(6)

C(11) -C(12) -C(13) -C(14)	1(1)	C(66) -C(61) -C(62) -C(63)	-1.0(8)
C(12) -C(13) -C(14) -C(15)	-1(1)	B(1) -C(61) -C(62) -C(63)	177.0(6)
C(13) -C(14) -C(15) -C(10)	1(1)	C(61) -C(62) -C(63) -C(64)	0.3(9)
C(11) -C(10) -C(15) -C(14)	-1.5(9)	C(61) -C(62) -C(63) -C(67)	-178.9(5)
C(9) -C(10) -C(15) -C(14)	177.7(6)	C(62) -C(63) -C(64) -C(65)	0.1(9)
Au(1) -O(1) -C(16) -O(2)	-3.9(9)	C(67) -C(63) -C(64) -C(65)	179.4(5)
C(1) -P(1) -C(17) -C(18)	-52.6(5)	C(63) -C(64) -C(65) -C(66)	0.2(9)
C(21) -P(1) -C(17) -C(18)	-175.6(4)	C(63) -C(64) -C(65) -C(68)	-179.4(5)
Au(1) -P(1) -C(17) -C(18)	51.8(4)	C(64) -C(65) -C(66) -C(61)	-1.0(9)
C(1) -P(1) -C(17) -C(19)	-169.3(5)	C(68) -C(65) -C(66) -C(61)	178.7(5)
C(21) -P(1) -C(17) -C(19)	67.6(6)	C(62) -C(61) -C(66) -C(65)	1.3(8)
Au(1) -P(1) -C(17) -C(19)	-65.0(5)	B(1) -C(61) -C(66) -C(65)	-176.8(5)
C(1) -P(1) -C(17) -C(20)	67.0(6)	C(64) -C(63) -C(67) -F(2)	88.5(7)
C(21) -P(1) -C(17) -C(20)	-56.0(6)	C(62) -C(63) -C(67) -F(2)	-92.2(7)
Au(1) -P(1) -C(17) -C(20)	171.4(5)	C(64) -C(63) -C(67) -F(3a)	-90.9(8)
C(1) -P(1) -C(21) -C(22)	-179.3(5)	C(62) -C(63) -C(67) -F(3a)	88.4(8)
C(17) -P(1) -C(21) -C(22)	-56.6(5)	C(64) -C(63) -C(67) -F(1)	-145.2(6)
Au(1) -P(1) -C(21) -C(22)	75.4(5)	C(62) -C(63) -C(67) -F(1)	34.1(8)
C(1) -P(1) -C(21) -C(24)	-55.1(6)	C(64) -C(63) -C(67) -F(2a)	37.3(8)
C(17) -P(1) -C(21) -C(24)	67.6(6)	C(62) -C(63) -C(67) -F(2a)	-143.4(7)
Au(1) -P(1) -C(21) -C(24)	-160.3(5)	C(64) -C(63) -C(67) -F(1a)	151.8(6)
C(1) -P(1) -C(21) -C(23)	62.8(5)	C(62) -C(63) -C(67) -F(1a)	-28.9(8)
C(17) -P(1) -C(21) -C(23)	-174.5(4)	C(64) -C(63) -C(67) -F(3)	-30.0(7)
Au(1) -P(1) -C(21) -C(23)	-42.5(5)	C(62) -C(63) -C(67) -F(3)	149.3(6)
C(51) -P(2) -C(31) -C(32)	-66.0(6)	C(66) -C(65) -C(68) -F(5)	-63.6(9)
C(47) -P(2) -C(31) -C(32)	63.1(6)	C(64) -C(65) -C(68) -F(5)	116.1(8)

Au(2) -P(2) -C(31) -C(32)	178.2(5)	C(66) -C(65) -C(68) -F(6a)	79.7(9)
C(51) -P(2) -C(31) -C(36)	116.7(4)	C(64) -C(65) -C(68) -F(6a)	-100.6(8)
C(47) -P(2) -C(31) -C(36)	-114.2(4)	C(66) -C(65) -C(68) -F(4a)	-154.9(8)
Au(2) -P(2) -C(31) -C(36)	0.8(4)	C(64) -C(65) -C(68) -F(4a)	24.8(9)
C(36) -C(31) -C(32) -C(33)	-1.7(9)	C(66) -C(65) -C(68) -F(4)	170.8(8)
P(2) -C(31) -C(32) -C(33)	-179.0(5)	C(64) -C(65) -C(68) -F(4)	-9.5(9)
C(31) -C(32) -C(33) -C(34)	1(1)	C(66) -C(65) -C(68) -F(6)	57.5(8)
C(32) -C(33) -C(34) -C(35)	0(1)	C(64) -C(65) -C(68) -F(6)	-122.9(7)
C(33) -C(34) -C(35) -C(36)	0.9(9)	C(66) -C(65) -C(68) -F(5a)	-39.6(8)
C(33) -C(34) -C(35) -C(37)	-179.9(6)	C(64) -C(65) -C(68) -F(5a)	140.1(7)
C(39) -N(2) -C(36) -C(35)	1.2(7)	C(74a)-C(69) -C(70a)-C(71a)	0.0
Au(2) -N(2) -C(36) -C(35)	179.0(4)	B(1) -C(69) -C(70a)-C(71a)	-171.7(6)
C(39) -N(2) -C(36) -C(31)	-178.2(5)	C(69) -C(70a)-C(71a)-C(72a)	0.0
Au(2) -N(2) -C(36) -C(31)	-0.4(6)	C(69) -C(70a)-C(71a)-C(75)	175(2)
C(34) -C(35) -C(36) -N(2)	178.3(5)	C(70a)-C(71a)-C(72a)-C(73a)	0.0
C(37) -C(35) -C(36) -N(2)	-0.9(8)	C(75) -C(71a)-C(72a)-C(73a)	-176(1)
C(34) -C(35) -C(36) -C(31)	-2.2(8)	C(71a)-C(72a)-C(73a)-C(74a)	0.0
C(37) -C(35) -C(36) -C(31)	178.5(5)	C(71a)-C(72a)-C(73a)-C(76a)	177(2)
C(32) -C(31) -C(36) -N(2)	-177.9(5)	C(72a)-C(73a)-C(74a)-C(69)	0.0
P(2) -C(31) -C(36) -N(2)	-0.4(7)	C(76a)-C(73a)-C(74a)-C(69)	-177(2)
C(32) -C(31) -C(36) -C(35)	2.6(8)	C(70a)-C(69) -C(74a)-C(73a)	0.0
P(2) -C(31) -C(36) -C(35)	-179.8(4)	B(1) -C(69) -C(74a)-C(73a)	169.8(7)
C(34) -C(35) -C(37) -C(38)	-179.8(6)	C(82) -C(77) -C(78) -C(79)	1(1)
C(36) -C(35) -C(37) -C(38)	-0.6(8)	B(1) -C(77) -C(78) -C(79)	-179.3(6)
C(35) -C(37) -C(38) -C(39)	1.9(9)	C(77) -C(78) -C(79) -C(80)	-2(1)
C(36) -N(2) -C(39) -C(38)	0.1(7)	C(77) -C(78) -C(79) -C(83)	179.1(6)

Au(2) -N(2) -C(39) -C(38)	-177.9(4)	C(78) -C(79) -C(80) -C(81)	1(1)
C(36) -N(2) -C(39) -C(40)	-178.3(4)	C(83) -C(79) -C(80) -C(81)	-179.8(8)
Au(2) -N(2) -C(39) -C(40)	3.8(5)	C(79) -C(80) -C(81) -C(82)	0(2)
C(37) -C(38) -C(39) -N(2)	-1.6(8)	C(79) -C(80) -C(81) -C(84)	179.4(9)
C(37) -C(38) -C(39) -C(40)	176.5(5)	C(80) -C(81) -C(82) -C(77)	-1(2)
N(2) -C(39) -C(40) -C(41)	-1.0(7)	C(84) -C(81) -C(82) -C(77)	179.5(9)
C(38) -C(39) -C(40) -C(41)	-179.2(5)	C(78) -C(77) -C(82) -C(81)	1(1)
N(2) -C(39) -C(40) -C(45)	175.4(5)	B(1) -C(77) -C(82) -C(81)	-179.1(9)
C(38) -C(39) -C(40) -C(45)	-2.8(9)	C(80) -C(79) -C(83) -F(14)	-6(1)
C(45) -C(40) -C(41) -C(42)	-1.9(8)	C(78) -C(79) -C(83) -F(14)	173.4(7)
C(39) -C(40) -C(41) -C(42)	174.7(5)	C(80) -C(79) -C(83) -F(15a)	163(1)
C(45) -C(40) -C(41) -Au(2)	-178.7(4)	C(78) -C(79) -C(83) -F(15a)	-18(1)
C(39) -C(40) -C(41) -Au(2)	-2.1(6)	C(80) -C(79) -C(83) -F(13a)	-66(1)
C(40) -C(41) -C(42) -C(43)	0.1(9)	C(78) -C(79) -C(83) -F(13a)	113(1)
Au(2) -C(41) -C(42) -C(43)	176.2(4)	C(80) -C(79) -C(83) -F(15)	118.9(8)
C(41) -C(42) -C(43) -C(44)	1(1)	C(78) -C(79) -C(83) -F(15)	-62.1(9)
C(42) -C(43) -C(44) -C(45)	-1(1)	C(80) -C(79) -C(83) -F(13)	-123.8(8)
C(43) -C(44) -C(45) -C(40)	-0.9(9)	C(78) -C(79) -C(83) -F(13)	55.2(8)
C(41) -C(40) -C(45) -C(44)	2.3(8)	C(80) -C(79) -C(83) -F(14a)	48(1)
C(39) -C(40) -C(45) -C(44)	-174.0(5)	C(78) -C(79) -C(83) -F(14a)	-133(1)
Au(2) -O(3) -C(46) -O(4)	3.4(9)	C(82) -C(81) -C(84) -F(18a)	-8(2)
C(31) -P(2) -C(47) -C(50)	46.8(5)	C(80) -C(81) -C(84) -F(18a)	173(1)
C(51) -P(2) -C(47) -C(50)	169.6(4)	C(82) -C(81) -C(84) -F(16)	62(2)
Au(2) -P(2) -C(47) -C(50)	-57.8(5)	C(80) -C(81) -C(84) -F(16)	-117(1)
C(31) -P(2) -C(47) -C(48)	-74.0(6)	C(82) -C(81) -C(84) -F(17a)	-141(1)
C(51) -P(2) -C(47) -C(48)	48.7(7)	C(80) -C(81) -C(84) -F(17a)	40(2)

Au(2) -P(2) -C(47) -C(48)	-178.6(6)	C(82) -C(81) -C(84) -F(17)	-173(1)
C(31) -P(2) -C(47) -C(49)	162.6(5)	C(80) -C(81) -C(84) -F(17)	8(2)
C(51) -P(2) -C(47) -C(49)	-74.7(6)	C(82) -C(81) -C(84) -F(18)	-56(1)
Au(2) -P(2) -C(47) -C(49)	58.0(6)	C(80) -C(81) -C(84) -F(18)	125(1)
C(31) -P(2) -C(51) -C(54)	179.9(4)	C(82) -C(81) -C(84) -F(16a)	100(1)
C(47) -P(2) -C(51) -C(54)	57.4(5)	C(80) -C(81) -C(84) -F(16a)	-79(1)
Au(2) -P(2) -C(51) -C(54)	-75.0(5)	C(90) -C(85) -C(86) -C(87)	2.4(9)
C(31) -P(2) -C(51) -C(52)	55.0(6)	B(1) -C(85) -C(86) -C(87)	-176.5(6)
C(47) -P(2) -C(51) -C(52)	-67.4(6)	C(85) -C(86) -C(87) -C(88)	-1(1)
Au(2) -P(2) -C(51) -C(52)	160.2(5)	C(85) -C(86) -C(87) -C(91)	179.5(6)
C(31) -P(2) -C(51) -C(53)	-63.2(5)	C(86) -C(87) -C(88) -C(89)	-0(1)
C(47) -P(2) -C(51) -C(53)	174.4(4)	C(91) -C(87) -C(88) -C(89)	179.0(6)
Au(2) -P(2) -C(51) -C(53)	42.0(4)	C(87) -C(88) -C(89) -C(90)	0(1)
C(106)-C(101)-C(102)-C(103)	0.5(8)	C(87) -C(88) -C(89) -C(92)	-177.3(6)
B(2) -C(101)-C(102)-C(103)	180.0(5)	C(88) -C(89) -C(90) -C(85)	1.0(9)
C(101)-C(102)-C(103)-C(104)	-1(1)	C(92) -C(89) -C(90) -C(85)	178.5(5)
C(101)-C(102)-C(103)-C(107)	174.6(7)	C(86) -C(85) -C(90) -C(89)	-2.3(9)
C(102)-C(103)-C(104)-C(105)	0.7(9)	B(1) -C(85) -C(90) -C(89)	176.6(6)
C(107)-C(103)-C(104)-C(105)	-175.2(7)	C(88) -C(87) -C(91) -F(20a)	158.7(7)
C(103)-C(104)-C(105)-C(106)	0.6(9)	C(86) -C(87) -C(91) -F(20a)	-22(1)
C(103)-C(104)-C(105)-C(108)	178.7(6)	C(88) -C(87) -C(91) -F(21)	4(1)
C(104)-C(105)-C(106)-C(101)	-1.5(8)	C(86) -C(87) -C(91) -F(21)	-176.5(8)
C(108)-C(105)-C(106)-C(101)	-179.5(5)	C(88) -C(87) -C(91) -F(19a)	-77.2(8)
C(102)-C(101)-C(106)-C(105)	0.9(8)	C(86) -C(87) -C(91) -F(19a)	102.1(8)
B(2) -C(101)-C(106)-C(105)	-178.6(5)	C(88) -C(87) -C(91) -F(19)	-121.9(8)
C(104)-C(103)-C(107)-F(33)	-34(1)	C(86) -C(87) -C(91) -F(19)	57.4(9)

C(102)-C(103)-C(107)-F(33)	149.8(7)	C(88) -C(87) -C(91) -F(21a)	36.7(8)
C(104)-C(103)-C(107)-F(31)	-153.9(8)	C(86) -C(87) -C(91) -F(21a)	-144.0(7)
C(102)-C(103)-C(107)-F(31)	30(1)	C(88) -C(87) -C(91) -F(20)	126.2(8)
C(104)-C(103)-C(107)-F(32)	87.1(9)	C(86) -C(87) -C(91) -F(20)	-54.5(9)
C(102)-C(103)-C(107)-F(32)	-88.9(9)	C(88) -C(89) -C(92) -F(24)	-142.2(6)
C(104)-C(105)-C(108)-F(34)	-87.9(8)	C(90) -C(89) -C(92) -F(24)	40.2(8)
C(106)-C(105)-C(108)-F(34)	90.1(8)	C(88) -C(89) -C(92) -F(23)	-17.4(9)
C(104)-C(105)-C(108)-F(35)	151.0(6)	C(90) -C(89) -C(92) -F(23)	164.9(8)
C(106)-C(105)-C(108)-F(35)	-31.0(9)	C(88) -C(89) -C(92) -F(22a)	74(1)
C(104)-C(105)-C(108)-F(36)	32.7(9)	C(90) -C(89) -C(92) -F(22a)	-103(1)
C(106)-C(105)-C(108)-F(36)	-149.2(6)	C(88) -C(89) -C(92) -F(24a)	-155(1)
C(114)-C(109)-C(110)-C(111)	1(1)	C(90) -C(89) -C(92) -F(24a)	27(1)
B(2) -C(109)-C(110)-C(111)	-179.6(6)	C(88) -C(89) -C(92) -F(22)	101.8(8)
C(109)-C(110)-C(111)-C(112)	0(1)	C(90) -C(89) -C(92) -F(22)	-75.8(9)
C(109)-C(110)-C(111)-C(115)	-175.3(6)	C(88) -C(89) -C(92) -F(23a)	-43(1)
C(110)-C(111)-C(112)-C(113)	-1(1)	C(90) -C(89) -C(92) -F(23a)	140(1)
C(115)-C(111)-C(112)-C(113)	175.1(7)	C(86) -C(85) -B(1) -C(61)	77.7(7)
C(111)-C(112)-C(113)-C(114)	-1(1)	C(90) -C(85) -B(1) -C(61)	-101.1(6)
C(111)-C(112)-C(113)-C(116)	177.4(6)	C(86) -C(85) -B(1) -C(77)	-41.8(7)
C(112)-C(113)-C(114)-C(109)	3(1)	C(90) -C(85) -B(1) -C(77)	139.4(6)
C(116)-C(113)-C(114)-C(109)	-175.4(5)	C(86) -C(85) -B(1) -C(69)	-165.6(6)
C(110)-C(109)-C(114)-C(113)	-3.0(9)	C(90) -C(85) -B(1) -C(69)	15.7(8)
B(2) -C(109)-C(114)-C(113)	178.0(6)	C(62) -C(61) -B(1) -C(85)	-12.2(8)
C(112)-C(111)-C(115)-F(38)	148.3(8)	C(66) -C(61) -B(1) -C(85)	165.8(5)
C(110)-C(111)-C(115)-F(38)	-36(1)	C(62) -C(61) -B(1) -C(77)	106.7(6)
C(112)-C(111)-C(115)-F(37a)	-8(1)	C(66) -C(61) -B(1) -C(77)	-75.4(7)

C(110)-C(111)-C(115)-F(37a)	168(1)	C(62) -C(61) -B(1) -C(69)	-134.4(6)
C(112)-C(111)-C(115)-F(38a)	-141(1)	C(66) -C(61) -B(1) -C(69)	43.5(7)
C(110)-C(111)-C(115)-F(38a)	35(1)	C(78) -C(77) -B(1) -C(85)	121.2(7)
C(112)-C(111)-C(115)-F(39)	23(1)	C(82) -C(77) -B(1) -C(85)	-59.0(8)
C(110)-C(111)-C(115)-F(39)	-160.8(7)	C(78) -C(77) -B(1) -C(61)	0.9(9)
C(112)-C(111)-C(115)-F(39a)	106(1)	C(82) -C(77) -B(1) -C(61)	-179.3(7)
C(110)-C(111)-C(115)-F(39a)	-79(1)	C(78) -C(77) -B(1) -C(69)	-113.7(7)
C(112)-C(111)-C(115)-F(37)	-93.6(9)	C(82) -C(77) -B(1) -C(69)	66.1(9)
C(110)-C(111)-C(115)-F(37)	82.2(9)	C(70) -C(69) -B(1) -C(85)	-68(1)
C(114)-C(113)-C(116)-F(42a)	-61.2(8)	C(70a)-C(69) -B(1) -C(85)	-71.3(8)
C(112)-C(113)-C(116)-F(42a)	120.6(7)	C(74a)-C(69) -B(1) -C(85)	118.1(9)
C(114)-C(113)-C(116)-F(41)	92(1)	C(74) -C(69) -B(1) -C(85)	113(1)
C(112)-C(113)-C(116)-F(41)	-86(1)	C(70) -C(69) -B(1) -C(61)	53(1)
C(114)-C(113)-C(116)-F(40a)	171.9(6)	C(70a)-C(69) -B(1) -C(61)	49.0(8)
C(112)-C(113)-C(116)-F(40a)	-6.3(9)	C(74a)-C(69) -B(1) -C(61)	-121.6(8)
C(114)-C(113)-C(116)-F(42)	-36.9(9)	C(74) -C(69) -B(1) -C(61)	-127(1)
C(112)-C(113)-C(116)-F(42)	144.9(8)	C(70) -C(69) -B(1) -C(77)	170.4(9)
C(114)-C(113)-C(116)-F(40)	-145.5(8)	C(70a)-C(69) -B(1) -C(77)	166.6(7)
C(112)-C(113)-C(116)-F(40)	36.3(9)	C(74a)-C(69) -B(1) -C(77)	-4(1)
C(114)-C(113)-C(116)-F(41a)	56.1(7)	C(74) -C(69) -B(1) -C(77)	-9(1)
C(112)-C(113)-C(116)-F(41a)	-122.1(6)	C(74) -C(69) -C(70) -C(71)	0(2)
C(122)-C(117)-C(118)-C(119)	0.6(8)	B(1) -C(69) -C(70) -C(71)	-179.7(9)
B(2) -C(117)-C(118)-C(119)	178.7(5)	C(69) -C(70) -C(71) -C(72)	-1(2)
C(117)-C(118)-C(119)-C(120)	2.3(9)	C(69) -C(70) -C(71) -C(75a)	179(1)
C(117)-C(118)-C(119)-C(123)	179.0(5)	C(70) -C(71) -C(72) -C(73)	-1(2)
C(118)-C(119)-C(120)-C(121)	-2.8(9)	C(75a)-C(71) -C(72) -C(73)	179(1)

C(123)-C(119)-C(120)-C(121)	-179.4(6)	C(71) -C(72) -C(73) -C(74)	3(2)
C(119)-C(120)-C(121)-C(122)	1(1)	C(71) -C(72) -C(73) -C(76)	175(1)
C(119)-C(120)-C(121)-C(124)	175.9(7)	C(70) -C(69) -C(74) -C(73)	2(2)
C(118)-C(117)-C(122)-C(121)	-3.0(8)	B(1) -C(69) -C(74) -C(73)	-178(1)
B(2) -C(117)-C(122)-C(121)	178.9(5)	C(72) -C(73) -C(74) -C(69)	-3(2)
C(120)-C(121)-C(122)-C(117)	3(1)	C(76) -C(73) -C(74) -C(69)	-176(1)
C(124)-C(121)-C(122)-C(117)	-172.9(6)	C(70) -C(71) -C(75a)-F(8a)	30(1)
C(120)-C(119)-C(123)-F(43)	92.0(9)	C(72) -C(71) -C(75a)-F(8a)	-150(1)
C(118)-C(119)-C(123)-F(43)	-84.7(8)	C(70) -C(71) -C(75a)-F(7a)	152.7(9)
C(120)-C(119)-C(123)-F(45)	-139.9(7)	C(72) -C(71) -C(75a)-F(7a)	-27(1)
C(118)-C(119)-C(123)-F(45)	43.4(9)	C(70) -C(71) -C(75a)-F(9a)	-91(1)
C(120)-C(119)-C(123)-F(44)	-23.0(9)	C(72) -C(71) -C(75a)-F(9a)	89(1)
C(118)-C(119)-C(123)-F(44)	160.3(6)	C(72) -C(73) -C(76) -F(10)	50(2)
C(120)-C(121)-C(124)-F(46)	-89.2(9)	C(74) -C(73) -C(76) -F(10)	-138(1)
C(122)-C(121)-C(124)-F(46)	86.3(9)	C(72) -C(73) -C(76) -F(12)	173(1)
C(120)-C(121)-C(124)-F(47)	150.6(7)	C(74) -C(73) -C(76) -F(12)	-15(2)
C(122)-C(121)-C(124)-F(47)	-34(1)	C(72) -C(73) -C(76) -F(11)	-69(1)
C(120)-C(121)-C(124)-F(48)	33(1)	C(74) -C(73) -C(76) -F(11)	104(1)
C(122)-C(121)-C(124)-F(48)	-151.8(7)	C(70a)-C(71a)-C(75) -F(8)	37(1)
C(130)-C(125)-C(126)-C(127)	1.1(9)	C(72a)-C(71a)-C(75) -F(8)	-147.9(9)
B(2) -C(125)-C(126)-C(127)	-173.0(5)	C(70a)-C(71a)-C(75) -F(9)	-79(1)
C(125)-C(126)-C(127)-C(128)	-1(1)	C(72a)-C(71a)-C(75) -F(9)	96(1)
C(125)-C(126)-C(127)-C(131)	179.8(7)	C(70a)-C(71a)-C(75) -F(7)	155.4(8)
C(126)-C(127)-C(128)-C(129)	-0(1)	C(72a)-C(71a)-C(75) -F(7)	-30(1)
C(131)-C(127)-C(128)-C(129)	179.1(7)	C(72a)-C(73a)-C(76a)-F(10a)	48(1)
C(127)-C(128)-C(129)-C(130)	1(1)	C(74a)-C(73a)-C(76a)-F(10a)	-135.9(8)

C(127)-C(128)-C(129)-C(132)	-176.6(7)	C(72a)-C(73a)-C(76a)-F(11a)	-73(1)
C(128)-C(129)-C(130)-C(125)	-1(1)	C(74a)-C(73a)-C(76a)-F(11a)	103(1)
C(132)-C(129)-C(130)-C(125)	177.0(7)	C(72a)-C(73a)-C(76a)-F(12a)	167.1(9)
C(126)-C(125)-C(130)-C(129)	-0(1)	C(74a)-C(73a)-C(76a)-F(12a)	-16(1)

(P^N^C^H)Au-H (4^H) (CCDC 2264147)

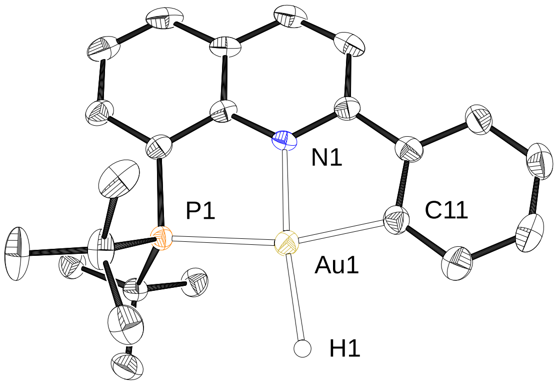

Note: The structure of $C_{23}H_{28}AuNP^+$ BF_4^- has been solved and refined successfully. The space group is non-centrosymmetric, but the presence of mirror or glide planes dictates that the compound in the crystal, if the molecule possesses an asymmetric center or chiral axis, is racemic. Refinement of the absolute structure parameter[12] yielded a value of 0.219(8), which indicates that the crystals are inversion twins. The BF_4^- anion is disordered though a pivot about one B–F bond. Two sets of positions were defined for three F-atoms and the site occupation factor of the major orientation of the anion refined to 0.841(7). Similarity restraints were applied to all B–F bonds, as well as to the F···F distances among the disordered F-atoms, while neighboring F-atoms within and between each of the disordered orientations were restrained to have similar atomic displacement parameters. The hydride H-atom was placed in the position indicated by a difference electron density map (Figure S69) and its position was allowed to refine together with an isotropic displacement parameter.

The unit cell constants and an orientation matrix for data collection were obtained from a least-squares refinement of the setting angles of 17896 reflections in the range 5° < 2θ < 58°. A total of 964 frames were collected using ω scans with κ offsets, 22.5 seconds exposure time and a rotation angle of 0.5° per frame, and a crystal-detector distance of 65.0 mm.

Table S14. Crystallographic data of **4^H**.

Crystallized from	CH_2Cl_2 / *n*-pentane
Empirical formula	$C_{23}H_{28}AuBF_4NP$
Formula weight [g mol^{-1}]	633.21
Crystal color, habit	colorless, plate
Crystal dimensions [mm]	0.03 x 0.12 x 0.31

Temperature [K]	160(1)
Crystal system	orthorhombic
Space group	*Fdd2 (#43)*
Z	16
Reflections for the cell determination	17896
2θ range for cell determination [º]	5 – 58
Unit cell parameters *a* [Å]	45.0288(4)
b [Å]	25.4812(3)
c [Å]	8.08952(9)
α [º]	90
β [º]	90
γ [º]	90
V [Å^3]	9281.82(17)
F(000)	4928
D_x [g·cm^3]	1.813
μ(Mo Kα) [mm^{-1}]	6.451
Scan type	*ω*
$2\theta_{(max)}$ [º]	57.6
Transmission factors (min; max)	0.473; 1.000
Total reflections measured	24778
Symmetry independent reflections	5636
R_{int}	0.023
Reflections with $I > 2\sigma(I)$	5377
Reflections used in refinement	5636
Parameters refined; restrains	319; 73
Final *R*(*F*) [$I > 2\sigma(I)$ reflections]	0.0241
$wR(F^2)$ (all data)	0.0599
Weights	$w = [\sigma^2(F_o^2) + (0.0354P)^2 + 44.4650P]^{-1}$ where $P = (F_o^2 + 2F_c^2)/3$
Goodness of fit	1.049
Final Δ_{max}/σ	0.005
$\Delta\rho$ (max; min) [e Å^{-3}]	0.79; -0.41
$\sigma(d_{(C-C)})$ [Å]	0.008 – 0.011

Table S15. Bond lengths (Å) of **4^H^** with standard uncertainties in parentheses.

Au(1) -N(1)	2.049(5)	C(10) -C(11)	1.402(9)
Au(1) -C(11)	2.055(6)	C(11) -C(12)	1.395(10)
Au(1) -P(1)	2.3591(15)	C(12) -C(13)	1.396(9)
P(1) -C(1)	1.828(6)	C(13) -C(14)	1.368(11)
P(1) -C(16)	1.862(6)	C(14) -C(15)	1.399(10)
P(1) -C(20)	1.865(6)	C(16) -C(19)	1.521(9)
N(1) -C(9)	1.343(7)	C(16) -C(18)	1.535(10)
N(1) -C(6)	1.363(7)	C(16) -C(17)	1.540(10)
C(1) -C(2)	1.387(8)	C(20) -C(23)	1.529(9)
C(1) -C(6)	1.420(8)	C(20) -C(22)	1.529(9)
C(2) -C(3)	1.397(10)	C(20) -C(21)	1.545(8)
C(3) -C(4)	1.365(11)	F(1) -B(1)	1.366(5)
C(4) -C(5)	1.408(10)	F(2) -B(1)	1.364(5)
C(5) -C(6)	1.411(8)	F(3) -B(1)	1.363(5)
C(5) -C(7)	1.427(10)	F(4) -B(1)	1.367(5)
C(7) -C(8)	1.356(10)	F(2a) -B(1)	1.360(6)
C(8) -C(9)	1.410(9)	F(3a) -B(1)	1.364(6)
C(9) -C(10)	1.478(9)	F(4a) -B(1)	1.363(6)
C(10) -C(15)	1.392(8)		

Table S16. Selected bond lengths (Å) and angles (°) of **4^H^** involving H-atoms.

Au(1) -H(1)	2.03(6)

N(1) -Au(1) -H(1)	172.9(18)	P(1) -Au(1) -H(1)	102.8(17)
C(11) -Au(1) -H(1)	91.6(17)		

Table S17. Bond angles (°) of **4^H^** with standard uncertainties in parentheses.

N(1) -Au(1) -C(11)	81.4(2)	C(12) -C(11) -C(10)	118.9(6)
N(1) -Au(1) -P(1)	84.19(14)	C(12) -C(11) -Au(1)	129.9(5)

C(11) -Au(1) -P(1)	165.45(18)
C(1) -P(1) -C(16)	109.9(3)
C(1) -P(1) -C(20)	105.2(3)
C(16) -P(1) -C(20)	115.6(3)
C(1) -P(1) -Au(1)	97.6(2)
C(16) -P(1) -Au(1)	112.1(2)
C(20) -P(1) -Au(1)	114.5(2)
C(9) -N(1) -C(6)	123.1(5)
C(9) -N(1) -Au(1)	115.7(4)
C(6) -N(1) -Au(1)	121.2(4)
C(2) -C(1) -C(6)	117.8(6)
C(2) -C(1) -P(1)	125.1(5)
C(6) -C(1) -P(1)	117.1(4)
C(1) -C(2) -C(3)	121.4(6)
C(4) -C(3) -C(2)	120.8(6)
C(3) -C(4) -C(5)	119.9(6)
C(4) -C(5) -C(6)	119.3(6)
C(4) -C(5) -C(7)	124.1(6)
C(6) -C(5) -C(7)	116.6(6)
N(1) -C(6) -C(5)	119.9(5)
N(1) -C(6) -C(1)	119.6(5)
C(5) -C(6) -C(1)	120.5(6)
C(8) -C(7) -C(5)	121.7(6)
C(7) -C(8) -C(9)	119.6(6)
N(1) -C(9) -C(8)	119.0(6)
N(1) -C(9) -C(10)	113.8(5)
C(8) -C(9) -C(10)	127.2(5)
C(15) -C(10) -C(11)	121.5(7)
C(15) -C(10) -C(9)	120.7(6)
C(11) -C(10) -C(9)	117.8(5)
C(10) -C(11) -Au(1)	111.1(4)
C(11) -C(12) -C(13)	119.3(7)
C(14) -C(13) -C(12)	121.4(6)
C(13) -C(14) -C(15)	120.4(6)
C(10) -C(15) -C(14)	118.5(7)
C(19) -C(16) -C(18)	109.3(6)
C(19) -C(16) -C(17)	111.1(6)
C(18) -C(16) -C(17)	108.8(5)
C(19) -C(16) -P(1)	114.7(5)
C(18) -C(16) -P(1)	107.3(5)
C(17) -C(16) -P(1)	105.4(5)
C(23) -C(20) -C(22)	110.6(5)
C(23) -C(20) -C(21)	108.9(5)
C(22) -C(20) -C(21)	108.8(6)
C(23) -C(20) -P(1)	108.8(5)
C(22) -C(20) -P(1)	114.3(4)
C(21) -C(20) -P(1)	105.2(4)
F(2a) -B(1) -F(4a)	108.4(6)
F(2a) -B(1) -F(3a)	108.1(6)
F(4a) -B(1) -F(3a)	107.8(6)
F(3) -B(1) -F(2)	107.5(4)
F(2a) -B(1) -F(1)	131.4(15)
F(3) -B(1) -F(1)	110.8(5)
F(4a) -B(1) -F(1)	108.3(11)
F(3a) -B(1) -F(1)	89.7(16)
F(2) -B(1) -F(1)	114.1(6)
F(3) -B(1) -F(4)	108.1(4)
F(2) -B(1) -F(4)	107.4(4)
F(1) -B(1) -F(4)	108.7(5)

Table S18. Torsion angles (°) of **4^H** with standard uncertainties in parentheses.

C(16)	-P(1)	-C(1)	-C(2)	-59.1(6)	N(1)	-C(9)	-C(10)	-C(15)	-174.4(5)
C(20)	-P(1)	-C(1)	-C(2)	66.0(6)	C(8)	-C(9)	-C(10)	-C(15)	7.1(9)
Au(1)	-P(1)	-C(1)	-C(2)	-176.0(5)	N(1)	-C(9)	-C(10)	-C(11)	4.6(7)
C(16)	-P(1)	-C(1)	-C(6)	121.0(5)	C(8)	-C(9)	-C(10)	-C(11)	-174.0(6)
C(20)	-P(1)	-C(1)	-C(6)	-113.9(5)	C(15)	-C(10)	-C(11)	-C(12)	-0.8(9)
Au(1)	-P(1)	-C(1)	-C(6)	4.1(4)	C(9)	-C(10)	-C(11)	-C(12)	-179.7(5)
C(6)	-C(1)	-C(2)	-C(3)	3.5(9)	C(15)	-C(10)	-C(11)	-Au(1)	176.1(5)
P(1)	-C(1)	-C(2)	-C(3)	-176.4(5)	C(9)	-C(10)	-C(11)	-Au(1)	-2.9(7)
C(1)	-C(2)	-C(3)	-C(4)	1(1)	C(10)	-C(11)	-C(12)	-C(13)	0.5(9)
C(2)	-C(3)	-C(4)	-C(5)	-5(1)	Au(1)	-C(11)	-C(12)	-C(13)	-175.7(5)
C(3)	-C(4)	-C(5)	-C(6)	3.1(9)	C(11)	-C(12)	-C(13)	-C(14)	0(1)
C(3)	-C(4)	-C(5)	-C(7)	-175.6(6)	C(12)	-C(13)	-C(14)	-C(15)	-1(1)
C(9)	-N(1)	-C(6)	-C(5)	2.1(8)	C(11)	-C(10)	-C(15)	-C(14)	0.1(9)
Au(1)	-N(1)	-C(6)	-C(5)	-176.5(4)	C(9)	-C(10)	-C(15)	-C(14)	179.0(6)
C(9)	-N(1)	-C(6)	-C(1)	-176.9(5)	C(13)	-C(14)	-C(15)	-C(10)	1(1)
Au(1)	-N(1)	-C(6)	-C(1)	4.5(7)	C(1)	-P(1)	-C(16)	-C(19)	78.1(7)
C(4)	-C(5)	-C(6)	-N(1)	-177.4(5)	C(20)	-P(1)	-C(16)	-C(19)	-40.8(7)
C(7)	-C(5)	-C(6)	-N(1)	1.4(8)	Au(1)	-P(1)	-C(16)	-C(19)	-174.5(5)
C(4)	-C(5)	-C(6)	-C(1)	1.6(8)	C(1)	-P(1)	-C(16)	-C(18)	-160.4(5)
C(7)	-C(5)	-C(6)	-C(1)	-179.6(5)	C(20)	-P(1)	-C(16)	-C(18)	80.8(5)
C(2)	-C(1)	-C(6)	-N(1)	174.2(5)	Au(1)	-P(1)	-C(16)	-C(18)	-52.9(5)
P(1)	-C(1)	-C(6)	-N(1)	-5.9(7)	C(1)	-P(1)	-C(16)	-C(17)	-44.5(5)
C(2)	-C(1)	-C(6)	-C(5)	-4.9(8)	C(20)	-P(1)	-C(16)	-C(17)	-163.4(4)
P(1)	-C(1)	-C(6)	-C(5)	175.1(4)	Au(1)	-P(1)	-C(16)	-C(17)	62.9(4)
C(4)	-C(5)	-C(7)	-C(8)	175.6(6)	C(1)	-P(1)	-C(20)	-C(23)	-173.3(4)
C(6)	-C(5)	-C(7)	-C(8)	-3.1(9)	C(16)	-P(1)	-C(20)	-C(23)	-51.9(5)
C(5)	-C(7)	-C(8)	-C(9)	1(1)	Au(1)	-P(1)	-C(20)	-C(23)	80.7(4)
C(6)	-N(1)	-C(9)	-C(8)	-4.0(8)	C(1)	-P(1)	-C(20)	-C(22)	-49.2(5)
Au(1)	-N(1)	-C(9)	-C(8)	174.7(4)	C(16)	-P(1)	-C(20)	-C(22)	72.3(5)
C(6)	-N(1)	-C(9)	-C(10)	177.4(5)	Au(1)	-P(1)	-C(20)	-C(22)	-155.2(4)

Au(1) -N(1) -C(9) -C(10)	-4.0(6)	C(1) -P(1) -C(20) -C(21)	70.1(5)
C(7) -C(8) -C(9) -N(1)	2.2(9)	C(16) -P(1) -C(20) -C(21)	-168.5(4)
C(7) -C(8) -C(9) -C(10)	-179.4(6)	Au(1) -P(1) -C(20) -C(21)	-35.9(5)

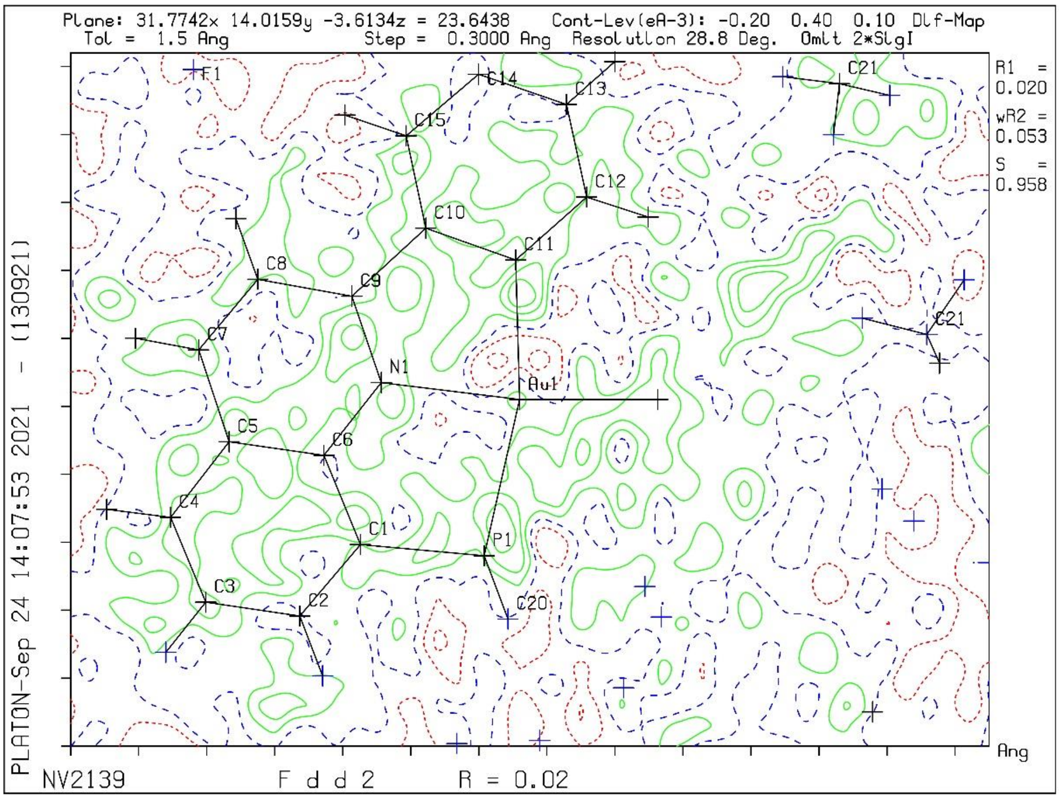

Figure S73. Difference electron density map of the region of the hydride H-atom, in the plane H-Au-C11 of complex **4^H^**. The map was generated by excluding the hydride H from the calculations, while including all other hydrogen atoms. Green contours indicate areas of an accumulation of electron density, while red contours indicate areas where there is a deficit of electron density. The position of the hydride was refined to a distance of 2.03(6) Å from Au.

8. DFT calculations

8.1 Optimization of the gold(III)-hydrides structures

DFT calculations were performed using Gaussian 16.[13] Gas phase geometry optimizations for complex **4^H^** were performed using different methods and conditions, as stated in Table S19.

Table S19. Methods and basis sets used for gas-phase optimizations of complex **4^H**.

**Method**	**Basis set (light atoms/Au)**	**$r_{Au\text{-}H}$ / Å**	**$r_{Au\text{-}C}$ / Å**	**$r_{Au\text{-}N}$ / Å**	**$r_{Au\text{-}P}$ / Å**
X-ray	-	2.03(6)	2.055(6)	2.049(6)	2.3591(15)
B3LYP	6-31G(d,p)/LANL2DZ	1.5648	2.0486	2.1142	2.4623
B3LYP	6-31+G(d,p)/SDDAll	1.56456	2.05848	2.10739	2.43041
B3LYP	6-31+G(d,p)/DEF2QZVPP	1.5648	2.05394	2.09169	2.41402
B3LYP	6-311++G(d,p)/DEF2QZVPP	1.56568	2.05514	2.09419	2.41736
B3LYP	6-311++G(d,p)/DEF2QZVPPD	1.56577	2.05523	2.09425	2.41738
B3PW91	6-311++G(d,p)/DEF2QZVPPD	1.56118	2.04033	2.07417	2.38766
PBEPBE	6-311++G(d,p)/DEF2QZVPPD	1.57366	2.05141	2.08773	2.39738
CAM-B3LYP	6-311++G(d,p)/DEF2QZVPPD	1.55889	2.03983	2.07656	2.39099
HSEH1PBE	6-311++G(d,p)/DEF2QZVPPD	1.56167	2.03831	2.07342	2.38433
PBEPBE	DEF2QZVPP	1.57254	2.05287	2.08488	2.38745
GD3BJ-PBEPBE	DEF2TZVP	1.57551	2.05327	2.08443	2.37657
**GD3BJ-PBE1PBE**	**DEF2TZVP**	**1.56096**	**2.03628**	**2.06508**	**2.35918**

As a good compromise between CPU time and accuracy, as well as to compare with reported calculations,[14] the optimizations of complexes **4^{iPr}** and **4^F** were performed using PBE1PBE[15] (= PBE0) with added empirical dispersion (GD3BJ)[16] in combination with DEF2TZVP[17] as basis sets for all atoms.

For further analysis of the gold(III)-hydrides, molecular orbitals (MO) and charge distributions (NBO) were performed using Gaussian. For further studies via AIM analysis[18] and evaluation of Intrinsic Bond Strength Index (IBSI),[19] Delocalization Index (DI),[20] and Bond order analysis (FBO)[21] were carried out with MultiWFN[22] package.

8.2 Investigation of the Au-H bond

In order to investigate the bond properties, in addition to the three (P^N^C) complexes **4**, the calculated structures of known, experimentally obtained, and characterized Au(III)-hydrides[14] have been optimized and computationally characterized using the method described above. The calculated metrics for the bond analysis in the hydride complexes can be found in Table S20 and a qualitative comparison of some of them has been depicted in Figure S74. Additionally to the bond length, DI (basin analysis) and IBSI, the Localized Orbital Locator (LOL), Electron Localization Function (ELF), and the eigenvalue for the Hessian $\nabla^2\rho$ have been obtained in an AIM calculation and are given for the found critical point between the gold(III)-center and the hydride. The relatively high values for LOL and ELF at the critical point located close to the H, as well as the negative $\nabla^2\rho$ are strong indicators for the covalent nature of the Au-H bond and hence are in perfect agreement to the results for bond length, IBSI and DI, as discussed in the main text. In addition to the DI by basin analysis, the DI was calculated in fuzzy atom space to obtain the bond order (FBO), which was found to be correspond to a single bond.

Table S20. Calculated and experimental data for the Au-H bond analysis in gold(III)-hydrides (method: GD3BJ-PBE1PBE/DEF2TZVP).

**Parameter**	**4^H**	**4^{iPr}**	**4^F**	**A - Ref 14** (C^N^C)Au-H	**B - Ref 14** (C^N^C)Au-H	**4 - Ref 14** (N^C^C)Au-H	**9 - Ref 14** P(C^C)Au-H
r_{Au-H} / Å	1.561	1.558	1.557	1.561	1.553	1.642	1.630
δ_{exp} / ppm	-5.51	-5.18	-5.60	-6.51	-8.34	6.09	1.89
LOL	0.618	0.618	0.627	0.616	0.625	0.554	0.561
ELF	0.724	0.724	0.739	0.720	0.736	0.607	0.620
$\nabla^2\rho$	-0.172	-0.174	-0.174	-0.173	-0.176	-0.143	-0.146
DI (basin analysis)	0.943	0.918	0.932	0.974	0.893	0.918	0.867
DI (fuzzy atom space) / FBO	0.941	0.905	0.914	0.981	0.857	0.940	0.856
IBSI	0.381	0.390	0.382	0.382	0.391	0.333	0.338
Charge(NBO): H	-0.048	-0.062	-0.023	-0.056	-0.024	-0.254	-0.182
Charge(NBO): Au	0.550	0.535	0.538	0.652	0.627	0.660	0.435

Note: For the previously reported compounds, the slight difference in the calculated bond distances in this work as compared to literature[14] stems from the use of different program packages (Gaussian 16 *vs* Turbomole).

All computationally obtained metrics for complexes **4**$^{\mathbf{H}}$, **4**$^{\mathbf{iPr}}$ and **4**$^{\mathbf{F}}$ speak for a highly covalent Au-H bond. For all (P^N^C)gold(III)-hydrides, the computational metrics show very subtle differences due to the electronic nature of the ligand scaffold, but generally reflect the overall clearly protic character of the Au-H observed experimentally. As our findings are in agreement with reported work, which also correlates the character of the Au-H to the chemical reactivity of the gold(III)-hydrides,[14] we propose that either of the calculated metrics presented in Table S20 could be potentially be used for *in silico* reactivity prediction for similar (P^N^C)Au(III)-H complexes.

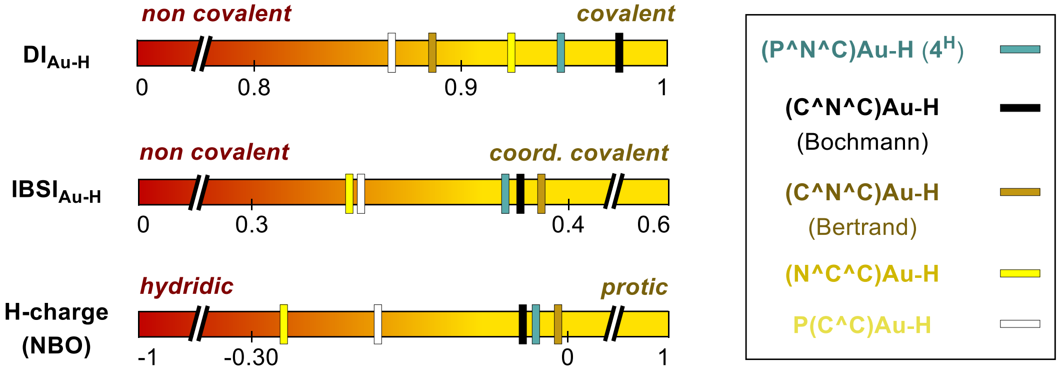

Figure S74. Calculated metrics for the Au-H bond analysis and comparison of DI, IBSI and H-charge (NBO) of complex **4**$^{\mathbf{H}}$ with previously reported gold(III)-hydrides.[14]

8.3 Correlation of chemical shift and calculated bond length

Calculated Au-H distances are reported to be correlated to the observed chemical ^{1}H NMR shift of the gold(III)-hydride.[14] As can be seen in Figure S75, our findings are in excellent agreement with reported results and are located in the region of previously reported mononuclear (C^N^C) complexes, clearly separated from (C^C) and (N^C^C)gold(III)-hydride complexes.

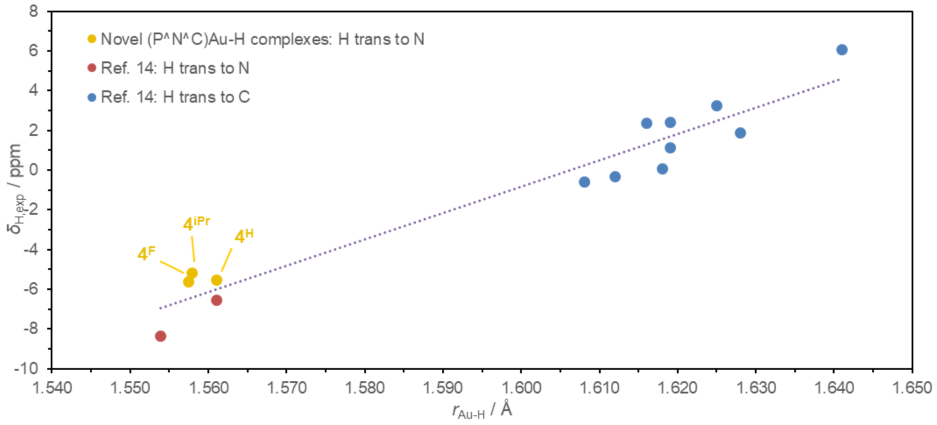

Figure S75. Correlation of experimental ^{1}H NMR shifts and Au-H distances calculated by DFT (GD3BJ-PBE1PBE/DEF2TZVP).

8.4 Molecular orbital analysis

In Figures S76-S78, the relevant occupied MOs for the Au-H bond as well as the lowest relevant unoccupied MO involved in the Au-H bond are shown. In all of the occupied MOs, a strong π-type electron donation by the d-orbitals of the gold(III)-center could be observed, which can be linked to the strong shielding effect observed by NMR. In the unoccupied MOs, the expanded lobe around the Au-H can account for the electrophilic character and hence the protic reactivity observed in experimental studies.

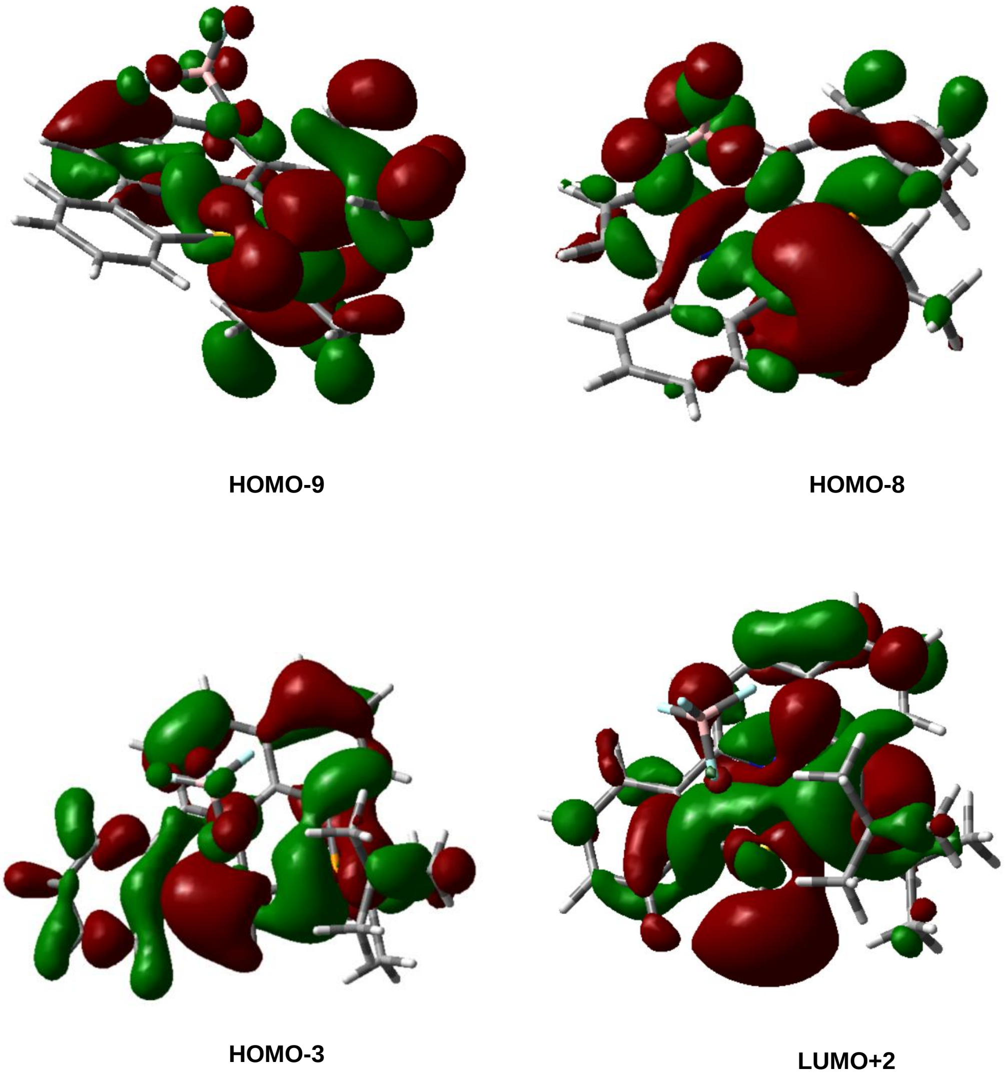

Figure S76. HOMO-9, HOMO-8, HOMO-3 and LUMO+2 for complex $\mathbf{4^H}$ (isosurface plots, 0.02 au).

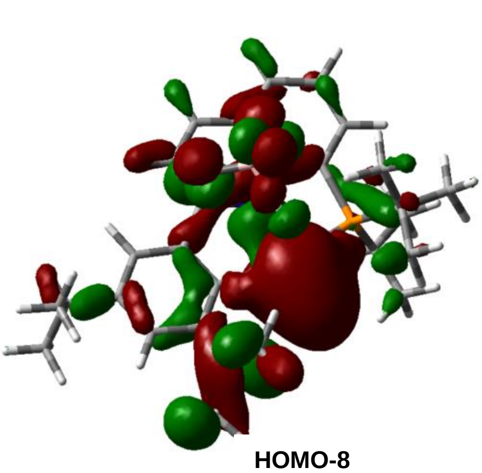

HOMO-8

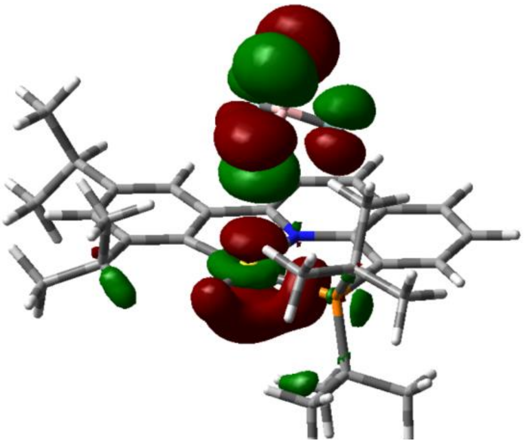

HOMO-5

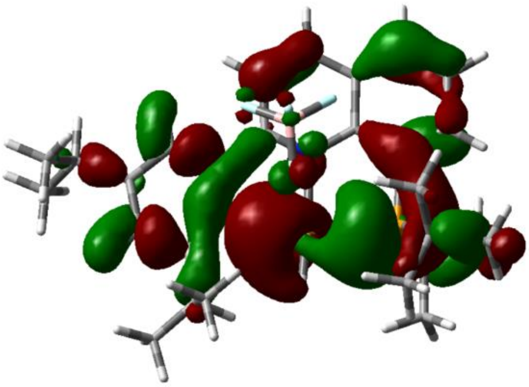

HOMO-2

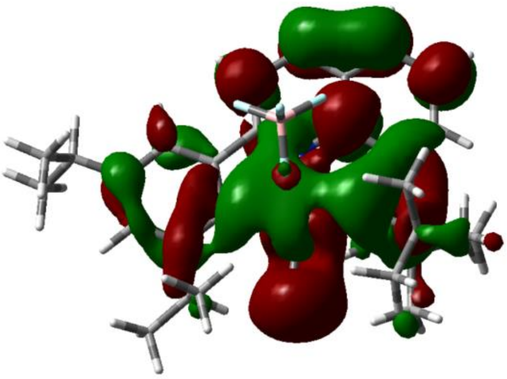

LUMO+2

Figure S77. HOMO-8, HOMO-5, HOMO-2 and LUMO+2 for complex **4**$^{\mathbf{iPr}}$ (isosurface plots, 0.02 au).

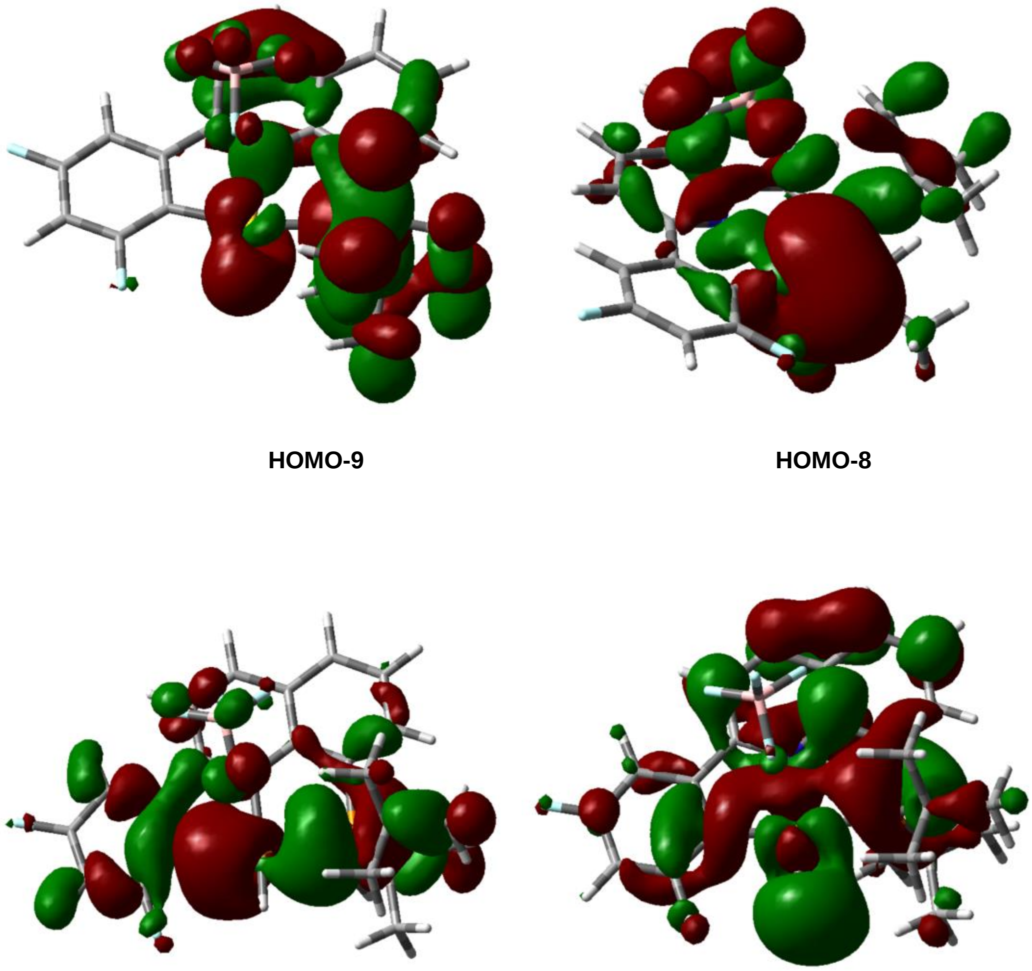

HOMO-9 HOMO-8

HOMO-3 LUMO+2

Figure S78. HOMO-9, HOMO-8, HOMO-3 and LUMO+2 for complex **4^{F}** (isosurface plots, 0.02 au).

8.5 Oxidation state analysis in 4^{H}

In order to evaluate the oxidation state in the (P^N^C)gold(III) hydride **4^{H}**, a localized bonding orbital analysis (LOBA)[23] was carried as implemented in the MultiWFN package[22] (Pipek-Mezey localization based on Mulliken population analysis). The threshold was chosen at 60% as in exemplary cases in the original publication and as a result, the oxidation state of the Au-center was calculated to be +3. Different thresholds were tested, and it was observed that for values in the broad range from 51% to 90% the same oxidation number was obtained. Thus, the calculations strongly support the hypothesized Au(III)-center of the complex.

8.6 Coordinates of the (P^N^C)gold(III)-hydride complexes

4^H

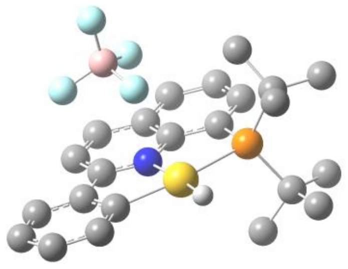

Method: GD3BJ-PBE1PBE/DEF2TZVP

E(RPBE1PBE) = -1848.5966Hartree

Dipole Moment = 12.3226 Debye

Point Group = C1

C	2.53769600	2.19748000	-0.92962800
C	1.59664900	1.21142900	-0.72727500
C	0.25572100	1.47992800	-1.09508600
C	-0.10103700	2.72133200	-1.66410200
C	0.89591800	3.68978900	-1.85836100
C	2.19246900	3.43279900	-1.49538500
C	-1.46495800	2.93001700	-1.96667900
C	-2.39129600	1.96577500	-1.71475000
C	-1.98326700	0.73846600	-1.15191500
H	3.56663600	2.03202700	-0.63705000
H	0.62013200	4.64921700	-2.28126600
H	2.95746900	4.18716900	-1.63151200
H	-1.76819500	3.88519900	-2.38055900
H	-3.43890700	2.14265300	-1.90848400
C	-2.85643000	-0.38469100	-0.82183100
C	-4.22722800	-0.36375100	-1.05952200

C	-2.26186700	-1.51719800	-0.22243700
C	-5.00880900	-1.45408800	-0.72019600
H	-4.69444500	0.50933900	-1.49839200
C	-3.05635500	-2.59845000	0.11061800
C	-4.42473900	-2.56837700	-0.13841400
H	-6.07655700	-1.42985400	-0.90123300
H	-2.61851600	-3.47083300	0.57873500
H	-5.03817500	-3.42024800	0.13343000
N	-0.69864300	0.54516500	-0.88617700
P	1.94240900	-0.40546700	0.04425100
C	2.74578000	-0.12543900	1.70468900
C	2.98174000	-1.34851000	-1.18747800
C	4.22611500	-0.60323900	-1.65920100
H	4.75722800	-1.23795000	-2.37494700
H	3.96577600	0.32161500	-2.17516900
H	4.91708300	-0.37140100	-0.85045400
C	2.07671200	-1.58528400	-2.39866200
H	1.20921900	-2.19730300	-2.14439600
H	1.72272800	-0.64606200	-2.82982700
H	2.64972200	-2.11168900	-3.16756700
C	3.36429400	-2.69508100	-0.57848800
H	3.82982400	-3.31268400	-1.35196500
H	4.08233200	-2.59137200	0.23604100
H	2.48861900	-3.22959900	-0.20394700
C	4.24954300	0.11716800	1.64485800
H	4.59829900	0.32259000	2.66100600
H	4.80259300	-0.74896300	1.27893000
H	4.51014100	0.98366100	1.03544100
C	2.04897200	1.07734600	2.33943500
H	2.28274500	2.01011000	1.82674200
H	0.96395800	0.96804100	2.35843500
H	2.39708100	1.16027000	3.37317200

C	2.45624700	-1.36194000	2.55912900
H	2.86255700	-2.27955900	2.13095300
H	2.91694100	-1.21662900	3.54023800
H	1.38372800	-1.49238800	2.70648100
Au	-0.26557300	-1.23622100	0.06444700
H	-0.03760400	-2.61838900	0.75310300
B	-1.63007200	1.95401300	2.02602400
F	-1.80483100	2.48251200	3.28639400
F	-0.66697500	2.69631900	1.30796200
F	-1.14923800	0.61578400	2.12791000
F	-2.82769200	1.94175800	1.30286900

4^{iPr}

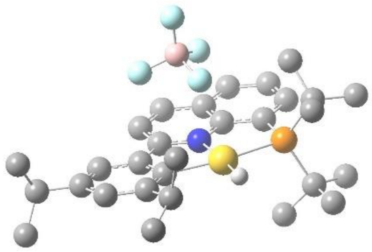

Method: GD3BJ-PBE1PBE/DEF2TZVP

E(RPBE1PBE) = -2084.2931 Hartree

Dipole Moment = 11.8514 Debye

Point Group = C1

C	3.80392000	1.76836400	-0.67156900
C	2.62483200	1.05829600	-0.60549600
C	1.41132600	1.74985500	-0.84032500
C	1.41567700	3.12758800	-1.14566700
C	2.64301800	3.80531000	-1.20737300
C	3.81703300	3.13742900	-0.97235900
C	0.16314500	3.75384700	-1.33568500
C	-0.99440000	3.04728400	-1.22876500
C	-0.94571700	1.66901000	-0.92540600
H	4.74570800	1.27208100	-0.47513000
H	2.64578400	4.86704000	-1.42712600
H	4.76204400	3.66526700	-1.00667800
H	0.13736100	4.81711600	-1.54708200
H	-1.95063900	3.53687700	-1.33826500

C	-2.09854400	0.79200300	-0.75095600
C	-3.39992400	1.25546600	-0.92097600
C	-1.85891800	-0.53955600	-0.35287100
C	-4.47984300	0.42261800	-0.69366500
H	-3.58325100	2.28156400	-1.21867900
C	-2.93709700	-1.38177800	-0.09867700
C	-4.22497900	-0.88104800	-0.28141000
H	-5.06783100	-1.53325200	-0.08213200
N	0.23440700	1.08939500	-0.75920900
P	2.50385100	-0.71098500	-0.17924800
C	3.37514800	-1.00799600	1.44410100
C	3.21493800	-1.63784300	-1.63561600
C	4.61548500	-1.19069000	-2.04172300
H	4.93226900	-1.78804800	-2.90202300
H	4.62716300	-0.14459200	-2.35023600
H	5.35367400	-1.33247500	-1.25421000
C	2.26625900	-1.36414600	-2.80494200
H	1.26123200	-1.74362400	-2.61074500
H	2.19506300	-0.29706800	-3.02742400
H	2.65334700	-1.86695300	-3.69604700
C	3.20110500	-3.13140500	-1.32015700
H	3.46196500	-3.68631800	-2.22596000
H	3.92646800	-3.39808900	-0.55041700
H	2.21255200	-3.46342500	-0.99564700
C	4.88351700	-1.19567300	1.32636000
H	5.28934500	-1.30433900	2.33628800

H	5.15565000	-2.09213300	0.76749000
H	5.37841400	-0.33557700	0.87296700
C	3.06879700	0.18993100	2.34170000
H	3.56226300	1.09996000	2.00106600
H	1.99993600	0.39289500	2.41344600
H	3.43546200	-0.04064200	3.34627600
C	2.74662800	-2.25559600	2.06992100
H	2.85544600	-3.14495100	1.44722600
H	3.24575100	-2.45007100	3.02337000
H	1.68626400	-2.09898600	2.27024900
Au	0.15409000	-0.88357500	-0.15821400
H	0.01667100	-2.37800400	0.25993100
B	-0.30366200	1.94073200	2.47435500
F	-0.32994400	2.27255300	3.81168500
F	0.81239400	2.52895600	1.84023400
F	-0.17701900	0.52558200	2.33543400
F	-1.46799500	2.35146700	1.81971000
C	-2.73607300	-2.77438100	0.45369000
H	-1.82297700	-3.16233100	-0.00774100
C	-5.89311400	0.92580900	-0.85298500
H	-5.82473000	1.95932700	-1.21076200
C	-6.62448200	0.94445600	0.48618400
H	-6.07916500	1.53485300	1.22481900
H	-7.62551900	1.36857000	0.37342700
H	-6.73423600	-0.06783500	0.88451600
C	-6.66745300	0.12202100	-1.89240000

H	-6.15794300	0.12776700	-2.85839000
H	-6.77869500	-0.91977200	-1.58008200
H	-7.66995300	0.53498600	-2.02994100
C	-2.50246300	-2.68887000	1.96138500
H	-2.26871100	-3.67416300	2.37407700
H	-1.68752000	-2.00309200	2.20040200
H	-3.39984800	-2.31411000	2.46156100
C	-3.86068300	-3.74737000	0.13356900
H	-4.78203800	-3.49624700	0.66592100
H	-4.08197900	-3.77419800	-0.93614000
H	-3.57794700	-4.75486000	0.44726800

4^F

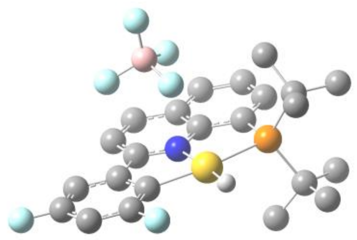

Method: GD3BJ-PBE1PBE/DEF2TZVP

E(RPBE1PBE) = -2046.9836 Hartree

Dipole Moment = 13.4943 Debye

Point Group = C1

C	3.03232400	1.95357400	-0.91365600
C	1.96727400	1.10134400	-0.72210400
C	0.67026100	1.56871500	-1.04745700
C	0.47818200	2.86843100	-1.56318100
C	1.59746100	3.69548900	-1.74884100
C	2.85149200	3.24596800	-1.42775400
C	-0.84882600	3.27539000	-1.82266900
C	-1.89604800	2.43972600	-1.57938400
C	-1.64886800	1.14935300	-1.07050300
H	4.03362300	1.63617200	-0.65256800
H	1.45109600	4.69940900	-2.13092100
H	3.71196500	3.89072900	-1.55682500
H	-1.02499900	4.27804200	-2.19564200
H	-2.91260800	2.76748500	-1.73934700
C	-2.66463400	0.14268500	-0.75324700
C	-4.02094400	0.37268700	-0.95459000
C	-2.20359700	-1.07720000	-0.21711200

C	-4.91051400	-0.62654200	-0.62047200
H	-4.40180000	1.30745600	-1.34228300
C	-3.14481800	-2.03163000	0.09297100
C	-4.50366400	-1.83705800	-0.09762500
H	-5.22112700	-2.60436600	0.16201000
N	-0.40002800	0.76866700	-0.84645400
P	2.10184600	-0.57578700	-0.01255100
C	2.95902200	-0.46842600	1.64025600
C	2.97571500	-1.61162600	-1.29535800
C	4.30127200	-1.02606800	-1.77197000
H	4.72955200	-1.70567600	-2.51467300
H	4.16008700	-0.06023500	-2.25842400
H	5.03065500	-0.91326300	-0.97161900
C	2.02484600	-1.68388000	-2.49236100
H	1.08828800	-2.18392300	-2.23848600
H	1.79228400	-0.69194300	-2.88650000
H	2.50844400	-2.25538900	-3.28981100
C	3.18331100	-3.01658500	-0.73514200
H	3.54501700	-3.66543400	-1.53785500
H	3.92517500	-3.03740300	0.06417800
H	2.25112000	-3.44062600	-0.35584000
C	4.48057700	-0.42496200	1.55398600
H	4.87123400	-0.30537800	2.56849000
H	4.90696000	-1.34247800	1.14637800
H	4.84345400	0.42182000	0.96958600
C	2.44008400	0.79220000	2.33104700
H	2.78811400	1.70426000	1.84660800
H	1.35072100	0.82726700	2.37108800
H	2.81413100	0.78898300	3.35892600
C	2.52230500	-1.68658600	2.45764100
H	2.79532400	-2.63365500	1.99005100
H	3.01593100	-1.63918800	3.43220900

H	1.44500200	-1.67957000	2.62573300
Au	-0.18457900	-1.09070700	0.03350500
H	-0.12558700	-2.51154000	0.66863400
B	-1.09980900	2.15832900	2.12531300
F	-1.18182900	2.66344800	3.40296900
F	-0.06393100	2.79435000	1.40610400
F	-0.79260700	0.76689100	2.17456600
F	-2.30298500	2.32057400	1.42832600
F	-2.76141600	-3.20576700	0.60247800
F	-6.21634600	-0.41851500	-0.80865900

A - Ref. 14

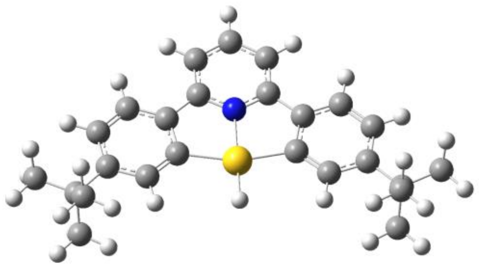

Method: GD3BJ-PBE1PBE/DEF2TZVP

E(RPBE1PBE) = -1159.2297 Hartree

Dipole Moment = 3.1197 Debye

Point Group = C1

.Au	-0.11221070	0.03263960	0.10356490
H	-1.23502380	0.39815090	1.12393910
N	1.35554970	-0.44173940	-1.23151980
C	-1.28736600	-0.47492920	-1.51446530
C	1.53974250	0.38754290	1.28719940
C	0.96739580	-0.83404080	-2.45043170
C	1.95533280	-1.15550910	-3.37540550
C	3.28283920	-1.05237690	-2.98821200
C	3.63499750	-0.63992000	-1.71205140
C	2.62113330	-0.32688470	-0.81258070
C	-0.48921260	-0.86074300	-2.62330770
C	-1.09195880	-1.24200360	-3.81430050
C	-2.47228170	-1.24811550	-3.93393470
C	-3.28287600	-0.87391330	-2.86763260

C	-2.66106440	-0.49367960	-1.67273390
C	2.74131030	0.12772170	0.57709080
C	3.96906570	0.30607710	1.20009500
C	4.03474550	0.73685630	2.51548860
C	2.87666640	1.00009110	3.23922380
C	1.64629780	0.81608430	2.59776660
C	-4.80278370	-0.86162690	-2.95950140
C	-5.37956410	-1.80359810	-1.89822040
C	-5.30867370	0.56171430	-2.70488530
C	-5.30694200	-1.31290890	-4.32592910
C	2.90477730	1.47380440	4.68625390
C	4.32433500	1.62580600	5.22164930
C	2.16551370	0.45698330	5.56163020
C	2.20477010	2.83292960	4.78393520
H	0.73454300	1.01699760	3.14826100
H	5.00670800	0.86578100	2.97255420
H	4.89226790	0.11011280	0.66485620
H	4.67384490	-0.56257950	-1.42280440
H	4.06289870	-1.29942520	-3.69947680
H	1.69396220	-1.47756520	-4.37388820
H	-0.48942260	-1.53930680	-4.66613620
H	-2.91136750	-1.54928550	-4.87555410
H	-3.27917950	-0.19929570	-0.83264230
H	4.86837430	0.67835750	5.20374690
H	4.28757870	1.96647320	6.25907780
H	4.89555540	2.36243350	4.65146470

H	2.20502910	3.18348540	5.81986770
H	1.16841160	2.77660240	4.44742910
H	2.71646200	3.57830570	4.17030970
H	2.64860950	-0.52177250	5.51225150
H	1.12797120	0.33436930	5.24660690
H	2.16561560	0.78728510	6.60403280
H	-5.00659750	0.92514740	-1.72144330
H	-6.40092620	0.58827830	-2.75474350
H	-4.91524080	1.25374760	-3.45350370
H	-4.95160590	-0.65834490	-5.12566970
H	-6.39912020	-1.28600960	-4.33797100
H	-4.99861290	-2.33605040	-4.55450780
H	-5.08245600	-1.50621170	-0.89130090
H	-5.03570620	-2.82813730	-2.05987870
H	-6.47211160	-1.79780490	-1.94366300

B - Ref. 14

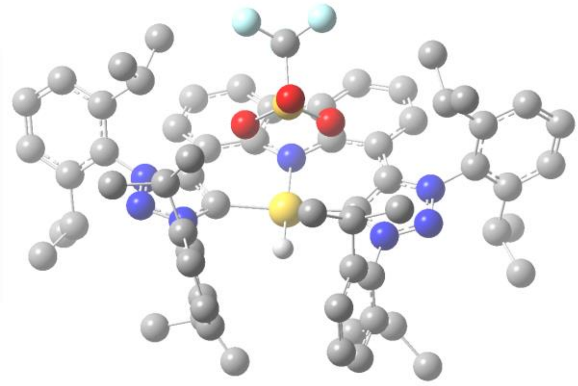

Method: GD3BJ-PBE1PBE/DEF2TZVP

E(RPBE1PBE) = -3961.9397 Hartree

Dipole Moment = 9.0604 Debye

Point Group = C1

Au	-0.05576000	0.51793300	-0.59588800
H	-0.06491600	1.96094700	-0.01958900
N	-0.05493400	-1.40021900	-1.37096700
N	-2.91729500	1.37546000	0.00572000
N	-4.14806300	0.95547100	0.14647100
N	-4.11708100	-0.28413000	-0.28202100
N	2.83106300	1.34256500	0.04900200
N	4.07553600	0.93810300	0.01132700
N	4.03742000	-0.18563900	-0.65776200
C	-1.15239200	-2.17790300	-1.61107500
C	-2.50588200	-1.92697800	-1.31110800
C	-3.42687000	-2.91405300	-1.67538800

H	-4.47606900	-2.76878500	-1.46855100
C	-3.04454400	-4.09334500	-2.30078400
C	-1.71466000	-4.33417800	-2.60529500
H	-1.41813400	-5.25360300	-3.09787000
C	-0.77290500	-3.37866100	-2.26616700
C	1.03632100	-2.06645800	-1.84915400
C	2.39231500	-1.69280500	-1.80576200
C	3.30156100	-2.52849400	-2.46054500
H	4.34801100	-2.26259300	-2.48760700
C	2.91017500	-3.71358300	-3.06998500
C	1.58488300	-4.11938900	-3.04320400
H	1.28637500	-5.05777600	-3.49734700
C	0.64944400	-3.29693000	-2.43932400
C	-2.87753700	-0.66399100	-0.71281200
C	-2.06733000	0.44596000	-0.49479000
C	2.77698600	-0.50420800	-1.07740100
C	1.96372400	0.50882900	-0.58474600
H	-3.80301800	-4.82418200	-2.55289500
H	3.65817100	-4.32712400	-3.55678000
C	-2.62570400	2.74979600	0.29455500
C	-2.16901000	3.07576200	1.57080700
C	-1.84375500	4.40875900	1.79316800
H	-1.46637500	4.70520700	2.76312900
C	-1.98263600	5.35330100	0.79090100
H	-1.71808500	6.38653700	0.98632900
C	-2.45796400	4.99266100	-0.45773800

H	-2.56654100	5.74581500	-1.22888400
C	-2.79353900	3.67218000	-0.73689300
C	-2.06826400	2.03525400	2.65730600
H	-1.85552500	1.07033000	2.18878000
C	-0.94230800	2.30644900	3.64078700
H	-1.15326900	3.17284500	4.27472100
H	0.00072800	2.48347300	3.12243500
H	-0.81220600	1.43709800	4.28630700
C	-3.40405500	1.90897900	3.39009300
H	-3.33954500	1.11443800	4.13616700
H	-4.22104100	1.67399100	2.70549200
H	-3.65030400	2.84353500	3.90255100
C	-3.28492200	3.26620900	-2.10782700
H	-3.74443000	2.27918200	-2.02114900
C	-2.12404900	3.14799900	-3.09219800
H	-1.65539800	4.12159100	-3.25444600
H	-2.47968500	2.77888600	-4.05738200
H	-1.35859200	2.46202200	-2.72421900
C	-4.35683700	4.21033000	-2.63851600
H	-5.18819300	4.30360700	-1.93742100
H	-4.74872800	3.83489200	-3.58667100
H	-3.95747700	5.20965600	-2.82622800
C	-5.33654400	-1.03112000	-0.18661000
C	-5.51300100	-1.85837000	0.92112800
C	-6.71318900	-2.55992900	0.98730200
H	-6.89260600	-3.21684300	1.83001300

C	-7.67198700	-2.43297100	-0.00103800
H	-8.59929900	-2.98949500	0.07382800
C	-7.45568400	-1.60300500	-1.08911000
H	-8.21423600	-1.51718000	-1.85772600
C	-6.27436300	-0.88300600	-1.20908500
C	-4.46771600	-2.02504200	1.99601900
H	-3.60958500	-1.39004300	1.77197900
C	-5.00461200	-1.59997500	3.35914000
H	-4.20285600	-1.65091500	4.09860800
H	-5.81341600	-2.25575500	3.69311200
H	-5.38555700	-0.57704500	3.33764800
C	-3.95031400	-3.46009200	2.03346300
H	-3.16357100	-3.54574600	2.78270000
H	-3.52708700	-3.75407900	1.07150000
H	-4.74853800	-4.16321500	2.28822700
C	-6.03022500	0.02255400	-2.39267500
H	-4.96570900	0.27135400	-2.41107500
C	-6.81010700	1.32519900	-2.23342100
H	-7.88488800	1.12836100	-2.20042000
H	-6.61483000	1.99518600	-3.07408600
H	-6.53256700	1.83815100	-1.31066400
C	-6.34692200	-0.65992500	-3.71808000
H	-5.79164000	-1.59395600	-3.82494800
H	-6.07456300	-0.00525700	-4.54910400
H	-7.41185400	-0.88354700	-3.81441600
C	2.54583300	2.55070500	0.77114500

C	2.74235300	2.55827600	2.15333500
C	2.43567900	3.74312900	2.81912100
H	2.56429200	3.78832000	3.89302900
C	1.95999800	4.84625800	2.14004800
H	1.71337300	5.75040600	2.68528300
C	1.80402600	4.80906200	0.76437100
H	1.44227200	5.68581800	0.24435000
C	2.10972400	3.66409100	0.04313600
C	3.33066100	1.39514400	2.91893600
H	3.20045200	0.48998400	2.32506400
C	2.64152500	1.13232200	4.25048400
H	3.08709000	0.24933600	4.71324800
H	1.58164200	0.92150800	4.12365600
H	2.76470600	1.96331500	4.95030600
C	4.82485500	1.64685900	3.12917500
H	4.98068700	2.56259400	3.70662800
H	5.35353600	1.74750400	2.18024100
H	5.27079000	0.82188100	3.68959800
C	2.05593200	3.65299200	-1.46782000
H	1.68097500	2.67620700	-1.78187300
C	3.46383700	3.82173300	-2.04043300
H	3.86827000	4.80153800	-1.77274900
H	3.44415200	3.74771700	-3.13085300
H	4.15176600	3.06636900	-1.65847300
C	1.11653100	4.69875000	-2.04596900
H	0.12601200	4.63977300	-1.59210300

H	1.01023100	4.54313200	-3.12165100
H	1.50098900	5.71206600	-1.90220800
C	5.25163600	-0.94340200	-0.73304500
C	5.39594100	-2.02802000	0.13280700
C	6.59266900	-2.73234200	0.04873500
H	6.74981100	-3.58148200	0.70297000
C	7.57857100	-2.36715000	-0.85010300
H	8.50287000	-2.93189100	-0.89392400
C	7.39284400	-1.28745700	-1.69710100
H	8.17207700	-1.01504700	-2.39870300
C	6.21620300	-0.54980600	-1.66024000
C	4.33366900	-2.44696000	1.12148400
H	3.45074600	-1.81757500	0.99805100
C	4.81988200	-2.25221000	2.55495700
H	4.00464600	-2.46204600	3.24971500
H	5.15767400	-1.22818900	2.72197900
H	5.65115000	-2.92386300	2.78677600
C	3.89299500	-3.88855500	0.88365900
H	4.71896400	-4.58749000	1.04169300
H	3.51673600	-4.02716700	-0.13103200
H	3.09540400	-4.14781000	1.57983300
C	6.00985500	0.63002600	-2.58027800
H	4.95222700	0.90400000	-2.54544900
C	6.81608700	1.83139100	-2.09299100
H	6.54376400	2.09807500	-1.07009800
H	6.63931200	2.69807200	-2.73464900

H	7.88639900	1.60952800	-2.11049700
C	6.33503400	0.29373300	-4.03108500
H	5.76162300	-0.56860800	-4.37761800
H	7.39592800	0.07173800	-4.16725800
H	6.09381100	1.14328700	-4.67403500
S	0.02312500	-1.39663800	2.64124700
O	-0.07337000	-0.99994600	4.02390700
O	1.22501300	-0.96649100	1.95073500
O	-1.19764500	-1.24744000	1.87147100
C	0.24277900	-3.22527600	2.72288200
F	1.37015300	-3.54173300	3.36856900
F	0.31173800	-3.75716200	1.50100100
F	-0.76733400	-3.81069400	3.36772800

4 - Ref. 14

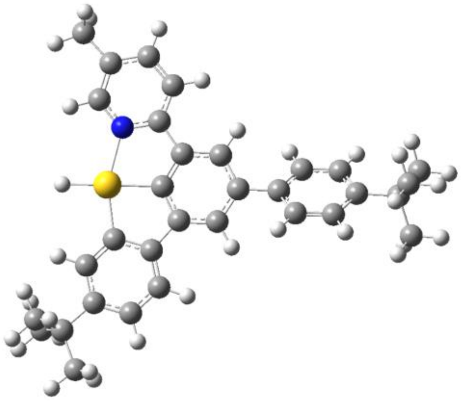

Method: GD3BJ-PBE1PBE/DEF2TZVP

E(RPBE1PBE) = -1429.3773 Hartree

Dipole Moment = 5.3965 Debye

Point Group = C1

Au	-2.98908460	0.68894200	1.78994930
H	-4.11423560	1.10300540	2.91260490
C	-1.58723400	0.19516460	0.45003150
N	-4.18069030	0.16916570	0.06126670
C	-1.34280090	0.99424770	2.90934750
C	-1.98417730	-0.20686200	-0.81076680
C	-1.00141380	-0.54682640	-1.74256030
C	0.34491500	-0.47666580	-1.38357590
C	0.71206600	-0.06440530	-0.09277860
C	-0.25791400	0.27804200	0.83963620
C	-3.43656390	-0.21097520	-1.00825930
C	-4.08077220	-0.56905400	-2.18754440

C	-5.45758610	-0.53465090	-2.26130130
C	-6.21348220	-0.14332710	-1.16009240
C	-5.50812460	0.19917810	-0.01799310
C	-0.12370490	0.72607310	2.23169240
C	1.06566790	0.90441790	2.91686660
C	1.06961760	1.33621040	4.23822860
C	-0.11389730	1.60258760	4.91268210
C	-1.31846150	1.42179030	4.21910690
C	-7.70646290	-0.09017130	-1.19388400
C	-0.15301470	2.07710960	6.35965760
C	1.24147090	2.22403500	6.95825000
C	-0.93501770	1.06417100	7.20140820
C	-0.85193390	3.43852950	6.42566220
H	-2.26033400	1.61978940	4.71449000
H	2.02091780	1.46273740	4.73839320
H	2.00986960	0.70514090	2.42021860
H	1.76598400	-0.04375750	0.16485700
C	1.38792770	-0.83575640	-2.36248650
H	-1.25547970	-0.84097330	-2.75523460
H	-3.49092700	-0.87279290	-3.04230510
H	-5.95503940	-0.81443030	-3.18430940
H	-6.02194660	0.51461850	0.88255880
H	1.78157660	1.27422880	6.96349540
H	1.16048070	2.56540960	7.99314760
H	1.84016610	2.95767650	6.41288120
H	-0.90105740	3.78904440	7.46067270

H	-1.87104720	3.38577500	6.03901050
H	-0.30872810	4.18210130	5.83746610
H	-0.45318350	0.08390080	7.17474700
H	-1.95708750	0.94410910	6.83782920
H	-0.98352730	1.39505590	8.24276150
H	-8.11046990	0.23825810	-0.23588950
H	-8.05881880	0.60178170	-1.96309920
H	-8.13064730	-1.07195600	-1.41961810
C	2.57791320	-0.11206920	-2.44810950
C	3.55456760	-0.45113880	-3.36462980
C	3.39785850	-1.52676410	-4.23966260
C	2.21054500	-2.24515620	-4.15159840
C	1.22653540	-1.90812940	-3.23365630
H	2.72679130	0.74556030	-1.80160710
H	4.45934760	0.14495730	-3.40137980
C	4.50354190	-1.86876810	-5.22963080
H	2.04106190	-3.09579520	-4.79917010
H	0.32710120	-2.51108530	-3.17425940
C	4.15272960	-3.07584820	-6.09276620
C	4.74509380	-0.67049390	-6.15286010
C	5.78971790	-2.18440390	-4.46031460
H	4.97362530	-3.28079020	-6.78377290
H	3.99571010	-3.97316890	-5.48915470
H	3.25376270	-2.89993060	-6.68858200
H	6.59870000	-2.42197150	-5.15662090
H	6.11048580	-1.33879170	-3.84922180

H	5.64539860	-3.04102000	-3.79780770
H	5.54566980	-0.89542080	-6.86262290
H	3.84268120	-0.42850720	-6.71938400
H	5.03536800	0.21904320	-5.59040880

9 - Ref. 14

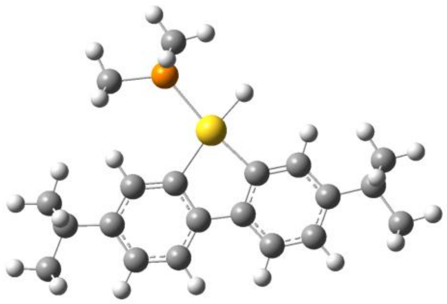

Method: GD3BJ-PBE1PBE/DEF2TZVP

E(RPBE1PBE) = -1373.2211 Hartree

Dipole Moment = 6.3157 Debye

Point Group = C1

Au	-0.54055300	0.12083100	0.66166700
H	-1.42475400	0.39779000	2.00038600
P	-2.64453000	-0.47210600	-0.18591300
C	1.17467500	0.68537100	1.60901500
C	0.80496500	-0.15734800	-0.91057500
C	2.13311300	0.16026300	-0.56269300
C	3.15312400	0.02810500	-1.49793900
C	2.87936000	-0.41732800	-2.78259100
C	1.57935900	-0.73260200	-3.13639900
C	0.55594200	-0.60057100	-2.20119600
C	1.26696800	1.10991900	2.92611700
C	2.49788700	1.47272300	3.46441500
C	3.63960500	1.41051200	2.68269800

C	3.55652700	0.98704500	1.36461400
C	2.33202600	0.62155500	0.81502900
H	-0.44973800	-0.85517300	-2.50911100
H	3.68246500	-0.51681600	-3.50404400
C	2.58092200	1.91515300	4.84276900
H	0.37845600	1.16074700	3.54218500
H	4.60050200	1.69192300	3.09863400
H	4.45806500	0.94290100	0.76365600
H	4.17465300	0.27324800	-1.22867600
C	1.28099300	-1.19922700	-4.47645100
C	-2.92516100	-1.01910900	-1.89558800
C	-3.37385900	-1.81517400	0.79132700
C	-3.81637100	0.90104100	-0.00716500
H	-3.98622900	-1.22621500	-2.05359700
H	-2.60462500	-0.24365900	-2.59221300
H	-2.35162300	-1.92508100	-2.09543200
H	-4.39456700	-2.02815900	0.46580800
H	-2.76517900	-2.71397300	0.68092300
H	-3.37218900	-1.52857500	1.84297500
H	-4.82132300	0.60116800	-0.31231000
H	-3.82525700	1.22548900	1.03331100
H	-3.48784700	1.73936800	-0.62359900
C	3.95800500	2.23411600	5.16588600
C	1.75669700	3.09452400	5.02231900
C	2.11109700	0.85924800	5.71845700
H	4.02036600	2.56639600	6.20107800

H	4.57702500	1.34836800	5.03103800
H	4.31085900	3.02713700	4.50821400
H	2.17345800	1.19152800	6.75364900
H	1.07686000	0.61969500	5.47578600
H	2.73011800	-0.02650000	5.58360900
H	1.81905900	3.42680400	6.05751100
H	2.10955200	3.88754500	4.36464800
H	0.72246100	2.85497200	4.77964700
C	2.50387900	-1.25038900	-5.25390400
C	0.69609400	-2.52424900	-4.40781900
C	0.34464200	-0.28967000	-5.10768100
H	0.47201100	-2.87470100	-5.41424400
H	1.39932600	-3.20735800	-3.93374400
H	-0.22233500	-2.48582500	-3.82392500
H	0.12055800	-0.64012200	-6.11410600
H	-0.57378700	-0.25124600	-4.52378600
H	0.78392100	0.70546700	-5.15922500
H	2.27979600	-1.60084000	-6.26032900
H	2.94315900	-0.25525200	-5.30544900
H	3.20711100	-1.93349700	-4.77982900

9. Photophysical measurements

Quantification of the molar absorption coefficient at different concentrations was carried out on a Perkin Elmer UV/Vis Lambda 465 using 10 mm quartz cells. Emission spectra were acquired on Edinburgh Instruments FS5 Spectrofluorometer using 450 W Xenon lamp excitation at 320 nm and absorption spectra for comparison was carried out on a Shimadzu UV-1900i UV-Vis spectrophotometer. All samples were prepared in anaerobic cuvettes in a glovebox under Argon atmosphere using solvent degassed by at least three freeze-pump-thaw cycles and pressurized with Argon. Complexes **2^{H}** and **3^{H}** showed no emission and their spectra are not included.

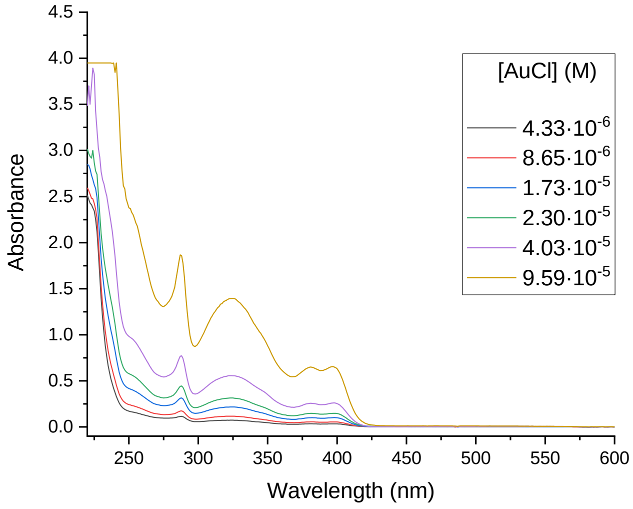

Figure S79. Absorption of compound **1^{H}** in dichloromethane at 298 K in the concentration range 9.59·10^{-5} M – 4.33·10^{-6} M.

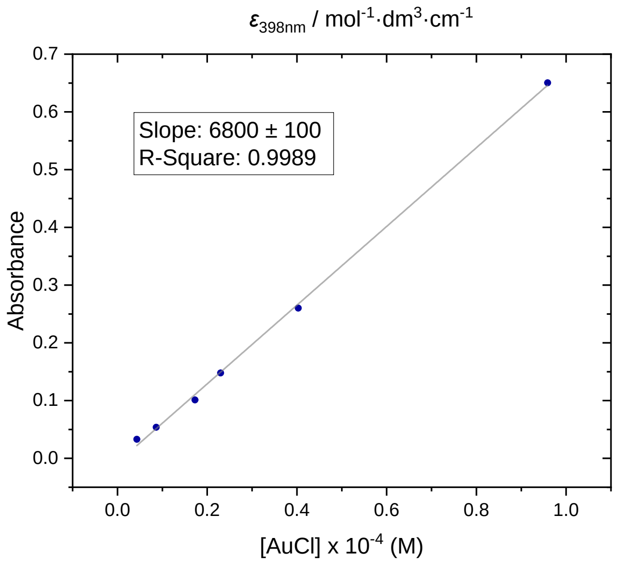

Figure S80. Molar absorption coefficient ε_{398nm} of compound **1^H^** in dichloromethane at 298 K.

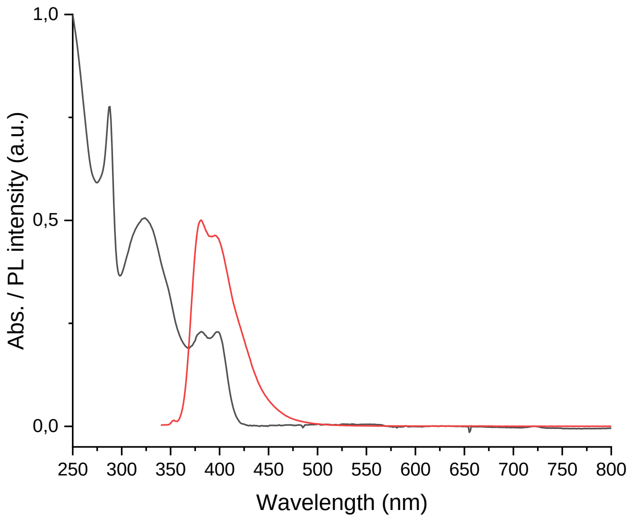

Figure S81. Absorption (in black) and PL spectra (in red, excitation at 320 nm) of **1^H^** in dichloromethane at 298 K.

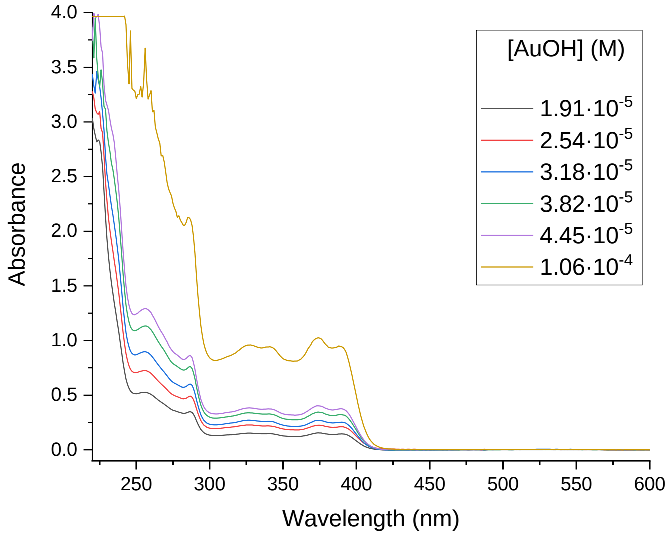

Figure S82. Absorption of compound **2^H** in dichloromethane at 298 K in the concentration range $1.06{\cdot}10^{-4}$ M – $1.91{\cdot}10^{-5}$ M.

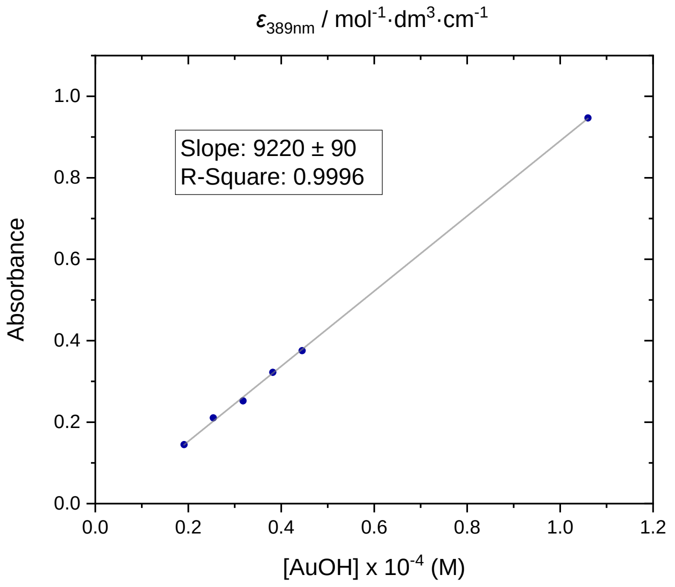

Figure S83. Molar absorption coefficient ε_{389nm} of compound **2^H** in dichloromethane at 298 K.

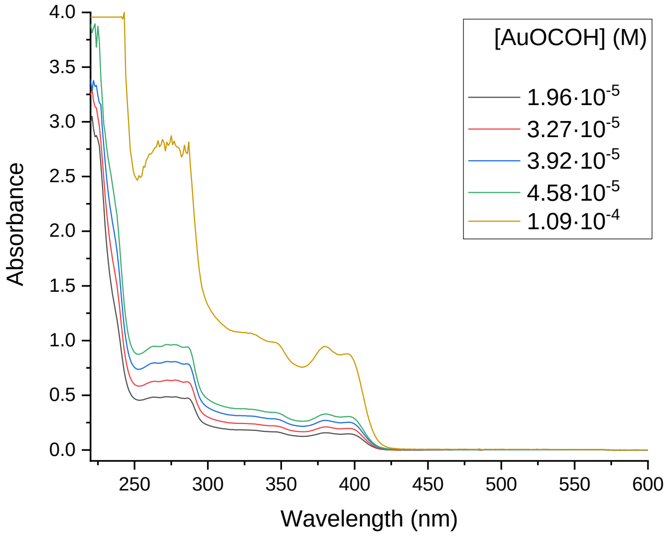

Figure S84. Absorption of compound **3**$^{\mathbf{H}}$ in dichloromethane at 298 K in the concentration range $1.09 \cdot 10^{-4}$ M – $1.96 \cdot 10^{-5}$ M.

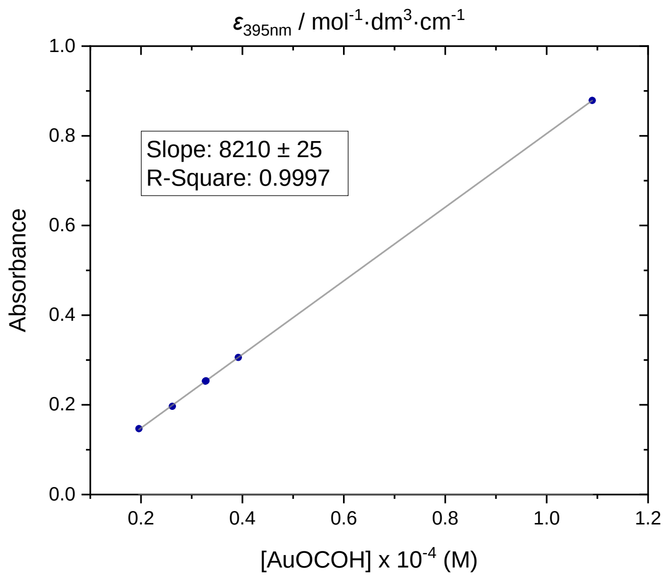

Figure S85. Molar absorption coefficient ε_{395nm} of compound **3**$^{\mathbf{H}}$ in dichloromethane at 298 K.

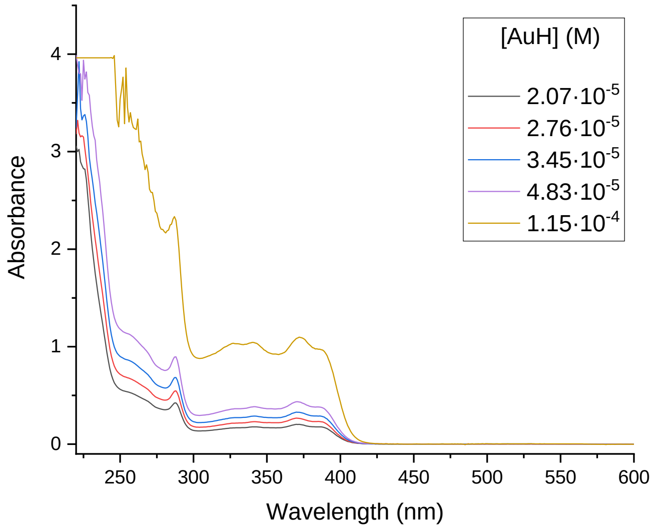

Figure S86. Absorption of compound $\mathbf{4^H}$ in dichloromethane at 298 K in the concentration range $1.15\cdot10^{-4}$ M – $2.07\cdot10^{-5}$ M.

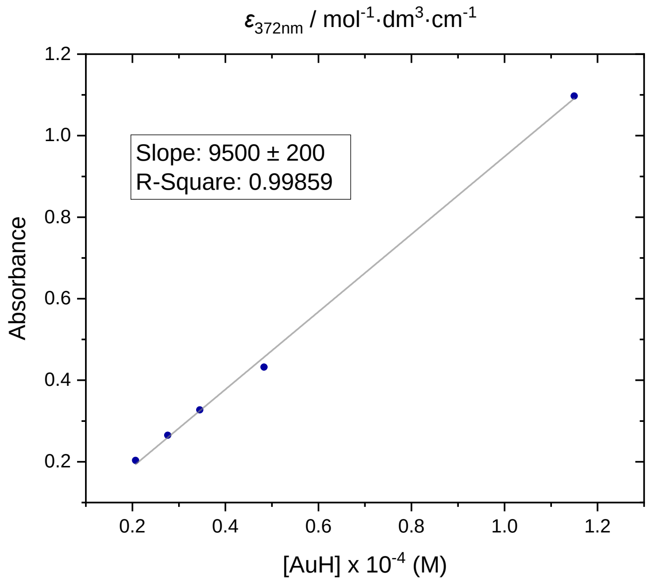

Figure S87. Molar absorption coefficient ε_{372nm} of compound $\mathbf{4^H}$ in dichloromethane at 298 K.

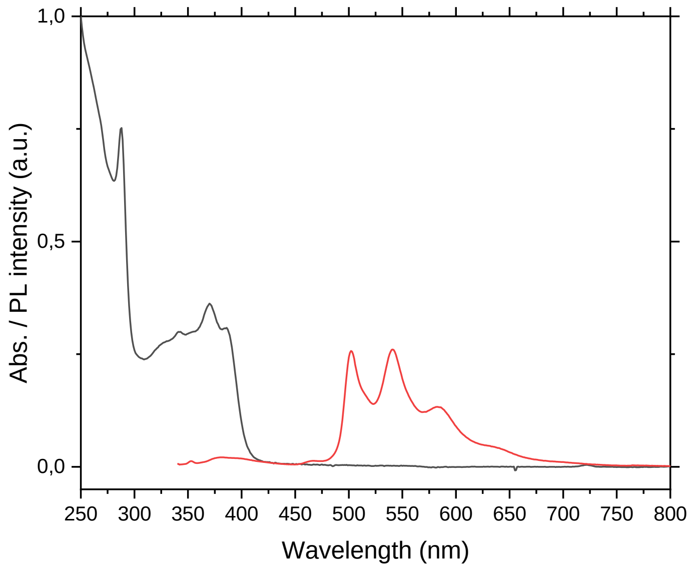

Figure S88. Absorption (in black) and PL spectra (in red, excitation at 320 nm) of $\mathbf{4^{H}}$ in dichloromethane at 298 K.

10. References

(1) Mao, L.; Moriuchi, T.; Sakurai, H.; Fujii, H.; Hirao, T. New tridentate cyclometalated platinum(II) and palladium(II) complexes of N,2-diphenyl-8-quinolinamine: syntheses, crystal structures, and photophysical properties. *Tetrahedron Lett.* **2005**, *46*, 8419-8422.

(2) Diemer, V.; Chaumeil, H.; Defoin, A.; Fort, A.; Boeglin, A.; Carré, C. Syntheses of sterically hindered pyridinium phenoxides as model compounds in nonlinear optics. *Eur. J. Org. Chem.* **2006**, 2727-2738.

(3) (a) Nutton, A.; Bailey, P. M.; Maitlis, P. M. Pentamethylcyclopentadienyl-rhodium and -iridium complexes. Part 29. Syntheses and X-ray structure determinations of [{Rh(C_5Me_5)}$_2$(OH)$_3$]OH·11H_2O and [{Ir(C_5Me_5)}$_2$(OH)$_3$]O_2CMe·14H_2O and related complexes. *J. Chem. Soc., Dalton Trans.* **1981**, 1997-2002; (b) Roesky, H. W.; Singh, S.; Yusuff, K. K. M.; Maguire, J. A.; Hosmane, N. S. Organometallic hydroxides of transition elements. *Chem. Rev.* **2006**, *106*, 3813-3843.

(4) Rigaku Oxford Diffraction, *CrysAlisPro* Software System, Version 1.171.41.105a, Rigaku Corporation, Wroclaw, Poland, 2021.

(5) Coppens, P.; Leiserowitz, L.; Rabinovich, D. Calculation of Absorption Corrections for Camera and Diffractometer Data. *Acta Crystallogr.* **1965**, *18*, 1035-1038.

(6) Sheldrick, G. M. SHELXT - Integrated space-group and crystal-structure determination. *Acta Crystallogr. Sect. A*, **2015**, *71*, 3-8.

(7) (a) Maslen, E. N.; Fox, A. G.; O'Keefe, M. A. in 'International Tables for Crystallography', Ed. A. J. C. Wilson, Kluwer Academic Publishers, Dordrecht, **1992**, Vol. C, Table 6.1.1.1, pp. 477-486; (b) Creagh, D. C.; McAuley, W. J. *ibid*. Table 4.2.6.8, pp. 219-222; (c) Creagh, D. C.; Hubbell, J. H. *ibid*. Table 4.2.4.3, pp. 200-206.

(8) Stewart, R. F.; Davidson, E. R.; Simpson, W. T. Coherent X-Ray Scattering for Hydrogen Atom in Hydrogen Molecule. *J. Chem. Phys.* **1965**, *42*, 3175-3187.

(9) Ibers, J. A.; Hamilton, W. C. Dispersion Corrections + Crystal Structure Refinements. *Acta Crystallogr.* **1964**, *17*, 781-782.

(10) Sheldrick, G. M. Crystal structure refinement with SHELXL. *Acta Crystallogr. Sect. C*, **2015**, *71*, 3-8.

(11) Spek, A. PLATON SQUEEZE: a tool for the calculation of the disordered solvent contribution to the calculated structure factors. *Acta Crystallogr. Sect. C* **2015**, *71*, 9-18.

(12) Parsons, S.; Flack, H. D.; Wagner, T. Use of intensity quotients and differences in absolute structure refinement. *Acta Crystallogr. Sect. B*, **2013**, *69*, 249-259.

(13) Gaussian 16, Revision C.01, Frisch, M. J.; Trucks, G. W.; Schlegel, H. B.; Scuseria, G. E.; Robb, M. A.; Cheeseman, J. R.; Scalmani, G.; Barone, V.; Petersson, G. A.; Nakatsuji, H.; Li, X.; Caricato, M.; Marenich, A. V.; Bloino, J.; Janesko, B. G.; Gomperts, R.; Mennucci, B.; Hratchian, H. P.; Ortiz, J. V.; Izmaylov, A. F.; Sonnenberg, J. L.; Williams-Young, D.; Ding, F.; Lipparini, F.; Egidi, F.; Goings, J.; Peng, B.; Petrone, A.; Henderson, T.; Ranasinghe, D.; Zakrzewski, V. G.; Gao, J.; Rega, N.; Zheng, G.; Liang, W.; Hada, M.; Ehara, M.; Toyota, K.; Fukuda, R.;

Hasegawa, J.; Ishida, M.; Nakajima, T.; Honda, Y.; Kitao, O.; Nakai, H.; Vreven, T.; Throssell, K.; Montgomery Jr., J. A.; Peralta, J. E.; Ogliaro, F.; Bearpark, M. J.; Heyd, J. J.; Brothers, E. N.; Kudin, K. N.; Staroverov, V. N.; Keith, T. A.; Kobayashi, R.; Normand, J.; Raghavachari, K.; Rendell, A. P.; Burant, J. C.; Iyengar, S. S.; Tomasi, J.; Cossi, M.; Millam, J. M.; Klene, M.; Adamo, C.; Cammi, R.; Ochterski, J. W.; Martin, R. L.; Morokuma, K.; Farkas, O.; Foresman, J. B.; Fox, D. J. Gaussian, Inc., Wallingford CT, **2019**.

(14) Rocchigiani, L.; Fernandez-Cestau, J.; Chambrier, I.; Hrobarik, P.; Bochmann, M. Unlocking Structural Diversity in Gold(III) Hydrides: Unexpected Interplay of *cis*/*trans*-Influence on Stability, Insertion Chemistry and NMR Chemical Shifts. *J. Am. Chem. Soc.* **2018**, *140*, 8287-8302.

(15) (a) Perdew, J. P.; Burke, K.; Ernzerhof, M. Generalized gradient approximation made simple. *Phys. Rev. Lett.*, **1996**, *77*, 3865-3568; (b) Perdew, J. P.; Burke, K.; Ernzerhof, M. Generalized gradient approximation made simple (vol 77, pg 3865, 1996). *Phys. Rev. Lett.*, **1997**, *78*, 1396; (c) Adamo, C.; Barone, V. Toward reliable density functional methods without adjustable parameters: The PBE0 model. *J. Chem. Phys*. **1999**, *110*, 6158-6169.

(16) Grimme, S.; Ehrlich, S.; Goerigk, L. Effect of the Damping Function in Dispersion Corrected Density Functional Theory. *J. Comp. Chem*., **2011**, *32*, 1456-1465.

(17) Weigend, F.; Ahlrichs, R. Balanced basis sets of split valence, triple zeta valence and quadruple zeta valence quality for H to Rn: Design and assessment of accuracy. *Phys. Chem. Chem. Phys.*, **2005**, *7*, 3297-3305.

(18) AIM: (a) Bader, R. F. W. (**1990**) Atoms in Molecules: A Quantum Theory. Oxford University Press, Oxford; ELF: (b) Lu, T.; Chen, F. W. Meaning and Functional Form of the Electron Localization Function. *Acta Phys. Chim. Sin*., **2011**, *27*, 2786-2792; LOL: (c) Schmider, H. L.; Becke, A. D. Chemical content of the kinetic energy density. *J. Mol. Struct.: THEOCHEM*, **2000**, *527*, 51-61; $\nabla^2\rho$: (d) Bader, R. F. W.; Johnson, S.; Tang, T. H.; Popelier, P. L. A. The electron pair. *J J. Phys. Chem.*, **1996**, *100*, 15398-15415.

(19) Klein, J.; Khartabil, H.; Boisson, J. C.; Contreras-García, J.; Piquemal, J. P.; Henon, E. New Way for Probing Bond Strength. *J. Phys. Chem. A*, **2020**, *124*, 1850-1860.

(20) Fradera, X.; Austen, M. A.; Bader, R. F. W. The Lewis model and beyond. *J. Phys. Chem. A*, **1999**, *103*, 304-314.

(21) (a) Mayer, I.; Salvador, P. Overlap populations, bond orders and valences for 'fuzzy' atoms. *Chem. Phys. Lett.,* **2004**, *383*, 368-375; (b) Matito, E.; Poater, J.; Sola, M.; Duran, M.; Salvador, P. Comparison of the AIM delocalization index and the Mayer and Fuzzy atom bond orders. *J. Phys. Chem. A*, **2005**, *109*, 9904-9910.

(22) Lu, T.; Chen, F. W. Multiwfn: A multifunctional wavefunction analyzer. *J. Comput. Chem.*, **2012**, *33*, 580-592.

(23) Thom, A. J. W.; Sundstrom, E. J.; Head-Gordon, M. LOBA: a localized orbital bonding analysis to calculate oxidation states, with application to a model water oxidation catalyst. *Phys. Chem. Chem. Phys*., **2009**, *11*, 11297-11304.
